# A novel cancer-associated fibroblast signature for kidney renal clear cell carcinoma via integrated analysis of single-cell and bulk RNA-sequencing

**DOI:** 10.1007/s12672-024-01175-x

**Published:** 2024-07-26

**Authors:** Ling Lu, Huaguo Feng, Guohua Dai, Shuangquan Liu, Yi Feng, Haoyang Tan, Xian Zhang, Guoqing Hong, Xing Lai

**Affiliations:** 1 Department of Hepatobiliary Surgery, Tongnan District People’s Hospital, No. 189, Jianshe Road, Dafo Street, Tongnan District, Chongqing, China; 2 Department of Renal Rheumatology Immunology, School of Medicine, Chongqing University Jiangjin Hospital, Chongqing University, Chongqing, China; 3 Department of Hepatobiliary Surgery, School of Medicine, Chongqing University Jiangjin Hospital, Chongqing University, Chongqing, China; 4 Department of Hepatobiliary Surgery, Jiangjin District Maternal and Child Health Hospital, Chongqing, China; 5 Chongqing Traditional Chinese Medicine Hospital, Chongqing, China

**Keywords:** Kidney renal clear cell carcinoma, Cancer-associated fibroblast, Single-cell RNA-sequencing, Prognosis signature

## Abstract

Cancer-associated fibroblasts (CAFs), integral components of the tumor microenvironment, play a pivotal role in tumor proliferation, metastasis, and clinical outcomes. However, its specific roles in Kidney Renal Clear Cell Carcinoma (KIRC) remain poorly understood. Employing the established Seurat single-cell analysis pipeline, we identified 21 CAFs marker genes. Subsequently, a prognostic signature consisting of 6 CAFs marker genes (*RGS5*,* PGF*,* TPM2*,* GJA4*,* SEPT4*, and *PLXDC1*) was developed in a cohort through univariate and LASSO Cox regression analyses. The model’s efficacy was then validated in an external cohort, with a remarkable predictive performance in 1-, 3-, and 5-year. Patients in the high-risk group exhibited significantly inferior survival outcomes (*p* < 0.001), and the risk score was an independent prognostic factor (*p* < 0.05). Distinct differences in immune cell profiles and drug susceptibility were observed between the two risk groups. In KIRC, the PGF-VEGFR1 signaling pathway displayed a notable increase. PGF expression was significantly elevated in tumor tissues, as demonstrated by quantitative real-time polymerase chain reaction. In vitro, transwell assays and CCK8 revealed that recombinant-PGF could enhance the capability of cell proliferation, migration, and invasion in 769P and 786-O cells. This study firstly developed a novel predictive model based on 6 CAFs genes for KIRC. Additionally, PGF may present a potential therapeutic target to enhance KIRC treatment.

**Supplementary Information:**

The online version contains supplementary material available at 10.1007/s12672-024-01175-x.

## Introduction

Kidney cancer is a leading cause of cancer-related mortality, with kidney renal clear cell carcinoma (KIRC) being the predominant histological subtype, constituting 70–85% of all kidney cancers [1]. Despite the advances in treatment strategies for KIRC, such as targeted therapies, radical or partial nephrectomy, immunotherapy, and personalized treatment approaches, the overall survival (OS) of patients still remains unsatisfactory [2]. Due to the absence of prominent clinical symptoms in the early stages of kidney cancer, a significant number of patients are diagnosed at advanced stages. Traditional prognosis methods, such as TNM staging systems, fail to fully capture the complexity of the tumor microenvironment (TME), thus limiting their effectiveness. With the rapid advancement of biomedical technology, especially single-cell RNA-sequencing (scRNA-seq), the development of a novel survival risk stratification based on gene expression profile for KIRC patients could offer additional insights into tumors and even enable personalized treatment strategies. For instance, Hu et al. employed scRNA-seq techniques to analyze intra-tumoral heterogeneity within KIRC tissue, uncovering a correlation between T cell exhaustion in the TME and a poor prognosis [3].

TME refers to the microenvironment around tumor cells, including surrounding blood vessels, immune cells, fibroblasts, bone-marrow-derived inflammatory cells, various molecules, and extracellular matrix, crucial for tumor initiation, invasion, and therapeutic response [4]. Cancer-related fibroblasts (CAFs) form the primary components of TME in solid tumors, and their plasticity and interconvertibility give rise to various functions, including the inhibition or promotion of tumor angiogenesis [5]. Studies have demonstrated that CAFs is mainly involved in tumor promotion and associated with poor clinical outcomes, and such functions could be seen in gastric, breast, or colon cancer [6–8]. CAFs could promote tumorigenesis, angiogenesis, and drug resistance through various mechanisms, including shaping the tumor immune microenvironment, metabolic reprogramming, and the generation of extracellular matrix components [9–11]. Recently, some researchers have explored targeting CAFs as a potential therapeutic strategy for malignant tumors, suggesting a novel strategy for cancer treatments [9, 11, 12]. Nevertheless, there is a limited number of researches about the role of CAFs in KIRC.

This study aimed to investigate the molecular characteristics of CAFs maker genes in KIRC and construct a novel survival-related risk stratification model. Additionally, the key biological functions of these marker genes were further elucidated through bioinformatics analysis and validated through experiments.

## Results

### Identification of different cell-type and CAFs maker genes in KIRC

**Fig. 1:**
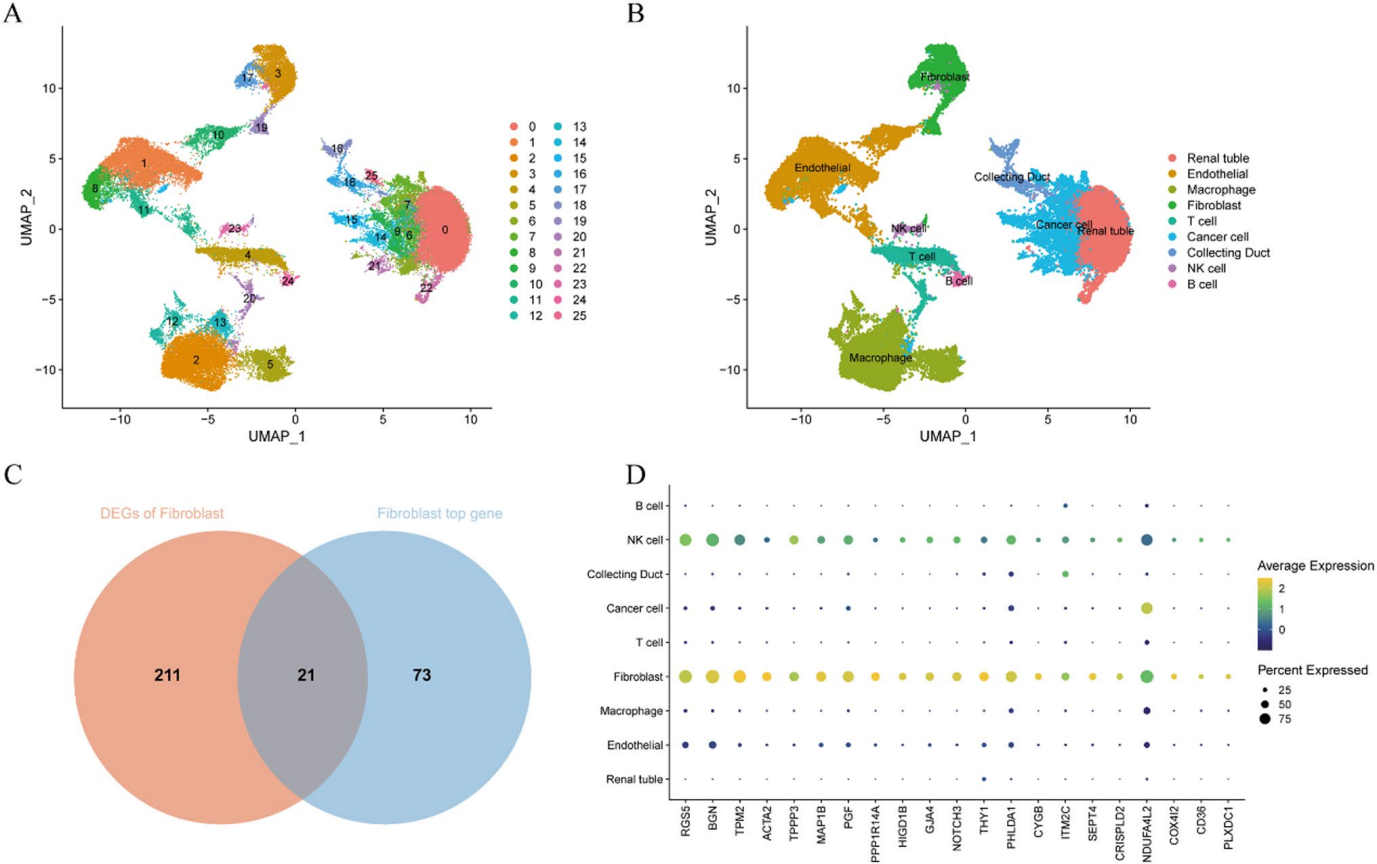
**A **UMAP plot of 54,776 profiled cells from 7 KIRC samples and 5 adjacent normal samples, and 26 clusters were presented. **B** 9 cell types were identified using their marker genes. **C **Venn diagram displaying the CAFs related selected intersection genes from different datasets.** D **Expression of CAFs marker genes in each cell type

In the scRNA-seq data from the GSE156632 dataset, 54,776 cells from 7 KIRC and 5 adjacent normal samples were included for subsequent analysis after data preprocessing. We conducted dimension reduction analysis using the UMAP method, identifying 26 clusters (Fig. 1A). Each cluster was annotated using reference data from Hu et al. and the CellMarker database, identifying cells in clusters 3, 17, and 19 as fibroblasts (Fig. 1B) [3]. 21 genes were observed in the distinct gene expression profiles of tumor and normal tissues, classifying it as CAFs marker genes (Fig. 1C). These 21 genes, as shown in Fig. 1D, exhibited higher expression compared to other cell types.

### Clustering analysis of KIRC based on the 21 CAFs marker genes

**Fig. 2:**
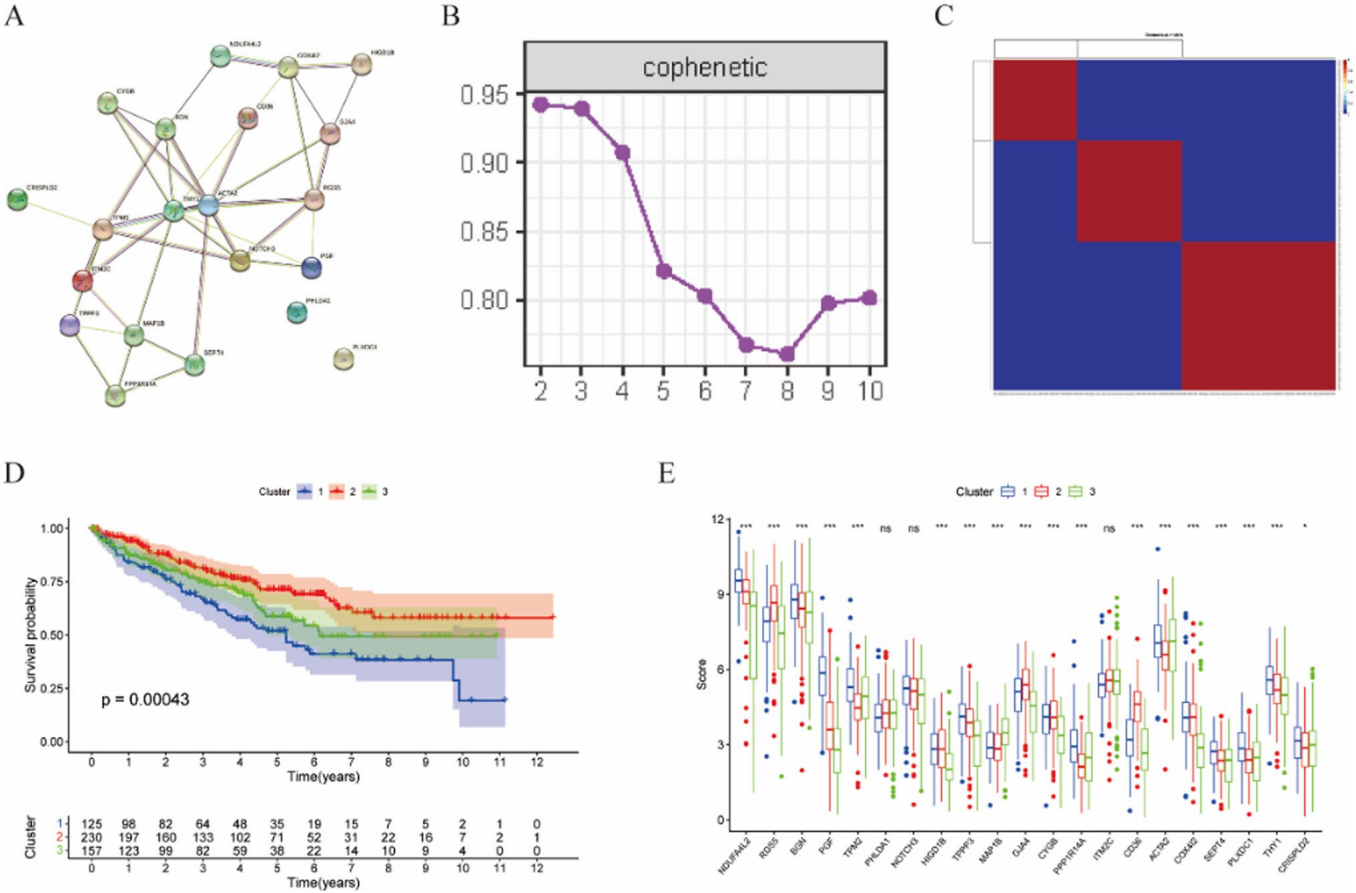
**A **The protein–protein interactions of 21 CAFs marker genes in KIRC from STEING database. **B **NFM clustering using 21 CAFs marker genes. The cophenetic correlation coefficient for k = 2–10 was shown. **C **Consensus clustering matrix for k = 3. **D** The Kaplan–Meier survival curve of three subclusters. **E **The gene expression profile of 21 CAFs marker genes in three subclusters

To assess the prognostic significance of the 21 CAFs marker genes, we conducted a clustering analysis of KIRC. As shown in Fig. 2A, these marker genes exhibited strong interconnections. Using NMF consensus clustering analysis, we clustered a metadata set comprising 512 KIRC patients from TCGA based on the expression profiles of the 21 CAFs marker genes. In Fig. 2B, C, k = 3 was determined as the optimal number of clusters, ensuring stable and robust clustering, categorizing the patients into three clusters. Figure 2D demonstrated that, in comparison to clusters one and three, patients in cluster two had a significantly longer survival (*p* = 0.00043), highlighting the considerable prognostic value of these CAFs marker genes. Furthermore, we analyzed the expression profiles of the 21 CAFs marker genes in KIRC patients (Fig. 2E). With the exception of *PHLDA1*, *NOTCH3*, and *ITM2C*, the remaining CAFs marker genes exhibited significant differential expression in the three clusters (*p* < 0.05).

### The establishment of a prognostic signature based on CAFs marker genes

**Fig. 3:**
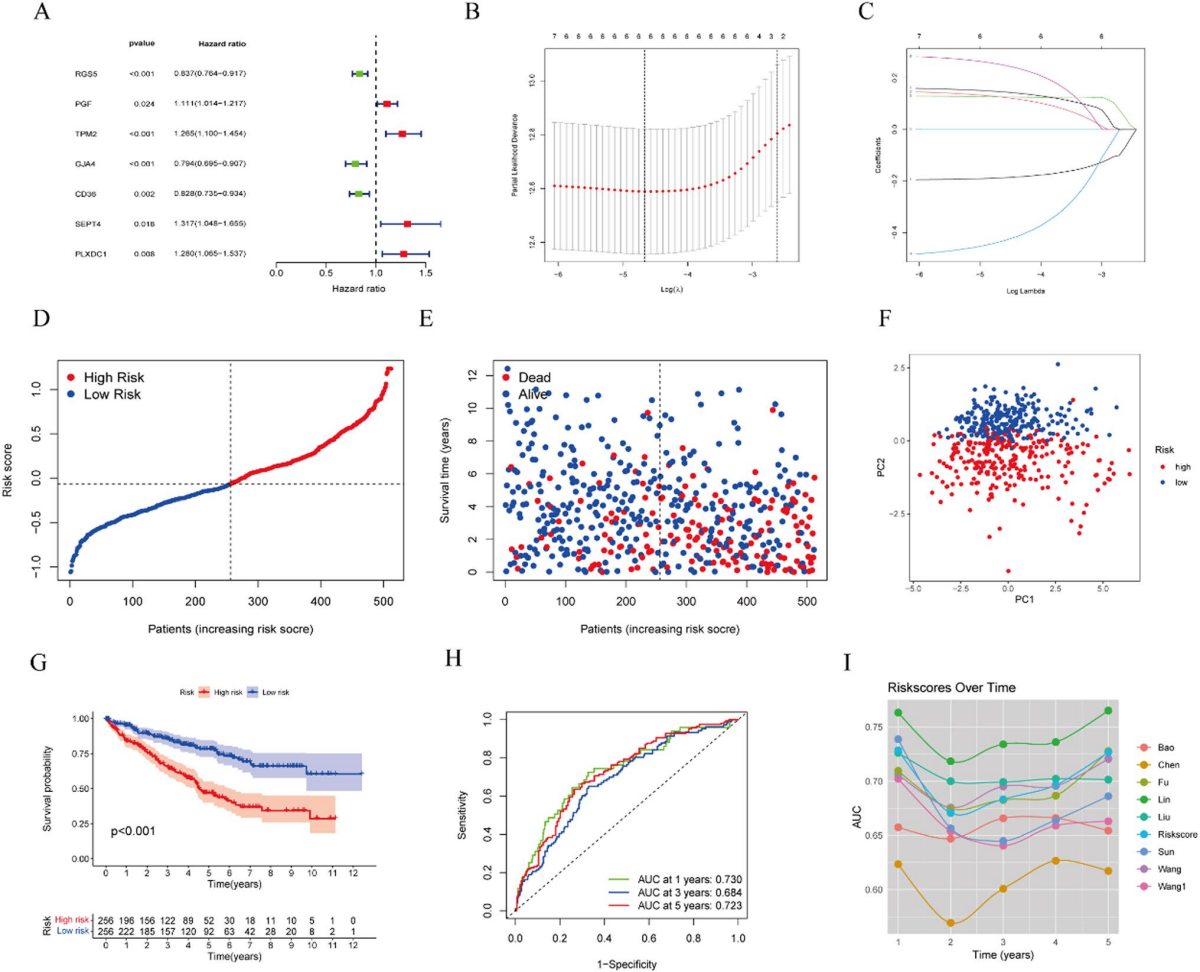
Construction of a CAFs marker gene prognostic signature in KIRC patients. **A **Univariate Cox regression analysis of 7 CAFs marker genes (P < 0.05). **B** LASSO coefficient profiles of CAFs marker genes. **C** Selection of the penalty parameter (λ) in the LASSO model via tenfold cross-validation. **D** The distribution and median value of risk scores in the training set. **E** The distribution of survival status and the risk score. **F **PCA plot of the TCGA KIRC cohort. **G** Kaplan–Meier curves for the OS of patients with KIRC in the high- and low-risk groups. **H **AUC of time-dependent ROC curves to evaluate the predictive performance of the risk model. **I** Comparison of the risk model and eight existing model

To establish a survival-related risk stratification model based on 21 CAFs marker genes, the TCGA KIRC cohort was set to the training dataset. Firstly, a univariate analysis of the 21 CAFs marker genes revealed that 7 genes (*RGS5*, *PGF*, *TPM2*, *GJA4*, *SEPT4*, *PLXDC1*, and *CD36*) were significantly associated with survival outcomes (Fig. 3A, *p* < 0.05). The LASSO Cox regression analysis with one standard error and tenfold cross-validation was performed to construct the survival-related risk stratification model using the expression profile of the seven marker genes mentioned above. Based on the optimal value of the penalty parameter (λ, Fig. 3B, C), we established a prognostic signature containing 6 CAFs marker genes. The risk score of each person was calculated as follows: risk score = (− 0.187 × *RGS5* expression) + (0.126 × *PGF* expression) + (0.127 × *TPM2* expression) + (0.252 × *SEPT4* expression) + (0.146 × *PLXDC1* expression) + (− 0.434 × *GJA4* expression). In Fig. 3D and 512 patients of the training dataset were classified into two groups (a high-risk and a low-risk). As shown in Fig. 3E, the patients with KIRC in the high-risk group had a high-risk of dying earlier than those in the low-risk group. The PCA plot (Fig. 3F) indicated that the patients in the two risk groups were distributed in different directions. Additionally, Kaplan–Meier analysis showed that the patients in the low-risk group, had a significantly better survival outcome (Fig. 3G, *p* < 0.001). The predictive performance was evaluated using time-dependent ROC curves. The AUC of this risk model was 0.730 at 1 year, 0.684 at 2 years, and 0.723 at 5 years (Fig. 3H).

To assess further the effectiveness of our risk model, though a comprehensive literature review, we compared the predictive performance of eight existing models (Fig. 3I) [13–19]. Surprisingly, the AUCs of six risk models were lower than those of our model, except for the Lin’s and Liu’s models. These results indicate that our model has good predictive performance in KIRP.

### Validation of the prognostic signature in the ArrayExpress cohort

**Fig. 4:**
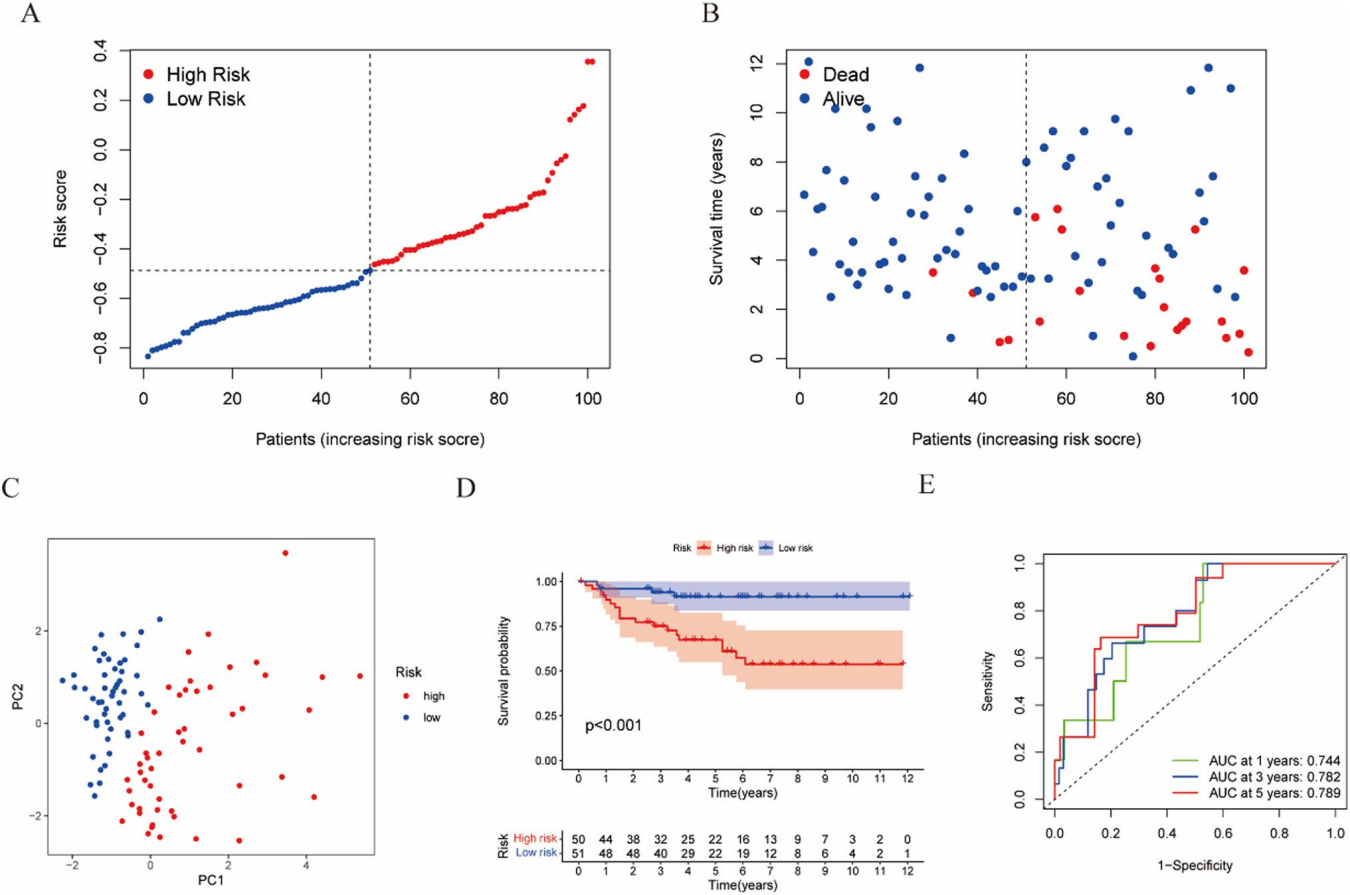
Validation of the CAFs marker gene prognostic signature in the E-MTAB-1980 cohort. **A** The distribution and median value of risk scores in the validation set. **B** The distributions of survival status and the risk score. **C** PCA plot of the E-MTAB-1980 cohort. **D** Kaplan–Meier curves for patients with KIRC in the high- and low-risk group. **E** AUC of time-dependent ROC curves in E-MTAB-1980 cohort

To assess the robustness and performance of this risk stratification model, patients in the E-MTAB-1980 cohort were divided into high- and low-risk groups based on median risk scores, which were calculated using the same formula (Fig. 4A). Similar results were obtained from this prognostic risk model, and the PCA plot proved that the patients in the two risk groups were distributed in two directions (Fig. 4C). Likewise, the Kaplan–Meier analysis demonstrated that patients in the low-risk group had a longer survival time (Fig. 4D), and patients with higher risk scores were more likely to die earlier (Fig. 4B). Meanwhile, the AUC of this risk model in the E-MTAB-1980 cohort was 0.744 at 1 year, 0782 at 3 years, and 0.789 at 5 years, indicating a strong prognostic performance (Fig. 4E).

### Independent prognostic value of the risk stratification model for KIRC patients

**Fig. 5:**
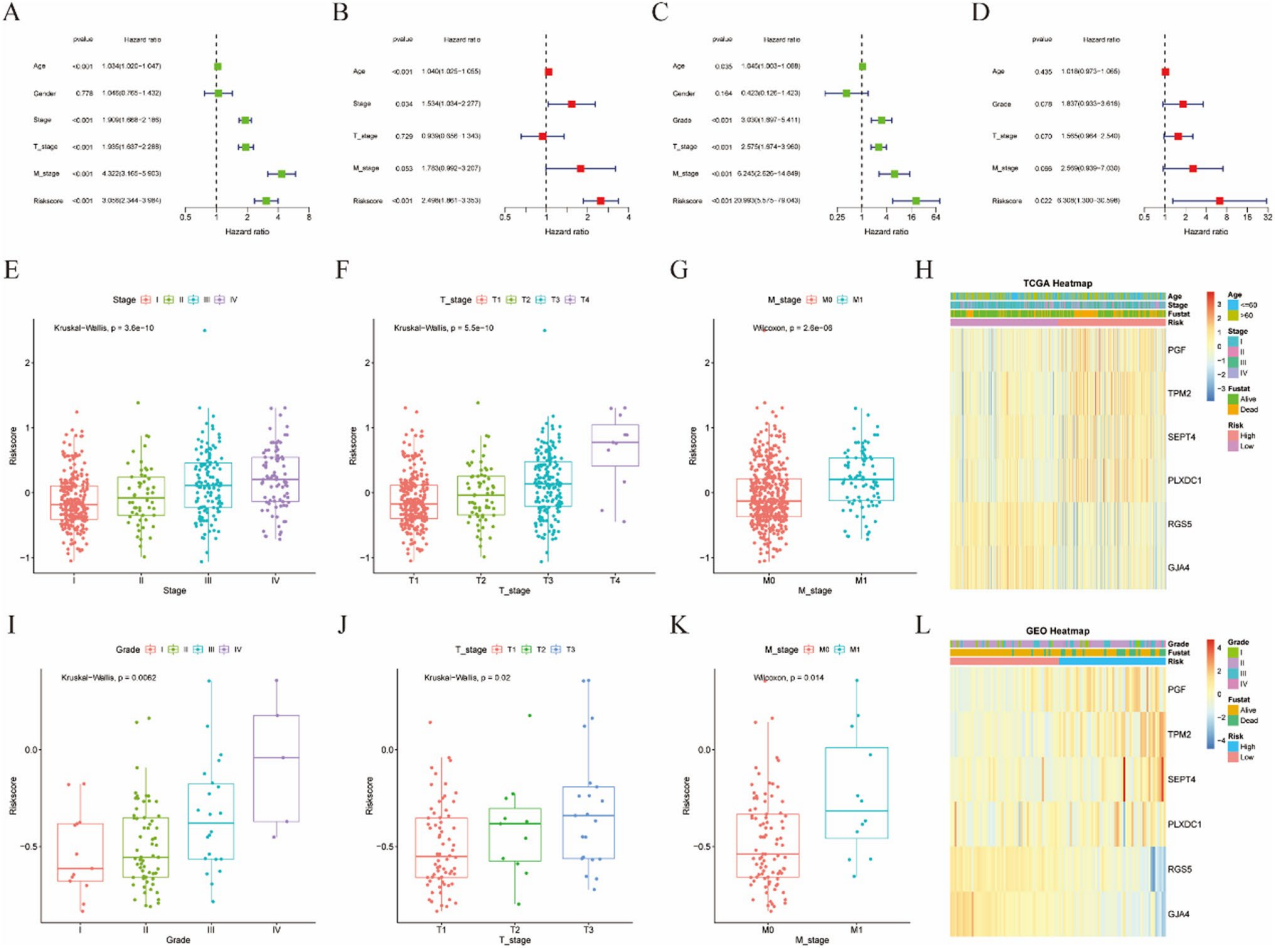
**A**, **B** Univariate and multivariate Cox regression analyses of risk scores and other clinical features in the TCGA cohort. **C**, **D** Univariate and multivariate Cox regression analyses of risk scores and other clinical features in E-MTAB-1980 cohort. **E**, **G** The relationships between risk scores and KIRC stage, T-stage, or M-stage in the TCGA cohort. **I**–**K** The relationships between risk scores and KIRC stage, T-stage, or M-stage in the E-MTAB-1980 cohort. **H**, **L** Heatmap of the expression profile of 6 CAFs marker genes and corresponding clinical features in the TCGA and E-MTAB-1980 cohorts

To investigate whether the risk score of the model independently influenced the prognosis of KIRC patients, univariate and multivariate Cox regression analyses were used. In the univariate Cox regression analysis, the risk score was significantly associated with survival outcomes in both TCGA and E-MTAB-1980 cohorts (Fig. 5A, C; HR: 3.056, 95% CI 2.344–3.984; HR: 20.993, 95% CI 5.575–79.043, respectively). After adjusting for other variables, the risk score was identified as an independent prognostic factor in the multivariate Cox regression analysis of the TCGA and E-MTAB-1980 cohorts (Fig. 5B, D; HR: 2.498, 95% CI 1.861–3.353; HR: 6.308, 95% CI 1.300–30.598, respectively). Subsequently, we observed that patients with high-risk scores had a higher possibility associated with poor clinical stages and pathological staging (Fig. 5E–G and I–K). In Fig. 5H, L, the four CAFs marker genes (*PGF*,* TPM2*,* SEPT4*, and *PLXDC1*) exhibited significantly higher gene expressions in the high-risk group, while RGS5 and GJA4 were upregulated in the low-risk group.

### The functional enrichment analysis

**Fig. 6:**
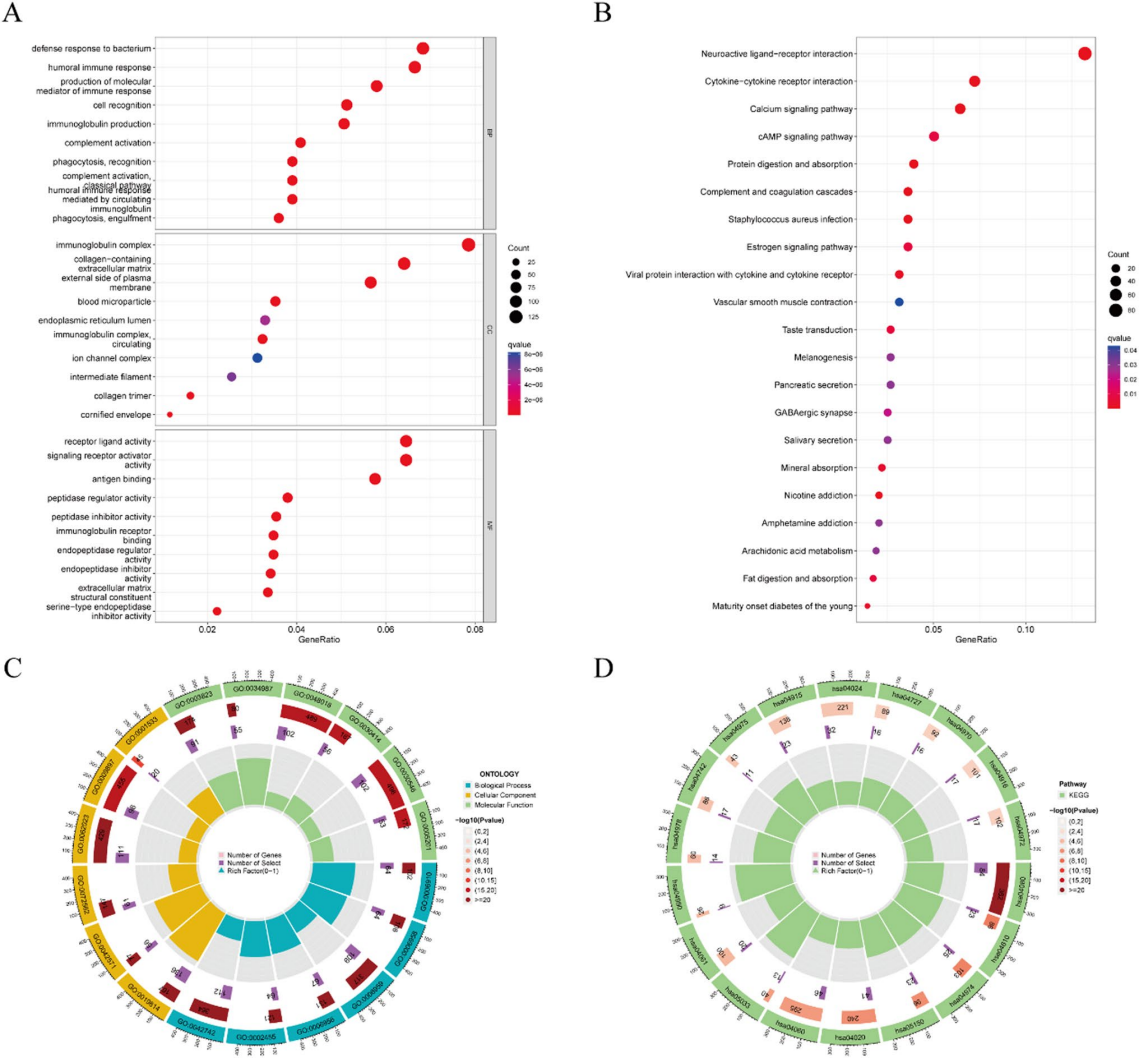
Enrichment analyses of DEGs in the low-risk and high-risk groups. **A** GO analysis. **B** KEGG analysis of the DEGs. **C**, **D** Circle plots of the results of **C** GO and **D** KEGG analyses

We then analyzed DEGs between the two risk groups in to explore differences in biological behaviors and pathways through KEGG and GO analysis. A total of 2517 DEGs were identified. In Fig. 6A, the top three biological processes were defense response to bacteria, humoral immune response, and production of molecular mediators of immune response. Regarding cellular components, the top categories were immunoglobulin complexes, collagen-containing extracellular matrix, and the external side of the plasma membrane. Additionally, highly enriched molecular functions were receptor ligand activity, signaling receptor activator activity, and antigen binding. The KEGG pathway analysis, as shown in Fig. 6B, indicated that the neuroactive ligand receptor interaction, cytokine-cytokine receptor interaction, and calcium signaling pathways were closely associated with these DEGs. And the Circle plots in Fig. 6C, D provided more detailed information on the GO and KEGG analyses.

### Immune characteristic analysis based on the risk score in KIRC

**Fig. 7:**
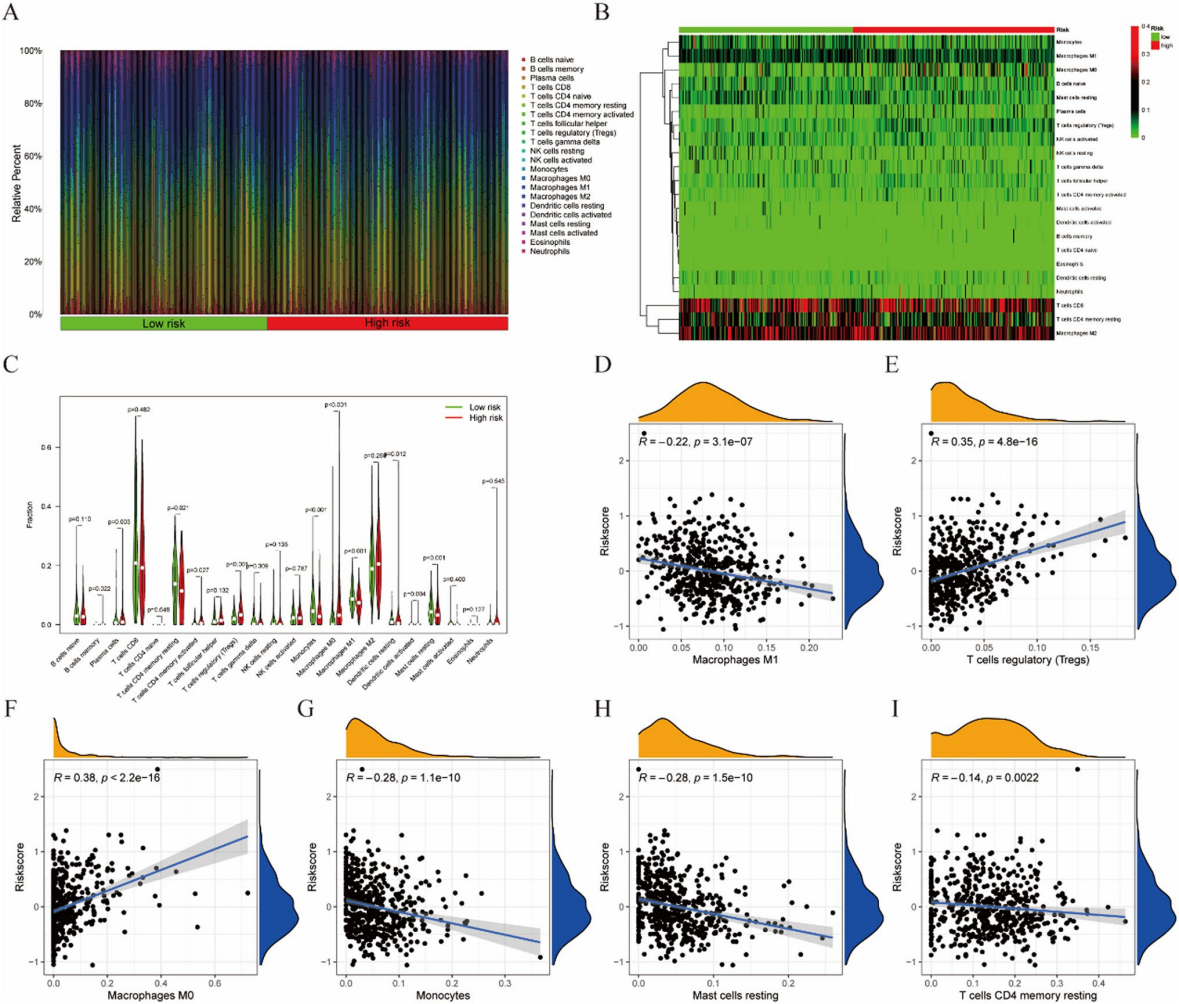
**A** The overview of 22 immune cells in 512 patients with KIRC. **B** Heatmap of 22 immune cells in the high- and the low-risk groups. **C** The fractions of different immune cells between the high- and low-risk groups. **D**–**I** The association between the risk score and immune cell infiltration (p < 0.05)

Subsequently, we investigated the correlation between the risk score and immune status in the 512 patients with KIRC. The relative proportions of the 22 infiltrated immune cells were shown in Fig. 7A, B. Compared to the low-risk group (Fig. 7C), the high-risk group exhibited significantly elevated infiltration levels of memory B cells (*P* < 0.05), plasma cells (*P* < 0.05), activated CD4 memory T cells (*P* < 0.05), regulatory T cells (*P* < 0.05), M0 macrophages (*P* < 0.01), resting dendritic cells (*P* < 0.05), and activated dendritic cells (*P* < 0.05). In contrast, resting CD4 memory T cells (*P* < 0.05), monocytes (*P* < 0.01), and M1 macrophages (*P* < 0.001) were downregulated in the high-risk group. Additionally, Spearman rank correlation analysis (Fig. 7D, I) revealed that the risk score positively correlated with regulatory T cells (*R* = 0.35, *P* < 0.001) and M0 macrophages (*R* = 0.38, *P* < 0.001). Conversely, other immune cells, including M1 macrophages (*R* = − 0.22, *P* < 0.001), monocytes (*R* = − 0.28, *P* < 0.001), resting mast cells (*R* = − 0.28, *P* < 0.001), and resting CD4 memory T cells (*R* = − 0.14, *P* < 0.005), exhibited negative correlations with risk scores.

### Drug sensitivity analysis of the CAFs signature in KIRC

**Fig. 8:**
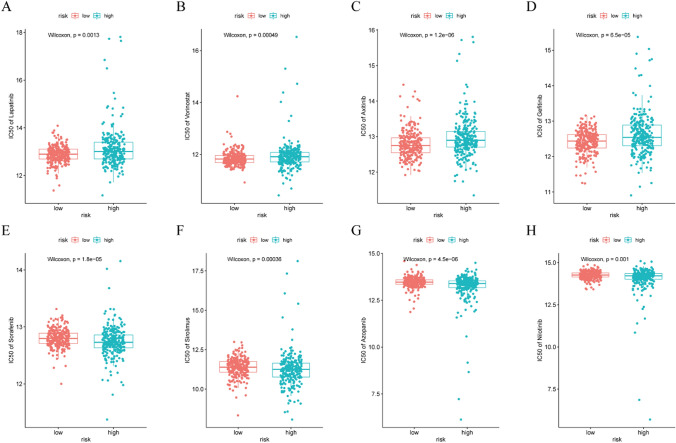
Drug susceptibility analysis of targeted therapy in KIRC between the high- and low-risk groups. **A** Laptinib. **B** Vorinostat. **C** Axitinib. **D** Gefitinib. **E** Sorafenib. **F** Sirolimus. **G** Azopanib. **H** Nilotinib

To explore whether the risk model’s possible clinical application values existed in the personalized treatment of KIRC, the IC50 values of 545 drugs for the two risk groups were calculated using the CTRP dataset as the training dataset. The results of the drug sensitivity analysis revealed that the high-risk group had higher IC50 values for lapatinib, vorinostat, axitinib, and gefitinib, indicating a decreasing benefit from these drugs (Fig. 8A, D). Conversely, the high-risk group had lower IC50 values for sorafenib, sirolimus, axitinib, and gefitinib, suggesting enhanced potential benefits from these drugs compared to the low-risk group (Fig. 8E, H).

### Performance of PGF as a prognostic gene in KIRC

**Fig. 9:**
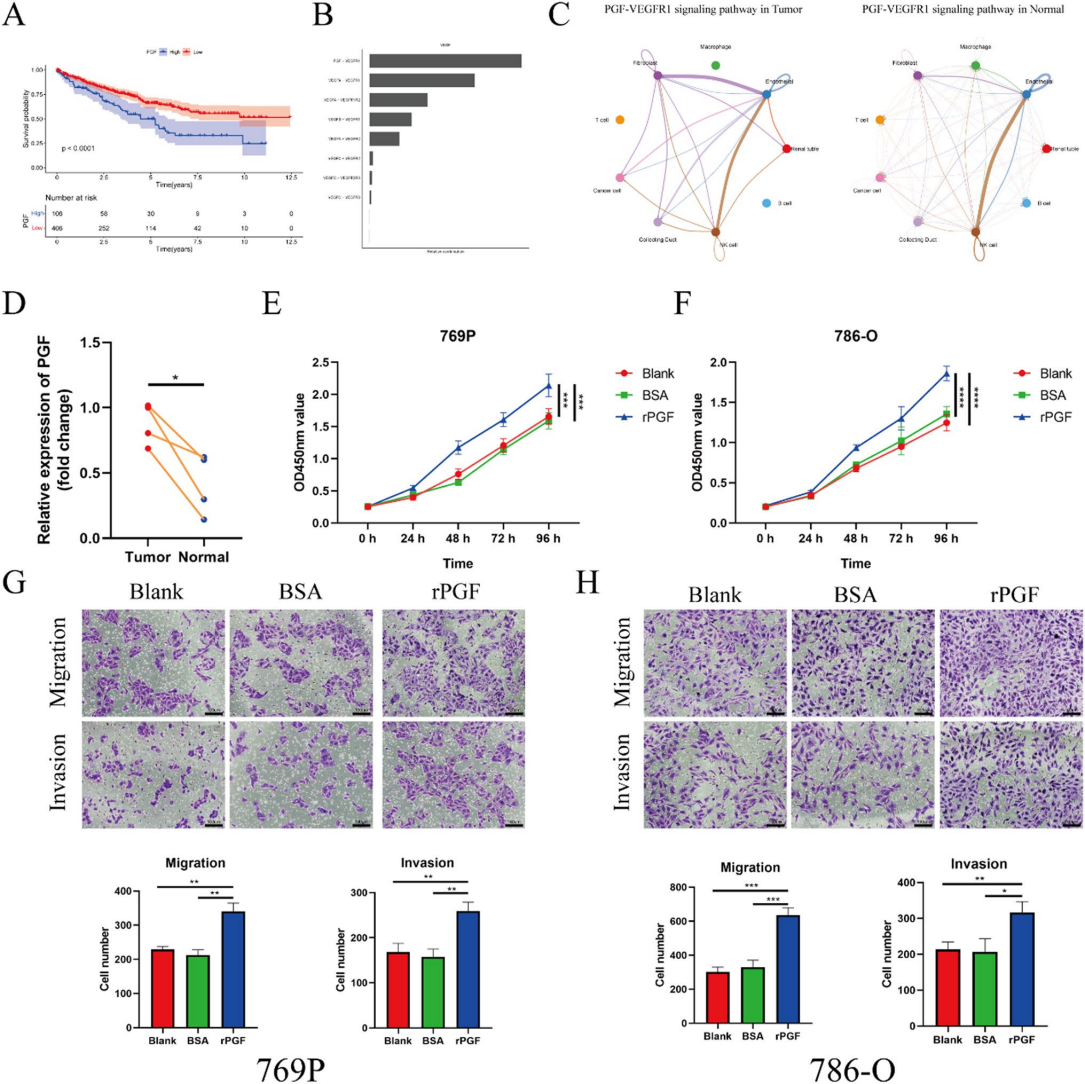
**A** The Kaplan–Meier curve of PGF expression in the TCGA-KIRC cohort. **B** The relative contribution of each ligand-receptor pair in the VEGF signaling pathway. **C** Hierarchical plot showing multiple cell clusters interactions via the PGF-VEGFR1 signaling in the KIRC and normal tissue. **D** The relative PGF mRNA level between KIRC and adjacent normal tissues assessed via qPCR. **E**, **F** Cell proliferation was analyzed by CCK8 assay in 769P and 786-O cells. **G**, **H** Cell migration and invasion were measured by transwell assays. *P < 0.05; **P < 0.01; ***P < 0.001; ****P < 0.0001

For investigating the roles of these CAFs marker genes in the progression of KIRC, we explored the potential molecular functions. In Fig. 9A, the Kaplan-Meier survival analysis showed that patients with high expression of PGF exhibited poorer survival outcomes (*P* < 0.001), suggesting that PGF might be a prognostic gene. Ligand-Receptor analysis showed that the PGF-VEGFR1 made the most significant relative contributions to the overall communication network of VEGF signaling (Fig. 9B), which was further explored. As showed in Fig. 9C, the communication probability of PGF-VEGFR1 in KIRC tissues significantly elevated among fibroblasts, endothelial cells, and cancer cell. The result of qPCR (Fig. 9D) showed that the mRNA expression of PGF also elevated in KIRC. Thus, we inferred that PGF played an important role in this cancer. Functionally, recombinant-PGF promoted cell proliferation in 769P cells and 786-O cells as showed in Fig. 9E, F. In addition, recombinant-PGF enhance the capability of cell migration and invasion (Fig. 9G, H). Taken together, these data showed PGF exerted its tumor-promoting in KIRC.

## Discussion

The crosstalk of CAFs and other stromal or cancer cells in the TME complex causes contrasting roles in tumor development, containing tumor-restraining and tumor-promoting functions, and such effects could also influence the therapeutic response [9]. The application of scRNA-seq technologies enabled a further understanding of the CAFs molecular characteristics and heterogeneity in tumors. In this study, the CAFs marker genes were identify in KIRC through scRNA-seq analysis. Subsequently, a novel risk stratification model based on 6 CAFs marker genes was developed in the TCGA cohort, which was further validated using an external cohort,. Lastly, PGF may serve as a potential therapeutic target for improving KIRP treatments.

Recently, Liu et al. revealed that patients with KIRC with a high infiltration level of CAFs associated significantly associated with poor survival outcomes and advanced pathological stages based on the specificity of CAFs gene signature [20]. Likewise, similar results were found in this 6-gene signature related to CAFs marker genes, implying that our risk stratification model deserves attention. For the CAFs genes with the constructed signature, Regulator-of-G-protein-signaling-5 (RGS5), a member of the B/R4 sub-family of RG-protein, is encoded by the *RGS5* gene and involved in various biological processes [21]. Silini et al. showed that *RGS5* could serve as a novel marker of cancer pathological angiogenesis in ovarian cancer, assisting tumor progression [22]. Furthermore, another research observed that *RGS5*^+^ CAFs in an epithelial ovarian subgroup supported the tumor cell metastasis with poor relapse-free survival [3]. Conversely, in our research, we found that elevated *RGS5*^+^ in KIRC correlated closely with better prognosis. Similar results have also been observed in lung cancer and other renal cell carcinoma research [23, 24]. Connexin 37 is encoded by the *GJA4* gene, and is involved in cell gap junctions and intercellular communication [25]. The *GJA4* gene had double effects on the tumor, including promoting tumor cell proliferation and suppression [26, 27]. In this study, patients with the high *GJA4* mRNA related significantly to improved survival outcomes, as seen in another research [25]. *TPM2* gene encodes a thin filament-associated protein playing a crucial role in muscle contraction, motility, and cell-matrix interactions, which is a specific gene in the fibroblast [28]. The *TPM2* gene, with high clinical relevance had been regarded as a poor prognostic biomarker in some studies, including human colon cancer, endometriosis, and prostate cancer, which supported our results [28–30]. However, the mechanism and signaling path of *TPM2* require further exploration. *SEPT4* gene belongs to the septin family of nucleotide-binding proteins, which could encode multiple protein isoforms. ARTS is a product of the *SEPT4* gene, inducing apoptosis via degradation of XIAP and Bcl-2 [31]. Bongiovanni et al. reported that high *SEPT4* mRNA expression increased the risk of transitional cell bladder cancer [32]. In our study, *SEPT4* expression was also upregulated in the high-risk group. Additionally, *PLXDC1* is a well-researched gene that participates in angiogenesis. Upregulated *PLXDC1* in various tumors, for example, gastric cancer and glioblastoma, has a poor survival outcome [33]. Combined with our results, *PLXDC1* could not only be a biomarker for poor outcomes but also for tumor anti-angiogenesis. In short, these genes mentioned above need further exploration.

PFG belongs to the pro-angiogenic vascular endothelial growth factor family, and its high expression is linked to tumor pathological angiogenesis [34, 35]. Chen et al. found that the *PGF* overexpression in gastric carcinoma increased the chance of lymph-node metastasis and decreased the survival time [36]. Additionally, inhibiting *PGF* could prolong patients’ survive time with metastatic colorectal cancer [37]. In our study, we obtained the similar survival outcomes, and then we validated that the PGF was upregulated in KIRC tissues from the protein expressional level. Notable, we observed that the communication probability of PGF-VEGFR1 signaling between the fibroblast and endothelial cells significantly promoted in KIRC tissue, and that might the mechanism of fibroblast involved in pathological angiogenesis. Considering our results, we speculated that upregulated *PGF* could be a prognostic risk factor and serve as a potential therapeutic target.

Some studies have highlighted the role of CAFs in reshaping the tumor immune microenvironment, mainly suppressing the antitumor immunity activity via various mechanisms and influencing the immunotherapy [38, 39]. In another research regarding renal cell carcinoma, Xu et al. reported that the differential infiltration of CD248^+^ CAFs correlated closely with survival time [12]. Therefore, we explored the infiltrated immune cells of TME. In the high-risk group, the infiltrated levels of B cells, plasma cells, activated memory CD4 T cells, regulatory T cells, M0 macrophages, resting dendritic cells, and activated dendritic cells increased significantly increased. Meanwhile, the resting CD4 memory T cells, monocytes, and M1 macrophages were upregulated in the low-risk group. Studies have confirmed that activated memory CD4 T cells could secret interleukin 17, which promotes tumor progression and is associated with inferior survival outcomes [40]. By contrast, Chen et al. reported better survival outcomes with high infiltration of activated memory CD4 T cells [41]. Activated dendritic cells stimulate the formation of M2 macrophages to secret some cytokines, such as IL-6, CXCL8, VEGF, and TGF-β, which could suppress the adaptive immune response and promote tumor growth [42]. Meanwhile, activated dendritic cells also directly expand the regulatory T cells [43]. Paluskievicz et al. summarized that regulatory T cells promoted angiogenesis and assisted tumor immune escape via cell binding or contact-independent mechanisms [44]. Also, regulatory T cells in TME are often associated with poor prognosis [45]. Furthermore, macrophages were differentiated into M1 and M2 macrophages. Among them, M1 macrophages are involved in the inhibition of tumor growth mainly through three aspects: presenting antigens to T cell receptors, recruiting chemokines, and activating nature killing cells [46]. Therefore, the 6 CAFs related genes could alternate the clinical prognosis by influencing the tumor immune microenvironment.

Subsequently, drug susceptibility analysis for KIRC was conducted based on the risk score. The differentiation of drug effects in various risk groups could provide an important reference for decision-making. Given that CAFs of TME are associated with inferior clinical prognosis in multiple cancers, targeting CAFs seems like a promising therapeutic strategy. Currently, some approaches have been taken to target CAFs. First, targeting the upstream of CAFs induces fibroblasts to differentiate and reprogram into tumor suppressive subtype [4]. Secondly, inhibiting the downstream of the CAFs signaling pathway (e.g., TGF-β and CXCL12/CXCR4) is another method [5]. Third, selecting a CAFs population for target therapy might receive a better clinical benefit, such as FAP^+^ CAFs [47]. However, due to the CAFs’ plasticity and the complexity in TME, the therapeutic response of targeting CAFs is poor and should be further explored in the future.

Notably, some limitations of our study deserve more consideration. Firstly, although KIRC is a common tumor type, the sample size of the TCGA might impact the robustness of our risk model, despite validation in the E-MTAB-1980 cohort. Secondly, potential biases in patient selection, such as ethnic diversity or different stages of KIRC, might influence the outcomes of this risk model. Third, due to the limited clinical cohort, some larger retrospective or prospective clinical researches are needed to confirm the predictive prognosis value of this risk-stratified model based on the 6 genes and its guiding role in the decision-making of drug selections. Lastly, the mechanism of these 6 genes in KIRC should be further explored in experiments.

In conclusion, a novel prognostic risk model based CAFs maker genes was constructed by integrated scRNA-seq and bulk RNA analyses in KIRC. These findings offer new insights of CAFs into the TME and provide potential targets for further research and clinical interventions.

## Materials and methods

### Data collection

The scRNA-seq data from 7 KIRC samples and matched 5 adjacent normal samples of the GSE156632 were downloaded from the GEO database. Additionally, bulk RNA-seq expression data from 535 KIRC samples and corresponding clinical information were sourced from The Cancer Genome Atlas (TCGA) database. 512 samples were finally included in our study based on the merged sample quality annotations for constructing survival-related risk stratification model. The E-MTAB-1980 dataset (*n* = 101 KIRC samples), was set as a validation dataset, sourced from the ArrayExpress database. The RNA-seq data were normalized using fragments per kilobase of transcript per million mapped reads and log2-transformed for subsequent analyses.

### Identification of CAFs marker genes using scRNA-seq analysis

Following the Seurat single-cell analysis standard workflow, we generated Seurat objects separately for the 7 KIRC samples and 5 adjacent normal samples. To maintain high-quality scRNA-seq data, cells with fewer than 100 measured genes, over 15% mitochondrial contamination, or over 5000 measured genes were excluded. 54,776 high-quality cells were included for further analysis.

The merged object underwent normalization using the ‘NormalizeData’ function in the ‘Seurat’ R package, and batch effect correction for the 12 samples was performed using the ‘Harmony’ R package. Dimension reduction analysis was carried out using the uniform manifold approximation and projection (UMAP) method, with the top two UMAP dimensions from 20 harmony dimensions visualized at a clustering resolution of 0.5. 26 clusters were annotated into 9 cell clusters using Hu et al.‘s cell-specific marker annotations and the CellMarker databases [3]. |Log2FC| > 1 and P value < 0.05 were defined as differentially expressed genes (DEGs) in fibroblasts between tumor and normal samples. Additionally, 94 DEGs were identified in fibroblast clusters across all samples using the ‘FindAllMarkers’ function using the ‘Seurat’ R package. The 21 CAFs genes which were their intersection were considered as CAFs marker genes, and their protein-protein interaction network was obtained from Search Tool for the Retrieval of Interacting Genes (https://www.string-db.org/, version 11.5) (Supplementary Table 1).

### Non negative matrix factorization clustering based on the 21 CAFs marker genes

Non-negative matrix factorization (NMF), commonly employed for clustering high-dimensional data, is a well-established data analysis technique [48] ‘NMF’ R package was used to identify new patient subgroups in KIRC, determining k = 3 as the optimal number of clusters. Subsequently, Kaplan–Meier survival analysis and differential gene expression profiles of the 21 CAFs marker genes was conducted in the three clusters using the ‘limma’ R package.

### Construction and validation of the prognostic risk model related to CAFs marker genes

We employed Univariate Cox regression analysis to identify 7 of the these CAFs marker genes as prognostic genes in the TCGA cohort. The optimal number of DEGs was selected using a tenfold cross-validation of LASSO-penalized Cox regression analysis with the ‘glmnet’ R package. The prognostic signature was constructed based on the expression profiles of 6 genes (*RGS5*, *PGF*, *TPM2*, *GJA4*, *SEPT4*, and *PLXDC1*) and their corresponding coefficients derived from the LASSO Cox regression model, with the penalty parameter (λ) determined according to the minimum criteria.

The risk score of each patient was calculated as follows: Risk score= ${\sum}_{i}^{6}Xi*Yi$ (X: coefficients, Y: gene expression level). To remove batch effects between the TCGA and E-MTAB-1980 cohorts, all gene expression data were centralized and standardized using the “Scale” function. Patients in the TCGA cohort (512 patients) and the E-MTAB-1980 cohort (101 patients) were divided into high-risk and low-risk groups separately, based on the median value of the risk score. Kaplan–Meier curves were employed to analyze OS between the two risk groups. Additionally, we used the area under the curve (AUC) of receiver operating characteristic (ROC) curves to evaluate the performance of the risk model in predicting 1-, 3-, and 5-year prognoses.

### Independent prognostic analysis of the clinical features and risk score

We analyzed the prognostic value of risk score along with other clinical features, including age, sex, gender, TNM grade, and tumor stage, using univariate and multivariate Cox regression models in the TCGA and E-MTAB-1980 cohorts.

In addition, we performed DEGs enrichment analysis between the low-risk group and the high-risk group using the “clusterProfiler” R package (Supplementary Table 2). The Gene Ontology (GO) and Kyoto Encyclopedia of Genes and Genomes (KEGG) were the primary enrichment methods to evaluate functional differences [49].

### Evaluation of the infiltrated immune cells and correlation analysis

The 22 infiltrated immune cells in 512 KIRC TME were assessed using the “CIBERSORT” R package (Supplementary Table 3). Then, the “limma” R package was used to evaluate the differences in the 22 infiltrated immune cells between the two risk groups. Additionally, the relationships between the infiltrated immune cells and risk scores were analyzed using the Spearman’s rank correlation analysis.

### Drug sensitivity analysis based on the risk score

The Cancer Therapeutics Response Portal (CTRP), containing drug sensitivity and molecular marker information regarding multiple cancer types, was set as the training dataset. With the ‘oncoPredict’ R package, we built a ridge regression modeling CTRP. Subsequently, we predicted the half-maximal inhibitory concentration (IC50) for 545 drugs in KIRC based on sensitivity scores.

### The cell–cell communication

The ‘‘CellChat’’ R package was applied to identify and visualize the cell cross-talk among cell clusters based on the Jin’s strategy [50]. The PGF-VEGFR1 pathway was further explored.

### Quantitative PCR

Four freshly isolated KIRC tissue were acquired from Tongnan District People’s Hospital, immediately immersed in liquid nitrogen. The total RNA was extracted using the RNA-Quick Purification Kit (YiShan Biotech), followed by reverse transcribed into complementary DNA (cDNA) using the RT Master Mix (MCE) according to the manufacturer’s instructions. Quantitative real-time polymerase chain reaction (qPCR) was conducted on the Bio-Rad CFX96 qPCR system, utilizing the SYBR RT-PCR kit sourced from MCE. To ensure consistency, all samples were normalized based on β-actin expression levels. The primer sequence for PGF was list as following: Forward: 5′-ACGGCTCGTCAGAGGTGGAAG-3′; Reverse: 5′-GAGACACAGGATGGGCTGAACATG-3′. Following data acquisition, the gene expression level was determined using the 2^−ΔΔCT^ method.

### CCK-8 assay

Cell Proliferation was assessed by Cell Counting Kit-8 (CCK8) assays (GlpBio Company Ltd). Briefly, the 769P and 786-O cells (Procell) at a density of 2 × 10^3^ cells/well were seeded into 96-well plates and were cultured with complete medium in 5% CO_2_. Then, CCK8 reagent, according to the manufacturer’s instructions, was added at 0, 24, 48, 72, and 96 h after being cocultured with or without recombinant-PGF. The absorbance of each well was recorded at 450 nm via a microplate reader.

### Transwell assays

Transwell assays were performed to detect the migration and invasion capabilities of 769P and 786-O cells. The invasion ability was determined by Boyden chambers were pre-coated with a basement membrane matrix, and those without coating were used for migration assays. The 2 × 104 cells (769P or 786-O) were plated within the top chamber containing 100 µL of serum-free medium, and 10% FBS in the lower chamber was served as the chemoattractant. After 24 h incubation with control BSA or with 100 ng/mL recombinant-PGF (HY-P74627A, MCE), non-migrated cells in the upper chamber were removed softly by a cotton swab. Subsequently, cells on the lower surface of membranes were fixed with 4% paraformaldehyde and stained with 0.1% crystal violet. Fields were randomly selected in each well using an inverted light microscope at magnification of 100×, and cell numbers were quantified using Image J software.

### Statistical analysis

The statistical analysis and generated visualization maps were generated by the R software (v4.2.1) or GraphPad Prism v9.0. Methods employed included Student’s t-test, the Kaplan–Meier method with a two-sided Log-rank test, and Spearman’s rank correlation analysis. Statistical significance was set at *P* < 0.05 or adjusted *P* < 0.05 for all analyses.

### Supplementary Information

Below is the link to the electronic supplementary material.

Supplementary file 1 (PDF 46 KB)

Supplementary file 2 (PDF 459 KB)

Supplementary file 3 (PDF 261 KB)

## Data Availability

The TCGA-KIRC and GSE156632 datasets were downloaded from the TCGA database (https://portal.gdc.cancer.gov/) and GEO database (https://www.ncbi.nlm.nih.gov/geo/); the E-MTAB-1980 dataset used in this study was obtained from the ArrayExpress database (https://www.ebi.ac.uk/arrayexpress/).
