# Supplementary material for: A novel cancer-associated fibroblast signature for kidney renal clear cell carcinoma via integrated analysis of single-cell and bulk RNA-sequencing
Source: Discov Oncol. 2024 Jul 26;15:309. doi: 10.1007/s12672-024-01175-x (PMC11282037; doi:10.1007/s12672-024-01175-x)
Supplement: Supplementary file 1 — (PDF 46 KB) [file 12672_2024_1175_MOESM1_ESM.pdf]

Supplementary Table 1. The CAF marker genes

DEG of Fibroblast	Fibroblast top gene	CAF marker genes
SMIM24	RGS5	RGS5
LGALS2	TAGLN	BGN
MIOX	BGN	TPM2
PCK1	TPM2	ACTA2
MT1G	MYL9	TPPP3
ASS1	MGP1	MAP1B
GLYAT	ACTA2	PGF
PDZK1IP1	PLAC9	PPP1R14A
FABP1	COL1A2	HIGD1B
FBP1	COL1A1	GJA4
PRAP1	CALD1	NOTCH3
GLYATL1	TIMP1	THY1
GATM	COL3A1	PHLDA1
MT1H	IGFBP71	CYGB
GSTA1	ID31	ITM2C
GSTA2	FRZB	SEPT4
PRODH2	TPPP3	CRISPLD2
PCSK1N	MAP1B	NDUFA4L2
ALDOB	PGF	COX4I2
HPD	C11orf961	CD36
PAH	PPP1R14A	PLXDC1
ALB	HIGD1B	
NAT8	LHFP1	
CXCL14	MFGE8	
FXYD2	GJA4	
RBP5	NOTCH3	
RP11-626P14.1	COL6A2	
BHMT	SOD3	
ASPDH	THY1	
PDZK1	PHLDA1	
MT1F	COL18A11	
MGST1	PLN	
BBOX1	TINAGL11	
RAB11FIP3	CPE	
UGT2B7	FN11	
DPEP1	LGALS11	
HRSP12	CYGB	
TMEM27	PDGFRB	
APOM	MYH11	
HAO2	ADIRF1	
KHK	TPM1	
PLA2G2A	RGS16	
C16orf89	ITM2C	
PI16	MYLK1	
SUCLG1	SEPT4	
AK4	MCAM1	
C11orf54	CRISPLD2	
RP11-536018.1	NDUFA4L2	
SLC7A7	COX4I2	

TTR	RASD1
SLPI	FXYD61
DCXR	DKK3
ALDH6A1	CD36
GCHFR	NR2F21
SPP1	ID11
CYB5A	SLIT3
PPP1R16A	CSRP1
CRYL1	SPARC1
SFRP1	COL6A1
DAB2	DSTN1
ACAA1	HES41
NDUFA4L2	KCNE4
DEFB1	SDC2
OGN	ISYNA1
MT1X	PMEPA11
GPX3	ADAMTS11
ECHS1	PTN
IGF1	OLFML2A1
TMEM176A	FILIP1L
RGS5	TGFB1I1
SH3YL1	ID4
GAS1	IFITM31
BGN	TPM41
PTGDS	COL4A21
CTSH	SEPT7
SFRP4	ITGB11
AKR1A1	SELM
FTL	MTHFD2
MT-ND4L	PLXDC1
APOE	TBX2-AS1
RARRES1	ARHGEF17
MFAP5	PRSS231
MT1E	S1PR3
ACAT1	PTP4A3
TMSB4X	OAZ21
APOD	FOS2
MT-ND4	EBF1
ALDH2	JUNE1
IL1RL1	KANK2
SCRG1	PALLD
MT-CO3	JAG11
C7	HEYL
ADI1	ITGA11
GCSH	GEM
ATP1B1	CTGF1
OCIAD2	
CLU	
DNPH1	
MT-ND3	
PEPD	

TMEM176B
MT-ND5
NDUFA4
RPS19
RPS3
PGF
ARHGDIB
B2M
RPL23A
SFRP2
RARRES2
TNXB
MSRA
RPS2
MALAT1
USP53
NDUFA3
ITM2B
SAT2
TSTD1
MT-CO2
RPL15
C3
LAMTOR5
HLA-B
PRDX3
MIF
DHRS4L2
PEBP1
EFEMP1
CFD
PCOLCE2
SRPX
PLCG2
MEG3
MFAP4
ID3
UQCRQ
ADH1B
VIM
ATP5I
PRDX1
ETFB
COX7C
ATPIF1
TXN
TPM2
COL18A1
HLA-C
SOD1
PHLDA1

C12orf75
LAPTM4A
PRDX6
COL4A2
FBLN1
SEPP1
FBLN5
HBA2
IGF2
ATP5J2
NOTCH3
HIGD1B
RPLP0
NDUFC1
TPPP3
DCN
LDHB
UQCR10
MAP1B
C11orf96
NDUFB1
CCDC80
COL4A1
THBS1
S100A2
MCAM
S100A13
GJA4
EPAS1
HES4
SPARCL1
FXYD6
PMEPA1
CYGB
PPP1R14A
FN1
HSPA1B
OLFML2A
OAZ2
MTRNR2L12
LINC00152
CTD-3252C9.4
ANXA2
IGFBP5
ITM2C
CD36
C1R
SERPINF1
ACTA2
COX4I2
SEPT4

MYH9
HBB
IGFBP2
ADM
C1S
PLXDC1
THY1
CCL5
CCL4
VWF
APOLD1
ANGPT2
RGS1
CRISPLD2
CCL3
PLVAP
ADAMTS4
CCL4L2
RGS2
NKG7
CTGF
FABP5
S100A10
IGFBP6
CYR61
C1QA
C1QB
PCOLCE
FBN1
CCL2
