# Supplementary material for: A novel cancer-associated fibroblast signature for kidney renal clear cell carcinoma via integrated analysis of single-cell and bulk RNA-sequencing
Source: Discov Oncol. 2024 Jul 26;15:309. doi: 10.1007/s12672-024-01175-x (PMC11282037; doi:10.1007/s12672-024-01175-x)
Supplement: Supplementary file 2 — (PDF 459 KB) [file 12672_2024_1175_MOESM2_ESM.pdf]

ementary Table 2. The DEGs between the low-risk group and the high-risk

gene_id	baseMean	log2FoldCh	lfcSE	stat	pvalue	padj
PAEP	148.95698	-6.181029	0.2754288	-22.44148	1.55E-111	3.40E-107
ADAMTS14	210.35554	-2.430502	0.1151553	-21.10629	6.96E-99	1.02E-94
VSTM2L	105.22505	-3.749588	0.1858078	-20.17992	1.47E-90	1.61E-86
SLC38A5	869.89387	-3.276321	0.1653612	-19.81311	2.29E-87	1.44E-83
LBP	3728.0645	-5.001168	0.2609327	-19.16651	7.05E-82	3.86E-78
PTPRH	202.19595	-3.470496	0.1828486	-18.98016	2.49E-80	1.21E-76
COL7A1	620.80025	-2.885015	0.1525421	-18.91291	8.93E-80	3.91E-76
APCDD1L	312.23548	-3.503561	0.185449	-18.89231	1.32E-79	5.25E-76
NIPAL4	33.247563	-3.210614	0.1711593	-18.75804	1.66E-78	6.08E-75
MTND1P23	503.75432	-3.82769	0.2049557	-18.67569	7.81E-78	2.63E-74
HS3ST3A1	61.680975	-3.011904	0.1656203	-18.1856	6.71E-74	2.10E-70
TNNT1	157.75409	-3.828953	0.2109168	-18.15386	1.20E-73	3.50E-70
PI3	95.56551	-3.720155	0.2062967	-18.03303	1.07E-72	2.94E-69
ADAM12	982.77661	-2.330246	0.1292838	-18.02427	1.26E-72	3.24E-69
PCBP3	163.74658	-3.045917	0.1697908	-17.93923	5.83E-72	1.42E-68
HMGA2	54.092768	-3.648735	0.2053008	-17.77263	1.15E-70	2.66E-67
PPP2R2C	199.07211	-3.222339	0.1815332	-17.75069	1.70E-70	3.73E-67
SPOCD1	74.394035	-1.998145	0.1139336	-17.5378	7.37E-69	1.54E-65
CPNE7	224.40507	-2.59883	0.1483838	-17.51424	1.12E-68	2.22E-65
HP	2326.2087	-4.540661	0.2595532	-17.49415	1.59E-68	3.02E-65
IGFN1	239.6806	-4.209831	0.2413055	-17.44606	3.69E-68	6.73E-65
GFPT2	864.91481	-2.355402	0.1361791	-17.29635	5.01E-67	8.44E-64
SAA1	4194.3517	-4.572083	0.2645898	-17.27989	6.67E-67	1.08E-63
TCHH	93.138119	-2.435012	0.1422031	-17.12348	9.92E-66	1.55E-62
PLTP	7814.8844	-2.010957	0.1187648	-16.93226	2.60E-64	3.68E-61
FRMD5	29.230218	-2.734075	0.1628636	-16.78752	3.01E-63	4.12E-60
MMP12	61.310065	-3.674617	0.2194651	-16.74351	6.32E-63	8.38E-60
TMEM158	115.70335	-2.036291	0.1219906	-16.69219	1.49E-62	1.92E-59
NPEPL1	1441.6836	-1.401271	0.0842327	-16.63571	3.84E-62	4.67E-59
LINC00942	44.107017	-3.23147	0.194676	-16.59922	7.06E-62	8.36E-59
ARSI	81.912212	-2.857429	0.173184	-16.49938	3.71E-61	4.27E-58
IGFBP1	2010.8143	-3.949107	0.2400779	-16.44927	8.49E-61	9.53E-58
WFDC3	57.809005	-1.924936	0.1178734	-16.33054	5.99E-60	6.24E-57
TMEM92-AS1	21.680409	-2.21084	0.135607	-16.30329	9.35E-60	9.53E-57
BCAS1	120.24264	-2.928967	0.1797104	-16.29826	1.02E-59	1.01E-56
FAM78B	244.90006	-1.353428	0.0831143	-16.28393	1.28E-59	1.25E-56
WFDC10B	8.8092087	-3.068523	0.1886199	-16.26829	1.66E-59	1.58E-56
PADI3	93.195719	-3.877164	0.2387209	-16.24141	2.57E-59	2.39E-56
NUMBL	724.20152	-1.156573	0.0716467	-16.14273	1.28E-58	1.17E-55
ITPKA	193.33315	-2.566463	0.1592583	-16.1151	2.00E-58	1.79E-55
MMP9	1769.3197	-2.472562	0.1545572	-15.99772	1.33E-57	1.16E-54
SAA2	781.23827	-4.163212	0.2634261	-15.8041	2.92E-56	2.50E-53
FZD2	208.70637	-1.404883	0.0893507	-15.72325	1.05E-55	8.50E-53
SERPINF1	3552.3837	-1.796027	0.1142386	-15.72171	1.07E-55	8.55E-53
LOXL1	829.11441	-1.687448	0.1075667	-15.68745	1.84E-55	1.44E-52
GRIN2D	112.98241	-1.42658	0.0911242	-15.65534	3.05E-55	2.35E-52

GCKR	22.499132	-2.967179	0.1896914	-15.64214	3.76E-55	2.84E-52
STEAP3-AS1	23.432213	-2.012361	0.1290451	-15.59425	7.97E-55	5.91E-52
RARRES1	875.86071	-2.165199	0.1396121	-15.50868	3.03E-54	2.21E-51
UBE2C	465.43158	-1.766905	0.1139863	-15.50103	3.41E-54	2.45E-51
CIDEC	79.584792	-3.557602	0.2308289	-15.41229	1.35E-53	9.41E-51
NUDT11	43.186147	-2.042994	0.1330902	-15.35045	3.52E-53	2.41E-50
SRPX2	500.68229	-2.253314	0.1472417	-15.3035	7.25E-53	4.81E-50
LINC00607	30.817278	-1.402783	0.0917011	-15.29734	7.96E-53	5.13E-50
PDGFRL	264.86812	-1.978782	0.1307492	-15.13418	9.64E-52	5.86E-49
CYP1B1-AS1	78.500816	-1.692645	0.1127278	-15.01533	5.83E-51	3.40E-48
CDHR4	13.135345	-2.615926	0.1745636	-14.98552	9.13E-51	5.26E-48
CPA4	262.86817	-3.073857	0.2054546	-14.96125	1.32E-50	7.39E-48
ZIC2	24.299734	-3.744632	0.2527973	-14.81279	1.21E-49	6.63E-47
KCNK17	58.935682	-2.702283	0.182552	-14.80281	1.40E-49	7.60E-47
CILP2	56.247253	-2.401149	0.1624073	-14.78473	1.84E-49	9.70E-47
ZP1	50.072001	-2.714598	0.1842201	-14.73563	3.81E-49	1.96E-46
BASP1	1312.7567	-1.511261	0.1025882	-14.73133	4.06E-49	2.07E-46
EGFL6	50.633535	-2.610599	0.1775006	-14.70755	5.77E-49	2.90E-46
CDCA3	244.41735	-1.258828	0.0856494	-14.69746	6.69E-49	3.33E-46
MYBL2	428.08656	-1.569395	0.1070054	-14.66651	1.06E-48	5.20E-46
FLNC	1487.4173	-2.199051	0.1501673	-14.64401	1.47E-48	7.16E-46
OTX1	13.554716	-2.929918	0.2002108	-14.63417	1.70E-48	8.19E-46
TROAP	197.22707	-1.439301	0.0984232	-14.62359	1.99E-48	9.46E-46
LINC01554	655.27389	-3.160394	0.2161681	-14.62008	2.09E-48	9.85E-46
CENPW	129.77758	-1.012369	0.0693254	-14.60315	2.68E-48	1.25E-45
CDH15	33.87477	-2.459683	0.1692747	-14.53071	7.74E-48	3.46E-45
STEAP3	2804.9074	-1.898562	0.1307412	-14.52153	8.85E-48	3.92E-45
MRC2	3584.8433	-1.321971	0.0912125	-14.49331	1.34E-47	5.85E-45
ITIH3	252.56269	-1.872133	0.1292063	-14.48949	1.41E-47	6.12E-45
TRPM8	47.662113	-2.196592	0.1517017	-14.47968	1.63E-47	6.99E-45
CARD14	202.35109	-1.90381	0.1315004	-14.47759	1.68E-47	7.14E-45
ONECUT2	76.509343	-2.640944	0.1824323	-14.47629	1.71E-47	7.21E-45
LEF1	945.34537	-1.703222	0.1177053	-14.47021	1.87E-47	7.80E-45
SPRED3	148.81432	-1.180737	0.0817083	-14.45064	2.48E-47	1.02E-44
ITIH4	56.865881	-2.063653	0.1430032	-14.43082	3.31E-47	1.33E-44
ZMIZ1-AS1	75.440257	-1.466528	0.1018512	-14.39873	5.27E-47	2.08E-44
GJB6	13.631746	-3.104758	0.216361	-14.3499	1.07E-46	4.17E-44
PTGES	529.01503	-2.030068	0.1414774	-14.34906	1.08E-46	4.19E-44
FKBP11	1545.9293	-1.08226	0.0756565	-14.30491	2.04E-46	7.77E-44
FBF1	195.68637	-1.130759	0.0790933	-14.29651	2.30E-46	8.69E-44
FRMD3	2906.1942	1.0978088	0.0768052	14.293424	2.40E-46	9.00E-44
PODNL1	186.05565	-2.163918	0.1517575	-14.25905	3.94E-46	1.45E-43
PLXNB3	206.13693	-1.768344	0.1246505	-14.18642	1.11E-45	4.06E-43
SEMA7A	459.08526	-1.173734	0.0827587	-14.18262	1.17E-45	4.25E-43
WFDC21P	83.35902	-1.990945	0.1405816	-14.1622	1.57E-45	5.59E-43
ABCG2	1215.0129	1.2908647	0.0913326	14.133663	2.36E-45	8.32E-43
FAM225A	31.841201	-1.382175	0.0979505	-14.11095	3.25E-45	1.13E-42
FIRRE	28.253077	-1.892913	0.1341475	-14.11068	3.26E-45	1.13E-42
COL1A1	65661.706	-1.822665	0.1292254	-14.10454	3.56E-45	1.22E-42
MLLT11	392.40692	-1.20721	0.0857413	-14.07968	5.06E-45	1.72E-42
GOLGA6L2	21.071528	-3.964577	0.2816082	-14.07834	5.16E-45	1.73E-42

F3	666.71331	-1.975293	0.1403567	-14.07338	5.54E-45	1.84E-42
COL5A1	7873.6895	-1.616605	0.1148844	-14.07158	5.68E-45	1.87E-42
LHX2	12.590747	-2.319887	0.1649959	-14.06028	6.66E-45	2.16E-42
AURKB	194.32803	-1.373792	0.097943	-14.02644	1.07E-44	3.42E-42
SBSN	9.7965065	-3.400801	0.2428454	-14.00397	1.47E-44	4.65E-42
FEZF1-AS1	6.2643865	-3.30053	0.2366249	-13.94837	3.22E-44	1.00E-41
KCNH3	45.123506	-1.831251	0.1313984	-13.93663	3.79E-44	1.17E-41
IGLC7	141.33634	-3.093577	0.2221733	-13.92417	4.52E-44	1.38E-41
FKBP9P1	403.474	-2.208441	0.1588494	-13.90274	6.10E-44	1.84E-41
RSPO4	51.310639	-2.366851	0.170521	-13.88011	8.36E-44	2.51E-41
RPL29P19	15.996893	-1.80069	0.1298747	-13.86483	1.03E-43	3.08E-41
CRP	108.64229	-3.344774	0.2415933	-13.84465	1.37E-43	4.00E-41
ZNF365	179.31726	-2.067068	0.1496438	-13.81325	2.12E-43	6.11E-41
HTR3A	14.554208	-2.857312	0.2077268	-13.75515	4.74E-43	1.35E-40
BRSK1	144.06318	-1.00414	0.0730082	-13.7538	4.83E-43	1.37E-40
OR13A1	8.3688967	-2.267314	0.1651251	-13.73089	6.63E-43	1.84E-40
CPXM1	445.68335	-2.094043	0.1525631	-13.72575	7.12E-43	1.96E-40
BARX1	12.772548	-3.13992	0.2290138	-13.71061	8.77E-43	2.37E-40
IGFL2	26.177915	-2.589874	0.1892961	-13.6816	1.31E-42	3.49E-40
CHRDL2	189.56293	-2.463498	0.1800892	-13.67932	1.35E-42	3.58E-40
IGLV3-9	183.13441	-2.743047	0.2006367	-13.67172	1.50E-42	3.94E-40
MIAT	479.55761	-1.925057	0.1408079	-13.67151	1.50E-42	3.94E-40
APBA2	225.72176	-1.557745	0.1139936	-13.6652	1.64E-42	4.27E-40
LINC00973	8.0483762	-3.103902	0.2272253	-13.66002	1.76E-42	4.56E-40
CLMP	414.20435	-2.442475	0.1790717	-13.63965	2.33E-42	5.96E-40
PLAU	2725.1895	-1.225145	0.0899161	-13.62543	2.83E-42	7.12E-40
LRP8	241.52605	-1.125154	0.0826893	-13.60701	3.64E-42	9.06E-40
MFSD2A	404.77695	-2.00185	0.1471337	-13.60565	3.71E-42	9.17E-40
TIMP1	27385.017	-1.227946	0.0902723	-13.6027	3.86E-42	9.44E-40
ADAM8	721.84674	-1.302464	0.0957569	-13.60178	3.91E-42	9.51E-40
PTPRN	423.77271	-2.25287	0.1656582	-13.5995	4.03E-42	9.76E-40
TICRR	118.37814	-1.177287	0.0866641	-13.58448	4.95E-42	1.19E-39
SPEG	226.21292	-1.560603	0.1148821	-13.58439	4.96E-42	1.19E-39
DMRT3	10.821496	-3.684012	0.2716514	-13.56154	6.77E-42	1.60E-39
RTL1	10.27554	-4.913702	0.3627699	-13.54496	8.49E-42	2.00E-39
GNB3	44.918854	-1.855808	0.1374556	-13.50114	1.54E-41	3.59E-39
C11orf86	131.7652	-2.92457	0.2166697	-13.49782	1.61E-41	3.73E-39
MMP13	27.885244	-3.722665	0.2765382	-13.46167	2.63E-41	6.03E-39
TMPRSS6	54.56647	-1.917334	0.1424417	-13.46048	2.67E-41	6.10E-39
IGHG3	11520.871	-2.599293	0.1931458	-13.45767	2.78E-41	6.30E-39
SLAMF9	7.7137111	-2.815995	0.2093318	-13.45231	2.98E-41	6.74E-39
TLL2	43.2937	-1.618255	0.1203477	-13.44649	3.23E-41	7.21E-39
ARTN	71.749633	-1.806864	0.1347	-13.41399	5.01E-41	1.11E-38
P4HA3	503.30457	-1.623008	0.1210265	-13.41035	5.26E-41	1.16E-38
P3H3	919.68339	-1.673584	0.1250025	-13.38841	7.07E-41	1.54E-38
TM4SF19	28.751591	-2.017249	0.150883	-13.36963	9.10E-41	1.95E-38
KREMEN2	26.566128	-1.793792	0.1341692	-13.36962	9.10E-41	1.95E-38
WFDC5	36.21765	-4.474049	0.3349784	-13.35623	1.09E-40	2.33E-38
LINC00941	36.237323	-2.026796	0.1518293	-13.34918	1.20E-40	2.54E-38
JAK3	1958.7297	-1.080448	0.0809384	-13.34901	1.20E-40	2.54E-38
CTHRC1	1762.2598	-1.791932	0.134265	-13.34624	1.25E-40	2.62E-38

INHBE	229.63089	-2.229283	0.1670556	-13.34455	1.27E-40	2.67E-38
ZBTB7C	502.20997	-2.044823	0.1533785	-13.33188	1.51E-40	3.15E-38
PDPN	227.82277	-1.905258	0.1429988	-13.3236	1.69E-40	3.47E-38
KIF18B	187.34252	-1.317846	0.098911	-13.32355	1.69E-40	3.47E-38
FAM20A	1136.9285	-1.432729	0.1076167	-13.31327	1.94E-40	3.97E-38
IL6	541.72465	-2.502992	0.1881914	-13.30025	2.31E-40	4.70E-38
RUNX1	3969.7554	-1.020696	0.0769831	-13.2587	4.02E-40	8.04E-38
RAB40A	56.346673	-1.082396	0.0816869	-13.25056	4.48E-40	8.92E-38
PLK1	345.78145	-1.194604	0.0905543	-13.19213	9.74E-40	1.90E-37
CFHR3	20.817787	-1.938312	0.1470021	-13.1856	1.06E-39	2.06E-37
PPP1R1A	1860.278	-2.85997	0.2171514	-13.17039	1.30E-39	2.51E-37
IGKV3-15	903.78582	-2.568334	0.1951178	-13.16299	1.43E-39	2.74E-37
DERL3	674.64264	-1.570544	0.1195188	-13.14056	1.93E-39	3.67E-37
KIRREL3	34.505737	-1.784351	0.1361717	-13.10368	3.14E-39	5.90E-37
COLGALT2	164.14602	-1.812355	0.138359	-13.09893	3.34E-39	6.25E-37
TF	745.94548	-2.735832	0.2090539	-13.08673	3.92E-39	7.31E-37
TMC3	9.8345147	-2.094998	0.1600913	-13.08626	3.95E-39	7.32E-37
IGHGP	1433.7365	-2.468575	0.1886608	-13.08473	4.03E-39	7.44E-37
MZB1	625.1588	-2.327637	0.1780545	-13.07262	4.72E-39	8.69E-37
LINC01436	92.956558	-2.843463	0.217577	-13.06877	4.97E-39	9.10E-37
EDNRB	10711.325	1.2175764	0.0932812	13.052758	6.13E-39	1.11E-36
SPON2	5378.7848	-1.513533	0.1159685	-13.05124	6.25E-39	1.13E-36
SAA2-SAA4	133.88241	-4.440584	0.3403868	-13.0457	6.72E-39	1.21E-36
SMCO3	71.612287	-1.809322	0.1388891	-13.0271	8.58E-39	1.53E-36
ZNF114	190.68095	-2.267275	0.1745946	-12.98594	1.47E-38	2.60E-36
ACTN2	145.5494	-2.026039	0.1561265	-12.97691	1.65E-38	2.89E-36
IL20RB	1613.003	-2.557201	0.1971938	-12.96796	1.86E-38	3.22E-36
PRSS3	20.704903	-2.582318	0.1993611	-12.95296	2.26E-38	3.88E-36
PTTG1	450.71265	-1.170836	0.0904476	-12.94492	2.51E-38	4.26E-36
KCND1	184.24157	-1.037279	0.0803276	-12.91311	3.80E-38	6.35E-36
SLC18A3	128.10249	-4.557759	0.3531748	-12.90511	4.21E-38	7.02E-36
HCAR2	60.470386	-2.041954	0.1584303	-12.88866	5.21E-38	8.65E-36
OXTR	180.09258	-1.460091	0.1133388	-12.88254	5.64E-38	9.30E-36
CCNB2	307.42222	-1.040357	0.0808027	-12.87528	6.20E-38	1.02E-35
GPR173	216.20543	-1.48693	0.1155288	-12.87064	6.59E-38	1.07E-35
CCL26	21.375771	-1.69697	0.131891	-12.86646	6.95E-38	1.12E-35
SLC17A9	670.06529	-1.557231	0.1210545	-12.86388	7.19E-38	1.15E-35
HJURP	231.71327	-1.258873	0.0980421	-12.84013	9.77E-38	1.56E-35
MMP19	593.3884	-1.415172	0.1102346	-12.83782	1.01E-37	1.60E-35
PYCR1	589.35155	-1.514235	0.117992	-12.83337	1.07E-37	1.69E-35
SHOX2	31.331251	-1.898012	0.1479795	-12.82618	1.17E-37	1.85E-35
C6orf141	82.95849	-2.133263	0.1663736	-12.82212	1.23E-37	1.94E-35
TCN1	27.035549	-2.782962	0.2173183	-12.80593	1.52E-37	2.38E-35
TRIM46	130.61822	-1.424809	0.1114615	-12.78297	2.04E-37	3.16E-35
CRABP1	33.643597	-4.156158	0.3253551	-12.77422	2.28E-37	3.52E-35
F2	314.19952	-3.021948	0.2369229	-12.75499	2.92E-37	4.48E-35
CDC20	376.50695	-1.233546	0.0967238	-12.75328	2.99E-37	4.56E-35
TBX5	63.214231	-3.56939	0.2799249	-12.75124	3.07E-37	4.67E-35
WNT10B	25.044581	-1.654072	0.1298178	-12.74149	3.48E-37	5.25E-35
PLXNA4	140.42331	-1.640986	0.128933	-12.72743	4.16E-37	6.25E-35
FOXM1	669.32461	-1.204518	0.0946898	-12.72067	4.54E-37	6.76E-35

PCOLCE	2187.1803	-1.235709	0.0973634	-12.69173	6.57E-37	9.66E-35
ACTBP12	6.2328723	-3.157515	0.2491992	-12.67065	8.60E-37	1.26E-34
RGS17	107.95663	-1.435738	0.1133218	-12.66956	8.72E-37	1.27E-34
C5orf38	57.726578	-2.210159	0.1746001	-12.65841	1.01E-36	1.46E-34
TMEM145	114.48717	-2.07606	0.164165	-12.64618	1.17E-36	1.69E-34
EPHB2	473.05933	-1.322262	0.104916	-12.60305	2.03E-36	2.92E-34
PLAUR	1438.1034	-1.147943	0.0911044	-12.6003	2.10E-36	3.01E-34
WNT5A	462.07355	-1.239515	0.0984586	-12.5892	2.42E-36	3.44E-34
IVL	4.9276191	-3.601631	0.2862596	-12.58169	2.66E-36	3.77E-34
COL22A1	365.00232	-1.958959	0.1557631	-12.57653	2.84E-36	4.02E-34
KCNN4	179.82122	-1.261837	0.1003725	-12.57155	3.03E-36	4.26E-34
IGHG4	3142.1608	-2.51585	0.2003605	-12.55662	3.66E-36	5.13E-34
PIK3R6	296.09241	-1.205737	0.0961412	-12.54131	4.44E-36	6.17E-34
MIXL1	13.800541	-1.931943	0.1541386	-12.53381	4.88E-36	6.74E-34
MAP7	3231.6097	1.1036853	0.0880638	12.532791	4.94E-36	6.80E-34
TRPV3	56.962133	-1.532081	0.1222896	-12.5283	5.23E-36	7.16E-34
GNAS-AS1	14.547289	-1.532134	0.1222942	-12.52826	5.23E-36	7.16E-34
DMBX1	4.9686494	-2.832296	0.2261058	-12.52642	5.35E-36	7.30E-34
GAS2L2	27.968064	-2.889245	0.2307332	-12.52201	5.66E-36	7.70E-34
RCN3	1532.943	-1.140999	0.0911555	-12.51707	6.02E-36	8.17E-34
C1R	19776.93	-1.407242	0.1124703	-12.51212	6.41E-36	8.64E-34
UNC5A	154.73229	-1.74756	0.1396923	-12.51007	6.58E-36	8.84E-34
DLX4	63.457203	-1.475986	0.1180139	-12.50688	6.85E-36	9.17E-34
FCRL5	187.08055	-2.255431	0.1807148	-12.48061	9.53E-36	1.26E-33
DLGAP1-AS2	123.57428	-1.06988	0.0857355	-12.47884	9.74E-36	1.28E-33
COL16A1	1233.6187	-1.424678	0.1142253	-12.47253	1.05E-35	1.38E-33
SLC52A1	50.994657	-1.971668	0.1585087	-12.43886	1.61E-35	2.09E-33
TNFSF14	442.26132	-1.623378	0.1305343	-12.43641	1.66E-35	2.14E-33
ALDH6A1	3646.7679	1.035786	0.0833192	12.43154	1.76E-35	2.27E-33
PLA2G4D	33.830246	-2.396616	0.1929264	-12.42243	1.97E-35	2.54E-33
UCN2	6.9655942	-2.580701	0.2078377	-12.41691	2.12E-35	2.71E-33
ANKRD18B	10.032895	-2.768122	0.2229937	-12.41345	2.21E-35	2.82E-33
CYP19A1	18.337685	-1.767941	0.1424571	-12.41034	2.30E-35	2.92E-33
DUXAP8	163.12125	-1.417199	0.1142061	-12.40914	2.33E-35	2.96E-33
IGDCC4	410.94836	-2.073576	0.1671351	-12.40659	2.41E-35	3.04E-33
IL1R2	918.23255	-2.041007	0.1650551	-12.36561	4.01E-35	5.02E-33
CENPA	81.745793	-1.242009	0.1005229	-12.35548	4.55E-35	5.68E-33
POU2AF1	332.9142	-2.025694	0.1640932	-12.34478	5.20E-35	6.47E-33
AGTR1	837.75314	1.6412523	0.1330024	12.340018	5.51E-35	6.84E-33
SPHK1	493.77713	-1.254758	0.1017184	-12.33561	5.82E-35	7.21E-33
ASGR1	96.344075	-1.04794	0.0850134	-12.32676	6.50E-35	8.00E-33
KIF2C	307.67798	-1.017721	0.0825945	-12.3219	6.90E-35	8.47E-33
IGKV3-11	3543.0507	-2.355189	0.191146	-12.32142	6.95E-35	8.50E-33
SERTM1	6.5055381	-2.642756	0.2145411	-12.31818	7.23E-35	8.82E-33
LINC00619	12.631629	-2.150335	0.1745744	-12.31759	7.28E-35	8.84E-33
ADA	805.96338	-1.064694	0.0865307	-12.30423	8.60E-35	1.04E-32
IFITM10	766.0296	-1.286097	0.1045288	-12.30375	8.65E-35	1.04E-32
RPLP0P2	26.932959	-1.514848	0.1232015	-12.2957	9.55E-35	1.15E-32
CA4	552.37881	1.8012326	0.1465633	12.28979	1.03E-34	1.23E-32
TPT1P5	28.775213	-1.539902	0.1254585	-12.27419	1.25E-34	1.49E-32
MOCOS	501.48248	-1.756188	0.1433832	-12.24821	1.72E-34	2.04E-32

IGKV1-17	429.71307	-2.433251	0.1987054	-12.24552	1.78E-34	2.11E-32
MUC12	174.69147	-1.813782	0.1482352	-12.23584	2.00E-34	2.37E-32
KLK10	23.5906	-2.463175	0.2014706	-12.22598	2.26E-34	2.67E-32
HAS2-AS1	8.5189512	-2.089133	0.170909	-12.22366	2.32E-34	2.74E-32
C10orf90	8.2884679	-2.378355	0.1947164	-12.21446	2.60E-34	3.06E-32
MXD3	299.11285	-1.075093	0.0880298	-12.21284	2.65E-34	3.11E-32
SNORD99	26.473466	-1.68561	0.1380418	-12.21086	2.72E-34	3.18E-32
C2CD4A	100.20643	-2.461166	0.2015628	-12.21042	2.73E-34	3.19E-32
KRT20	9.4430412	-2.920311	0.2392514	-12.20603	2.89E-34	3.35E-32
TREM1	274.30041	-1.70243	0.1394818	-12.20539	2.91E-34	3.37E-32
KIF20A	453.17949	-1.197759	0.098304	-12.18424	3.77E-34	4.36E-32
LINC00460	66.930321	-2.832831	0.2326055	-12.1787	4.04E-34	4.65E-32
IL2RA	295.32816	-1.491175	0.1224841	-12.17444	4.25E-34	4.88E-32
GTSE1	220.07771	-1.03007	0.0846758	-12.16487	4.78E-34	5.47E-32
PDGFD	5729.9916	1.0234071	0.0841669	12.159252	5.12E-34	5.83E-32
PKP3	288.74439	-2.574586	0.2118732	-12.15154	5.63E-34	6.39E-32
OACYLP	77.393136	-1.569133	0.1292489	-12.1404	6.45E-34	7.26E-32
POPDC3	6.9316962	-2.159821	0.1782288	-12.11825	8.45E-34	9.44E-32
GUCY2D	34.067819	-1.756637	0.1449592	-12.11815	8.46E-34	9.44E-32
CCNO	63.416985	-2.20178	0.1818235	-12.10944	9.41E-34	1.04E-31
SYN1	144.88928	-1.355567	0.1120292	-12.10012	1.05E-33	1.16E-31
ECM1	1563.6852	-1.067879	0.0882557	-12.09984	1.06E-33	1.16E-31
HADHAP1	12.940359	-1.765152	0.1461789	-12.07529	1.43E-33	1.56E-31
EMILIN1	2423.5909	-1.358633	0.1125805	-12.0681	1.56E-33	1.70E-31
CCNA1	40.02885	-2.305354	0.1910969	-12.0638	1.64E-33	1.78E-31
LINC00525	9.1873535	-1.670803	0.1385324	-12.06074	1.70E-33	1.84E-31
IGHV3-43	85.866527	-2.417128	0.2005233	-12.0541	1.85E-33	1.99E-31
PLA2G2A	65.007252	-3.010915	0.250009	-12.04323	2.11E-33	2.26E-31
GAD1	259.75506	-2.164048	0.1798421	-12.03304	2.38E-33	2.55E-31
C10orf55	27.48403	-1.071663	0.0890761	-12.03087	2.45E-33	2.61E-31
CACNA1B	10.038634	-2.161134	0.1796625	-12.02885	2.51E-33	2.66E-31
IGHV4-31	184.99765	-2.237669	0.1860246	-12.02889	2.51E-33	2.66E-31
CORO6	104.64597	-1.672281	0.1390813	-12.02376	2.67E-33	2.82E-31
MFAP2	217.26419	-1.71232	0.142423	-12.02277	2.70E-33	2.85E-31
IGHV1-69	223.40424	-2.653264	0.2208002	-12.01658	2.91E-33	3.05E-31
IGLV6-57	677.97137	-2.315619	0.1927005	-12.01667	2.90E-33	3.05E-31
MXRA8	4569.437	-1.381478	0.1149693	-12.01606	2.93E-33	3.07E-31
SPC24	238.58307	-1.078013	0.089785	-12.0066	3.28E-33	3.42E-31
CD44	11422.022	-1.132207	0.0943326	-12.00229	3.46E-33	3.60E-31
SAA4	21.577375	-3.282028	0.273684	-11.99204	3.91E-33	4.05E-31
COL6A3	13830.359	-1.448741	0.1209978	-11.97329	4.90E-33	5.04E-31
IGFLR1	146.13917	-1.008698	0.0842908	-11.96689	5.30E-33	5.42E-31
AQP6	249.45121	3.2101839	0.2684224	11.95945	5.79E-33	5.92E-31
RGS20	48.516855	-1.765775	0.1477	-11.95515	6.10E-33	6.20E-31
IGHG1	101795.13	-2.489748	0.2082803	-11.95383	6.20E-33	6.27E-31
LINC00922	5.2968733	-3.441373	0.2880772	-11.94601	6.81E-33	6.88E-31
IGHV1-24	414.24225	-2.549025	0.213663	-11.93012	8.25E-33	8.25E-31
ATP2B3	9.8256548	-2.571972	0.215661	-11.926	8.66E-33	8.65E-31
BTBD11	379.72809	-1.765317	0.1481596	-11.91496	9.89E-33	9.80E-31
MMP17	95.133396	-1.747792	0.1467252	-11.91201	1.02E-32	1.01E-30
CDH17	52.106471	-1.857517	0.1560133	-11.90615	1.10E-32	1.08E-30

FAM225B	21.763045	-1.160952	0.0975501	-11.90109	1.17E-32	1.15E-30
VAC14-AS1	27.075832	-1.445071	0.1215225	-11.89139	1.31E-32	1.29E-30
FRMPD3	27.684094	-1.4404	0.1212242	-11.88212	1.47E-32	1.43E-30
STRA6	69.725897	-1.865619	0.1570847	-11.87652	1.57E-32	1.53E-30
RIN1	612.10191	-1.009865	0.0850717	-11.87075	1.68E-32	1.63E-30
ISL2	6.5819939	-1.941548	0.1636302	-11.86546	1.79E-32	1.73E-30
SLN	71.76064	-3.044052	0.2565551	-11.8651	1.80E-32	1.73E-30
LINC01127	1094.4084	-2.350375	0.1982617	-11.85491	2.03E-32	1.95E-30
MAST1	34.52334	-1.576715	0.1330653	-11.84919	2.17E-32	2.08E-30
BIRC5	488.37164	-1.141243	0.0963294	-11.8473	2.22E-32	2.12E-30
COX6B2	10.516381	-1.771359	0.1495783	-11.84235	2.36E-32	2.25E-30
DRP2	12.093241	-1.507527	0.12733	-11.83952	2.44E-32	2.32E-30
GCK	20.310155	-1.634506	0.1381321	-11.83292	2.64E-32	2.50E-30
MACROD2	626.57085	-1.648848	0.1394389	-11.82488	2.90E-32	2.74E-30
C1S	23719.453	-1.3879	0.1174158	-11.82038	3.06E-32	2.89E-30
CD177	53.63123	-2.099662	0.1777261	-11.81404	3.30E-32	3.11E-30
DUXAP10	25.180355	-1.319323	0.1117693	-11.80399	3.72E-32	3.48E-30
LYPD1	103.93428	-1.627424	0.1378945	-11.80196	3.81E-32	3.55E-30
GPR84	76.389827	-1.263836	0.1070915	-11.80146	3.84E-32	3.57E-30
S100G	10.688449	-4.335102	0.3680195	-11.77954	4.98E-32	4.60E-30
CD19	47.279036	-1.679021	0.1426312	-11.77176	5.46E-32	5.03E-30
ZIC5	11.995261	-3.361477	0.285988	-11.75391	6.74E-32	6.15E-30
PIM2	1836.252	-1.075982	0.0915453	-11.75355	6.77E-32	6.17E-30
FAP	403.34697	-1.296368	0.1104244	-11.73987	7.96E-32	7.22E-30
P2RX5	46.822757	-1.388515	0.1183228	-11.73498	8.43E-32	7.63E-30
CHI3L2	416.25417	-1.662293	0.1416929	-11.73166	8.77E-32	7.92E-30
MAFA-AS1	6.7036341	-2.865419	0.2442889	-11.72963	8.98E-32	8.10E-30
IGLV3-10	395.54522	-2.360134	0.2012453	-11.72765	9.20E-32	8.27E-30
SV2B	132.25312	-1.747208	0.1490175	-11.72485	9.51E-32	8.53E-30
EN2	31.824297	-2.422855	0.2068027	-11.71578	1.06E-31	9.44E-30
ST8SIA2	12.604	-2.661872	0.2272421	-11.71381	1.08E-31	9.64E-30
EMCN	4296.2733	1.0836012	0.0926112	11.700538	1.27E-31	1.12E-29
HTRA4	93.072776	-1.528459	0.1306366	-11.70008	1.27E-31	1.12E-29
SCGN	2493.1005	2.0464486	0.1750376	11.69148	1.41E-31	1.24E-29
CERCAM	3081.4207	-1.030354	0.0881963	-11.68251	1.57E-31	1.36E-29
PANX2	129.45726	-2.168508	0.1856469	-11.68082	1.60E-31	1.39E-29
LINC00313	3.9799262	-2.076014	0.1777814	-11.67734	1.66E-31	1.44E-29
WFDC13	3.347487	-2.123704	0.1819695	-11.67066	1.80E-31	1.55E-29
KCNS1	216.66297	-3.002506	0.2573731	-11.66597	1.90E-31	1.63E-29
IGLV1-44	1365.0766	-2.328892	0.1998465	-11.6534	2.20E-31	1.89E-29
PRRX1	939.92132	-1.440724	0.1236811	-11.6487	2.33E-31	1.99E-29
MEI1	169.95087	-1.224121	0.1051127	-11.6458	2.41E-31	2.05E-29
PLA2G2D	196.6388	-2.071246	0.1778628	-11.64519	2.43E-31	2.06E-29
B3GALT5	391.34357	-2.185743	0.1877047	-11.64459	2.45E-31	2.07E-29
TENM3	441.28561	-1.860984	0.1601188	-11.62252	3.17E-31	2.66E-29
THBS2	8488.5979	-1.545751	0.1330697	-11.61611	3.41E-31	2.86E-29
TNNI3	12.129782	-3.070267	0.2643404	-11.61483	3.46E-31	2.90E-29
C10orf99	1177.5808	-2.891833	0.2489842	-11.61452	3.48E-31	2.90E-29
CEMIP	211.9176	-1.306188	0.1125723	-11.6031	3.97E-31	3.30E-29
IGLL5	2235.1315	-2.250511	0.1940295	-11.59881	4.18E-31	3.46E-29
KCNMB2-AS1	10.864165	-1.80865	0.1559411	-11.59829	4.20E-31	3.47E-29

CTSK	1458.5407	-1.359164	0.1172649	-11.59054	4.60E-31	3.80E-29
GRIK4	128.97188	-1.767794	0.1526689	-11.57927	5.25E-31	4.32E-29
PIF1	85.101407	-1.118978	0.0966629	-11.57608	5.45E-31	4.48E-29
APOLD1	15468.15	1.1480241	0.0992392	11.568255	5.97E-31	4.90E-29
IGHV5-51	1151.869	-2.230832	0.1929612	-11.56104	6.49E-31	5.32E-29
IQGAP3	569.7644	-1.050786	0.0909567	-11.5526	7.16E-31	5.85E-29
IGLV2-11	939.20839	-2.132104	0.1848087	-11.53682	8.61E-31	7.00E-29
NUF2	144.79244	-1.052464	0.0912643	-11.53205	9.10E-31	7.35E-29
TGFBI	101582.73	-1.889347	0.1640127	-11.51951	1.05E-30	8.46E-29
ST6GALNAC5	24.517753	-2.067338	0.1798038	-11.49774	1.35E-30	1.08E-28
SCN4A	431.22878	1.3786387	0.1199707	11.491462	1.46E-30	1.15E-28
IGLV7-46	226.52989	-2.24793	0.1957825	-11.48177	1.63E-30	1.28E-28
IGKC	43779.732	-2.16816	0.1888751	-11.47933	1.68E-30	1.32E-28
SEMA3E	28.159858	-2.58288	0.2253668	-11.46078	2.08E-30	1.62E-28
TPX2	917.41329	-1.053945	0.0920877	-11.44502	2.49E-30	1.93E-28
FUT8-AS1	15.903987	-1.123804	0.0981951	-11.44461	2.50E-30	1.94E-28
SLPI	1489.6854	-2.63363	0.2303822	-11.43157	2.91E-30	2.24E-28
MUC17	31.256661	-3.043021	0.266307	-11.42674	3.07E-30	2.35E-28
DNER	307.92383	-2.356122	0.2062115	-11.42576	3.11E-30	2.38E-28
TMC5	58.554118	-1.820586	0.1594015	-11.42139	3.27E-30	2.49E-28
IZUMO4	103.93861	-1.183175	0.103706	-11.40894	3.77E-30	2.86E-28
ROR1-AS1	8.9765979	-1.740052	0.1525666	-11.40519	3.94E-30	2.98E-28
CPEB1	107.96059	-2.02352	0.1774322	-11.40447	3.97E-30	2.99E-28
PDIA2	11.167762	-1.948046	0.170882	-11.39995	4.18E-30	3.15E-28
LLOXNC01-2	12.515567	-1.067246	0.0936217	-11.39956	4.20E-30	3.16E-28
NMU	58.632412	-2.151524	0.1887864	-11.3966	4.35E-30	3.26E-28
COL11A1	370.17235	-2.210938	0.1941982	-11.38496	4.97E-30	3.71E-28
CEP55	334.06907	-1.0533	0.0925174	-11.38488	4.97E-30	3.71E-28
NAV3	147.75241	-1.454508	0.1278079	-11.38043	5.23E-30	3.87E-28
HSD11B1	132.95342	-1.582318	0.1390882	-11.37637	5.48E-30	4.03E-28
BEAN1	78.656046	-1.42295	0.1251147	-11.37317	5.69E-30	4.17E-28
IGLC2	5016.3875	-2.063217	0.1814163	-11.37283	5.71E-30	4.18E-28
COL1A2	53859.503	-1.280451	0.1127054	-11.36104	6.54E-30	4.77E-28
JSRP1	88.287497	-1.744488	0.1536455	-11.35398	7.09E-30	5.16E-28
PRRX2	79.78366	-1.411472	0.1243919	-11.34698	7.68E-30	5.58E-28
IL20RB-AS1	4.4741101	-2.89532	0.2552833	-11.34159	8.16E-30	5.91E-28
ARL9	16.465534	-1.569865	0.1385598	-11.32988	9.33E-30	6.72E-28
AIM2	151.13318	-1.415525	0.1249576	-11.32804	9.53E-30	6.86E-28
ARL4C	5734.4948	-1.124055	0.0992455	-11.32601	9.76E-30	7.01E-28
TAS1R3	44.275496	-1.327576	0.117372	-11.31084	1.16E-29	8.29E-28
NPR3	14457.923	1.4357251	0.127011	11.303944	1.25E-29	8.95E-28
ATG9B	337.62053	-1.552772	0.1374397	-11.29784	1.34E-29	9.55E-28
LTK	52.87891	-1.314903	0.116387	-11.29769	1.35E-29	9.55E-28
ANKRD20A19	4.0912113	-2.683784	0.2383352	-11.26054	2.05E-29	1.44E-27
LINC01460	4.9673038	-1.689013	0.1501438	-11.24931	2.33E-29	1.63E-27
IGHG2	12594.591	-2.017882	0.1793818	-11.24909	2.34E-29	1.63E-27
AIRE	10.028434	-1.727815	0.1536299	-11.24661	2.41E-29	1.67E-27
C16orf74	551.0511	-1.582979	0.1407936	-11.24325	2.50E-29	1.74E-27
WNT7B	37.846897	-2.06307	0.1834997	-11.2429	2.51E-29	1.74E-27
COL10A1	122.50044	-2.012741	0.1791155	-11.23711	2.68E-29	1.85E-27
SIX4	41.075253	-1.754538	0.15621	-11.23191	2.84E-29	1.96E-27

FSCN2	32.556386	-1.525074	0.1359222	-11.2202	3.25E-29	2.23E-27
HOTTIP	6.4905376	-2.589043	0.2309467	-11.21057	3.62E-29	2.47E-27
IRX4	2.6227068	-3.275949	0.2922804	-11.20824	3.72E-29	2.53E-27
CD79A	370.92731	-1.809223	0.161607	-11.1952	4.30E-29	2.92E-27
WNT4	54.092201	-1.611432	0.1441945	-11.17541	5.38E-29	3.62E-27
OBP2A	4.660435	-1.890786	0.1692458	-11.17183	5.60E-29	3.75E-27
IGLV1-51	1254.5364	-2.102064	0.1881616	-11.17159	5.62E-29	3.76E-27
IGLV2-23	1672.6405	-2.155806	0.1930345	-11.16798	5.85E-29	3.90E-27
MPP4	10.329641	-1.215434	0.1088676	-11.16434	6.09E-29	4.06E-27
IGKV3-20	3243.7991	-2.129146	0.1907767	-11.16041	6.37E-29	4.23E-27
SLC12A3	127.74047	2.1008325	0.1883003	11.156822	6.63E-29	4.40E-27
RHPN1	1043.7975	-1.026872	0.0920453	-11.15616	6.68E-29	4.42E-27
AFAP1-AS1	111.46425	-2.052802	0.1842304	-11.14258	7.78E-29	5.13E-27
TRABD2A	194.63719	-1.393797	0.1250979	-11.14165	7.87E-29	5.17E-27
KCNJ6	12.427895	-2.041681	0.1834629	-11.12858	9.11E-29	5.96E-27
B3GAT1	161.4658	-1.426555	0.1282023	-11.12737	9.23E-29	6.04E-27
IGLV1-40	1355.5334	-2.066336	0.1857191	-11.12614	9.36E-29	6.10E-27
HOTAIRM1	310.93668	-1.005676	0.0904251	-11.12164	9.84E-29	6.39E-27
TIMP3	1047.762	1.1059691	0.0995076	11.114419	1.07E-28	6.92E-27
IGLV3-19	1146.2084	-2.137259	0.1925671	-11.09877	1.27E-28	8.20E-27
BEND6	115.97099	-1.341179	0.1208846	-11.0947	1.33E-28	8.54E-27
IGSF5	9.0117598	-1.776289	0.1601732	-11.0898	1.41E-28	8.97E-27
SLCO5A1	42.393226	-1.233577	0.1113544	-11.07793	1.61E-28	1.01E-26
SULT2B1	52.050781	-1.579268	0.1426276	-11.07266	1.70E-28	1.07E-26
REEP2	85.841407	-1.57567	0.1423339	-11.07024	1.75E-28	1.10E-26
EDN3	10.40195	-2.696646	0.2437318	-11.06399	1.88E-28	1.17E-26
IL11	11.1677	-1.77581	0.160579	-11.05879	1.99E-28	1.24E-26
G0S2	1809.4208	-1.501469	0.1358324	-11.05384	2.10E-28	1.31E-26
IGHV4-34	548.61551	-2.119767	0.1918218	-11.05071	2.17E-28	1.35E-26
CALY	9.6514602	-1.975372	0.1787674	-11.04995	2.19E-28	1.36E-26
CST2	14.015081	-2.039606	0.1846459	-11.04604	2.29E-28	1.42E-26
ACHE	348.22843	-1.439608	0.1303376	-11.04522	2.31E-28	1.43E-26
RUFY4	69.36285	-1.667652	0.1510101	-11.04332	2.36E-28	1.46E-26
BNC1	51.749746	-2.353098	0.2133086	-11.03142	2.70E-28	1.66E-26
AQP9	861.83725	-1.869734	0.1697634	-11.01376	3.28E-28	2.00E-26
PTX3	113.16138	-1.489133	0.1353067	-11.00561	3.59E-28	2.19E-26
CADM3	1163.583	-2.082622	0.1893104	-11.0011	3.78E-28	2.30E-26
ADAM33	58.946522	-1.890143	0.1719683	-10.99123	4.21E-28	2.56E-26
TRIM54	345.02802	-2.151901	0.1959121	-10.98401	4.56E-28	2.76E-26
ROBO3	402.00422	-1.00992	0.0919834	-10.97938	4.80E-28	2.89E-26
CELSR3	162.34524	-1.138628	0.1038162	-10.96773	5.46E-28	3.27E-26
SLC12A8	496.74163	-1.477991	0.134758	-10.96775	5.46E-28	3.27E-26
SLC12A5	78.16209	-1.011829	0.0922646	-10.96661	5.53E-28	3.31E-26
MDK	3128.2468	-1.367003	0.1246624	-10.96564	5.59E-28	3.34E-26
KRT78	3.9808951	-2.508308	0.2287752	-10.96407	5.69E-28	3.39E-26
PLEKHG4B	122.45875	-1.59428	0.1454511	-10.96094	5.89E-28	3.49E-26
HSH2D	219.97401	-1.159017	0.1058401	-10.95065	6.60E-28	3.90E-26
NFIA-AS2	10.783804	-1.598007	0.1459471	-10.94922	6.70E-28	3.95E-26
IGHA1	26123.407	-1.864996	0.1704599	-10.94097	7.34E-28	4.30E-26
LINC01191	6.0326068	-1.837843	0.1679893	-10.94024	7.40E-28	4.32E-26
CHST6	20.814897	-1.508912	0.1379589	-10.9374	7.64E-28	4.45E-26

SLC22A24	63.431353	1.9039715	0.1741747	10.931392	8.16E-28	4.73E-26
TFF2	20.788851	-2.688558	0.2460484	-10.92695	8.57E-28	4.96E-26
SPINK13	405.84652	-1.95885	0.1793703	-10.92071	9.18E-28	5.30E-26
TNFRSF18	94.243054	-1.147396	0.10508	-10.91926	9.33E-28	5.38E-26
KDELR3	1351.4276	-1.156848	0.1059588	-10.9179	9.47E-28	5.45E-26
KLHL35	39.493907	-1.362451	0.124829	-10.91454	9.82E-28	5.65E-26
CARD11	1109.9024	-1.157863	0.1061596	-10.90681	1.07E-27	6.10E-26
BMPER	101.11121	-1.368198	0.1255175	-10.90045	1.15E-27	6.53E-26
SERPINE1	23377.83	-1.507022	0.1383357	-10.89395	1.23E-27	6.99E-26
TMEM63C	50.564458	-1.447845	0.1330523	-10.88178	1.41E-27	7.93E-26
MAGEC3	6.8949714	-2.481886	0.2281135	-10.88005	1.43E-27	8.07E-26
FST	170.49547	-1.661699	0.1527418	-10.87914	1.45E-27	8.14E-26
LHFPL5	5.7335253	-1.914902	0.1760296	-10.87829	1.46E-27	8.20E-26
USH2A	35.80498	-1.087689	0.1000097	-10.87584	1.50E-27	8.41E-26
HHIPL2	9.6493272	-1.794501	0.1650031	-10.87556	1.51E-27	8.42E-26
SLC28A3	22.970528	-1.465279	0.1347487	-10.87416	1.53E-27	8.54E-26
FNDC1	585.8558	-1.492719	0.1373173	-10.87058	1.59E-27	8.87E-26
UGT1A10	90.277306	-3.108107	0.2859495	-10.86943	1.61E-27	8.97E-26
IGLV3-1	584.62651	-2.132464	0.1962056	-10.86852	1.63E-27	9.05E-26
CREB3L1	561.65481	-1.668578	0.1535803	-10.86453	1.70E-27	9.43E-26
CHGA	9.8664532	-1.912399	0.1762486	-10.85058	1.98E-27	1.09E-25
COL25A1	309.12487	1.4018138	0.1293746	10.835306	2.34E-27	1.29E-25
FBN2	146.0282	-1.325458	0.1223366	-10.83452	2.36E-27	1.29E-25
ARMC12	30.487537	-1.202092	0.1110197	-10.82773	2.54E-27	1.39E-25
AMZ1	131.98306	-1.526142	0.1409872	-10.82469	2.63E-27	1.44E-25
LINC00475	180.16509	-1.47084	0.1360666	-10.80971	3.10E-27	1.69E-25
CYP26C1	16.672573	-1.938995	0.1795107	-10.80156	3.38E-27	1.84E-25
TEX19	3.7587162	-2.477151	0.2295752	-10.79015	3.83E-27	2.07E-25
NFKBIZ	1462.5701	-1.166819	0.1081614	-10.78776	3.93E-27	2.13E-25
FCHO1	373.58639	-1.049334	0.0973586	-10.77803	4.37E-27	2.36E-25
SP5	75.719106	-2.050894	0.1903193	-10.77607	4.47E-27	2.40E-25
FCRL2	23.061801	-1.812231	0.1683314	-10.76585	4.99E-27	2.67E-25
DPF1	16.184204	-1.100673	0.1022379	-10.76581	4.99E-27	2.67E-25
INSL3	23.559686	-1.234376	0.1146615	-10.76539	5.01E-27	2.68E-25
HOXA13	122.98619	-1.810929	0.1683302	-10.75819	5.42E-27	2.90E-25
ZPLD1	39.178295	-2.338087	0.2173565	-10.75692	5.50E-27	2.93E-25
NTRK1	29.128888	-1.24203	0.1155201	-10.75163	5.82E-27	3.10E-25
IGHV1-69-2	414.09957	-2.202376	0.204982	-10.74424	6.31E-27	3.34E-25
NXF3	12.745201	-1.19585	0.1114025	-10.7345	7.01E-27	3.69E-25
MFAP4	2710.0895	-1.741228	0.162264	-10.73084	7.29E-27	3.83E-25
LAMP5	87.942518	-1.736317	0.1618479	-10.72808	7.51E-27	3.94E-25
SCG5	261.63142	-1.580245	0.1475421	-10.71047	9.09E-27	4.71E-25
PRAME	900.0526	-2.151476	0.2009165	-10.70831	9.30E-27	4.81E-25
IGFL1P1	4.2418878	-3.304737	0.3087347	-10.70413	9.73E-27	5.03E-25
CASP5	27.177527	-1.100124	0.1028203	-10.69948	1.02E-26	5.28E-25
CCDC78	135.37163	-1.642593	0.1535504	-10.69742	1.05E-26	5.39E-25
SCNN1B	227.40779	-1.97736	0.184925	-10.69277	1.10E-26	5.64E-25
AKR1B15	7.5363323	-2.816392	0.2634493	-10.69045	1.13E-26	5.77E-25
LINC01271	13.286156	-1.17487	0.1099123	-10.68916	1.14E-26	5.83E-25
CACNA1G	9.5659036	-1.429063	0.133745	-10.68498	1.20E-26	6.07E-25
LINC01451	20.634529	-1.343911	0.125809	-10.68215	1.23E-26	6.26E-25

LMO1	50.602636	-2.21957	0.2079474	-10.67371	1.35E-26	6.81E-25
INTS4P1	30.029364	-1.367224	0.1281977	-10.66497	1.48E-26	7.45E-25
LINC00242	28.294757	-1.06211	0.0996033	-10.66341	1.51E-26	7.56E-25
ACTBL2	9.9097798	-2.639842	0.2476154	-10.66106	1.55E-26	7.73E-25
ZFHX4	247.99593	-1.4752	0.1384025	-10.65877	1.59E-26	7.92E-25
PTGIS	1284.0549	-1.552405	0.1457004	-10.65478	1.66E-26	8.24E-25
CCDC91	2994.5351	-1.201253	0.1128101	-10.64846	1.77E-26	8.78E-25
SCNN1G	201.36803	-2.574137	0.2417571	-10.64762	1.79E-26	8.85E-25
SLC30A3	23.225009	-1.442668	0.1355098	-10.64623	1.82E-26	8.97E-25
SMPX	3.1340078	-4.178434	0.3927513	-10.63888	1.96E-26	9.68E-25
PNOC	47.862257	-1.554441	0.1461373	-10.63686	2.01E-26	9.88E-25
PRG4	39.762474	-1.290151	0.1213256	-10.6338	2.07E-26	1.02E-24
OTOF	22.791193	-1.124107	0.1057806	-10.62677	2.24E-26	1.10E-24
CDC25C	81.292861	-1.044408	0.09831	-10.62362	2.31E-26	1.13E-24
STEAP1B	31.086958	-1.148444	0.1081704	-10.61699	2.48E-26	1.21E-24
TREML3P	5.1854825	-1.86868	0.1760381	-10.6152	2.53E-26	1.23E-24
LINC00189	22.459225	-1.825637	0.171992	-10.61466	2.55E-26	1.24E-24
SMKR1	42.34095	-1.364635	0.1286506	-10.60729	2.76E-26	1.34E-24
CYP26A1	15.017417	-1.874366	0.1767379	-10.60534	2.81E-26	1.36E-24
ROR2	473.54615	-1.701405	0.1605082	-10.60011	2.98E-26	1.44E-24
CHST4	6.8613797	-1.890909	0.1784658	-10.59535	3.13E-26	1.51E-24
TPBGL	97.747514	-1.502631	0.141855	-10.59273	3.22E-26	1.55E-24
VIPR1	154.54894	1.1824882	0.1116421	10.591778	3.25E-26	1.56E-24
LINC00896	12.436011	-1.794549	0.1695538	-10.58395	3.54E-26	1.69E-24
L1CAM	547.52411	-1.902095	0.1797958	-10.57919	3.72E-26	1.78E-24
CAMK2N2	31.159916	-1.334835	0.1262031	-10.57688	3.81E-26	1.82E-24
CCSER1	114.97306	1.0609398	0.1003342	10.574058	3.93E-26	1.87E-24
SIX2	44.035056	-1.542265	0.1459375	-10.56798	4.19E-26	1.99E-24
MATN4	8.0007191	-1.552117	0.1469022	-10.56565	4.30E-26	2.04E-24
KRT79	2.192495	-2.656043	0.251419	-10.56421	4.37E-26	2.07E-24
ZBP1	161.16988	-1.158649	0.1097624	-10.55598	4.77E-26	2.25E-24
C2	2980.9917	-1.321834	0.1253048	-10.54895	5.14E-26	2.42E-24
GPR150	19.575464	-1.346949	0.1277075	-10.54714	5.24E-26	2.46E-24
APOA4	7.8966421	-5.043226	0.478169	-10.54695	5.25E-26	2.47E-24
PPP1R27	3.6423627	-1.839276	0.1744384	-10.54399	5.42E-26	2.54E-24
ERC2	28.59092	-1.451351	0.1376546	-10.54342	5.45E-26	2.55E-24
NTNG2	195.79252	-1.200755	0.1138893	-10.54317	5.46E-26	2.55E-24
COMP	378.39145	-2.195624	0.2082512	-10.54315	5.46E-26	2.55E-24
BLACAT1	22.204739	-1.608028	0.1526402	-10.53476	5.97E-26	2.78E-24
PLEKHS1	49.579842	-1.996809	0.1895915	-10.53216	6.14E-26	2.85E-24
BMP7	48.204006	-2.233549	0.212078	-10.53173	6.17E-26	2.86E-24
FOXE1	7.5350558	-2.689343	0.255632	-10.52037	6.96E-26	3.21E-24
LINC01503	344.80739	-1.132949	0.1078107	-10.50869	7.88E-26	3.61E-24
CILP	86.965432	-1.393046	0.1325741	-10.50768	7.96E-26	3.64E-24
IGLV4-69	349.92023	-1.991689	0.1895675	-10.50649	8.06E-26	3.69E-24
SLC11A1	806.49502	-1.009062	0.0960672	-10.50371	8.31E-26	3.79E-24
NFE4	5.9723657	-1.551401	0.1477078	-10.50318	8.35E-26	3.81E-24
HAMP	100.22315	-1.523929	0.1451058	-10.50219	8.44E-26	3.85E-24
GPC2	34.172113	-1.007913	0.0960011	-10.49897	8.73E-26	3.97E-24
TUBBP6	6.2890389	-2.801248	0.2668305	-10.49823	8.80E-26	4.00E-24
BEST4	160.25363	-1.528457	0.1456724	-10.49243	9.36E-26	4.24E-24

IGHV3-23	1625.3386	-1.969642	0.1877324	-10.49175	9.43E-26	4.27E-24
LINC00705	2.7496968	-1.998565	0.1905949	-10.48593	1.00E-25	4.53E-24
C1QTNF1	3813.0696	-1.04475	0.0996507	-10.48412	1.02E-25	4.62E-24
STYK1	30.076843	-1.123794	0.1072144	-10.48174	1.05E-25	4.73E-24
LINC01583	4.773858	-2.365387	0.2259344	-10.46935	1.19E-25	5.37E-24
CACNA2D4	444.42267	-1.029813	0.0984466	-10.46063	1.31E-25	5.85E-24
CDK5R2	12.202607	-2.232432	0.2136323	-10.44988	1.47E-25	6.54E-24
E2F7	121.15584	-1.111384	0.1063728	-10.448	1.50E-25	6.66E-24
PYDC1	7.0138596	-2.307508	0.2208983	-10.44602	1.53E-25	6.79E-24
CHRDL1	459.02985	-2.38772	0.228658	-10.44232	1.59E-25	7.04E-24
GAPDHP14	7.6897286	-1.756285	0.1682281	-10.43991	1.63E-25	7.20E-24
ZCCHC12	11.606642	-1.239328	0.1187808	-10.43374	1.74E-25	7.65E-24
MYO1A	37.398217	-1.453264	0.1393068	-10.43211	1.77E-25	7.76E-24
ELFN1-AS1	4.1191725	-2.044646	0.1961146	-10.42577	1.89E-25	8.27E-24
EME2	805.80889	-1.040029	0.0998147	-10.4196	2.02E-25	8.79E-24
SLC8A2	11.249124	-1.640751	0.1576683	-10.40635	2.32E-25	1.01E-23
ASIC1	90.710127	-1.440848	0.1385347	-10.40063	2.46E-25	1.06E-23
RNASE2	89.802528	-1.142643	0.1099019	-10.39693	2.56E-25	1.10E-23
IGLV1-47	835.23622	-2.02554	0.1951033	-10.38188	3.00E-25	1.29E-23
SLC27A2	2480.8048	1.2474938	0.1201792	10.380278	3.05E-25	1.31E-23
IGLV7-43	155.91892	-2.004466	0.1931557	-10.37747	3.14E-25	1.34E-23
MMP23B	5.9187357	-1.469908	0.1416916	-10.37399	3.26E-25	1.39E-23
IGHV3-30	677.44255	-2.012568	0.1940947	-10.369	3.43E-25	1.46E-23
CHRM3	420.48957	1.1972714	0.1154752	10.368211	3.46E-25	1.47E-23
IGHV3-48	172.66318	-2.065548	0.1992278	-10.36777	3.48E-25	1.48E-23
APLP1	242.18865	-1.419932	0.1370848	-10.35805	3.85E-25	1.63E-23
MAT1A	206.09968	-2.455228	0.2370867	-10.35582	3.94E-25	1.66E-23
RYR2	232.64793	-1.630862	0.1575354	-10.35236	4.08E-25	1.72E-23
IGHV1-18	1020.1234	-2.042996	0.1976509	-10.33639	4.82E-25	2.03E-23
HCN2	38.553116	-1.318444	0.1275775	-10.33446	4.92E-25	2.06E-23
IGHV3-21	592.89334	-2.022851	0.1958263	-10.32983	5.17E-25	2.16E-23
KCNK15-AS1	8.066863	-1.722908	0.1669426	-10.32036	5.70E-25	2.38E-23
ENTHD1	6.2273076	-1.624374	0.1574087	-10.31947	5.75E-25	2.40E-23
VWA3A	28.009103	-1.478401	0.1432888	-10.31763	5.87E-25	2.44E-23
ALOX12P2	70.415459	-1.318337	0.127809	-10.3149	6.03E-25	2.50E-23
DQX1	10.40588	-1.874166	0.1817716	-10.31056	6.31E-25	2.62E-23
IGHM	10764.858	-1.863179	0.1809687	-10.29559	7.38E-25	3.03E-23
CLDN11	139.04563	-1.228778	0.1193642	-10.29437	7.47E-25	3.07E-23
IGKV1-5	2092.5549	-1.927316	0.1872504	-10.29272	7.60E-25	3.12E-23
YES1P1	23.765159	-1.300344	0.126615	-10.27006	9.61E-25	3.91E-23
ADAMTSL5	68.210292	-1.163298	0.1133331	-10.26441	1.02E-24	4.14E-23
LIPI	5.0526684	-1.895914	0.1847737	-10.26074	1.06E-24	4.29E-23
PABPC1L	1229.3019	-1.203652	0.1173427	-10.25758	1.09E-24	4.43E-23
MT2P1	16.155526	-1.460801	0.1424338	-10.256	1.11E-24	4.49E-23
ISLR	1369.1625	-1.526456	0.1488704	-10.25359	1.14E-24	4.59E-23
PADI1	569.37168	-1.944029	0.1896894	-10.24849	1.20E-24	4.83E-23
IGKV3OR2-2	12.922933	-2.095531	0.2047599	-10.23409	1.40E-24	5.57E-23
P2RX6	24.824728	-1.267906	0.1238921	-10.23396	1.40E-24	5.57E-23
GYG2	116.65278	-1.909985	0.1868901	-10.21983	1.62E-24	6.41E-23
FBLN1	2347.3801	-1.269037	0.1241924	-10.21831	1.64E-24	6.51E-23
LINC01234	143.02901	-2.627173	0.2572232	-10.21359	1.72E-24	6.83E-23

NPC1L1	159.30772	-2.122412	0.2078829	-10.20965	1.80E-24	7.10E-23
IGHJ3	26.962042	-2.152494	0.2108616	-10.20809	1.82E-24	7.21E-23
VPS9D1-AS1	73.81406	-1.137677	0.111506	-10.20283	1.93E-24	7.59E-23
SNCB	7.1294432	-2.278518	0.2233618	-10.20102	1.96E-24	7.72E-23
ANLN	737.53923	-1.070976	0.1050367	-10.19621	2.06E-24	8.10E-23
IGHV4-55	40.27249	-1.953197	0.1916983	-10.18891	2.22E-24	8.71E-23
PTK6	161.57768	-1.366012	0.1340905	-10.18723	2.26E-24	8.84E-23
WNT2	24.916643	-2.090344	0.2052084	-10.18645	2.28E-24	8.91E-23
VWA3B	8.0161614	-1.206031	0.1184049	-10.18565	2.30E-24	8.97E-23
KRT39	3.0051635	-2.370686	0.2329821	-10.1754	2.55E-24	9.92E-23
TRPM3	1084.6553	1.2843633	0.1263024	10.168954	2.73E-24	1.05E-22
LINC00488	22.623454	-2.328246	0.2291331	-10.16111	2.96E-24	1.14E-22
STMN2	43.69788	-2.393944	0.2356122	-10.16053	2.97E-24	1.14E-22
AMH	59.131867	-1.543485	0.1519452	-10.15817	3.05E-24	1.17E-22
POM121L9P	36.428543	-1.314195	0.1294117	-10.15515	3.14E-24	1.21E-22
CCL21	710.77718	-2.198769	0.2165314	-10.1545	3.16E-24	1.21E-22
KLF17	3.1992318	-2.50536	0.246733	-10.15414	3.18E-24	1.22E-22
MSX2	47.116814	-1.431569	0.1411417	-10.14278	3.57E-24	1.36E-22
IGHV3-49	338.40513	-1.942226	0.1915127	-10.1415	3.62E-24	1.37E-22
ANXA8	9.7858098	-2.566384	0.2533556	-10.12957	4.08E-24	1.55E-22
IGKV1-8	87.28551	-2.065705	0.204069	-10.12258	4.39E-24	1.66E-22
C20orf141	2.0432063	-3.032457	0.2996872	-10.11874	4.56E-24	1.72E-22
FGF7	316.95192	-1.82604	0.1806064	-10.11061	4.96E-24	1.87E-22
FDCSP	51.867742	-3.584091	0.3546528	-10.10592	5.20E-24	1.95E-22
SOWAHB	509.48845	1.0358332	0.1024998	10.105714	5.21E-24	1.95E-22
WNT5B	264.43908	-1.344081	0.1330011	-10.10579	5.21E-24	1.95E-22
TPSG1	248.7361	-2.057432	0.2036104	-10.10475	5.26E-24	1.97E-22
OLFM2	317.17913	-1.111894	0.110052	-10.10336	5.34E-24	2.00E-22
GDF5	15.354755	-1.747017	0.1729337	-10.10224	5.40E-24	2.02E-22
STOML3	8.6203264	1.7789838	0.1764187	10.083875	6.51E-24	2.42E-22
CCL13	42.620301	-1.596964	0.1584948	-10.07582	7.07E-24	2.62E-22
MEG9	10.173641	-1.813391	0.1799951	-10.07467	7.15E-24	2.65E-22
PLAC1	4.4007374	-1.638689	0.1626586	-10.07441	7.17E-24	2.66E-22
IGHV4-28	71.051238	-1.867329	0.1853882	-10.07254	7.31E-24	2.70E-22
SNORD14E	16.439498	-1.733608	0.1721575	-10.0699	7.51E-24	2.77E-22
TNXA	62.724617	-1.600965	0.1590583	-10.06528	7.87E-24	2.89E-22
RGS11	81.830238	-1.250385	0.1242601	-10.06264	8.08E-24	2.96E-22
APOL1	22186.507	-1.210396	0.120294	-10.06198	8.13E-24	2.98E-22
RPSAP52	4.3176375	-2.148218	0.2135654	-10.05883	8.40E-24	3.07E-22
GBA3	4788.5038	1.3204557	0.1313511	10.052868	8.92E-24	3.25E-22
LINC00160	10.157943	-1.987387	0.1977292	-10.05106	9.09E-24	3.30E-22
DAB1	39.29256	-1.656233	0.1650404	-10.03532	1.07E-23	3.85E-22
IGHJ2	10.129517	-2.18868	0.2180982	-10.0353	1.07E-23	3.85E-22
SEMA3A	205.35616	-1.344439	0.1339971	-10.03334	1.09E-23	3.92E-22
ZFPM2-AS1	211.65445	-1.1578	0.1154172	-10.03143	1.11E-23	3.99E-22
KRT25	14.106119	-2.615669	0.2609217	-10.02473	1.19E-23	4.27E-22
PKD2L1	62.608979	-1.398386	0.1395294	-10.02216	1.22E-23	4.37E-22
TNFRSF17	45.285457	-1.686779	0.1684313	-10.01464	1.31E-23	4.70E-22
NKX2-2	3.7767446	-2.52102	0.251999	-10.00409	1.46E-23	5.21E-22
DCST2	44.218211	-1.014918	0.1014915	-10.00003	1.52E-23	5.42E-22
ABCA17P	153.37041	-1.284881	0.1284894	-9.999897	1.53E-23	5.42E-22

NKX3-2	6.2159083	-1.597365	0.1597567	-9.998734	1.54E-23	5.48E-22
KCND3	400.3985	-1.545087	0.1547045	-9.98734	1.73E-23	6.10E-22
CXCL5	767.89833	-2.326087	0.2329164	-9.986788	1.74E-23	6.13E-22
TERT	19.933371	-2.315599	0.2318885	-9.985831	1.76E-23	6.18E-22
IGLV2-14	1903.8328	-1.865218	0.1868573	-9.982044	1.83E-23	6.41E-22
SLC22A31	6.5521137	-2.13104	0.213495	-9.981687	1.83E-23	6.42E-22
ANXA8L1	13.950921	-1.673389	0.1676538	-9.981215	1.84E-23	6.44E-22
FLG-AS1	160.02669	-1.207068	0.1209606	-9.979018	1.88E-23	6.58E-22
HSD17B6	51.76041	-1.19183	0.1195232	-9.971538	2.03E-23	7.08E-22
ACSM4	11.554614	-1.283376	0.1287252	-9.969884	2.06E-23	7.19E-22
SSC4D	106.92929	-1.007019	0.1010287	-9.967647	2.11E-23	7.34E-22
ATP1A3	118.8722	-1.308423	0.1312811	-9.966575	2.13E-23	7.41E-22
CCL19	324.91969	-1.91793	0.1925306	-9.96169	2.24E-23	7.75E-22
SPOCK1	5126.3203	-1.609218	0.1615796	-9.959287	2.30E-23	7.94E-22
EYA2	214.47338	-1.10718	0.1112151	-9.955309	2.39E-23	8.25E-22
PODN	882.4773	-1.391706	0.1397968	-9.955206	2.39E-23	8.25E-22
HOXB-AS4	25.122972	-1.914657	0.1924791	-9.947354	2.59E-23	8.87E-22
COL3A1	44408.465	-1.09911	0.1105434	-9.942793	2.71E-23	9.28E-22
IGHV3-74	603.52826	-1.807823	0.1818722	-9.940075	2.79E-23	9.52E-22
LINC01151	12.142092	-2.758726	0.2775665	-9.938972	2.82E-23	9.62E-22
CCDC80	4241.7737	-1.239377	0.1247078	-9.938248	2.84E-23	9.68E-22
CYP1B1	3276.2026	-1.263696	0.1272048	-9.93434	2.95E-23	1.01E-21
IGKV1D-16	35.820258	-1.99283	0.2007491	-9.926966	3.18E-23	1.08E-21
C1QL2	1.3938093	-2.45752	0.2476055	-9.925145	3.24E-23	1.09E-21
LINC00565	15.68256	-1.125486	0.1134154	-9.923575	3.29E-23	1.11E-21
PTGDS	1275.72	-1.55882	0.1570913	-9.92302	3.31E-23	1.12E-21
GREM1	305.7832	-1.898406	0.1914233	-9.917321	3.50E-23	1.18E-21
CD36	9367.1362	1.0202985	0.1028876	9.9166302	3.52E-23	1.18E-21
LINC01133	7.4476602	-1.775361	0.1790457	-9.915691	3.56E-23	1.19E-21
DBH-AS1	150.11551	-1.098701	0.1108461	-9.911951	3.69E-23	1.24E-21
VGF	24.252075	-1.99084	0.200944	-9.907438	3.86E-23	1.29E-21
IGKV1D-8	41.382901	-1.952838	0.1972179	-9.901926	4.08E-23	1.35E-21
LINC01121	6.4331795	-1.997863	0.2017953	-9.900442	4.14E-23	1.37E-21
NCAM1	1369.1992	-1.792524	0.1811229	-9.896733	4.30E-23	1.42E-21
APOC4-APOC	11.024158	-1.220892	0.1233816	-9.895251	4.37E-23	1.44E-21
ATP2A1-AS1	8.3752857	-1.247958	0.126215	-9.887553	4.71E-23	1.55E-21
SYT12	373.07333	-1.446302	0.1463871	-9.879981	5.08E-23	1.67E-21
HSBP1P2	2.7722044	-2.774573	0.280931	-9.876349	5.27E-23	1.73E-21
RNU4-62P	12.881343	-1.509025	0.1527968	-9.876025	5.29E-23	1.73E-21
POSTN	7390.385	-1.21705	0.123232	-9.876084	5.29E-23	1.73E-21
ICAM5	31.232604	-1.463334	0.148227	-9.872255	5.49E-23	1.79E-21
FAIM2	73.91552	-1.181651	0.1197928	-9.864128	5.95E-23	1.94E-21
AEBP1	27607.231	-1.176497	0.1192919	-9.86234	6.06E-23	1.97E-21
CCIN	10.549707	-1.22897	0.1246363	-9.860453	6.18E-23	2.01E-21
SLAMF8	1248.6805	-1.013144	0.1027595	-9.859375	6.24E-23	2.03E-21
CRABP2	329.85131	-1.721768	0.1746702	-9.85725	6.38E-23	2.07E-21
RORB	25.022565	-1.719684	0.1745327	-9.853072	6.65E-23	2.15E-21
CPLX2	5.1291242	-2.469864	0.2507329	-9.850577	6.82E-23	2.20E-21
CCND2-AS1	10.339075	-1.349854	0.1370458	-9.849657	6.88E-23	2.22E-21
C1QL1	2152.0907	-1.757546	0.1786106	-9.840099	7.56E-23	2.43E-21
PRKCG	6.8690299	-1.92755	0.1961916	-9.824836	8.80E-23	2.81E-21

SCRG1	57.867779	-1.21599	0.1237772	-9.824024	8.87E-23	2.84E-21
NALCN-AS1	5.34324	-2.178519	0.2219378	-9.815898	9.62E-23	3.06E-21
IL31RA	19.575074	-1.428092	0.1455375	-9.812539	9.94E-23	3.16E-21
EFNA5	1923.6434	-1.432225	0.1459681	-9.811908	1.00E-22	3.18E-21
TFAP2A-AS1	21.08152	-1.58091	0.1615718	-9.784564	1.31E-22	4.12E-21
CNFN	64.312767	-1.348461	0.1378607	-9.781332	1.35E-22	4.25E-21
GSG1	40.904543	-1.918822	0.1962494	-9.777466	1.41E-22	4.41E-21
HCAR3	14.238775	-1.758751	0.1799478	-9.773671	1.46E-22	4.57E-21
FNDC4	321.28433	-1.270887	0.1300644	-9.771212	1.50E-22	4.67E-21
PRAC2	4.2570013	-3.014737	0.3085602	-9.770335	1.51E-22	4.71E-21
LRRC15	111.40005	-1.861571	0.1906505	-9.764311	1.60E-22	4.98E-21
SLC6A7	5.1826854	-1.386838	0.1420925	-9.76011	1.67E-22	5.18E-21
HAS2	96.963735	-1.336262	0.1369121	-9.760003	1.67E-22	5.19E-21
SYCE1L	129.178	-1.158206	0.1187416	-9.754007	1.77E-22	5.49E-21
AK5	61.378976	-1.183557	0.1213818	-9.750695	1.83E-22	5.66E-21
FAM90A1	73.023881	-1.043873	0.107099	-9.746805	1.90E-22	5.86E-21
MIR23C	5.3604912	-1.982017	0.2034081	-9.744045	1.96E-22	6.01E-21
IGF2BP2	400.59356	-1.587676	0.1629512	-9.743255	1.97E-22	6.05E-21
HOXA11-AS	56.500831	-1.191788	0.1224938	-9.729373	2.26E-22	6.91E-21
TG	127.69429	-1.022318	0.1051221	-9.725055	2.36E-22	7.18E-21
AGMAT	1936.2285	1.0867351	0.1118719	9.714101	2.63E-22	7.95E-21
UCHL1	1235.906	-1.684609	0.1734247	-9.713778	2.63E-22	7.97E-21
NKX2-5	8.1657385	-2.757868	0.2840673	-9.708503	2.77E-22	8.37E-21
HOXB13	75.940841	-2.830355	0.2917558	-9.701109	2.98E-22	8.95E-21
IGHV1-2	468.2604	-1.940439	0.2000955	-9.697563	3.09E-22	9.24E-21
VTN	90.039144	-1.529134	0.1577262	-9.694865	3.17E-22	9.47E-21
C4orf48	71.043687	-1.128538	0.1165218	-9.685206	3.49E-22	1.04E-20
ERMN	36.027111	-1.089966	0.1125486	-9.684403	3.51E-22	1.04E-20
FOXB1	5.5087012	-1.934084	0.1998846	-9.676005	3.81E-22	1.13E-20
MAP7D2	2273.8872	-1.631013	0.1686066	-9.673486	3.91E-22	1.15E-20
DLL3	5.7500684	-1.892563	0.1957015	-9.67066	4.02E-22	1.18E-20
SRPX	400.64065	-1.144841	0.1184348	-9.666421	4.19E-22	1.23E-20
DCST1	16.084714	-1.090444	0.1128375	-9.663849	4.29E-22	1.26E-20
TAL2	89.170245	1.136894	0.1177374	9.6561877	4.63E-22	1.35E-20
IGLC6	10.282413	-2.157061	0.2234507	-9.653407	4.75E-22	1.39E-20
TSKU	2950.4157	-1.215236	0.1259245	-9.650515	4.89E-22	1.42E-20
GABRA3	6.4432473	-1.844963	0.1913929	-9.639665	5.44E-22	1.58E-20
GOLGA8B	1112.0561	-1.176878	0.1220914	-9.639325	5.45E-22	1.58E-20
FSD1	24.142199	-1.191274	0.1236746	-9.632325	5.84E-22	1.69E-20
HPR	18.287863	-2.397007	0.2490537	-9.624461	6.30E-22	1.82E-20
MNX1-AS2	2.2606777	-1.971178	0.2049602	-9.617368	6.75E-22	1.94E-20
REN	5341.1708	2.1314729	0.2216954	9.6144206	6.95E-22	2.00E-20
IFNE	10.062884	-1.654247	0.1722069	-9.606161	7.53E-22	2.16E-20
KRT34	3.2813466	-2.632838	0.2743395	-9.597009	8.23E-22	2.35E-20
IGKV1-6	292.20921	-1.885424	0.1965496	-9.592613	8.59E-22	2.44E-20
RGS4	420.57885	-1.097652	0.1145195	-9.584844	9.26E-22	2.63E-20
LINC00506	8.0762332	-1.165278	0.1216083	-9.582225	9.50E-22	2.69E-20
IGKV1-9	393.26016	-1.773351	0.1850749	-9.581802	9.54E-22	2.70E-20
GABRG1	27.626537	2.0463516	0.2136179	9.5794947	9.75E-22	2.76E-20
WNT5A-AS1	7.8292854	-1.137228	0.1187261	-9.578587	9.84E-22	2.78E-20
MSC	2529.2313	-1.095073	0.1144108	-9.571411	1.05E-21	2.97E-20

CYP4F3	434.52878	-1.780133	0.1859965	-9.570788	1.06E-21	2.98E-20
LINC00582	5.5044216	-1.777689	0.1857549	-9.570077	1.07E-21	3.00E-20
CSF2	3.1721863	-1.820812	0.1903914	-9.563522	1.14E-21	3.20E-20
LINC00592	2.1777632	-1.732695	0.1814494	-9.549192	1.31E-21	3.65E-20
IL21R-AS1	7.1203203	-1.229377	0.1287425	-9.549121	1.31E-21	3.65E-20
TMEM119	886.25216	-1.216096	0.1273763	-9.547267	1.33E-21	3.71E-20
COL8A2	507.61624	-1.017405	0.1065873	-9.545277	1.36E-21	3.78E-20
BCL2A1	278.18845	-1.041892	0.1093382	-9.529072	1.59E-21	4.41E-20
HSPB6	702.24558	-1.350164	0.1417037	-9.52808	1.60E-21	4.45E-20
SYBU	1387.5755	-1.389784	0.145893	-9.526046	1.63E-21	4.53E-20
KRT14	34.850177	-2.001471	0.2101222	-9.525275	1.65E-21	4.56E-20
RPL23AP77	4.5797368	-2.162415	0.2273461	-9.511556	1.88E-21	5.16E-20
PAGE2	1.8932695	-2.772217	0.2914848	-9.510675	1.89E-21	5.20E-20
IFI30	139.20286	-1.014192	0.1066598	-9.508656	1.93E-21	5.29E-20
PRSS53	201.41255	-1.156765	0.1216687	-9.5075	1.95E-21	5.34E-20
IL21-AS1	23.406957	-2.08267	0.2190576	-9.507406	1.95E-21	5.34E-20
GJB3	32.417905	-1.876255	0.1973906	-9.505289	1.99E-21	5.44E-20
CCDC154	57.994019	-1.268797	0.1336808	-9.491245	2.28E-21	6.18E-20
XKR7	9.0814427	-1.65896	0.1747941	-9.490939	2.29E-21	6.20E-20
C1orf147	9.5502915	-1.095577	0.1154952	-9.485908	2.40E-21	6.50E-20
LINC00520	3.789419	-1.884674	0.1987586	-9.482224	2.49E-21	6.72E-20
IGF2BP3	168.3068	-1.884448	0.198772	-9.48045	2.53E-21	6.83E-20
CEL	33.381566	-1.206412	0.1273173	-9.475633	2.65E-21	7.13E-20
SEC14L5	54.824496	-1.224571	0.1292811	-9.472158	2.74E-21	7.34E-20
C1QTNF1-AS	27.08077	-1.47092	0.1552881	-9.4722	2.74E-21	7.34E-20
PCSK1	68.691989	-1.212351	0.127995	-9.471868	2.75E-21	7.36E-20
IGLV9-49	98.037818	-2.073681	0.2189542	-9.470844	2.78E-21	7.43E-20
MYLK2	18.448245	-1.054156	0.1113762	-9.464823	2.94E-21	7.85E-20
ADAMDEC1	382.43943	-1.485197	0.156983	-9.460879	3.05E-21	8.14E-20
CLCA2	5.8172857	-1.597408	0.1691394	-9.444332	3.58E-21	9.48E-20
SLC7A5	4373.0014	-1.222052	0.1293969	-9.444212	3.58E-21	9.49E-20
C3orf80	56.679673	-1.041865	0.1105199	-9.426953	4.22E-21	1.11E-19
KLK13	8.946119	-2.068333	0.2194263	-9.426094	4.26E-21	1.12E-19
IGKV6D-21	18.925135	-2.334928	0.2478753	-9.419766	4.52E-21	1.19E-19
BRS3	10.585604	-2.497453	0.2652698	-9.414764	4.74E-21	1.24E-19
RASGEF1C	49.276074	-1.680331	0.1786939	-9.403403	5.28E-21	1.38E-19
CR1	188.32465	-1.173665	0.1248765	-9.398608	5.53E-21	1.44E-19
DLGAP1-AS3	5.6840255	-2.222473	0.2366329	-9.392071	5.88E-21	1.53E-19
HSPB3	3.6639153	-2.21256	0.2356268	-9.390105	5.99E-21	1.55E-19
IGLV8-61	309.32791	-1.871981	0.1994377	-9.386297	6.21E-21	1.61E-19
RPL29P14	11.391648	-1.431804	0.152547	-9.385986	6.23E-21	1.61E-19
MYOZ3	76.685589	-1.047379	0.1116057	-9.384637	6.31E-21	1.63E-19
IGHV3-33	402.25546	-1.833265	0.1953914	-9.382528	6.44E-21	1.66E-19
ANKRD36BP2	107.23002	-1.46851	0.156591	-9.378	6.72E-21	1.73E-19
TMEM45A	2903.5548	-1.215303	0.1296204	-9.375868	6.86E-21	1.76E-19
IGHV3-11	456.01663	-1.820191	0.19424	-9.370832	7.20E-21	1.85E-19
LAIR2	33.232292	-1.35581	0.1446928	-9.370267	7.23E-21	1.86E-19
SPATA12	9.0718798	-1.108528	0.1183403	-9.367289	7.44E-21	1.91E-19
ADD2	254.39303	-1.314073	0.1402827	-9.367323	7.44E-21	1.91E-19
LINC00601	1.1204204	-2.421101	0.2586437	-9.36076	7.92E-21	2.02E-19
UNC93A	64.841812	-2.568389	0.2745913	-9.353495	8.48E-21	2.16E-19

SYTL1	347.45962	-1.053262	0.1127385	-9.342522	9.41E-21	2.38E-19
ATP8B3	717.23542	-1.337415	0.1432341	-9.337268	9.89E-21	2.49E-19
LINC00930	6.0370929	-1.720437	0.184412	-9.329316	1.07E-20	2.68E-19
CCDC144NL-	93.005873	-1.084217	0.1162673	-9.325217	1.11E-20	2.78E-19
C5orf46	1010.7892	-1.895898	0.2033881	-9.321579	1.15E-20	2.87E-19
IGKV3D-15	43.852855	-2.164716	0.2323054	-9.318408	1.18E-20	2.96E-19
WNT16	6.9756144	-1.30957	0.1408841	-9.295368	1.47E-20	3.64E-19
MT2A	11181.799	-1.117769	0.1202689	-9.293911	1.49E-20	3.69E-19
MTTP	205.65354	-2.003607	0.2156756	-9.28991	1.54E-20	3.83E-19
TRIM72	10.723318	-1.358677	0.1465023	-9.2741	1.79E-20	4.41E-19
KRTAP2-3	5.2430034	-2.790086	0.3008889	-9.272812	1.81E-20	4.46E-19
GNG8	5.4078633	-1.400319	0.1511051	-9.267189	1.91E-20	4.68E-19
HTRA3	627.07509	-1.01245	0.1092974	-9.263265	1.98E-20	4.84E-19
ASIC3	73.156542	-1.021595	0.1103867	-9.254694	2.15E-20	5.24E-19
SOCS1	433.8712	-1.00951	0.1092163	-9.243221	2.39E-20	5.81E-19
DIO2	388.2553	-1.415303	0.1531508	-9.241239	2.44E-20	5.91E-19
HSPA7	499.67379	-1.126387	0.1219487	-9.236564	2.55E-20	6.15E-19
UGT1A8	28.030997	-2.203245	0.2386825	-9.230863	2.68E-20	6.48E-19
TFAP2C	56.630522	-1.89269	0.2050986	-9.2282	2.75E-20	6.64E-19
KIF1A	12.506909	-1.847669	0.2002832	-9.22528	2.83E-20	6.80E-19
IGHA2	3834.0558	-1.65877	0.1798685	-9.222125	2.91E-20	7.00E-19
HAO2	1346.8216	1.4760438	0.1600694	9.2212758	2.94E-20	7.05E-19
GJB4	21.118897	-1.961868	0.2128886	-9.215465	3.10E-20	7.43E-19
LUM	5185.5788	-1.580368	0.171642	-9.207351	3.34E-20	8.00E-19
ACTG2	1809.7055	-1.149092	0.1248549	-9.203419	3.47E-20	8.29E-19
FGF5	35.476888	-2.284681	0.2482596	-9.202791	3.49E-20	8.33E-19
C4BPA	39.461964	-2.040052	0.221832	-9.196382	3.70E-20	8.82E-19
MCF2	36.768866	1.0371417	0.1127913	9.1952242	3.74E-20	8.91E-19
FOXH1	8.4548541	-1.18124	0.1285671	-9.187729	4.01E-20	9.53E-19
KRT15	54.574791	-1.857242	0.2021808	-9.186045	4.08E-20	9.67E-19
LINC01093	3.0535194	-1.904519	0.2073593	-9.184631	4.13E-20	9.79E-19
IGKV3D-20	97.489517	-1.766391	0.1924188	-9.179929	4.31E-20	1.02E-18
MRGPRF	197.91529	-1.208785	0.1317042	-9.17803	4.39E-20	1.04E-18
FGA	2995.2017	-2.466808	0.2687946	-9.177299	4.42E-20	1.04E-18
PGF	10303.644	-1.304024	0.1421005	-9.176773	4.44E-20	1.05E-18
MUC13	191.7964	-1.567831	0.1708542	-9.176427	4.46E-20	1.05E-18
TMEM174	976.2252	1.7701162	0.1929121	9.1757677	4.48E-20	1.05E-18
DPYSL5	15.541338	-2.147436	0.2340338	-9.175753	4.48E-20	1.05E-18
RN7SL67P	6.1983135	-1.550847	0.1691224	-9.169971	4.73E-20	1.11E-18
LEP	14.569462	-1.823859	0.1989506	-9.167394	4.85E-20	1.13E-18
LHB	3.9888306	-1.327534	0.1448363	-9.165756	4.92E-20	1.15E-18
ARHGEF4	47.182563	-1.423401	0.1553822	-9.160642	5.16E-20	1.20E-18
MOXD1	875.61554	-1.329791	0.1453103	-9.151387	5.62E-20	1.31E-18
CNPY1	5.2185818	-1.942483	0.2122894	-9.150164	5.68E-20	1.32E-18
UNC13A	85.332459	-1.132376	0.1238514	-9.143018	6.07E-20	1.41E-18
LINC00862	14.801984	-1.568017	0.1715625	-9.139624	6.27E-20	1.45E-18
GJB2	1929.2544	-1.211861	0.1328485	-9.122134	7.37E-20	1.70E-18
TBX18	148.47577	-1.198308	0.1314067	-9.119078	7.58E-20	1.74E-18
APOC2	27.122694	-1.14728	0.1258365	-9.117228	7.71E-20	1.77E-18
C1orf210	593.84756	1.1569603	0.1269765	9.1116061	8.12E-20	1.86E-18
TAC3	4.6961846	-1.825446	0.2003488	-9.111342	8.14E-20	1.86E-18

SFRP4	796.22176	-1.061829	0.116668	-9.101286	8.93E-20	2.03E-18
DRAXINP1	1.4866337	-2.562812	0.2816174	-9.100332	9.01E-20	2.05E-18
RANBP17	127.71181	-1.003838	0.1103674	-9.095418	9.42E-20	2.14E-18
CDC42P2	3.0444562	-3.033098	0.3335754	-9.09269	9.66E-20	2.19E-18
IGKV1-39	16.822384	-2.400629	0.2640641	-9.091086	9.81E-20	2.22E-18
IGHV2-5	78.764569	-1.835937	0.202101	-9.084254	1.04E-19	2.36E-18
C17orf50	2.6535678	-1.482777	0.1632367	-9.083605	1.05E-19	2.37E-18
PNPLA5	2.2390378	-2.721366	0.2996137	-9.082917	1.06E-19	2.39E-18
IGKV1D-39	5.504095	-2.421906	0.2667698	-9.078634	1.10E-19	2.48E-18
TUBB8	4.625426	-1.457534	0.1606506	-9.072697	1.16E-19	2.61E-18
MPP2	96.26161	-1.137554	0.125407	-9.070897	1.18E-19	2.64E-18
EVX1	8.8722846	-1.764458	0.1947646	-9.059438	1.31E-19	2.92E-18
MNX1	36.209519	-1.637705	0.1808489	-9.055658	1.36E-19	3.02E-18
SERBP1P3	5.2115762	-1.384693	0.1529347	-9.054145	1.38E-19	3.06E-18
CEACAM22P	9.3672176	-1.376226	0.1520384	-9.051829	1.41E-19	3.12E-18
IGLV1-36	71.858343	-1.905594	0.210576	-9.049436	1.44E-19	3.19E-18
RNU1-47P	7.4707586	-1.427354	0.1577433	-9.048586	1.45E-19	3.21E-18
CASKIN1	19.502318	-1.690653	0.1868788	-9.046786	1.47E-19	3.26E-18
HGFAC	23.19764	-1.452252	0.1605844	-9.043541	1.52E-19	3.36E-18
RPS23P6	20.008344	-1.456591	0.1611093	-9.04101	1.55E-19	3.43E-18
IGKV1OR2-1	34.249109	-1.884395	0.2084673	-9.039283	1.58E-19	3.48E-18
DCSTAMP	39.498043	-1.142628	0.1264157	-9.038656	1.59E-19	3.50E-18
ARID3C	11.884006	-1.199769	0.1327827	-9.035577	1.63E-19	3.59E-18
ZFHX4-AS1	4.3831852	-2.247965	0.2487922	-9.035515	1.63E-19	3.60E-18
LAT	67.510881	-1.102855	0.1221011	-9.032312	1.68E-19	3.70E-18
RTBDN	4.1196273	-1.979338	0.2197819	-9.005917	2.14E-19	4.67E-18
CDCP1	2420.8313	-1.250534	0.1388823	-9.004267	2.17E-19	4.73E-18
HTR2A	21.072832	-1.424469	0.158211	-9.003605	2.18E-19	4.76E-18
SNTG2	15.847942	-1.449793	0.1611315	-8.997577	2.31E-19	5.02E-18
NUTF2P6	3.5098202	-1.379926	0.1534775	-8.991061	2.45E-19	5.32E-18
NDP	13.198465	-1.799502	0.2002565	-8.985986	2.56E-19	5.56E-18
ADAM23	126.45139	-1.077569	0.1199842	-8.980929	2.68E-19	5.81E-18
ADAMTS12	488.21185	-1.011498	0.1126308	-8.980654	2.69E-19	5.82E-18
EYA1	101.52918	-1.761103	0.1961574	-8.978011	2.76E-19	5.95E-18
ENPP7P11	5.420767	-1.325847	0.1478023	-8.970405	2.95E-19	6.35E-18
CEP164P1	15.308144	-1.104375	0.1231331	-8.968951	2.99E-19	6.43E-18
EPS8L3	324.57153	-2.047028	0.2283896	-8.962879	3.16E-19	6.77E-18
IGHV1OR21-	6.6527664	-2.288935	0.255414	-8.961668	3.20E-19	6.84E-18
CAPN11	134.545	-1.06037	0.1183675	-8.958287	3.30E-19	7.05E-18
C2-AS1	6.7337543	-1.476982	0.164878	-8.958026	3.31E-19	7.06E-18
REG3G	55.83145	-2.644837	0.2959078	-8.938045	3.96E-19	8.40E-18
PCK1	8972.1805	1.5797372	0.1767998	8.9351731	4.07E-19	8.60E-18
GXYLT2	541.325	-1.206439	0.1350745	-8.931657	4.20E-19	8.86E-18
RUNDC3A-AS	22.64599	-1.511061	0.1695085	-8.914364	4.91E-19	1.03E-17
CHAT	9.1762828	-3.290749	0.3691655	-8.914023	4.92E-19	1.03E-17
IGHV3-7	50.549123	-1.756631	0.1971375	-8.910689	5.07E-19	1.06E-17
CYP4A11	4707.5569	1.8184797	0.2041484	8.9076347	5.21E-19	1.09E-17
PTH	1.1701799	-2.869211	0.3223237	-8.901643	5.50E-19	1.14E-17
SPIB	68.876615	-1.186714	0.1335885	-8.883358	6.49E-19	1.34E-17
SIGLEC15	66.178002	-1.064499	0.1198385	-8.88278	6.52E-19	1.34E-17
S100B	96.97759	-1.015296	0.1143546	-8.878484	6.78E-19	1.39E-17

IGKV2-28	12.69291	-2.763348	0.311571	-8.869078	7.38E-19	1.51E-17
SEMA3B	1600.8614	-1.028067	0.11599	-8.863412	7.76E-19	1.58E-17
IRF4	258.5929	-1.121934	0.1267178	-8.853795	8.46E-19	1.71E-17
NEUROD4	3.4606306	-3.551432	0.401239	-8.851163	8.66E-19	1.75E-17
LRRN1	28.210271	-1.318336	0.1489604	-8.850245	8.73E-19	1.76E-17
TMEM236	29.909892	-1.003311	0.113394	-8.848013	8.91E-19	1.80E-17
IGHV3-15	727.17157	-1.6511	0.1866565	-8.845661	9.10E-19	1.83E-17
LHFPL3-AS2	483.61776	1.2213276	0.138079	8.8451395	9.14E-19	1.84E-17
LRRC3	214.65286	-1.026149	0.1160588	-8.841631	9.43E-19	1.89E-17
IBSP	75.456426	-1.870298	0.2115592	-8.840539	9.53E-19	1.91E-17
PYGO1	161.71114	-1.043925	0.1180974	-8.839528	9.61E-19	1.93E-17
SCARNA7	22.755093	-1.175155	0.1329598	-8.838422	9.71E-19	1.95E-17
TWIST2	35.627061	-1.184997	0.1341131	-8.835804	9.94E-19	1.99E-17
TUBB2B	139.38297	-1.164628	0.1318297	-8.834338	1.01E-18	2.01E-17
NPFFR2	5.0055082	-1.847267	0.2091215	-8.833465	1.01E-18	2.03E-17
TEX29	17.211437	-1.035915	0.1172967	-8.831573	1.03E-18	2.06E-17
SULT1A3	4.4596029	-1.218993	0.138074	-8.828553	1.06E-18	2.11E-17
CRHBP	136.66839	1.2432495	0.14094	8.821125	1.13E-18	2.25E-17
CFH	5631.6939	-1.052609	0.119342	-8.820109	1.14E-18	2.27E-17
FAM9A	2.2625255	-2.077944	0.2357866	-8.812813	1.22E-18	2.41E-17
SSX1	3.8975015	-3.477493	0.3945952	-8.812812	1.22E-18	2.41E-17
IGKV2D-28	3.6547799	-3.159015	0.3587165	-8.806439	1.29E-18	2.55E-17
ERN2	8.6844174	-1.697418	0.1927703	-8.805393	1.30E-18	2.57E-17
MIR4489	3.3798743	-1.162679	0.1320626	-8.803998	1.32E-18	2.60E-17
HCN4	20.721231	-1.370652	0.1557306	-8.801425	1.35E-18	2.66E-17
NTF4	2.1428067	-1.88651	0.2143506	-8.801047	1.36E-18	2.67E-17
TMIGD1	26.782959	1.0328384	0.1173858	8.7986632	1.38E-18	2.72E-17
TRIM17	53.544493	-1.054555	0.1199351	-8.79271	1.46E-18	2.86E-17
SYT8	39.798739	-2.101985	0.2390832	-8.791857	1.47E-18	2.88E-17
IGHV1-46	549.80594	-1.743661	0.1984307	-8.787253	1.53E-18	2.99E-17
IGKV2-30	45.858619	-1.650595	0.1880799	-8.776026	1.69E-18	3.29E-17
LINC01411	12.501347	-2.520027	0.2872955	-8.77155	1.76E-18	3.41E-17
DNAJC12	233.79494	-1.018922	0.1162741	-8.763102	1.90E-18	3.66E-17
CPA2	4.5964962	-1.286327	0.1469135	-8.75568	2.03E-18	3.89E-17
RNVU1-3	6.6145542	-1.184516	0.1353192	-8.753493	2.07E-18	3.96E-17
S100A7	3.2412309	-2.400366	0.27435	-8.749286	2.15E-18	4.10E-17
LINC01260	16.432592	-1.044876	0.119457	-8.746876	2.19E-18	4.18E-17
ITGB2-AS1	232.23027	-1.044893	0.119502	-8.743733	2.26E-18	4.30E-17
MT1XP1	6.7571476	-1.108947	0.126856	-8.741779	2.29E-18	4.37E-17
SNORD104	33.713478	-1.112882	0.1273189	-8.7409	2.31E-18	4.40E-17
LINC01522	7.9478083	-1.685998	0.1930738	-8.7324	2.49E-18	4.73E-17
IL1A	11.085216	-1.279862	0.1468421	-8.715911	2.88E-18	5.43E-17
MISP	326.19467	-1.62134	0.1860425	-8.714889	2.91E-18	5.47E-17
TRIM29	197.16099	-1.564594	0.1796054	-8.711284	3.00E-18	5.64E-17
TFPI2	3255.5324	-1.391181	0.1598969	-8.700488	3.30E-18	6.18E-17
IGLV3-21	1271.7823	-1.699344	0.195447	-8.694654	3.48E-18	6.50E-17
ASMTL-AS1	255.82387	-1.22889	0.1413934	-8.691284	3.58E-18	6.69E-17
IGKV1OR22-	4.1626533	-2.172189	0.2500743	-8.686176	3.75E-18	6.98E-17
LIN7A	3088.3505	1.0253502	0.1182517	8.6709097	4.29E-18	7.91E-17
IGLC3	2753.5052	-1.616585	0.1864944	-8.668274	4.39E-18	8.09E-17
ARL14	11.595146	-2.360973	0.2723732	-8.668154	4.39E-18	8.09E-17

TPTE2	7.4875493	-1.271516	0.1468899	-8.656252	4.88E-18	8.94E-17
RUNDC3A	126.47447	-1.215571	0.1404678	-8.65374	4.98E-18	9.13E-17
CHRNA1	70.950454	-1.595803	0.1845074	-8.648992	5.20E-18	9.50E-17
SLC13A3	903.60792	-1.797621	0.2079225	-8.64563	5.35E-18	9.77E-17
CPHL1P	30.896616	-1.18961	0.1377487	-8.636094	5.82E-18	1.06E-16
MCIDAS	4.3338852	-2.087465	0.2418938	-8.629677	6.15E-18	1.12E-16
MRGPRF-AS1	2.1591729	-1.729058	0.200422	-8.62709	6.29E-18	1.14E-16
SAP25	10.700497	-1.184163	0.1373531	-8.621306	6.62E-18	1.19E-16
IGKV2-24	212.78184	-1.620334	0.1879612	-8.620578	6.66E-18	1.20E-16
FAM180A	57.776512	-1.335137	0.1549016	-8.619263	6.74E-18	1.21E-16
VSIG1	245.9791	-1.037786	0.120534	-8.609908	7.31E-18	1.31E-16
IGHV1OR15-	12.466191	-1.968783	0.2288535	-8.602808	7.78E-18	1.39E-16
MFAP5	202.89175	-1.49392	0.1736675	-8.602188	7.82E-18	1.40E-16
CRYGN	9.8666441	-1.439742	0.1674179	-8.599689	7.99E-18	1.43E-16
PI15	118.30243	-1.61175	0.1875705	-8.59277	8.49E-18	1.51E-16
ERVV-2	10.044735	-2.381306	0.2771363	-8.592542	8.51E-18	1.51E-16
MMEL1	22.865685	-1.062643	0.1237032	-8.590268	8.68E-18	1.54E-16
ANKFN1	11.087927	-1.81831	0.2116962	-8.589244	8.75E-18	1.55E-16
PCSK6-AS1	2.9621916	-1.715884	0.1998662	-8.585159	9.07E-18	1.61E-16
RNU7-40P	4.8171494	-1.229654	0.1432359	-8.58482	9.10E-18	1.61E-16
CXCL13	424.28407	-1.567553	0.1825998	-8.584636	9.11E-18	1.61E-16
CPN2	279.7785	-2.27338	0.2648973	-8.582117	9.31E-18	1.64E-16
IGLV4-60	77.660185	-2.000291	0.233133	-8.580044	9.48E-18	1.67E-16
LCN2	83.493049	-1.744166	0.2032997	-8.579284	9.55E-18	1.68E-16
RAPSN	14.833136	-1.099318	0.1282443	-8.572064	1.02E-17	1.79E-16
HMGB1P16	1.7695034	-2.341352	0.2731498	-8.571679	1.02E-17	1.79E-16
MS4A8	5.2695982	-2.084334	0.243204	-8.57031	1.03E-17	1.81E-16
ANKRD33	10.992049	-1.499638	0.1750403	-8.567389	1.06E-17	1.85E-16
XKR4	25.315305	-1.539417	0.1797406	-8.564659	1.08E-17	1.89E-16
HMSD	6.7251647	-1.130597	0.1320634	-8.56102	1.12E-17	1.95E-16
RNU6-26P	4.0051265	-1.182854	0.1381787	-8.56032	1.13E-17	1.96E-16
UNC5B-AS1	16.060399	-1.299557	0.1518649	-8.557321	1.16E-17	2.01E-16
LINC01085	4.8891517	-1.191406	0.1392446	-8.556214	1.17E-17	2.03E-16
IGLV3-16	13.48998	-1.958654	0.2289727	-8.554095	1.19E-17	2.06E-16
RN7SL236P	1.8894537	-1.557385	0.1821398	-8.550492	1.23E-17	2.12E-16
FMOD	2008.6405	-1.153013	0.1348647	-8.549404	1.24E-17	2.14E-16
ADRA1D	11.697164	-1.114031	0.1303051	-8.5494	1.24E-17	2.14E-16
KLHDC7B	156.29613	-1.021448	0.1194961	-8.54796	1.25E-17	2.17E-16
UPK3B	59.088561	-1.133714	0.1326326	-8.547775	1.25E-17	2.17E-16
RPS3AP5	54.392389	1.0105597	0.1183036	8.5420899	1.32E-17	2.27E-16
TNN	103.3951	1.1276324	0.1320928	8.5366689	1.38E-17	2.37E-16
PEX5L	51.94247	-1.391135	0.1630265	-8.533183	1.42E-17	2.44E-16
AKR1B10	202.42632	-2.019126	0.2366275	-8.532932	1.43E-17	2.45E-16
HRK	26.949596	-1.615105	0.1893798	-8.528389	1.48E-17	2.54E-16
IGLV2-5	4.0567602	-2.091238	0.2452235	-8.527887	1.49E-17	2.55E-16
IGKV2OR22-	11.753515	-1.888712	0.2215305	-8.525744	1.52E-17	2.60E-16
GRIK2	20.420069	-1.082733	0.1270579	-8.521576	1.57E-17	2.69E-16
NANOGP1	3.0638661	-1.640251	0.1924993	-8.520819	1.58E-17	2.70E-16
MLPH	389.23191	-1.15389	0.1354931	-8.516228	1.65E-17	2.81E-16
LAMB3	2047.2706	-1.326633	0.1557894	-8.515552	1.66E-17	2.82E-16
SNORA73B	31.151976	-1.245328	0.1462593	-8.51452	1.67E-17	2.84E-16

PTGER1	17.662371	-1.663214	0.195349	-8.514063	1.68E-17	2.85E-16
FCF1P7	5.2009171	-1.118539	0.1313933	-8.512913	1.70E-17	2.88E-16
ARL14EPL	1.4935702	-2.537026	0.2981273	-8.509874	1.74E-17	2.95E-16
ACTL8	4.3736943	-2.876307	0.3380864	-8.507608	1.78E-17	3.01E-16
EPS15P1	4.1771006	-1.774317	0.2086151	-8.505219	1.81E-17	3.06E-16
SLC24A3	250.72726	-1.133672	0.1332907	-8.505258	1.81E-17	3.06E-16
SLC16A12	5858.6541	1.1424216	0.1343373	8.5041274	1.83E-17	3.09E-16
VGLL3	445.32598	-1.124256	0.1322365	-8.501858	1.87E-17	3.14E-16
SST	1122.6631	2.3059917	0.2712987	8.4998262	1.90E-17	3.19E-16
RNY3P16	8.7573621	-1.079489	0.1270543	-8.496278	1.96E-17	3.29E-16
SCG2	551.57441	-1.423544	0.167558	-8.495831	1.97E-17	3.30E-16
GRIA1	50.432437	-1.588176	0.186938	-8.495734	1.97E-17	3.30E-16
IL22RA2	2.984615	-2.336902	0.2751755	-8.492406	2.02E-17	3.39E-16
FLJ16779	132.33039	-1.951605	0.2298491	-8.49081	2.05E-17	3.44E-16
LL22NC03-6	3.1630317	-2.004987	0.2361655	-8.489755	2.07E-17	3.47E-16
SYT14	19.65657	-2.327713	0.2744323	-8.481923	2.22E-17	3.70E-16
EPS8L1	466.98743	-1.279423	0.1508541	-8.481193	2.23E-17	3.72E-16
CXCL8	726.41534	-1.413712	0.166873	-8.471783	2.42E-17	4.02E-16
GDNF	19.372366	-1.475433	0.1742645	-8.46663	2.53E-17	4.19E-16
EN1	30.776348	-1.404001	0.1658405	-8.46597	2.54E-17	4.21E-16
LINC00928	2.6982754	-2.040512	0.2411072	-8.463089	2.60E-17	4.31E-16
IGHV1-67	15.070623	-1.936547	0.2288357	-8.462605	2.61E-17	4.33E-16
SLC7A11-AS	2.5618485	-1.336007	0.1579373	-8.459101	2.69E-17	4.44E-16
KRT80	833.93463	-1.07443	0.1270998	-8.453433	2.83E-17	4.65E-16
HMGB2P1	6.7599824	-1.133999	0.1341984	-8.450168	2.91E-17	4.78E-16
PRR33	59.290007	-1.177118	0.1393194	-8.449065	2.94E-17	4.82E-16
IGHV4-4	37.590251	-1.775355	0.2101786	-8.446889	2.99E-17	4.91E-16
TNS4	41.680756	-1.136979	0.1350136	-8.421215	3.73E-17	6.06E-16
HTR1D	14.70355	-1.28914	0.1532488	-8.412073	4.03E-17	6.53E-16
NPIPB11	84.795066	-1.042842	0.1241725	-8.398335	4.53E-17	7.31E-16
AKNAD1	7.6280672	-1.203199	0.1432946	-8.396682	4.59E-17	7.40E-16
LCN1	4.750838	-1.48303	0.1766438	-8.395595	4.64E-17	7.47E-16
CRYBA4	2.4145443	-1.361126	0.1623869	-8.381989	5.20E-17	8.34E-16
GNG4	83.950036	-1.464691	0.1748895	-8.374952	5.52E-17	8.82E-16
ASIP	23.694101	-1.210116	0.1446483	-8.365918	5.96E-17	9.49E-16
MIR4326	1.715866	-1.386178	0.1657067	-8.365251	6.00E-17	9.54E-16
ATP2C2	52.48381	-1.141358	0.1364997	-8.361612	6.19E-17	9.82E-16
HAR1B	5.4097084	-1.158679	0.1386435	-8.357254	6.42E-17	1.02E-15
GRM4	13.328183	-1.324806	0.1586888	-8.348452	6.92E-17	1.09E-15
TBX1	20.484072	-1.367474	0.1638159	-8.34763	6.96E-17	1.10E-15
PKD1L2	138.15709	-1.344459	0.161072	-8.346943	7.01E-17	1.10E-15
MMP7	7325.2484	-1.500263	0.1797392	-8.346891	7.01E-17	1.10E-15
SPINK1	340.98695	-1.675284	0.2007505	-8.345109	7.11E-17	1.12E-15
FAM87A	8.9307193	-1.282318	0.1539096	-8.331636	7.97E-17	1.25E-15
FCRLA	45.964949	-1.205294	0.1446777	-8.330889	8.02E-17	1.25E-15
IGKV1-16	340.8949	-1.65081	0.1982351	-8.327537	8.25E-17	1.29E-15
KRT16	21.621599	-1.919085	0.2305241	-8.324876	8.44E-17	1.32E-15
SSC5D	1042.1013	-1.035999	0.1244518	-8.324499	8.47E-17	1.32E-15
CBX3P7	4.1070958	-1.354731	0.1628591	-8.318429	8.91E-17	1.39E-15
IGKV2D-40	31.807048	-1.89212	0.2274666	-8.318231	8.93E-17	1.39E-15
NPW	5.9769607	-1.253603	0.1507284	-8.316967	9.02E-17	1.40E-15

CPA5	7.3928758	-1.423509	0.1712244	-8.313705	9.28E-17	1.44E-15
MARCO	305.93303	-1.332419	0.1602711	-8.313538	9.29E-17	1.44E-15
IGHV3-73	145.736	-1.593683	0.1919024	-8.304653	1.00E-16	1.55E-15
IGKV4-1	2539.0997	-1.547992	0.1864599	-8.30201	1.02E-16	1.58E-15
CCL11	26.702679	-1.703597	0.205233	-8.300793	1.03E-16	1.59E-15
MMP10	10.909098	-1.490875	0.1797788	-8.292831	1.11E-16	1.70E-15
RAB3B	109.22549	-1.647357	0.198682	-8.291427	1.12E-16	1.72E-15
IGHV4-39	1078.9293	-1.696102	0.2046418	-8.28815	1.15E-16	1.77E-15
SLC7A4	9.590796	-1.525441	0.1841493	-8.283716	1.19E-16	1.83E-15
MIR8071-2	4.8971986	-1.708814	0.2064838	-8.275777	1.28E-16	1.95E-15
MYBPC2	55.07132	-1.142911	0.138128	-8.274294	1.29E-16	1.97E-15
PACERR	7.7131588	-1.292851	0.1562726	-8.273048	1.31E-16	1.99E-15
TMEM59L	46.793648	-1.246971	0.1507692	-8.270725	1.33E-16	2.03E-15
MEG8	3.6447341	-1.497814	0.1812236	-8.265009	1.40E-16	2.12E-15
GABRB3	988.35689	1.1727165	0.1419443	8.2618083	1.43E-16	2.17E-15
MXRA5	3758.6941	-1.124136	0.1360918	-8.260128	1.46E-16	2.20E-15
KCNG1	47.737162	-1.410368	0.1708875	-8.253197	1.54E-16	2.33E-15
AMPD1	19.987714	-1.500619	0.181883	-8.250462	1.58E-16	2.38E-15
LINC01305	2.3926588	-1.80658	0.2189844	-8.249812	1.59E-16	2.39E-15
PROX1-AS1	5.8046592	-1.824398	0.2213934	-8.240526	1.71E-16	2.57E-15
CYP4A22	549.11323	1.7320561	0.2102354	8.2386502	1.74E-16	2.61E-15
IGHV3-53	167.14002	-1.545173	0.1875693	-8.237878	1.75E-16	2.62E-15
CCL7	6.6669087	-1.615954	0.1962682	-8.233398	1.82E-16	2.72E-15
LRFN2	4.0355469	-1.633622	0.1984208	-8.233116	1.82E-16	2.72E-15
RPTN	5.0551615	-2.493904	0.3029913	-8.230941	1.86E-16	2.77E-15
MEG3	528.78157	-1.173846	0.142622	-8.230475	1.86E-16	2.78E-15
IGHV3-72	142.25301	-1.530756	0.1859917	-8.230242	1.87E-16	2.78E-15
WT1-AS	20.028741	-1.625742	0.1976549	-8.225152	1.95E-16	2.90E-15
IGKV2D-29	91.501571	-1.601076	0.1947145	-8.222685	1.99E-16	2.96E-15
BEAN1-AS1	5.4133539	-1.076646	0.1309834	-8.219712	2.04E-16	3.03E-15
IGHV5-78	13.93241	-1.177659	0.1432958	-8.218376	2.06E-16	3.06E-15
SLC34A2	4284.004	-1.780583	0.2168116	-8.212581	2.16E-16	3.20E-15
GK-IT1	4.2569458	-1.306533	0.1591364	-8.210146	2.21E-16	3.27E-15
SOX1	6.1328641	-2.713388	0.330551	-8.208683	2.24E-16	3.31E-15
CTXN3	122.44345	2.2043521	0.2688033	8.2006151	2.39E-16	3.53E-15
LINC01518	1.1685477	-2.278313	0.2778241	-8.200558	2.39E-16	3.53E-15
PROX1	149.35254	-1.122896	0.1369546	-8.199037	2.42E-16	3.57E-15
PLK5	3.8975169	-1.954419	0.2383867	-8.198525	2.43E-16	3.58E-15
IGKV3-7	32.19932	-1.657322	0.2022018	-8.196376	2.48E-16	3.64E-15
CDC37P1	4.392059	-1.351466	0.1649157	-8.194888	2.51E-16	3.68E-15
NRAP	74.133659	-1.848825	0.2256295	-8.194077	2.53E-16	3.71E-15
EML6	283.56188	1.280146	0.1562893	8.1908742	2.59E-16	3.80E-15
P2RX6P	1.3802308	-2.233642	0.2728651	-8.185886	2.70E-16	3.95E-15
CALB2	37.049679	-1.164789	0.1423368	-8.183332	2.76E-16	4.03E-15
SLC5A1	3379.0566	1.4522657	0.1774914	8.1821741	2.79E-16	4.06E-15
PRELP	2788.1891	-1.169851	0.1430831	-8.176026	2.93E-16	4.26E-15
IGLV2-8	542.48387	-1.549093	0.1894744	-8.175735	2.94E-16	4.27E-15
TGM4	4.7062066	-1.201818	0.1470082	-8.175174	2.95E-16	4.29E-15
STAR	9.912929	-1.083866	0.1326727	-8.169479	3.10E-16	4.49E-15
MYH16	18.366294	-1.04848	0.1284692	-8.161337	3.31E-16	4.79E-15
GRIN2A	936.06823	-1.41372	0.1732473	-8.160127	3.35E-16	4.83E-15

CAPN8	24.699024	-1.283251	0.1573078	-8.157583	3.42E-16	4.93E-15
MPPED1	3.3246978	-1.352864	0.1659005	-8.154675	3.50E-16	5.04E-15
TTLL6	300.783	-1.161228	0.1424461	-8.152057	3.58E-16	5.15E-15
ZFP42	1.4739903	-2.134946	0.2620016	-8.148598	3.68E-16	5.29E-15
RNU6-725P	1.2583042	-2.02027	0.2483393	-8.135121	4.12E-16	5.89E-15
SGCD	276.05356	-1.122372	0.1379824	-8.134162	4.15E-16	5.93E-15
NEFM	128.15343	-1.663701	0.2047226	-8.12661	4.41E-16	6.29E-15
RBFOX3	11.924087	-1.160023	0.1428134	-8.122647	4.56E-16	6.47E-15
ZNF280A	1.8240017	-2.513854	0.3096128	-8.119347	4.69E-16	6.64E-15
MTND4P24	39.795909	-1.379872	0.1699782	-8.117938	4.74E-16	6.71E-15
CTXN1	178.79393	-1.086784	0.1338923	-8.116853	4.78E-16	6.76E-15
SLC5A8	2270.0879	1.5522064	0.1912916	8.114348	4.88E-16	6.90E-15
ATP13A4	152.94278	1.2050561	0.1485155	8.1140103	4.90E-16	6.92E-15
CADM3-AS1	46.801903	-1.05157	0.1296042	-8.113702	4.91E-16	6.93E-15
OCSTAMP	1.1218185	-2.24018	0.2761168	-8.11316	4.93E-16	6.96E-15
ROS1	9.6207506	-2.124592	0.2618947	-8.11239	4.96E-16	7.00E-15
NCAM1-AS1	2.9567955	-1.829833	0.2257365	-8.106061	5.23E-16	7.35E-15
IGHV3-35	12.405742	-1.78116	0.2198139	-8.103038	5.36E-16	7.52E-15
TDRD5	21.084898	-1.637664	0.2021589	-8.100878	5.46E-16	7.66E-15
CXCL1	584.04515	-1.396048	0.1723452	-8.1003	5.48E-16	7.68E-15
PLA2G2C	7.8249152	-1.201643	0.1483665	-8.099155	5.53E-16	7.75E-15
PSAT1	1101.3521	-1.313456	0.1621859	-8.09846	5.57E-16	7.79E-15
IGHV6-1	38.7	-1.724045	0.2129032	-8.09779	5.60E-16	7.83E-15
WT1	250.85728	-1.578304	0.1949438	-8.096197	5.67E-16	7.93E-15
LINC00304	11.125365	-1.322274	0.1633629	-8.094094	5.77E-16	8.06E-15
GAPDHS	1.1948105	-1.831427	0.2263116	-8.092499	5.85E-16	8.16E-15
SYT3	22.428923	-1.202641	0.1486221	-8.091942	5.87E-16	8.19E-15
PCDH11X	2.9531027	-1.861675	0.2300856	-8.091228	5.91E-16	8.23E-15
IGHV3OR15-	7.9101496	-1.89397	0.2342072	-8.086728	6.13E-16	8.52E-15
A1CF	2366.6421	1.1571306	0.1431153	8.085306	6.20E-16	8.62E-15
PRSS50	35.774019	-1.121283	0.1387836	-8.07936	6.51E-16	9.03E-15
NPTX1	27.08916	-1.114691	0.1379795	-8.07867	6.55E-16	9.07E-15
VTA1P1	1.5206703	-1.78181	0.2205948	-8.077297	6.62E-16	9.17E-15
TRPC2	178.11519	-1.544124	0.1913254	-8.070668	6.99E-16	9.66E-15
TBC1D3L	41.171157	-1.179179	0.1461366	-8.06902	7.09E-16	9.78E-15
TFR2	189.62821	-1.299964	0.1613983	-8.054382	7.99E-16	1.09E-14
GLYATL1	3566.6234	1.019498	0.1267527	8.0432036	8.75E-16	1.19E-14
SCG3	31.864841	-1.432631	0.1782294	-8.038128	9.12E-16	1.24E-14
C6orf118	3.3635447	-1.702662	0.21194	-8.033701	9.46E-16	1.29E-14
SCN3A	63.530125	-1.497786	0.1864663	-8.032474	9.55E-16	1.30E-14
CXorf65	22.617876	-1.082438	0.1347933	-8.030358	9.72E-16	1.32E-14
LIF	2185.3819	-1.022964	0.1273926	-8.030007	9.75E-16	1.32E-14
MTND5P1	3.2837385	-1.502957	0.1874571	-8.017605	1.08E-15	1.45E-14
CCT5P1	3.0420695	-1.252427	0.1563849	-8.008621	1.16E-15	1.56E-14
PCDH7	331.76857	-1.058405	0.1322114	-8.005398	1.19E-15	1.60E-14
PDGFRA	825.31861	-1.304194	0.162919	-8.005168	1.19E-15	1.60E-14
IGHV3-13	97.835817	-1.589069	0.1985161	-8.004739	1.20E-15	1.61E-14
AVPR1B	231.85564	1.1187589	0.1398033	8.002381	1.22E-15	1.63E-14
EVX1-AS	1.2361119	-2.442731	0.3052881	-8.001394	1.23E-15	1.65E-14
EPHA10	92.575616	-1.594338	0.1994469	-7.993796	1.31E-15	1.75E-14
LINC01013	18.923112	-1.031401	0.1290614	-7.991557	1.33E-15	1.78E-14

IGLV1-50	8.9888224	-1.462571	0.183091	-7.988217	1.37E-15	1.82E-14
SMIM23	2.2192532	-1.586002	0.1985552	-7.987714	1.37E-15	1.83E-14
RNU6-403P	1.7621623	-2.622735	0.3285588	-7.982545	1.43E-15	1.90E-14
IGHV3-47	7.4534129	-1.586011	0.1987153	-7.981323	1.45E-15	1.92E-14
SACS-AS1	1.5913153	-1.560449	0.1955774	-7.978675	1.48E-15	1.96E-14
HMGCS2	2471.5049	1.6289542	0.2042224	7.9763749	1.51E-15	2.00E-14
IGHV2-26	125.56646	-1.70232	0.2135646	-7.970982	1.57E-15	2.08E-14
DUSP13	3.3686329	-1.976213	0.2480417	-7.967259	1.62E-15	2.14E-14
LRRC37A7P	388.88758	1.2121471	0.1521776	7.965343	1.65E-15	2.17E-14
RPL7P18	4.1183457	-1.248017	0.1567156	-7.96358	1.67E-15	2.20E-14
CLVS2	43.367725	1.4698933	0.1845953	7.9627899	1.68E-15	2.21E-14
GBX2	7.2512552	-1.192374	0.1498421	-7.957533	1.76E-15	2.30E-14
RPL22P19	6.0944295	-1.060104	0.133239	-7.956412	1.77E-15	2.32E-14
SEZ6L	20.732906	-1.083942	0.1362764	-7.953997	1.81E-15	2.37E-14
GIPR	70.809194	-1.031543	0.1296961	-7.953538	1.81E-15	2.38E-14
ENPP7P8	108.8733	1.2374312	0.1556707	7.9490317	1.88E-15	2.46E-14
GPR45	7.2814516	-1.162326	0.1462257	-7.948848	1.88E-15	2.46E-14
LINC00524	4.9900743	-2.80661	0.3532097	-7.946017	1.93E-15	2.52E-14
ISCA2P1	2.0088464	-1.510387	0.1901006	-7.945199	1.94E-15	2.53E-14
REG1B	64.079862	-2.041977	0.2570226	-7.944737	1.95E-15	2.54E-14
TRIM67	7.7668135	-1.030235	0.1298221	-7.935743	2.09E-15	2.72E-14
C10orf126	77.119925	1.4220449	0.179287	7.9316681	2.16E-15	2.81E-14
IGLJ2	3.4424097	-1.965556	0.2478909	-7.929117	2.21E-15	2.86E-14
MRPS36P5	1.550643	-2.399452	0.3026206	-7.928912	2.21E-15	2.87E-14
IGHV3OR16-	3.6777505	-2.170673	0.2737739	-7.928705	2.21E-15	2.87E-14
CUBN	25358.814	1.0275059	0.1296622	7.9244805	2.29E-15	2.96E-14
IGHV3-20	30.914283	-1.649049	0.2083147	-7.916143	2.45E-15	3.16E-14
SMCR2	3.1588583	-1.265058	0.1598317	-7.914941	2.47E-15	3.19E-14
CPEB1-AS1	3.3781299	-1.680171	0.2122833	-7.914761	2.48E-15	3.19E-14
LINC01482	7.3112056	-1.078892	0.136365	-7.911795	2.54E-15	3.26E-14
CLEC2L	3.185209	-1.795884	0.2270455	-7.909797	2.58E-15	3.31E-14
IGHV2-70	63.738907	-1.900678	0.2404337	-7.905207	2.67E-15	3.43E-14
MNX1-AS1	4.848961	-2.088513	0.264241	-7.903817	2.70E-15	3.46E-14
SH3GL3	7.2952604	-1.440754	0.182303	-7.903071	2.72E-15	3.48E-14
IGHV3-66	96.680938	-1.608659	0.2036692	-7.89839	2.83E-15	3.61E-14
OTP	2.7593332	-1.508763	0.1910321	-7.897959	2.84E-15	3.62E-14
FAM133A	5.4850077	-1.437061	0.1820135	-7.895352	2.89E-15	3.69E-14
IGHV3OR16-	6.940185	-1.778511	0.2252792	-7.8947	2.91E-15	3.71E-14
LY6H	141.64809	-1.306541	0.1655158	-7.893754	2.93E-15	3.73E-14
MIR222HG	125.53679	-1.168545	0.1481134	-7.88953	3.03E-15	3.86E-14
IGFBP2	1934.2536	-1.067343	0.1353397	-7.886399	3.11E-15	3.94E-14
NMNAT1P3	2.2162007	-1.57064	0.19918	-7.885532	3.13E-15	3.97E-14
KRBOX1	19.944262	-1.111009	0.1409424	-7.882718	3.20E-15	4.05E-14
CYP4F26P	1.7585404	-2.408706	0.3056097	-7.88164	3.23E-15	4.08E-14
HMGB3P7	4.3992347	-1.901206	0.2412247	-7.881474	3.24E-15	4.09E-14
TMEM213	249.04293	2.0832208	0.264431	7.8781272	3.32E-15	4.20E-14
NKX2-8	2.5412475	-2.883648	0.3660755	-7.877195	3.35E-15	4.23E-14
IGKJ5	6.4241992	-1.746898	0.2217893	-7.876387	3.37E-15	4.25E-14
LINC00567	4.0363711	-1.408725	0.178959	-7.871772	3.50E-15	4.40E-14
ULBP1	37.462243	-1.074611	0.1365244	-7.8712	3.51E-15	4.42E-14
SFN	415.16821	-1.390389	0.1766774	-7.869646	3.56E-15	4.47E-14

LOX	20858.168	-1.094454	0.1391739	-7.863931	3.72E-15	4.66E-14
SULT4A1	52.625308	-1.77803	0.2261462	-7.862303	3.77E-15	4.72E-14
AQP8	4.8994784	-1.052249	0.133855	-7.861107	3.81E-15	4.76E-14
HGF	1462.2207	-1.014024	0.1290243	-7.859171	3.87E-15	4.83E-14
KLRG2	13.357333	-1.499971	0.1908934	-7.857638	3.91E-15	4.89E-14
IGHV1-17	3.3365878	-2.12329	0.2703043	-7.855185	3.99E-15	4.98E-14
CR2	30.323325	-1.517189	0.1931739	-7.854004	4.03E-15	5.02E-14
SYT5	13.671398	-1.153257	0.1468937	-7.850958	4.13E-15	5.14E-14
KRTAP5-10	18.193162	-1.054601	0.134421	-7.845509	4.31E-15	5.35E-14
SLC4A3	258.67075	-1.303417	0.166212	-7.841896	4.44E-15	5.50E-14
IGHV3-52	8.6161516	-1.74941	0.2233893	-7.831218	4.83E-15	5.96E-14
EPO	721.62736	-1.989817	0.2542332	-7.826739	5.01E-15	6.15E-14
RNU6-1266F	1.0988785	-1.688761	0.2157872	-7.826051	5.03E-15	6.18E-14
AP3B2	111.0699	-1.173941	0.1500117	-7.825667	5.05E-15	6.20E-14
TTLL2	13.692103	-1.390131	0.1777006	-7.822883	5.16E-15	6.33E-14
SCGB3A2	41.954181	-1.558043	0.1992951	-7.817769	5.38E-15	6.58E-14
RELN	357.88774	-1.421547	0.1818979	-7.815078	5.49E-15	6.71E-14
PILRB	586.65151	-1.051139	0.1345064	-7.814789	5.51E-15	6.72E-14
LILRP2	5.9701217	-1.169714	0.1498578	-7.805495	5.93E-15	7.21E-14
RNA5SP498	5.7596334	-1.203889	0.1544632	-7.794022	6.49E-15	7.85E-14
CYP4F12	141.40308	-1.160511	0.1489176	-7.792975	6.54E-15	7.91E-14
TMEM249	5.1739699	-1.347842	0.1730074	-7.790659	6.67E-15	8.05E-14
IGHV3-62	5.2410394	-1.962464	0.2519674	-7.788561	6.78E-15	8.18E-14
EFCAB8	9.6683607	-1.151505	0.1479729	-7.781864	7.15E-15	8.61E-14
RNA5SP425	1.8660452	-1.471734	0.1893541	-7.77239	7.70E-15	9.25E-14
ABCB5	19.117384	-1.550179	0.1996026	-7.766326	8.08E-15	9.68E-14
ANKRD1	65.539826	-1.088835	0.1402101	-7.765735	8.12E-15	9.72E-14
C1QTNF3	1245.9109	-1.12383	0.1447701	-7.762862	8.30E-15	9.94E-14
ADRA2A	270.10468	-1.060739	0.1367309	-7.757863	8.64E-15	1.03E-13
MANSC4	4.5120762	-1.176638	0.1516765	-7.757548	8.66E-15	1.03E-13
C19orf81	15.962071	-1.533605	0.1976934	-7.757488	8.66E-15	1.03E-13
KRT5	82.645123	-1.613227	0.2079867	-7.756397	8.74E-15	1.04E-13
IGHV3OR16-	10.378328	-1.781546	0.2300152	-7.745343	9.53E-15	1.13E-13
IGLV3-27	73.28574	-1.593857	0.2060019	-7.7371	1.02E-14	1.20E-13
GPR78	4.6163313	-1.507685	0.1948844	-7.736308	1.02E-14	1.21E-13
CD300LG	158.96083	1.2176019	0.1574367	7.733915	1.04E-14	1.23E-13
DPYSL4	139.72189	-1.449018	0.1874115	-7.731743	1.06E-14	1.25E-13
KRT13	29.280161	-1.838487	0.2378138	-7.730785	1.07E-14	1.26E-13
ASCL1	4.5228047	-1.904132	0.2463505	-7.729359	1.08E-14	1.27E-13
MUC16	38.573497	-1.183495	0.1531194	-7.729231	1.08E-14	1.27E-13
MEGF11	492.81854	-1.203633	0.1557831	-7.72634	1.11E-14	1.30E-13
NKX6-3	1.7989694	-1.821195	0.2357344	-7.725621	1.11E-14	1.31E-13
KRT19	12052.855	-1.515499	0.1963053	-7.720114	1.16E-14	1.36E-13
MGAM	4127.5121	1.1125307	0.1441547	7.7176175	1.19E-14	1.39E-13
FMR1NB	1.3783674	-1.54048	0.1996442	-7.716127	1.20E-14	1.40E-13
IGKV1D-43	10.417061	-1.85393	0.2403273	-7.714187	1.22E-14	1.42E-13
CACNG6	2.7784119	-1.427044	0.1850107	-7.713308	1.23E-14	1.43E-13
STRCP1	12.843756	-1.257125	0.1630878	-7.708271	1.28E-14	1.48E-13
CDC20B	20.063502	-1.075404	0.1395473	-7.706379	1.29E-14	1.50E-13
RASL11B	86.625373	-1.114339	0.1447791	-7.696822	1.39E-14	1.61E-13
GPR35	484.5066	-1.08757	0.1413208	-7.695754	1.41E-14	1.63E-13

DSC3	10.718078	-1.293039	0.1680274	-7.695407	1.41E-14	1.63E-13
IGSF23	27.769534	-1.338158	0.1739067	-7.694692	1.42E-14	1.64E-13
CCL18	1024.8261	-1.48583	0.1931038	-7.694464	1.42E-14	1.64E-13
FOSL1P1	6.5783182	-1.380848	0.1795299	-7.691465	1.45E-14	1.68E-13
PCDHGB5	262.20464	1.0620763	0.1381435	7.6882124	1.49E-14	1.72E-13
ST8SIA3	2.6294732	-2.107077	0.2743641	-7.679855	1.59E-14	1.83E-13
TDRG1	1.1229147	-2.112923	0.2751914	-7.678013	1.62E-14	1.86E-13
TCAM1P	6.0332151	-1.312651	0.1709662	-7.677841	1.62E-14	1.86E-13
RNU6-549P	1.4531337	-1.467267	0.1911285	-7.67686	1.63E-14	1.87E-13
LSAMP	45.520188	-1.398451	0.1822571	-7.672956	1.68E-14	1.93E-13
CP	28661.044	-1.268549	0.1654485	-7.667339	1.76E-14	2.01E-13
ANKRD2	73.337632	-1.400083	0.1826399	-7.665808	1.78E-14	2.03E-13
IGKV1-12	33.075521	-1.572783	0.2051933	-7.664885	1.79E-14	2.05E-13
CALML6	9.036665	-1.181876	0.1541999	-7.66457	1.79E-14	2.05E-13
IGHV1-12	8.2695234	-1.6997	0.2218402	-7.661824	1.83E-14	2.09E-13
CACNG4	42.384158	-1.665818	0.2174639	-7.660206	1.86E-14	2.12E-13
IGKV1OR2-3	2.8454657	-2.514564	0.328298	-7.659394	1.87E-14	2.13E-13
DAW1	16.8454	-1.227555	0.1602788	-7.658873	1.88E-14	2.14E-13
RTN4RL1	341.40681	-1.076574	0.1405752	-7.658352	1.88E-14	2.15E-13
IDSP1	6.1769125	-1.189214	0.1552979	-7.657631	1.89E-14	2.15E-13
LKAAEAR1	2.8734446	-1.449086	0.1893352	-7.65355	1.96E-14	2.22E-13
IGLV10-54	157.09446	-1.753264	0.2292387	-7.648204	2.04E-14	2.31E-13
NALCN	131.17041	-1.162393	0.1520236	-7.646134	2.07E-14	2.34E-13
MRPL37P1	8.429829	-1.053793	0.1378622	-7.643815	2.11E-14	2.38E-13
KRT18P40	1.5533608	-1.449671	0.1897004	-7.641896	2.14E-14	2.42E-13
CLEC4GP1	5.0011236	-1.720078	0.2255849	-7.624966	2.44E-14	2.74E-13
LYPD6	42.968864	-1.172353	0.1539078	-7.617243	2.59E-14	2.89E-13
SLC16A9	5219.6851	1.0583754	0.1390726	7.6102369	2.74E-14	3.05E-13
HBE1	6.103293	-1.19463	0.1571036	-7.604092	2.87E-14	3.19E-13
RNU6-1010P	3.8585142	-1.353367	0.1781196	-7.598078	3.01E-14	3.34E-13
C2CD4B	80.597987	-1.097857	0.1446436	-7.590081	3.20E-14	3.54E-13
HLA-DPA3	3.951771	-1.076519	0.1419542	-7.583566	3.36E-14	3.70E-13
SERPINB4	1.515348	-2.725057	0.3593545	-7.5832	3.37E-14	3.71E-13
WFDC12	5.5755785	-2.216228	0.2922616	-7.583029	3.38E-14	3.71E-13
NAT8B	538.83643	1.1669715	0.1541942	7.5681922	3.78E-14	4.14E-13
CYP4F35P	24.768399	-1.112468	0.1469965	-7.567989	3.79E-14	4.14E-13
MMP20	3.215928	-1.640938	0.2168835	-7.565987	3.85E-14	4.20E-13
SNORA77	2.8416115	-1.492536	0.1972773	-7.565676	3.86E-14	4.21E-13
IGHV1-58	52.456341	-1.692865	0.2238113	-7.563805	3.91E-14	4.27E-13
EPYC	8.8137905	-1.51588	0.2004985	-7.560556	4.01E-14	4.37E-13
ENAM	464.85967	1.0837004	0.1433628	7.5591475	4.06E-14	4.42E-13
LINC00668	5.5547834	-1.942844	0.2573136	-7.550491	4.34E-14	4.71E-13
MT1X	3682.4351	-1.155484	0.1530379	-7.550313	4.34E-14	4.71E-13
LINC00840	13.48559	-1.27905	0.1694527	-7.548123	4.42E-14	4.79E-13
FNDC1-IT1	0.726725	-1.758446	0.2331301	-7.542769	4.60E-14	4.98E-13
KLHL30-AS1	5.8584949	-1.136332	0.1508407	-7.533327	4.95E-14	5.34E-13
SMOC1	156.7034	-1.380384	0.1834151	-7.52601	5.23E-14	5.62E-13
MMP27	1.2574948	-2.664652	0.3540894	-7.525365	5.26E-14	5.65E-13
OPN4	20.441846	-1.286043	0.1711175	-7.515557	5.67E-14	6.06E-13
KNG1	174.58125	1.9429494	0.2586102	7.5130417	5.78E-14	6.17E-13
TRAJ31	0.8654167	-1.821901	0.2425493	-7.511468	5.85E-14	6.24E-13

IGHV3-41	7.8707645	-1.699641	0.2265555	-7.502098	6.28E-14	6.68E-13
NPSR1-AS1	5.9406146	-1.922358	0.2562923	-7.500648	6.35E-14	6.75E-13
RNU6-1280F	5.3266983	-1.191392	0.158875	-7.498922	6.43E-14	6.83E-13
WSCD2	41.978055	-1.223606	0.1631809	-7.498463	6.46E-14	6.85E-13
FBLL1	12.593607	-1.214834	0.1621759	-7.490838	6.84E-14	7.24E-13
IGKV2-26	2.8990573	-1.926322	0.2571959	-7.489708	6.90E-14	7.30E-13
HABP2	4103.3043	-1.65974	0.2217098	-7.486095	7.10E-14	7.49E-13
CYP4F23P	3.4796193	-1.87082	0.2500646	-7.481348	7.36E-14	7.75E-13
C20orf203	13.111181	-1.047998	0.1402221	-7.473844	7.79E-14	8.18E-13
FLG	120.84815	-1.069486	0.1431912	-7.46894	8.08E-14	8.48E-13
PLG	1267.3895	1.9961627	0.2673087	7.4676303	8.17E-14	8.56E-13
ETV4	70.406917	-1.064573	0.1426567	-7.462481	8.49E-14	8.88E-13
IGHV3-65	1.6545283	-2.215007	0.2970074	-7.457751	8.80E-14	9.19E-13
HES7	8.7036173	-1.119049	0.1500575	-7.457468	8.82E-14	9.21E-13
MT1M	177.47596	-1.174142	0.1574686	-7.456355	8.89E-14	9.29E-13
GRPR	26.258796	-1.143682	0.1535506	-7.448242	9.46E-14	9.84E-13
TNNT3	53.507084	-1.306272	0.1754258	-7.446293	9.60E-14	9.98E-13
MGAT4EP	3.3028822	-1.447636	0.1944534	-7.44464	9.72E-14	1.01E-12
FOXN4	6.6388905	-1.347521	0.1813195	-7.431752	1.07E-13	1.11E-12
CAPN9	7.5719128	-1.036234	0.1397017	-7.417476	1.19E-13	1.23E-12
ADRB3	3.3990492	-1.193434	0.1609404	-7.415379	1.21E-13	1.25E-12
EEF1DP4	12.945296	-1.029983	0.1389355	-7.41339	1.23E-13	1.27E-12
PROM2	493.78803	-1.330621	0.179491	-7.4133	1.23E-13	1.27E-12
CCR9	10.338691	-1.012843	0.1366435	-7.412304	1.24E-13	1.27E-12
STRC	8.7891164	-1.176246	0.1587451	-7.409653	1.27E-13	1.30E-12
CNTN6	54.0999	-1.392528	0.1879404	-7.409412	1.27E-13	1.30E-12
IGHV1OR16-	2.3757926	-2.217904	0.2994288	-7.407118	1.29E-13	1.32E-12
FCRL4	1.9875842	-1.861073	0.2513468	-7.404402	1.32E-13	1.35E-12
ZNF587P1	2.7863792	-1.2073	0.163115	-7.401531	1.35E-13	1.38E-12
GREM2	19.464773	-1.35755	0.1835311	-7.396841	1.39E-13	1.42E-12
NKAIN4	792.04228	-1.467552	0.1984043	-7.396778	1.40E-13	1.42E-12
NCAM2	32.758775	-1.214165	0.1642589	-7.391778	1.45E-13	1.48E-12
IGLL3P	5.0416237	-1.559432	0.2110398	-7.389282	1.48E-13	1.50E-12
TGM1	199.45825	-1.226496	0.1660084	-7.388158	1.49E-13	1.51E-12
LINC01423	3.4707212	-1.086694	0.1470907	-7.387918	1.49E-13	1.51E-12
KRT42P	2.9651975	-1.24362	0.1683366	-7.387698	1.49E-13	1.52E-12
HS3ST2	398.25757	-1.15042	0.1558145	-7.383267	1.54E-13	1.56E-12
ACE2	5094.7672	1.1584252	0.1569772	7.379577	1.59E-13	1.60E-12
LINC00545	2.2093582	-1.419503	0.1923787	-7.378688	1.60E-13	1.61E-12
YBX2P1	1.9854234	-1.215111	0.1650624	-7.361525	1.82E-13	1.82E-12
RN7SL368P	5.5585377	-1.128223	0.1533987	-7.354841	1.91E-13	1.91E-12
B4GALNT4	187.96341	-1.463834	0.1990496	-7.354114	1.92E-13	1.92E-12
DOCK3	62.9201	-1.025991	0.139622	-7.348349	2.01E-13	2.00E-12
GOLGA8DP	0.6839854	-2.17673	0.2962257	-7.348213	2.01E-13	2.00E-12
MRPS18CP6	1.4593158	-1.40631	0.1914106	-7.347087	2.03E-13	2.02E-12
MAPT-AS1	24.596343	1.4191367	0.1931624	7.3468589	2.03E-13	2.02E-12
GRP	7.3800503	-1.826289	0.2486289	-7.345441	2.05E-13	2.04E-12
DIRAS1	76.606116	-1.34924	0.1838266	-7.339739	2.14E-13	2.12E-12
IGHV3-64	44.225436	-1.529624	0.2084977	-7.336409	2.19E-13	2.17E-12
B3GALT5-AS	10.012006	-1.665792	0.2270683	-7.336082	2.20E-13	2.17E-12
FABP4	713.65149	1.3211239	0.1801151	7.334889	2.22E-13	2.19E-12

ST8SIA5	72.858854	-1.114297	0.1519829	-7.331728	2.27E-13	2.24E-12
NEB	1104.8356	-1.319657	0.1803218	-7.318345	2.51E-13	2.46E-12
LCE1C	1.4521223	-2.550425	0.3487566	-7.312907	2.61E-13	2.56E-12
TRNP1	321.54215	-1.172061	0.160305	-7.311443	2.64E-13	2.58E-12
IGKV3D-11	38.93545	-1.475766	0.2019041	-7.309239	2.69E-13	2.62E-12
ALOXE3	6.8836609	-1.075104	0.1471884	-7.304269	2.79E-13	2.72E-12
NLRP7	11.465641	-1.099641	0.1506137	-7.301072	2.85E-13	2.78E-12
LINC00648	7.0123141	-1.939517	0.2656723	-7.30041	2.87E-13	2.79E-12
KIF5A	34.524048	-1.00879	0.1381837	-7.30035	2.87E-13	2.79E-12
SLC25A2	3.9619186	-1.094397	0.1499162	-7.300058	2.88E-13	2.80E-12
NR4A1	10477.569	1.0038219	0.1375507	7.2978301	2.92E-13	2.84E-12
CBLC	97.49764	-1.153885	0.1581738	-7.295046	2.99E-13	2.90E-12
UBL4B	3.2015899	-1.18383	0.1624169	-7.288837	3.13E-13	3.03E-12
NANOGP6	1.49715	-1.587043	0.2177759	-7.287505	3.16E-13	3.05E-12
SOD1P3	1.2650906	-1.199368	0.1648204	-7.276819	3.42E-13	3.29E-12
IGHV3-19	7.3706762	-1.625557	0.2234353	-7.27529	3.46E-13	3.33E-12
COX6CP2	1.6992355	-1.488231	0.2046753	-7.271183	3.56E-13	3.43E-12
FOSB	10312.898	1.2914446	0.177636	7.2701732	3.59E-13	3.45E-12
H19	2530.2392	-1.189766	0.1636894	-7.268436	3.64E-13	3.49E-12
IGHD	495.89329	-1.377449	0.1895639	-7.266412	3.69E-13	3.54E-12
IGLV1-41	22.248651	-1.878846	0.2585743	-7.266175	3.70E-13	3.55E-12
CHRNB4	15.643346	-1.129006	0.1554319	-7.263668	3.77E-13	3.61E-12
IGLV5-52	3.8778947	-1.135303	0.1563233	-7.26253	3.80E-13	3.64E-12
C19orf33	1876.1382	-1.006182	0.1386131	-7.258921	3.90E-13	3.73E-12
CFAP99	4.8159867	-1.046249	0.1441541	-7.257853	3.93E-13	3.76E-12
KCNG3	2.593891	-1.614026	0.22239	-7.257636	3.94E-13	3.76E-12
LINC01426	775.91415	-1.04136	0.143544	-7.254643	4.03E-13	3.84E-12
CAMK2A	17.249836	-1.005786	0.1386961	-7.251726	4.11E-13	3.92E-12
RCOR2	39.658397	-1.016346	0.1401906	-7.249744	4.18E-13	3.97E-12
TEKT4	6.0973002	-1.218831	0.1681413	-7.248848	4.20E-13	4.00E-12
DIO3OS	75.255625	-1.197572	0.1652331	-7.247773	4.24E-13	4.03E-12
IGKV2OR2-1	1.9408126	-2.103606	0.290264	-7.247215	4.25E-13	4.04E-12
PLA2G3	1.9061312	-2.261454	0.3120705	-7.246612	4.27E-13	4.06E-12
IGHV3-63	6.7072957	-1.404792	0.1939873	-7.241669	4.43E-13	4.20E-12
IGLV3-25	642.23836	-1.344562	0.1858679	-7.233965	4.69E-13	4.43E-12
KCNIP3	811.89606	-1.026327	0.141901	-7.232701	4.73E-13	4.47E-12
TUBA3E	66.174903	-1.825479	0.2526181	-7.226239	4.97E-13	4.67E-12
ISM1-AS1	3.9104843	-1.107941	0.1535135	-7.217221	5.31E-13	4.98E-12
RHCG	576.7165	2.1566235	0.2988401	7.2166467	5.33E-13	4.99E-12
HCN1	3.1936087	-1.517113	0.2103528	-7.212233	5.50E-13	5.15E-12
RN7SL558P	4.6832715	-1.045465	0.1449963	-7.210285	5.58E-13	5.22E-12
LHX3	0.8278195	-2.438249	0.3387579	-7.197616	6.13E-13	5.70E-12
ZIC4	6.4744146	-1.681991	0.233705	-7.197072	6.15E-13	5.73E-12
FAR2P1	6.6519003	-1.47919	0.2055358	-7.196752	6.17E-13	5.74E-12
TGM5	17.777452	-1.430919	0.1988548	-7.195799	6.21E-13	5.78E-12
OR52B3P	3.3719332	-1.021951	0.1420229	-7.195674	6.22E-13	5.78E-12
IGLVI-70	6.8392639	-1.949714	0.2709795	-7.195061	6.24E-13	5.80E-12
NPPB	1.3215723	-1.88709	0.262304	-7.194287	6.28E-13	5.83E-12
TNFRSF13B	18.666252	-1.119726	0.1556513	-7.193808	6.30E-13	5.85E-12
IGKV1D-12	9.0920998	-1.888204	0.2624998	-7.193161	6.33E-13	5.88E-12
HSD17B13	59.247737	1.052539	0.1463302	7.1929055	6.34E-13	5.89E-12

IGLV2-34	6.4814566	-1.620286	0.2254036	-7.188376	6.56E-13	6.07E-12
PTBP1P	3.1399165	-1.085595	0.1510624	-7.186399	6.65E-13	6.16E-12
SEL1L2	4.3315103	-1.21457	0.1691525	-7.180329	6.95E-13	6.42E-12
LRP2	31972.269	1.0017055	0.1395294	7.1791733	7.01E-13	6.47E-12
NKAIN1	154.74148	-1.012885	0.141343	-7.16615	7.71E-13	7.08E-12
IGKV1-13	4.1660159	-1.994984	0.2784577	-7.164407	7.81E-13	7.16E-12
MIR4768	27.073608	-1.233554	0.172327	-7.158215	8.17E-13	7.48E-12
IGKV1-33	6.0546653	-2.011965	0.2812172	-7.154487	8.40E-13	7.67E-12
DCN	4910.3151	-1.247674	0.1744014	-7.154036	8.43E-13	7.70E-12
GJD4	2.8877877	-1.065921	0.149126	-7.14779	8.82E-13	8.03E-12
POTEF	2.4332912	-1.132673	0.1584847	-7.146893	8.88E-13	8.08E-12
IGLV3-22	1.5775796	-2.30538	0.3225914	-7.14644	8.91E-13	8.10E-12
COL19A1	54.528509	1.0772693	0.1508703	7.1403673	9.31E-13	8.45E-12
MAGEB2	1.9252453	-3.42465	0.4798214	-7.137344	9.52E-13	8.63E-12
GNA14-AS1	3.4614396	-1.289537	0.1806905	-7.13672	9.56E-13	8.66E-12
MT1JP	4.0763943	-1.529103	0.214312	-7.134939	9.68E-13	8.76E-12
WNK2	119.27746	-1.257509	0.176297	-7.1329	9.83E-13	8.88E-12
MS4A1	141.46873	-1.027503	0.1440989	-7.13054	1.00E-12	9.03E-12
SLX1A	2.1585473	-1.203944	0.16894	-7.12646	1.03E-12	9.29E-12
ASB18	3.8739398	-1.376725	0.1931938	-7.126134	1.03E-12	9.31E-12
TSPEAR-AS2	18.87919	-1.193007	0.1674282	-7.125482	1.04E-12	9.35E-12
CST6	18.485435	-1.078211	0.1513873	-7.122198	1.06E-12	9.57E-12
COL5A1-AS1	0.7725339	-1.732669	0.2433982	-7.118658	1.09E-12	9.79E-12
IGHV4-59	583.4918	-1.324452	0.1860541	-7.118637	1.09E-12	9.79E-12
IL1RL1	562.96834	1.1499286	0.1615934	7.1161856	1.11E-12	9.96E-12
HHATL	63.646891	-2.135696	0.3001391	-7.115687	1.11E-12	1.00E-11
SLC6A19	3134.6142	1.6395548	0.2306428	7.108631	1.17E-12	1.05E-11
LIPM	17.396433	-1.074468	0.1511769	-7.107352	1.18E-12	1.06E-11
CST5	7.5767162	-2.053034	0.2890057	-7.103786	1.21E-12	1.08E-11
ORM2	6.217233	-1.206619	0.1699596	-7.099447	1.25E-12	1.12E-11
RNU2-38P	0.660534	-1.610945	0.226915	-7.099332	1.25E-12	1.12E-11
IGHV1OR16-	1.4257112	-2.499207	0.3521427	-7.097144	1.27E-12	1.14E-11
LINC01098	2.1296851	-2.095759	0.2954167	-7.094247	1.30E-12	1.16E-11
CYP27C1	12.910266	-1.026555	0.1448578	-7.086641	1.37E-12	1.22E-11
TNIP3	95.832266	-1.062136	0.1499126	-7.085039	1.39E-12	1.23E-11
IGF2	2856.086	-1.228512	0.1734545	-7.082618	1.41E-12	1.25E-11
ARSF	200.54563	1.1705943	0.1653656	7.078827	1.45E-12	1.29E-11
IGHV3-25	2.8700812	-1.829254	0.2584451	-7.077922	1.46E-12	1.29E-11
LEMD1	15.728311	-1.086463	0.153542	-7.075998	1.48E-12	1.31E-11
GLP2R	88.667891	-1.519267	0.2147298	-7.07525	1.49E-12	1.32E-11
KLK4	54.366535	-2.387764	0.3375543	-7.073718	1.51E-12	1.33E-11
KRT6A	29.636694	-2.101775	0.297575	-7.063012	1.63E-12	1.43E-11
PITX1	101.90257	-1.769813	0.2509105	-7.053565	1.74E-12	1.52E-11
SLC25A48	234.86426	1.0528993	0.1493575	7.0495248	1.80E-12	1.57E-11
TMEM151A	14.979607	-1.372832	0.1948105	-7.047009	1.83E-12	1.59E-11
FCN2	40.460451	1.1628104	0.1650639	7.0446083	1.86E-12	1.62E-11
SYNGR3	51.746636	-1.038261	0.1474528	-7.041311	1.90E-12	1.66E-11
IGKV2D-24	10.483639	-1.430323	0.2031501	-7.040722	1.91E-12	1.66E-11
IGFL3	1.1113009	-1.807939	0.2568756	-7.038188	1.95E-12	1.69E-11
LINC01563	1.7029702	-1.628707	0.2316129	-7.03202	2.04E-12	1.76E-11
EEF1A1P31	5.5809162	-1.099006	0.1563126	-7.030823	2.05E-12	1.78E-11

ASB17	6.8651743	1.194195	0.1700063	7.0244142	2.15E-12	1.86E-11
KRT75	1.7343267	-2.373325	0.3378699	-7.024375	2.15E-12	1.86E-11
RNU1-67P	4.2988426	-1.189847	0.16948	-7.020574	2.21E-12	1.91E-11
DRD2	9.4004281	-1.113642	0.1586405	-7.019912	2.22E-12	1.91E-11
ARHGAP40	42.887749	-1.81608	0.2587348	-7.019078	2.23E-12	1.92E-11
LINC01060	107.48051	1.1541252	0.1645488	7.0138763	2.32E-12	1.99E-11
RPL7L1P9	9.2312464	-1.070645	0.1529464	-7.000134	2.56E-12	2.19E-11
WFDC2	4051.0047	-1.162208	0.1660368	-6.999702	2.57E-12	2.20E-11
MIA	3.8079753	-1.224233	0.1752079	-6.987317	2.80E-12	2.39E-11
PTPRZ1	17.295011	-1.314702	0.188248	-6.983886	2.87E-12	2.45E-11
AGR2	36.133633	-1.762744	0.2524245	-6.983254	2.88E-12	2.46E-11
GUCY2EP	4.598348	-1.604852	0.2298981	-6.980711	2.94E-12	2.50E-11
GCSAML-AS1	5.4695078	-1.068756	0.1532439	-6.974219	3.08E-12	2.61E-11
KRTAP16-1	2.1077173	-1.428347	0.2048442	-6.972842	3.11E-12	2.63E-11
CPB1	40.175512	-1.30454	0.187155	-6.970375	3.16E-12	2.67E-11
MYADML2	1.1948698	-1.248347	0.1791338	-6.968796	3.20E-12	2.70E-11
MKX	44.803004	-1.127834	0.1618438	-6.968661	3.20E-12	2.70E-11
MIR137HG	1.2827368	-2.058244	0.2953817	-6.968083	3.21E-12	2.71E-11
OR7E100P	3.2765414	-1.48724	0.2136224	-6.962004	3.35E-12	2.82E-11
PTGES2-AS1	5.5417014	-1.049444	0.1508078	-6.958821	3.43E-12	2.89E-11
CHI3L1	1477.8881	-1.035989	0.1489124	-6.957032	3.48E-12	2.92E-11
PGA4	1.2906552	1.8166924	0.2613895	6.9501365	3.65E-12	3.06E-11
TACR3	3.2961158	1.8989067	0.2732661	6.9489286	3.68E-12	3.08E-11
LINC01050	0.7875384	-2.123092	0.3055517	-6.94839	3.69E-12	3.10E-11
PTPRJ-AS1	1.9206064	-1.412928	0.2034907	-6.943453	3.83E-12	3.20E-11
ZNF705G	1.1892774	-2.401517	0.3459773	-6.941256	3.89E-12	3.25E-11
PRELID2P1	1.9676058	-1.117166	0.1609674	-6.940326	3.91E-12	3.27E-11
FFAR3	3.5979538	-1.192746	0.1719133	-6.938064	3.98E-12	3.32E-11
LINC01122	4.9411405	-1.089339	0.1572982	-6.92531	4.35E-12	3.62E-11
IGHV1-68	1.2538147	-1.922236	0.2776022	-6.924426	4.38E-12	3.64E-11
FAM169B	55.349441	-1.180836	0.1706199	-6.920859	4.49E-12	3.73E-11
IGKV1D-42	5.7123782	-1.479954	0.2138851	-6.919388	4.54E-12	3.76E-11
F11	68.269592	1.2351089	0.1785626	6.9169536	4.61E-12	3.82E-11
FABP6	1429.4342	-1.010445	0.146194	-6.91167	4.79E-12	3.96E-11
CCL25	4.7904484	-1.235958	0.1790084	-6.904467	5.04E-12	4.15E-11
ARHGDIG	3.4045813	-1.303972	0.1892745	-6.889317	5.61E-12	4.60E-11
PPIAP14	2.216057	-1.23049	0.178779	-6.882746	5.87E-12	4.80E-11
SERPINA9	4.9831981	-1.663285	0.2417117	-6.881276	5.93E-12	4.85E-11
LINC00923	6.8334018	-1.303589	0.1895722	-6.876477	6.14E-12	5.02E-11
UGT1A7	18.38058	-1.11504	0.1621896	-6.874918	6.20E-12	5.07E-11
CHRNA9	1.6890496	-1.558264	0.2267893	-6.870978	6.38E-12	5.20E-11
MT1E	2772.5142	-1.041349	0.1515684	-6.870488	6.40E-12	5.22E-11
CES5A	6.2071903	-1.773727	0.2582818	-6.86741	6.54E-12	5.32E-11
XDH	56.227003	-1.511836	0.2203762	-6.860251	6.87E-12	5.59E-11
FLG2	6.7336029	-1.007878	0.1469404	-6.859092	6.93E-12	5.63E-11
IGHV3-22	3.0766367	-1.81412	0.2645269	-6.857978	6.98E-12	5.67E-11
IGHV3-71	9.798264	-1.561028	0.2276304	-6.857731	7.00E-12	5.68E-11
RNU6ATAC39	1.6734654	-1.504861	0.219577	-6.853454	7.21E-12	5.84E-11
FAM83A	8.3681398	-1.483121	0.2166086	-6.84701	7.54E-12	6.09E-11
CPZ	14.437447	-1.262042	0.1843786	-6.844838	7.66E-12	6.17E-11
CELA2A	2.559491	-1.105016	0.1614377	-6.844845	7.66E-12	6.17E-11

IFNB1	0.729335	-1.385893	0.2027123	-6.836751	8.10E-12	6.52E-11
STK31	16.83327	-1.018057	0.1489659	-6.834159	8.25E-12	6.62E-11
MYBPH	8.7245904	-1.053705	0.1541905	-6.833787	8.27E-12	6.64E-11
OSTN-AS1	5.6900552	-1.136328	0.1663071	-6.832706	8.33E-12	6.69E-11
SEMA3B-AS1	14.803853	-1.094376	0.1602784	-6.82797	8.61E-12	6.90E-11
IGSF9	43.07671	-1.06456	0.1559803	-6.824967	8.79E-12	7.04E-11
BMP5	73.135541	1.4679835	0.2151545	6.822926	8.92E-12	7.13E-11
TPTEP1	278.93427	-1.05788	0.1550883	-6.821145	9.03E-12	7.21E-11
TENM2	259.11025	-1.152095	0.1689261	-6.820112	9.10E-12	7.26E-11
TNR	34.456507	-1.18245	0.173411	-6.81877	9.18E-12	7.32E-11
MIR196A1	4.0661743	-1.61712	0.2372451	-6.816243	9.35E-12	7.45E-11
FGL1	7.121154	-1.632392	0.2394886	-6.816155	9.35E-12	7.45E-11
SALL4	40.837787	-1.134947	0.1668682	-6.801456	1.04E-11	8.22E-11
MUC5B	27.24507	-1.05195	0.1549883	-6.787283	1.14E-11	9.03E-11
MIR6753	3.4777823	-1.035845	0.1526356	-6.78639	1.15E-11	9.08E-11
ALX1	15.052879	-1.296215	0.1911901	-6.779717	1.20E-11	9.47E-11
SHD	3.3385049	-1.203377	0.177661	-6.773448	1.26E-11	9.87E-11
MESTIT1	7.2339478	-1.180603	0.1744125	-6.769025	1.30E-11	1.02E-10
TRIML2	3.8898901	-1.307919	0.1933239	-6.765431	1.33E-11	1.04E-10
KLB	74.879183	-1.173046	0.173448	-6.763096	1.35E-11	1.06E-10
KRT31	1.7182813	-1.476058	0.2183528	-6.759969	1.38E-11	1.08E-10
TSPEAR-AS1	15.790798	-1.146114	0.169651	-6.755712	1.42E-11	1.11E-10
TCF23	15.096933	-1.08565	0.1608312	-6.750242	1.48E-11	1.15E-10
LINC00836	1.887003	-2.143088	0.3175172	-6.749518	1.48E-11	1.15E-10
LINC01470	6.0427797	-1.050742	0.155713	-6.747941	1.50E-11	1.17E-10
PCP4	72.595464	-1.732651	0.2568933	-6.744631	1.53E-11	1.19E-10
RGS7	120.82248	1.7580024	0.2611787	6.731034	1.68E-11	1.30E-10
LCNL1	9.8032918	-1.153356	0.1713588	-6.730651	1.69E-11	1.30E-10
TUBAL3	13.792766	-1.878845	0.2796231	-6.719205	1.83E-11	1.40E-10
GABRB2	57.003929	1.0849575	0.161549	6.7159673	1.87E-11	1.43E-10
PPIAL4C	1.2300792	-1.345488	0.2003568	-6.715459	1.87E-11	1.44E-10
PPY	1.0539983	-1.456468	0.216903	-6.714837	1.88E-11	1.44E-10
OR2H2	1.2897506	-1.415582	0.2110905	-6.706044	2.00E-11	1.53E-10
KRTAP5-1	8.3054659	-1.086801	0.1620813	-6.705286	2.01E-11	1.54E-10
SPATA21	9.8415731	-1.102693	0.1645613	-6.700803	2.07E-11	1.58E-10
LINC01500	1.5995488	-1.315685	0.1964023	-6.698929	2.10E-11	1.60E-10
DDX39BP2	2.6097414	-1.250001	0.1866347	-6.69758	2.12E-11	1.61E-10
RAMP1	258.72918	-1.142652	0.1706949	-6.694117	2.17E-11	1.65E-10
IGKV1D-33	2.006892	-2.146569	0.3206875	-6.693647	2.18E-11	1.65E-10
IGHV3OR16-	3.327809	-1.62337	0.2425933	-6.691735	2.21E-11	1.67E-10
NKD2	566.96481	-1.123246	0.1678614	-6.691506	2.21E-11	1.68E-10
DPP10	5.4501345	-1.617772	0.2417998	-6.690542	2.22E-11	1.69E-10
NOTUM	7.1024366	-1.173092	0.1753389	-6.690427	2.23E-11	1.69E-10
CSRP3	1.513169	-1.411395	0.2110182	-6.688497	2.25E-11	1.71E-10
CES1	384.68144	-1.109207	0.1660477	-6.680052	2.39E-11	1.80E-10
OR1H1P	1.9054774	-1.672902	0.2504951	-6.678382	2.42E-11	1.82E-10
FAM83E	9.2950442	-1.261161	0.1888588	-6.677797	2.43E-11	1.83E-10
MIR499A	2.657499	-1.03987	0.1558351	-6.672887	2.51E-11	1.89E-10
LGALS12	318.75209	-1.195211	0.1791821	-6.670372	2.55E-11	1.92E-10
CCL1	0.7759181	-1.661162	0.2490413	-6.670225	2.55E-11	1.92E-10
RPS15AP6	3.401437	-1.09956	0.1648526	-6.669959	2.56E-11	1.92E-10

LPO	5.8640206	-1.093583	0.1639954	-6.668374	2.59E-11	1.94E-10
PTGER3	4811.165	1.0255456	0.1538024	6.6679434	2.59E-11	1.95E-10
IGHV3-76	2.6817036	-1.740436	0.2612521	-6.661903	2.70E-11	2.03E-10
PSG5	5.5006734	-1.98771	0.2986832	-6.654911	2.83E-11	2.12E-10
RN7SL233P	3.1622243	-1.183411	0.1778766	-6.652987	2.87E-11	2.15E-10
PTCSC3	10.175519	1.3793846	0.2074884	6.6480071	2.97E-11	2.21E-10
IGLJCOR18	1.1103684	-3.150797	0.4740632	-6.646365	3.00E-11	2.24E-10
TMEM190	1.1279044	-1.490361	0.2243216	-6.64386	3.06E-11	2.27E-10
ATP6V0E1P2	1.5604712	-1.086743	0.1637714	-6.635729	3.23E-11	2.40E-10
LINC00643	4.8095612	-1.059882	0.1598552	-6.63026	3.35E-11	2.48E-10
LRTM1	1.5424922	-1.77627	0.2680599	-6.626391	3.44E-11	2.55E-10
KIRREL2	12.357474	-1.165761	0.1763325	-6.611149	3.81E-11	2.80E-10
GOLGA8IP	14.179305	-1.028924	0.1556442	-6.610743	3.82E-11	2.81E-10
LINC00595	2.2618372	-1.056993	0.1601152	-6.601454	4.07E-11	2.98E-10
RBFOX1	7.0060482	-1.352526	0.2052448	-6.589821	4.40E-11	3.21E-10
OR1J1	0.8490905	-1.548605	0.2350092	-6.589551	4.41E-11	3.21E-10
IGLV3-12	10.593068	-1.278403	0.1940103	-6.589359	4.42E-11	3.21E-10
SNX18P7	1.1741098	-2.288798	0.3477535	-6.58167	4.65E-11	3.37E-10
IGKV7-3	2.258759	-1.663097	0.2527164	-6.580883	4.68E-11	3.39E-10
APOB	1848.7955	-1.402955	0.2132061	-6.580277	4.70E-11	3.40E-10
OR7E62P	1.1315899	-1.544227	0.2346947	-6.579726	4.71E-11	3.41E-10
ESPNP	26.206261	1.0697061	0.1627761	6.5716393	4.98E-11	3.59E-10
IGKV1D-27	7.2324032	-1.680043	0.2556558	-6.571502	4.98E-11	3.59E-10
SEMA3D	544.55418	1.0772322	0.1641273	6.5633926	5.26E-11	3.78E-10
IGHV4OR15-	1.4153307	-2.1183	0.3227666	-6.562944	5.28E-11	3.79E-10
CCNYL2	133.62726	-1.009489	0.153859	-6.561136	5.34E-11	3.84E-10
SPDEF	32.698545	-1.180436	0.1803561	-6.545029	5.95E-11	4.26E-10
BCHE	133.80548	-1.2122	0.1852142	-6.544853	5.96E-11	4.26E-10
IGLV5-48	6.4429356	-1.635662	0.2499933	-6.542824	6.04E-11	4.32E-10
IGHV3-60	5.5523104	-1.524003	0.2330206	-6.540205	6.14E-11	4.39E-10
VCX3A	1.0917585	-1.67535	0.2562525	-6.537888	6.24E-11	4.45E-10
KRT6C	1.8639436	-2.547391	0.3896583	-6.5375	6.26E-11	4.46E-10
AWAT2	1.5622157	-1.326014	0.2028691	-6.536301	6.31E-11	4.50E-10
SLCO6A1	1.887996	-1.824446	0.2792383	-6.533654	6.42E-11	4.57E-10
IGHV1-3	25.603309	-1.53385	0.2348033	-6.53249	6.47E-11	4.61E-10
MYBPHL	2.3523729	-1.045356	0.1601845	-6.525952	6.76E-11	4.80E-10
FOXA2	46.298565	-1.515413	0.2322445	-6.525078	6.80E-11	4.82E-10
CICP27	2.3983873	-1.183022	0.1813486	-6.523468	6.87E-11	4.87E-10
IGHV3-36	0.9393291	-1.910757	0.2929643	-6.522149	6.93E-11	4.91E-10
MIR31HG	13.298241	-1.294075	0.1987053	-6.512536	7.39E-11	5.22E-10
PRELID1P2	1.4310563	1.4066605	0.2160096	6.5120288	7.41E-11	5.23E-10
DMRT2	33.480872	-1.697125	0.2607286	-6.509161	7.56E-11	5.33E-10
CYP2C9	102.99799	-1.270329	0.1952546	-6.506013	7.72E-11	5.44E-10
UGT3A2	24.626548	-1.124561	0.1728771	-6.50497	7.77E-11	5.47E-10
KRT8P30	1.929811	-1.316112	0.202342	-6.504392	7.80E-11	5.49E-10
OR2A4	71.448073	-1.022501	0.1572431	-6.502678	7.89E-11	5.55E-10
SNORD93	2.4444574	-1.003141	0.1543159	-6.500571	8.00E-11	5.63E-10
SERPINA12	11.389663	-1.80249	0.2774583	-6.496438	8.22E-11	5.77E-10
HPCAL4	51.209932	-1.204675	0.1854524	-6.495871	8.26E-11	5.79E-10
IGKV1OR2-6	6.4669311	-1.474034	0.2272194	-6.487271	8.74E-11	6.11E-10
IGHV1-45	11.966581	-1.204232	0.1857765	-6.482156	9.04E-11	6.31E-10

LHCGR	14.800026	-1.965038	0.3036085	-6.472274	9.65E-11	6.71E-10
LY6D	5.1027666	-1.718684	0.265671	-6.46922	9.85E-11	6.84E-10
AK3P2	1.2652472	-1.293579	0.2001768	-6.462182	1.03E-10	7.14E-10
TRAV27	17.997522	-1.108735	0.1719005	-6.449866	1.12E-10	7.71E-10
FAM9B	9.2624509	-1.20117	0.1862387	-6.449624	1.12E-10	7.72E-10
MC4R	1.6779084	-1.868789	0.2898405	-6.447645	1.14E-10	7.81E-10
TLX2	1.263023	-1.368384	0.212339	-6.444338	1.16E-10	7.97E-10
COLEC12	513.38555	-1.000984	0.1553542	-6.44324	1.17E-10	8.03E-10
IGKV2D-30	6.4271265	-1.774545	0.2755228	-6.440646	1.19E-10	8.15E-10
BFSP2-AS1	0.9397778	-1.33637	0.2074933	-6.440547	1.19E-10	8.16E-10
TOX3	401.34334	1.1535331	0.1792997	6.4335466	1.25E-10	8.52E-10
LPA	14.565546	1.3036444	0.2026676	6.4324269	1.26E-10	8.57E-10
SFRP2	2932.3863	-1.301782	0.2025678	-6.426402	1.31E-10	8.89E-10
EEF1A2	582.4453	-1.449096	0.2254932	-6.426341	1.31E-10	8.90E-10
PDCL2	1.4246536	-1.970594	0.3067749	-6.423582	1.33E-10	9.05E-10
PRSS29P	1.3904388	-2.205815	0.3436534	-6.41872	1.37E-10	9.32E-10
CERS1	27.022056	-1.083197	0.1689332	-6.411986	1.44E-10	9.71E-10
IGHV3OR16-	2.2111392	-1.756066	0.2739647	-6.409825	1.46E-10	9.85E-10
ZNF804B	10.409155	1.8487124	0.2887743	6.4019292	1.53E-10	1.03E-09
LGI3	14.185151	-1.125069	0.1759573	-6.39399	1.62E-10	1.09E-09
MYL2	2.0623353	-1.162699	0.1818675	-6.393112	1.63E-10	1.09E-09
SLITRK2	346.23629	-1.185232	0.1855667	-6.387094	1.69E-10	1.13E-09
VIL1	1470.8152	1.1056946	0.1732158	6.3833361	1.73E-10	1.16E-09
THEG	2.6703844	-1.506574	0.2360271	-6.383054	1.74E-10	1.16E-09
SLC6A18	683.61008	1.587187	0.2487022	6.3818779	1.75E-10	1.17E-09
SUMO1P1	1.2028239	-1.100516	0.1724524	-6.381563	1.75E-10	1.17E-09
ANKRD30BP3	2.6226109	-1.482611	0.2323794	-6.380133	1.77E-10	1.18E-09
MFSD6L	18.040288	-1.094801	0.1716894	-6.376635	1.81E-10	1.21E-09
GPR143	496.32711	-1.127453	0.1768242	-6.376126	1.82E-10	1.21E-09
NT5DC4	9.8670132	-1.035481	0.1624038	-6.375967	1.82E-10	1.21E-09
CLPSL2	0.8073493	-2.007516	0.3149618	-6.37384	1.84E-10	1.23E-09
IGHV3-6	1.522436	-1.900132	0.2986013	-6.363442	1.97E-10	1.31E-09
IL21	0.766284	-1.675611	0.2633825	-6.361892	1.99E-10	1.32E-09
RPSAP41	1.2996095	-1.088252	0.1711007	-6.360299	2.01E-10	1.33E-09
IGLV2-33	3.442458	-1.577829	0.248246	-6.355907	2.07E-10	1.37E-09
REG3A	2.7292412	-1.970922	0.3102498	-6.352694	2.12E-10	1.40E-09
IGHV7-27	2.1298467	-1.57478	0.2479392	-6.351479	2.13E-10	1.41E-09
MIR148A	1.5012943	-1.204722	0.189724	-6.349866	2.16E-10	1.42E-09
IGHJ3P	6.7602459	-1.576837	0.2483597	-6.349003	2.17E-10	1.43E-09
TUBBP9	1.4110447	-1.430293	0.2252861	-6.348785	2.17E-10	1.43E-09
TUBA3D	775.68422	-1.417233	0.223354	-6.345231	2.22E-10	1.46E-09
IGKV6-21	39.281438	-1.317653	0.2084671	-6.320676	2.60E-10	1.70E-09
NPTX2	12085.015	-1.11525	0.1764901	-6.319052	2.63E-10	1.71E-09
ZBED2	85.76421	-1.035435	0.1638839	-6.318099	2.65E-10	1.72E-09
FA2H	166.61804	-1.119015	0.1771614	-6.316361	2.68E-10	1.74E-09
INSL5	2.0827653	-1.167418	0.1849192	-6.313124	2.73E-10	1.77E-09
PTHLH	2895.6618	-1.248094	0.1977259	-6.312244	2.75E-10	1.78E-09
LINC01293	3.7007248	-1.124855	0.1782893	-6.309156	2.81E-10	1.82E-09
LINC01124	19.434965	-1.124357	0.1782891	-6.30637	2.86E-10	1.85E-09
NAV2-AS2	1.0072881	-1.382322	0.2192447	-6.304929	2.88E-10	1.86E-09
VN1R51P	4.7217717	-1.026018	0.1629185	-6.297737	3.02E-10	1.95E-09

ONECUT3	5.8837934	-1.134764	0.1802571	-6.295256	3.07E-10	1.98E-09
RSU1P2	1.5991105	-1.567765	0.2492221	-6.290633	3.16E-10	2.03E-09
IGKV1D-17	13.671592	-1.584161	0.2519488	-6.287631	3.22E-10	2.07E-09
LINC01531	5.743286	-1.19485	0.1900963	-6.285499	3.27E-10	2.10E-09
MT1A	106.38059	-1.043949	0.1663516	-6.275562	3.48E-10	2.23E-09
SOSTDC1	198.16262	1.1825927	0.1886476	6.2687918	3.64E-10	2.32E-09
MIR8083	1.8932366	-1.377637	0.2197918	-6.267919	3.66E-10	2.33E-09
ANKRD20A1C	1.0195946	-1.704401	0.2720777	-6.264391	3.74E-10	2.38E-09
TM4SF4	48.66056	-1.074815	0.1715776	-6.264306	3.74E-10	2.39E-09
RTP3	5.6829264	-1.706412	0.272551	-6.260893	3.83E-10	2.44E-09
SLC17A3	13795.604	1.0221717	0.1632889	6.2598951	3.85E-10	2.45E-09
HAND1	1.5277864	-1.813839	0.2898647	-6.257536	3.91E-10	2.49E-09
MYL10	1.4712207	-1.269361	0.2030577	-6.251235	4.07E-10	2.58E-09
COCH	65.882401	-1.040563	0.1665874	-6.24635	4.20E-10	2.66E-09
SERPINB3	2.096063	-2.194585	0.351471	-6.244	4.27E-10	2.70E-09
TFF1	11.993636	-1.759559	0.2820197	-6.239134	4.40E-10	2.78E-09
PRIMA1	2441.1379	-1.216839	0.1952196	-6.233182	4.57E-10	2.88E-09
IGKV3D-7	1.845849	-1.722173	0.2763245	-6.232429	4.59E-10	2.89E-09
SPATA3-AS1	2.554311	-1.02309	0.1641567	-6.232398	4.59E-10	2.89E-09
OR1J4	0.8050279	-1.807259	0.2902243	-6.22711	4.75E-10	2.98E-09
HMGN1P14	1.4190843	-1.722273	0.2766333	-6.225832	4.79E-10	3.00E-09
TREML4	5.5671231	-1.193957	0.1918321	-6.223968	4.85E-10	3.04E-09
IGHJ1	3.5583544	-1.732013	0.2783204	-6.22309	4.87E-10	3.05E-09
PASD1	1.1974951	-3.117349	0.5009519	-6.22285	4.88E-10	3.06E-09
DDX43P3	8.5616397	-1.184417	0.190407	-6.220452	4.96E-10	3.10E-09
SP8	6.8499759	-2.341622	0.37658	-6.218125	5.03E-10	3.15E-09
IGLV2-18	67.256442	-1.237135	0.198999	-6.216792	5.07E-10	3.17E-09
RAD1P2	2.7552071	-1.030187	0.1657878	-6.213892	5.17E-10	3.23E-09
DKFZP434AC	3.7817214	-1.100945	0.1771834	-6.213591	5.18E-10	3.23E-09
TH	1.8169338	-1.535888	0.2472649	-6.211506	5.25E-10	3.27E-09
PAX7	1.0142503	-1.840243	0.2964188	-6.208254	5.36E-10	3.33E-09
TRBV8-2	4.4916679	-1.424028	0.2294066	-6.20744	5.39E-10	3.35E-09
SPRR1A	1.3122411	-2.315431	0.373027	-6.207142	5.40E-10	3.36E-09
SIGLECL1	1.2588642	-1.449671	0.2337105	-6.202848	5.55E-10	3.44E-09
DSG3	5.2153024	-1.540253	0.2483221	-6.202642	5.55E-10	3.45E-09
ZNF648	4.7519543	-1.50357	0.2426496	-6.196466	5.77E-10	3.57E-09
CES1P1	5.0908729	-1.556738	0.2513726	-6.192952	5.90E-10	3.65E-09
NPIPB1P	3.8765423	-1.084762	0.1751888	-6.191961	5.94E-10	3.67E-09
NMRK2	37.496521	-1.854006	0.2994384	-6.19161	5.96E-10	3.68E-09
RNU7-186P	2.938631	-1.020436	0.1649871	-6.184944	6.21E-10	3.82E-09
UCA1	7.6449075	-1.48487	0.2404714	-6.174828	6.62E-10	4.06E-09
THRB-IT1	3.5254243	-1.219152	0.1976417	-6.168493	6.89E-10	4.22E-09
DEPDC1-AS1	1.0695107	-1.255939	0.2036737	-6.16643	6.98E-10	4.27E-09
SCEL	15.731822	-1.365286	0.2215261	-6.163092	7.13E-10	4.35E-09
CLEC4M	1.4705966	-1.151278	0.1868055	-6.162981	7.14E-10	4.36E-09
SCARNA5	12.722733	-1.108056	0.1800783	-6.153188	7.59E-10	4.62E-09
NKX6-1	4.1424534	-1.493868	0.2429947	-6.147739	7.86E-10	4.77E-09
KLK8	2.9554224	-1.669266	0.2716196	-6.145604	7.97E-10	4.83E-09
C1QL4	476.51489	1.1560761	0.1882145	6.1423325	8.13E-10	4.92E-09
IGHV1-14	3.9682172	-1.516449	0.2469375	-6.141025	8.20E-10	4.96E-09
LINC01502	2.6151421	-1.608143	0.2619188	-6.139854	8.26E-10	5.00E-09

IGLL1	4.4299521	-1.134845	0.1848367	-6.139718	8.27E-10	5.00E-09
KLF14	1.7312724	-1.551128	0.2527574	-6.136827	8.42E-10	5.09E-09
TRMT112P3	1.007238	-1.254743	0.2045189	-6.135097	8.51E-10	5.14E-09
CYP4F24P	4.8147699	-1.341464	0.2186536	-6.135113	8.51E-10	5.14E-09
LINC01170	1.6483498	-1.268776	0.2069234	-6.131623	8.70E-10	5.24E-09
CYP1D1P	6.59746	1.1442527	0.18671	6.1285032	8.87E-10	5.34E-09
CRHR1	20.179901	-1.471336	0.2404314	-6.119568	9.38E-10	5.63E-09
TUBB3	0.4963105	-1.79905	0.2940956	-6.117227	9.52E-10	5.70E-09
TMEM246-AS	53.03485	1.0274301	0.1680432	6.1140834	9.71E-10	5.81E-09
NEUROG3	2.7113145	-1.204292	0.1970587	-6.111336	9.88E-10	5.90E-09
LCE5A	1.1949615	-1.318193	0.2157162	-6.110773	9.91E-10	5.92E-09
MYF6	1.0789057	-1.556166	0.2548482	-6.106247	1.02E-09	6.08E-09
ASIC5	1.0226637	-2.042469	0.3351045	-6.09502	1.09E-09	6.49E-09
GSDMC	15.407064	-1.217138	0.1998108	-6.091453	1.12E-09	6.63E-09
IGLJ1	1.2916233	-2.118247	0.3478955	-6.088746	1.14E-09	6.73E-09
FAM83C	1.4281375	-1.368606	0.2247908	-6.088357	1.14E-09	6.75E-09
CHP2	3.5606352	-1.218031	0.2001049	-6.086963	1.15E-09	6.80E-09
SP7	3.102185	-1.15931	0.190473	-6.086482	1.15E-09	6.82E-09
RN7SL180P	3.7270355	-1.1454	0.1882927	-6.083086	1.18E-09	6.96E-09
MAGEL2	18.301995	-1.184885	0.1949797	-6.076968	1.22E-09	7.21E-09
FIBCD1	210.39876	-1.247638	0.2053585	-6.075416	1.24E-09	7.27E-09
IGKV1OR10-	1.6001776	-2.074935	0.3418114	-6.07041	1.28E-09	7.49E-09
IFNWP19	10.977221	-1.322611	0.2181133	-6.06387	1.33E-09	7.79E-09
PRSS3P2	24.792868	-1.260246	0.207913	-6.061411	1.35E-09	7.91E-09
LINC01250	1.3395448	-1.233491	0.2037069	-6.055222	1.40E-09	8.20E-09
IGHV4-61	79.970006	-1.316318	0.2174059	-6.054658	1.41E-09	8.23E-09
SCRT2	0.6268788	-1.308701	0.216249	-6.051824	1.43E-09	8.37E-09
LRFN5	35.931419	-1.082529	0.1789692	-6.048692	1.46E-09	8.53E-09
F11-AS1	14.922522	1.087662	0.179932	6.0448489	1.50E-09	8.72E-09
PPP1R14D	436.37562	-1.166405	0.1931022	-6.040351	1.54E-09	8.96E-09
CEACAM20	4.0268633	-1.162853	0.1925171	-6.040258	1.54E-09	8.96E-09
BFSP2	9.7418009	-1.046357	0.1734485	-6.03267	1.61E-09	9.37E-09
BTG4	1.3008489	-1.365958	0.2266992	-6.025421	1.69E-09	9.77E-09
KRT23	19.653407	-1.079978	0.179302	-6.023236	1.71E-09	9.89E-09
RNU6ATAC27	1.4757072	-1.055592	0.1752982	-6.021696	1.73E-09	9.98E-09
KERA	3.7713414	-1.495553	0.248562	-6.01682	1.78E-09	1.03E-08
DMRTA2	1.6040314	-1.132248	0.1883543	-6.011265	1.84E-09	1.06E-08
LINC01444	1.4033529	-2.119085	0.3525981	-6.009915	1.86E-09	1.07E-08
IGLV2-28	8.2214341	-1.425197	0.2372178	-6.007969	1.88E-09	1.08E-08
RNU4-90P	1.5799359	-1.552737	0.258689	-6.002333	1.95E-09	1.12E-08
CRNN	2.9783803	-1.424855	0.2374125	-6.0016	1.95E-09	1.12E-08
SLC22A6	1833.9852	1.3423994	0.2237201	6.0003515	1.97E-09	1.13E-08
LINC01037	1.771535	-1.20583	0.2011501	-5.994675	2.04E-09	1.17E-08
LGI1	4.4284374	-1.194086	0.1992402	-5.993198	2.06E-09	1.18E-08
RNU6-1300F	0.6029572	-1.790951	0.2989922	-5.989959	2.10E-09	1.20E-08
GRM7-AS3	5.7898613	1.4609589	0.2440828	5.9855061	2.16E-09	1.23E-08
CASC19	2.5499555	-1.060973	0.1772833	-5.984619	2.17E-09	1.24E-08
MAGEA10	2.5026942	-1.347539	0.2253126	-5.980752	2.22E-09	1.26E-08
LMO7DN	1.6111838	-1.156504	0.1933897	-5.980174	2.23E-09	1.27E-08
OR3A3	0.902779	-2.317464	0.3878349	-5.975388	2.30E-09	1.30E-08
ITIH4-AS1	1.2217617	-1.170082	0.1960197	-5.969207	2.38E-09	1.35E-08

SLC15A5	2.8228714	-1.14107	0.1915492	-5.95706	2.57E-09	1.45E-08
RNU6-37P	2.4887827	-1.006225	0.1691204	-5.949753	2.69E-09	1.51E-08
IGKV2-18	1.4185557	-1.894919	0.3187243	-5.945322	2.76E-09	1.55E-08
ANKRD60	0.7252118	-2.570574	0.432519	-5.943262	2.79E-09	1.57E-08
CHIT1	968.99881	-1.238546	0.2084641	-5.941293	2.83E-09	1.59E-08
GOLGA8G	1.1141445	-1.264714	0.2130328	-5.936711	2.91E-09	1.63E-08
PITX3	1.5946721	-1.361055	0.2293049	-5.935567	2.93E-09	1.64E-08
KRT6B	7.8402977	-1.844944	0.311269	-5.927167	3.08E-09	1.72E-08
MIR8071-1	1.6428259	-1.496469	0.252705	-5.9218	3.18E-09	1.77E-08
CELF5	82.442811	-1.163948	0.1965525	-5.921816	3.18E-09	1.77E-08
RPS14P4	2.0511519	-1.005953	0.1699254	-5.919964	3.22E-09	1.79E-08
CDH18	2.6869538	-1.480195	0.250224	-5.915481	3.31E-09	1.84E-08
SPRR2A	1.9436134	-2.056376	0.3476778	-5.914602	3.33E-09	1.84E-08
TCL6	157.36849	1.1953766	0.2024546	5.9044176	3.54E-09	1.96E-08
MIR4537	1.4198448	-1.632557	0.2767169	-5.899735	3.64E-09	2.01E-08
KRT4	8.3771276	-1.614174	0.2736638	-5.898385	3.67E-09	2.03E-08
LINC00698	1.1718027	-1.454458	0.2467619	-5.894173	3.77E-09	2.08E-08
PRAMENP	6.1678274	-1.447572	0.2458511	-5.888003	3.91E-09	2.15E-08
MTUS2-AS1	2.5937402	-1.280968	0.217578	-5.887396	3.92E-09	2.16E-08
MLN	1.1039236	-1.205017	0.204717	-5.886257	3.95E-09	2.17E-08
NRSN1	5.3334996	-1.197527	0.2034559	-5.885928	3.96E-09	2.18E-08
GAPDHP37	2.7322643	-1.104651	0.1880362	-5.874673	4.24E-09	2.32E-08
VCX3B	2.3139274	-1.176783	0.2003796	-5.872766	4.29E-09	2.35E-08
LINC01397	2.291794	-1.100959	0.1875619	-5.869843	4.36E-09	2.39E-08
KLRF2	4.7843607	-1.189849	0.2028058	-5.86694	4.44E-09	2.43E-08
LINC01179	13.503686	1.283538	0.2187863	5.8666286	4.45E-09	2.43E-08
AFM	62.663303	1.1531481	0.196658	5.8637246	4.53E-09	2.47E-08
LINC01208	1.2699361	-1.400246	0.2388638	-5.862109	4.57E-09	2.49E-08
WFIKKN2	4.3759464	-1.122225	0.1915428	-5.858873	4.66E-09	2.54E-08
FOXG1	6.0236012	-1.132315	0.1932677	-5.85879	4.66E-09	2.54E-08
FOXA1	41.914062	-1.344053	0.2294813	-5.85692	4.72E-09	2.57E-08
IGHV3-37	1.5257146	-1.698187	0.2899901	-5.856018	4.74E-09	2.58E-08
KRT19P1	4.1162042	-1.312637	0.2242956	-5.852262	4.85E-09	2.64E-08
RNU7-77P	0.7363347	-1.279543	0.2186863	-5.851044	4.88E-09	2.65E-08
MS4A15	3.3025032	-1.10255	0.1884421	-5.850869	4.89E-09	2.66E-08
LINC01168	2.0349467	-1.33833	0.2289013	-5.846754	5.01E-09	2.72E-08
IGLV3-29	2.445017	-1.359079	0.2326294	-5.842251	5.15E-09	2.79E-08
MRGPRX3	0.749952	-1.592489	0.2727183	-5.839319	5.24E-09	2.83E-08
MROH3P	13.540249	-1.023927	0.1754033	-5.83756	5.30E-09	2.86E-08
MRAP	1.7270224	-1.071017	0.1835904	-5.833728	5.42E-09	2.92E-08
PAX3	1.2081254	-1.572731	0.2696406	-5.832695	5.45E-09	2.94E-08
IGKV1OR2-9	1.0396535	-2.279457	0.3911041	-5.82826	5.60E-09	3.01E-08
SDR9C7	1.8672153	-1.078987	0.185276	-5.823674	5.76E-09	3.10E-08
TRAF6P1	2.4270883	1.0877117	0.1868107	5.8225335	5.80E-09	3.11E-08
SCGB2A1	15.977601	-1.229334	0.2113888	-5.81551	6.04E-09	3.24E-08
NLGN4Y-AS1	2.4858547	-1.358048	0.2336013	-5.813527	6.12E-09	3.28E-08
GSG1L	2.5132549	-1.065413	0.1832929	-5.812625	6.15E-09	3.29E-08
FMN2	16.364816	-1.016866	0.1749901	-5.810988	6.21E-09	3.32E-08
ONECUT1	3.091803	-1.233189	0.212286	-5.809092	6.28E-09	3.36E-08
ARHGEF9-IT	0.8433791	-1.254222	0.2160692	-5.804723	6.45E-09	3.44E-08
ERVV-1	31.790598	-1.310592	0.2257892	-5.804493	6.46E-09	3.45E-08

RPSAP51	0.9468931	-1.270605	0.2189285	-5.803745	6.49E-09	3.46E-08
SLITRK3	1.7332955	-1.515436	0.2611167	-5.803675	6.49E-09	3.46E-08
IMPDH1P11	0.7146389	-1.590772	0.2743958	-5.797364	6.74E-09	3.59E-08
C1orf94	0.7844816	-1.758072	0.3033585	-5.79536	6.82E-09	3.63E-08
RNU6-501P	0.5329815	-1.409652	0.2432574	-5.7949	6.84E-09	3.64E-08
TEX13B	0.9787662	-1.229186	0.2124762	-5.785055	7.25E-09	3.84E-08
CNGA3	1.6782861	-1.346971	0.2328538	-5.784623	7.27E-09	3.85E-08
CXCL6	347.88162	-1.240476	0.2144595	-5.784199	7.29E-09	3.86E-08
KCNB2	1.7432783	-1.550368	0.2680884	-5.783049	7.34E-09	3.89E-08
SLC6A14	2.3249084	-1.529619	0.2646479	-5.779828	7.48E-09	3.96E-08
CLIC6	1065.742	-1.154473	0.1997812	-5.778688	7.53E-09	3.98E-08
IGHE	10.548805	-1.353464	0.2348136	-5.763994	8.21E-09	4.32E-08
IGHV3OR16-	2.3509324	-1.609213	0.2791902	-5.763858	8.22E-09	4.33E-08
MAGEC2	3.0469651	-3.073164	0.5332544	-5.763034	8.26E-09	4.35E-08
MMP3	10.339579	-1.296611	0.2250257	-5.762057	8.31E-09	4.37E-08
PHEX-AS1	4.1596166	-1.080329	0.1875234	-5.761035	8.36E-09	4.40E-08
RPL32P33	1.1928103	-1.170757	0.2032705	-5.7596	8.43E-09	4.43E-08
ANKRD62	1.6682939	-1.087199	0.1888176	-5.75793	8.52E-09	4.47E-08
RIMS2	60.242224	-1.259219	0.2189039	-5.75238	8.80E-09	4.61E-08
SPRR1B	1.9713594	-2.288392	0.3978397	-5.752047	8.82E-09	4.62E-08
OGN	282.14079	-1.162433	0.2022844	-5.746528	9.11E-09	4.77E-08
NYAP2	2.8733457	-1.080291	0.1880505	-5.744687	9.21E-09	4.81E-08
MYO16-AS1	1.3028161	-1.555478	0.2708424	-5.74311	9.30E-09	4.85E-08
ASNSP1	1.0072134	-1.559391	0.2724395	-5.723805	1.04E-08	5.41E-08
HYAL4	3.2804388	-1.140328	0.1992836	-5.722138	1.05E-08	5.46E-08
INSRR	22.164242	-1.112629	0.1944998	-5.720461	1.06E-08	5.51E-08
GGT6	109.13712	1.2429056	0.2175178	5.7140417	1.10E-08	5.71E-08
RAB5CP1	1.7195286	-1.128824	0.1977377	-5.708693	1.14E-08	5.88E-08
CT45A10	1.2370588	-2.979897	0.522135	-5.707138	1.15E-08	5.93E-08
OR2AT1P	8.6185949	-1.76711	0.3099796	-5.70073	1.19E-08	6.14E-08
CALML3	9.6619971	-1.199554	0.2104775	-5.699202	1.20E-08	6.19E-08
RNU6-942P	1.0814498	-1.116418	0.195905	-5.698776	1.21E-08	6.21E-08
BMS1P17	0.4620208	-1.528843	0.2685093	-5.693817	1.24E-08	6.38E-08
KRT8P50	1.6842686	-1.003865	0.1764172	-5.690287	1.27E-08	6.51E-08
LHX5	0.9108723	-1.68725	0.2966371	-5.687927	1.29E-08	6.59E-08
IGHV7-56	3.1100397	-1.6022	0.282073	-5.68009	1.35E-08	6.89E-08
LINC01524	1.7764105	-1.224058	0.2155937	-5.677614	1.37E-08	6.98E-08
GAL	4.4403925	-1.179297	0.2077261	-5.677173	1.37E-08	7.00E-08
RPRML	1.1982452	-1.149851	0.2026026	-5.675401	1.38E-08	7.06E-08
CYCSP6	0.8192794	-1.795118	0.3164481	-5.672708	1.41E-08	7.17E-08
OR4C6	1.2243925	-2.388551	0.4211723	-5.671197	1.42E-08	7.23E-08
FEZF1	1.0552478	-1.479905	0.2610247	-5.669595	1.43E-08	7.29E-08
GLRA3	4.4338054	-1.22216	0.2156462	-5.667431	1.45E-08	7.38E-08
SPOCK3	5.861935	1.176163	0.2075422	5.6671039	1.45E-08	7.39E-08
PKP1	616.3264	-1.082308	0.1910395	-5.665365	1.47E-08	7.46E-08
PGLYRP2	22.618343	-1.134781	0.2003348	-5.664422	1.48E-08	7.49E-08
C10orf53	0.8821752	-1.924901	0.3403008	-5.656468	1.55E-08	7.83E-08
GRIN2B	67.874148	-1.01269	0.1791075	-5.654089	1.57E-08	7.92E-08
NPAP1	6.1792921	-1.208183	0.2138123	-5.650673	1.60E-08	8.07E-08
RSPO2	2.6923111	-1.467366	0.259686	-5.650537	1.60E-08	8.08E-08
GATA4	7.1927417	-1.532362	0.2713033	-5.648153	1.62E-08	8.18E-08

FGF23	2.4205041	-1.477488	0.2616179	-5.647502	1.63E-08	8.21E-08
ZBED1P1	2.9472931	-1.017862	0.180263	-5.64654	1.64E-08	8.25E-08
MTND6P21	0.9641682	-1.332422	0.2360472	-5.644725	1.65E-08	8.33E-08
UGT1A13P	0.6710744	-1.66883	0.2958101	-5.641558	1.69E-08	8.47E-08
GDF10	23.535202	-1.008022	0.1789376	-5.633372	1.77E-08	8.86E-08
DUX4L9	1.1404206	-1.620662	0.2878099	-5.631014	1.79E-08	8.97E-08
LEFTY2	16.962789	-1.02013	0.1811716	-5.630741	1.79E-08	8.99E-08
FOXD3-AS1	1.6181477	-1.3745	0.2441623	-5.629455	1.81E-08	9.05E-08
THRSP	42.299213	-1.2668	0.2251359	-5.626822	1.84E-08	9.18E-08
CSAG3	0.8420947	-1.881274	0.3349976	-5.615785	1.96E-08	9.75E-08
BANF1P1	1.0028728	-1.116327	0.1988341	-5.614364	1.97E-08	9.83E-08
CRYBA2	0.776105	-1.947443	0.3470762	-5.610995	2.01E-08	1.00E-07
RNA5SP245	1.2190938	-1.052859	0.1876738	-5.610047	2.02E-08	1.01E-07
EFNA2	7.7972368	-1.214576	0.2165847	-5.607858	2.05E-08	1.02E-07
IGKV5-2	29.74841	-1.265263	0.225708	-5.60575	2.07E-08	1.03E-07
EPPIN	1.3067621	-1.530102	0.273027	-5.604215	2.09E-08	1.04E-07
IGHV3-42	1.7773695	-1.516599	0.2706833	-5.602855	2.11E-08	1.05E-07
LINC01559	482.13562	-1.45233	0.2592483	-5.60208	2.12E-08	1.05E-07
SLC6A5	1.4026493	-1.650875	0.2948786	-5.598489	2.16E-08	1.07E-07
LRIT2	9.5669164	-1.221626	0.2182684	-5.596897	2.18E-08	1.08E-07
CHRNA4	18.251254	1.4327322	0.2561272	5.5938318	2.22E-08	1.10E-07
MLIP-IT1	7.2063025	-1.034357	0.184983	-5.591631	2.25E-08	1.11E-07
SLC22A13	92.028851	1.0345194	0.1852825	5.583471	2.36E-08	1.16E-07
ANKRD30B	5.9063344	-1.39234	0.2494432	-5.581792	2.38E-08	1.17E-07
TBC1D3E	2.9893014	-1.467781	0.2634155	-5.572115	2.52E-08	1.23E-07
EREG	31.716031	-1.012109	0.1816481	-5.571811	2.52E-08	1.24E-07
IGLJ3	0.8747543	-1.848862	0.3318882	-5.570736	2.54E-08	1.24E-07
MROH2A	103.48593	-1.102331	0.1981153	-5.564088	2.64E-08	1.29E-07
IGKV1-27	252.01084	-1.077825	0.1937997	-5.561543	2.67E-08	1.31E-07
IGKV2-4	1.2232612	-1.352001	0.2432026	-5.559157	2.71E-08	1.32E-07
ASPG	1019.7853	1.0169556	0.1830276	5.5562971	2.76E-08	1.34E-07
CTNNA2	9.8038629	-1.29561	0.2331894	-5.556043	2.76E-08	1.35E-07
ATP4A	2.8124285	-1.269165	0.228635	-5.551054	2.84E-08	1.38E-07
RPL6P7	2.061882	-1.062719	0.1914665	-5.550417	2.85E-08	1.39E-07
WFDC11	0.5188771	-1.920554	0.3463144	-5.545695	2.93E-08	1.42E-07
ASB4	31.258869	-1.043783	0.1884782	-5.537951	3.06E-08	1.48E-07
SLC12A1	555.14216	1.3219162	0.2389341	5.5325547	3.16E-08	1.53E-07
GAGE10	1.050705	-1.170227	0.2118682	-5.523375	3.33E-08	1.60E-07
IGLCOR22-1	1.0347442	-1.457552	0.2640417	-5.520158	3.39E-08	1.63E-07
ALDH3B2	7.9443292	-1.211723	0.2195195	-5.519888	3.39E-08	1.63E-07
FETUB	4.4075492	-1.791535	0.3246781	-5.51788	3.43E-08	1.65E-07
PSAT1P3	2.1451412	-1.073345	0.1945748	-5.516362	3.46E-08	1.66E-07
CYP4F2	56.600949	-1.27185	0.2308626	-5.509121	3.61E-08	1.73E-07
UPK1A-AS1	1.9192965	-1.22503	0.2223833	-5.508643	3.62E-08	1.73E-07
SERPINA3	7.4203438	-1.183677	0.2149425	-5.506949	3.65E-08	1.75E-07
PWRN1	12.098894	-1.23476	0.2242867	-5.505274	3.69E-08	1.76E-07
PSMD10P2	2.0336894	-1.057309	0.192218	-5.500573	3.79E-08	1.81E-07
SLC7A14	3.3411082	-1.027058	0.1871919	-5.486658	4.10E-08	1.95E-07
RNASE9	0.6018227	-1.359523	0.2479605	-5.482821	4.19E-08	1.99E-07
ABCA13	19.436637	-1.021042	0.1863816	-5.478237	4.30E-08	2.03E-07
IGKV1OR9-2	0.8599713	-2.152682	0.3930371	-5.477046	4.32E-08	2.05E-07

ITLN2	1.447493	-1.294748	0.2365884	-5.472577	4.44E-08	2.10E-07
LINC01226	2.1352302	-1.378205	0.2520379	-5.468243	4.55E-08	2.15E-07
IGHV1OR15-	16.613253	-1.196874	0.2192952	-5.457821	4.82E-08	2.27E-07
LINC01429	1.3939362	-1.352471	0.2478791	-5.456175	4.87E-08	2.29E-07
LINC01048	0.7554346	-1.418622	0.2601685	-5.452704	4.96E-08	2.33E-07
IGHV3-16	1.1883067	-1.651795	0.3035988	-5.440717	5.31E-08	2.48E-07
IGLV1-62	0.9697503	-1.544529	0.2842483	-5.433733	5.52E-08	2.57E-07
CDHR1	1734.6169	-1.010175	0.1859346	-5.432956	5.54E-08	2.58E-07
SNTG1	3.5863868	-1.424463	0.2626444	-5.42354	5.84E-08	2.72E-07
RNU6-1321F	0.642556	-1.210306	0.2232153	-5.422144	5.89E-08	2.74E-07
RNA5SP40	4.081965	-1.296868	0.2393945	-5.417287	6.05E-08	2.80E-07
RASA3-IT1	0.8349709	-1.045243	0.1929625	-5.41682	6.07E-08	2.81E-07
RN7SL316P	0.7361829	-1.191482	0.2200081	-5.415625	6.11E-08	2.83E-07
RNU6ATAC38	0.7213706	-1.36423	0.2522833	-5.407532	6.39E-08	2.95E-07
FOXL2NB	2.6226501	-1.171072	0.2168921	-5.399331	6.69E-08	3.08E-07
GDNF-AS1	3.9430293	-1.119785	0.2076441	-5.392807	6.94E-08	3.19E-07
REG1A	3424.1989	-1.187886	0.220302	-5.392077	6.96E-08	3.20E-07
BPIFA2	4.2340352	-1.28102	0.2377005	-5.389217	7.08E-08	3.25E-07
CSMD3	3.880788	-1.21587	0.2257439	-5.386056	7.20E-08	3.30E-07
OR2A14	0.690952	-1.153171	0.2142225	-5.383051	7.32E-08	3.35E-07
CALML5	1.2085207	-2.204092	0.4104335	-5.370156	7.87E-08	3.59E-07
LUZP2	19.54233	1.0553346	0.1965355	5.3696883	7.89E-08	3.60E-07
LINC00906	28.672016	-1.047915	0.1951532	-5.369704	7.89E-08	3.60E-07
RNU6-446P	0.5137469	-1.427072	0.2657965	-5.36904	7.92E-08	3.61E-07
RBPJL	3.8781952	-1.033632	0.1926083	-5.366497	8.03E-08	3.66E-07
SPRR2D	1.1797963	-2.418176	0.4510218	-5.36155	8.25E-08	3.76E-07
RNU6-353P	1.4712787	-1.07936	0.2013382	-5.360928	8.28E-08	3.77E-07
IGHD3-9	0.8011509	-1.587139	0.2962065	-5.358218	8.40E-08	3.82E-07
ITIH1	44.356313	-1.15651	0.2158763	-5.35728	8.45E-08	3.84E-07
CLIC4P2	0.9822111	-1.260008	0.2362008	-5.334478	9.58E-08	4.32E-07
IGLC5	0.6318625	-1.766506	0.3311875	-5.333855	9.61E-08	4.33E-07
CFHR4	1.5156568	-1.401788	0.2628284	-5.333473	9.64E-08	4.34E-07
P2RX2	1.4487649	-1.167877	0.2190078	-5.332583	9.68E-08	4.36E-07
SAGE1	4.3039716	-1.168984	0.219449	-5.326908	9.99E-08	4.49E-07
TSPAN8	226.93494	-1.094864	0.2058303	-5.319257	1.04E-07	4.67E-07
SNX18P25	0.6161197	-1.601495	0.3015287	-5.311251	1.09E-07	4.86E-07
SCARA5	46.44431	-1.041875	0.1961726	-5.311012	1.09E-07	4.87E-07
DBX2	1.1800082	-1.090336	0.2054084	-5.30814	1.11E-07	4.94E-07
IGKV1OR22-	2.8903664	-1.491022	0.2809098	-5.307832	1.11E-07	4.95E-07
ANKRD34C	3.6236272	-1.094453	0.2062886	-5.305446	1.12E-07	5.01E-07
LINC01281	4.9382419	-1.035218	0.1951455	-5.304852	1.13E-07	5.02E-07
GUCA1C	1.6823309	-1.593296	0.3003848	-5.304185	1.13E-07	5.04E-07
RNU6-1263F	0.8750629	-1.209229	0.2282114	-5.298724	1.17E-07	5.18E-07
C1orf87	2.6193558	-1.231566	0.2324941	-5.297192	1.18E-07	5.22E-07
TAGLN3	15.275986	-1.033067	0.195316	-5.289211	1.23E-07	5.44E-07
ARX	64.639831	-1.557502	0.2945156	-5.288351	1.23E-07	5.47E-07
HOXD12	1.1385891	-1.187859	0.2248169	-5.283676	1.27E-07	5.60E-07
IGHV3OR16-	2.2887699	-1.211532	0.2293921	-5.281488	1.28E-07	5.67E-07
ELSPBP1	3.1673031	-1.670044	0.3162726	-5.280392	1.29E-07	5.70E-07
IGKV2OR22-	2.4671288	-1.358975	0.2575042	-5.277488	1.31E-07	5.78E-07
RNU6-53P	0.8188349	-1.036671	0.1964409	-5.277267	1.31E-07	5.78E-07

RN7SL672P	1.3964799	-1.158879	0.2200314	-5.266879	1.39E-07	6.10E-07
DNMT3L	2.302357	-1.083001	0.2060585	-5.255793	1.47E-07	6.45E-07
SPRR3	3.4068401	-1.714737	0.3268167	-5.246787	1.55E-07	6.76E-07
ADARB2-AS1	0.7387997	-1.76829	0.3373254	-5.242091	1.59E-07	6.92E-07
DPRXP2	1.608708	-1.002394	0.1912248	-5.241964	1.59E-07	6.93E-07
WFDC8	0.7633749	-1.24078	0.2367539	-5.2408	1.60E-07	6.97E-07
LINC01388	1.1936806	1.0523899	0.2008112	5.240693	1.60E-07	6.97E-07
PTPRQ	5.9458197	-1.056325	0.2019912	-5.229558	1.70E-07	7.37E-07
OR2T10	54.67959	1.0274422	0.1966369	5.225074	1.74E-07	7.54E-07
LBX1	0.6848821	-1.673844	0.3204207	-5.223894	1.75E-07	7.59E-07
MDC1-AS1	0.702906	-1.025762	0.1963917	-5.223041	1.76E-07	7.62E-07
IGLV5-45	77.408049	-1.011017	0.1935794	-5.22275	1.76E-07	7.63E-07
RNA5SP25	1.3610962	-1.059725	0.2029378	-5.221922	1.77E-07	7.66E-07
NPM1P47	0.6060137	-1.192879	0.2287848	-5.21398	1.85E-07	7.98E-07
GUCA1A	1.615813	-1.247038	0.2393156	-5.210853	1.88E-07	8.11E-07
ISM2	48.722621	-1.066372	0.2047886	-5.207183	1.92E-07	8.26E-07
FAXC	65.538722	-1.031593	0.1981912	-5.205042	1.94E-07	8.35E-07
KRT40	1.3288903	-1.037782	0.1994564	-5.203049	1.96E-07	8.43E-07
GOLGA8F	0.8761228	-1.054781	0.2027459	-5.202477	1.97E-07	8.46E-07
TPRG1-AS2	0.7482254	-1.157693	0.2226466	-5.199687	2.00E-07	8.58E-07
IGKV1OR-3	0.9270168	-1.740192	0.3347513	-5.198462	2.01E-07	8.63E-07
IGKV1OR2-1	2.118036	-1.829516	0.3519915	-5.197614	2.02E-07	8.67E-07
SNORA70B	1.5132345	-1.095593	0.2111147	-5.189562	2.11E-07	9.03E-07
CNGB1	9.692555	-1.007178	0.1943636	-5.181927	2.20E-07	9.38E-07
MIR6775	0.7129935	-1.318509	0.2545876	-5.179	2.23E-07	9.52E-07
GRID1-AS1	1.7153897	-1.026072	0.1981367	-5.178608	2.24E-07	9.54E-07
CALCA	13.011898	1.498915	0.2895601	5.1765246	2.26E-07	9.64E-07
IGHD3-3	1.260609	-1.618819	0.3127621	-5.175878	2.27E-07	9.67E-07
LINC00276	2.1576557	-1.487014	0.2873651	-5.174649	2.28E-07	9.73E-07
IL20RA	25.538482	-1.089175	0.2105512	-5.172968	2.30E-07	9.81E-07
SNRPCP19	0.7949072	-1.043719	0.2018258	-5.171388	2.32E-07	9.89E-07
IGHD2-2	0.8069618	-2.170511	0.4197928	-5.170433	2.34E-07	9.93E-07
TECTB	1.0614608	-1.585331	0.3067605	-5.167977	2.37E-07	1.01E-06
LRRC74B	3.1601587	-1.108928	0.214604	-5.167323	2.37E-07	1.01E-06
KRTAP5-6	1.1033247	-1.243244	0.2407004	-5.165111	2.40E-07	1.02E-06
RNU6-951P	0.6310919	-1.230984	0.238343	-5.164755	2.41E-07	1.02E-06
RN7SKP106	0.4452924	-1.263379	0.2446853	-5.163281	2.43E-07	1.03E-06
TRERNA1	0.5247643	-1.180082	0.2285715	-5.162854	2.43E-07	1.03E-06
MYL7	0.7311288	-1.117228	0.2164988	-5.160432	2.46E-07	1.04E-06
RPL23AP50	1.0325284	-1.058276	0.2051406	-5.158784	2.49E-07	1.05E-06
KRT24	1.4180963	-1.247474	0.2421318	-5.152043	2.58E-07	1.09E-06
MIR766	0.5413589	-1.339889	0.2604625	-5.144267	2.69E-07	1.13E-06
NTNG1	38.347456	-1.110834	0.2159794	-5.143241	2.70E-07	1.14E-06
LDLRAD1	1.3192766	-1.513441	0.2945918	-5.137416	2.79E-07	1.17E-06
RN7SKP241	0.4766707	-1.300761	0.2532379	-5.136517	2.80E-07	1.18E-06
ELMOD1	89.038378	1.071291	0.2086426	5.134574	2.83E-07	1.19E-06
MIR6784	0.7734387	-1.09235	0.2127856	-5.133569	2.84E-07	1.19E-06
IRX6	286.75521	-1.115499	0.2175503	-5.127545	2.94E-07	1.23E-06
PRSS22	20.035703	-1.166707	0.2276837	-5.124246	2.99E-07	1.25E-06
SPACA3	1.9839342	-1.131319	0.2209423	-5.120428	3.05E-07	1.27E-06
CYP24A1	608.43882	-1.165716	0.2277898	-5.117507	3.10E-07	1.29E-06

IGHV3-75	1.8934231	-1.342628	0.2624918	-5.114933	3.14E-07	1.31E-06
HS3ST4	1.3590401	-1.360938	0.2663295	-5.109978	3.22E-07	1.34E-06
RNU1-72P	0.7125904	-1.402934	0.2746958	-5.107229	3.27E-07	1.36E-06
RNU6-44P	4.0127279	1.1230979	0.2199404	5.1063737	3.28E-07	1.37E-06
RBAKDN	1.501054	-1.018432	0.1994756	-5.105548	3.30E-07	1.37E-06
LINC01213	3.3904011	-1.079716	0.2114846	-5.105413	3.30E-07	1.37E-06
MED28P8	0.688873	-1.828395	0.3585769	-5.099032	3.41E-07	1.42E-06
ANKRD30BP2	0.8617103	-1.848261	0.3628401	-5.093872	3.51E-07	1.45E-06
RNU6-437P	0.7790766	-1.154254	0.2268338	-5.088546	3.61E-07	1.49E-06
CCDC33	1.8547127	-1.4531	0.285563	-5.088544	3.61E-07	1.49E-06
SLC6A21P	1.610174	-1.263067	0.248348	-5.085875	3.66E-07	1.51E-06
CYP11A1	81.679356	-1.3123	0.2584463	-5.07765	3.82E-07	1.58E-06
ASS1P7	0.9389229	-1.192467	0.2351758	-5.070533	3.97E-07	1.63E-06
PPIAP32	0.5864795	-1.063266	0.2100182	-5.062731	4.13E-07	1.70E-06
MIR4671	0.5720714	-1.298222	0.2564278	-5.062719	4.13E-07	1.70E-06
HNRNPKP5	0.9789662	-1.093672	0.2160957	-5.061055	4.17E-07	1.71E-06
TUBA3C	5.6529426	-1.334261	0.263719	-5.059403	4.21E-07	1.73E-06
MKX-AS1	0.6715982	-1.64699	0.3257255	-5.056375	4.27E-07	1.75E-06
FGG	4827.0449	-1.546035	0.3057725	-5.056161	4.28E-07	1.75E-06
SPANXN3	0.6209998	-1.9722	0.3904274	-5.051389	4.39E-07	1.79E-06
LINC00678	1.1855166	1.6949362	0.3356311	5.0499981	4.42E-07	1.81E-06
KRT82	1.2437878	-1.372856	0.2720108	-5.047063	4.49E-07	1.83E-06
RNU1-56P	1.1245536	-1.037457	0.2055931	-5.046166	4.51E-07	1.84E-06
SLC25A24P1	2.1477496	-1.140413	0.2260542	-5.044866	4.54E-07	1.85E-06
KRTAP1-1	0.8423049	-1.95283	0.3872872	-5.04233	4.60E-07	1.87E-06
MIR581	1.6098136	-1.102524	0.2186744	-5.041852	4.61E-07	1.88E-06
NDUFAF4P2	0.8508895	-1.020326	0.2025197	-5.038158	4.70E-07	1.91E-06
IGHV3-54	0.6314495	-1.608026	0.3203531	-5.019541	5.18E-07	2.10E-06
ZNF536	9.7230792	-1.010893	0.2018755	-5.007507	5.51E-07	2.23E-06
DYDC2	10.681388	-1.136926	0.2271551	-5.005066	5.58E-07	2.25E-06
UTS2R	1.4785895	-1.283793	0.2566779	-5.001572	5.69E-07	2.29E-06
DCD	1.7051925	-1.450309	0.2902705	-4.996407	5.84E-07	2.35E-06
C14orf39	1.8040842	-1.087167	0.2175973	-4.996235	5.85E-07	2.35E-06
IGKV2-29	22.986567	-1.416419	0.2835845	-4.9947	5.89E-07	2.37E-06
SPRR2E	1.1271398	-2.265292	0.4538329	-4.991467	5.99E-07	2.40E-06
KCNH1-IT1	0.7319137	-1.82303	0.3652831	-4.990731	6.02E-07	2.41E-06
YTHDF1P1	1.0983573	-1.158502	0.2322005	-4.98923	6.06E-07	2.43E-06
SLC36A2	60.033233	-1.062168	0.2129299	-4.988347	6.09E-07	2.44E-06
WFDC6	0.6531019	-1.668893	0.3347613	-4.985322	6.19E-07	2.48E-06
GIP	1.9122353	-1.555585	0.3123159	-4.980806	6.33E-07	2.53E-06
IGHV3OR16-	6.3121594	-1.091289	0.2194628	-4.972547	6.61E-07	2.64E-06
TEX26	1.0934697	-1.122031	0.2257679	-4.969845	6.70E-07	2.67E-06
THEGL	1.2709327	-1.236736	0.2491577	-4.963669	6.92E-07	2.75E-06
IGLV3-6	0.8560658	-1.681547	0.3388231	-4.962906	6.94E-07	2.76E-06
NPY4R	3.6948414	-1.002607	0.2020901	-4.961191	7.01E-07	2.78E-06
IGKV1-37	0.873066	-2.043319	0.4118671	-4.961111	7.01E-07	2.78E-06
DBX1	0.5948133	-1.664922	0.3356424	-4.960404	7.03E-07	2.79E-06
PGLYRP4	1.6565656	-1.096006	0.2210227	-4.958795	7.09E-07	2.81E-06
KLK11	4.8814613	-1.508762	0.3042616	-4.958766	7.09E-07	2.81E-06
ZSCAN10	1.960559	-1.023372	0.2067594	-4.949581	7.44E-07	2.94E-06
IGHV3OR16-	1.4909236	-1.515284	0.306678	-4.940962	7.77E-07	3.07E-06

SSTR5-AS1	7.7167098	-1.023564	0.2072838	-4.937984	7.89E-07	3.11E-06
LINC00407	0.5845699	-1.027617	0.2082374	-4.934832	8.02E-07	3.16E-06
RLN3	1.1249136	-1.141128	0.2315495	-4.928226	8.30E-07	3.26E-06
RNU6-1149F	0.6982598	-1.29906	0.2636453	-4.927301	8.34E-07	3.27E-06
LMX1B	23.264612	-1.137155	0.2307954	-4.927114	8.35E-07	3.28E-06
CYP1A2	4.073911	-1.303479	0.2647448	-4.923531	8.50E-07	3.33E-06
LRRC38	1.9807604	-1.365039	0.2775808	-4.917627	8.76E-07	3.43E-06
NKX2-3	14.32581	1.3651833	0.2779456	4.9116918	9.03E-07	3.53E-06
CD300LD	1.0635876	-1.151469	0.234636	-4.90747	9.23E-07	3.60E-06
AGR3	12.839191	-1.494542	0.3046562	-4.905669	9.31E-07	3.63E-06
SERPINB5	4.7302042	-1.134912	0.2314282	-4.903948	9.39E-07	3.66E-06
FAHD2P1	0.8303505	-1.427006	0.2911143	-4.901875	9.49E-07	3.69E-06
RNU6-1069F	0.8595571	-1.134245	0.2315337	-4.898834	9.64E-07	3.75E-06
SLC22A12	3121.0476	1.0222052	0.2087364	4.8971095	9.73E-07	3.78E-06
SHCBP1L	1.0226572	-1.156438	0.2362624	-4.894718	9.84E-07	3.82E-06
CKMT1B	32.067019	-1.286489	0.2630223	-4.891178	1.00E-06	3.89E-06
R3HDML	0.8238214	-1.549234	0.3170097	-4.887024	1.02E-06	3.96E-06
TRAV28	1.8135852	-1.538502	0.3148269	-4.886817	1.02E-06	3.97E-06
MIR6075	1.1428366	-1.000986	0.2049503	-4.884042	1.04E-06	4.02E-06
CYCSP28	0.7693959	-1.349725	0.2767201	-4.877583	1.07E-06	4.14E-06
BCL2L12P1	1.4470115	-1.021109	0.2097108	-4.869131	1.12E-06	4.31E-06
GCNT1P3	1.260884	-1.045692	0.2148069	-4.868057	1.13E-06	4.33E-06
OR7E28P	0.7745054	-2.222747	0.4566365	-4.867651	1.13E-06	4.34E-06
LINC00491	0.7457474	-1.818116	0.373698	-4.865201	1.14E-06	4.39E-06
MTND4P20	14.552752	-1.073861	0.2207288	-4.86507	1.14E-06	4.39E-06
RPS23P3	0.7064204	-1.859245	0.382572	-4.859858	1.17E-06	4.50E-06
PAUPAR	0.6141543	-1.992548	0.4103678	-4.855517	1.20E-06	4.60E-06
GFRA4	0.5518446	-1.841138	0.37923	-4.854938	1.20E-06	4.61E-06
KCNK16	0.7811991	-1.303907	0.2689671	-4.847829	1.25E-06	4.77E-06
RNU2-5P	1.0667177	-1.031107	0.2127679	-4.84616	1.26E-06	4.81E-06
TMEM196	18.408178	-1.310501	0.2704976	-4.844779	1.27E-06	4.84E-06
OR11H13P	0.5855027	-1.4217	0.2942663	-4.831339	1.36E-06	5.16E-06
IL36G	1.0147783	-1.049691	0.2173056	-4.830486	1.36E-06	5.18E-06
ZIC1	7.1474388	-1.222982	0.2532229	-4.829665	1.37E-06	5.20E-06
LINC01443	16.093723	-1.444994	0.2992291	-4.829055	1.37E-06	5.21E-06
SLC38A8	3.6524242	-1.494364	0.3097254	-4.824804	1.40E-06	5.32E-06
INSM1	1.8746932	-1.090707	0.2262318	-4.821194	1.43E-06	5.41E-06
DES	908.7157	-1.013385	0.2106456	-4.810854	1.50E-06	5.68E-06
IGHEP1	1.0801116	-1.702264	0.3541786	-4.806232	1.54E-06	5.80E-06
FUT9	21.135927	1.2879207	0.2679868	4.8059105	1.54E-06	5.81E-06
KRT223P	972.93239	-1.065255	0.2219897	-4.798669	1.60E-06	6.00E-06
KCNK4	0.7447359	-1.22708	0.2559241	-4.794702	1.63E-06	6.12E-06
KCNJ6-AS1	0.5997452	-1.862383	0.3885746	-4.792858	1.64E-06	6.17E-06
VN1R110P	0.8336716	-1.050658	0.21925	-4.792058	1.65E-06	6.19E-06
RNU6-665P	0.7741523	-1.005765	0.2105547	-4.776738	1.78E-06	6.65E-06
AKR1B10P1	1.7413937	-1.632199	0.3417864	-4.775494	1.79E-06	6.69E-06
DPPA2	1.9197774	-1.464663	0.3068544	-4.773155	1.81E-06	6.76E-06
NBPF6	0.9453302	-1.19934	0.2514604	-4.769498	1.85E-06	6.88E-06
SLC34A1	396.26589	1.0456821	0.219258	4.7691847	1.85E-06	6.89E-06
MAGEA4	1.6095208	-2.721857	0.5708553	-4.768033	1.86E-06	6.92E-06
MIR642A	0.4895209	-1.122591	0.2355564	-4.765698	1.88E-06	7.00E-06

KCNU1	1.6653046	-1.420656	0.298288	-4.762698	1.91E-06	7.09E-06
LINC01198	0.9262265	-1.109175	0.2330723	-4.758933	1.95E-06	7.22E-06
SCGB2A2	6.698927	-1.347212	0.2833669	-4.754302	1.99E-06	7.37E-06
BPIFB4	1.8509704	-1.40518	0.2955771	-4.754023	1.99E-06	7.38E-06
PNLIPRP2	2.0669588	-1.241801	0.2612202	-4.753846	2.00E-06	7.39E-06
RNU6-937P	0.5928011	-1.056885	0.2223614	-4.753007	2.00E-06	7.41E-06
IGFBPL1	3.3743349	-1.040526	0.2190652	-4.749847	2.04E-06	7.51E-06
LBX1-AS1	0.7749583	-1.727677	0.3639157	-4.747464	2.06E-06	7.60E-06
OTC	3.3206586	1.0735969	0.2262134	4.7459479	2.08E-06	7.65E-06
LIX1	362.81477	1.0406491	0.2195458	4.7400084	2.14E-06	7.87E-06
KRT3	0.5002229	-1.584963	0.3349708	-4.731644	2.23E-06	8.17E-06
APOC3	26.452239	-1.303203	0.2755925	-4.72873	2.26E-06	8.28E-06
TMEFF1	1.977226	-1.043356	0.2206571	-4.728403	2.26E-06	8.30E-06
SPATA16	0.5980019	-1.366876	0.2890896	-4.72821	2.27E-06	8.30E-06
RPL31P50	0.9016601	-1.345953	0.284708	-4.727487	2.27E-06	8.33E-06
IRGC	0.9260472	-1.187795	0.2512803	-4.726974	2.28E-06	8.35E-06
IGKV2D-18	0.7215664	-1.479326	0.3137063	-4.715641	2.41E-06	8.79E-06
C1QTNF8	0.9557238	-1.19359	0.2533338	-4.711529	2.46E-06	8.96E-06
KRT35	0.9109917	-1.479595	0.3149621	-4.697691	2.63E-06	9.54E-06
MIR205HG	12.818236	-1.367081	0.2917036	-4.686543	2.78E-06	1.00E-05
KRT17P7	0.7046665	-1.098125	0.2344527	-4.68378	2.82E-06	1.02E-05
KCNJ13	56.359082	-1.007833	0.2151991	-4.683259	2.82E-06	1.02E-05
KLK5	4.2149656	-1.407141	0.3006764	-4.67992	2.87E-06	1.03E-05
PVALB	301.21416	1.4004769	0.2997744	4.6717698	2.99E-06	1.07E-05
LINC01181	0.5239368	-1.201451	0.2572438	-4.670476	3.01E-06	1.08E-05
CALHM3	0.7376062	-1.05483	0.2263307	-4.66057	3.15E-06	1.13E-05
CEACAM7	1.4631425	-1.548609	0.3323783	-4.659174	3.17E-06	1.14E-05
UGT2B4	8.0055396	1.2379965	0.2659566	4.6548815	3.24E-06	1.16E-05
C14orf180	191.26817	1.0700518	0.2299322	4.6537714	3.26E-06	1.16E-05
MIR7-3HG	1.1013168	-1.022547	0.2199924	-4.648102	3.35E-06	1.19E-05
KLF2P1	1.1174421	-1.766433	0.3801354	-4.646853	3.37E-06	1.20E-05
TPSP2	4.3165954	-1.459807	0.3141902	-4.646253	3.38E-06	1.21E-05
RNU7-61P	1.1719325	-1.165605	0.2513481	-4.637413	3.53E-06	1.25E-05
ZNF839P1	1.1080636	-1.172797	0.2529023	-4.637352	3.53E-06	1.25E-05
LINC01494	0.857877	-1.030605	0.2225038	-4.631851	3.62E-06	1.28E-05
PCNPP3	0.5305609	-1.267207	0.2738481	-4.627407	3.70E-06	1.31E-05
LINC01214	1.9910075	-1.529136	0.3306394	-4.624785	3.75E-06	1.33E-05
OR1Q1	0.9194642	-1.279098	0.2771411	-4.615333	3.92E-06	1.38E-05
SP9	0.6485377	-2.079671	0.4511416	-4.609796	4.03E-06	1.42E-05
CIDEA	3.3876115	-1.320122	0.2866614	-4.605163	4.12E-06	1.45E-05
RLBP1	1.5659803	-1.027647	0.223218	-4.603784	4.15E-06	1.45E-05
GPR87	8.7230881	-1.309879	0.2845948	-4.602609	4.17E-06	1.46E-05
HMGB3P21	0.6597846	-1.183606	0.2571765	-4.602311	4.18E-06	1.46E-05
PRSS54	1.2087192	-1.06393	0.2313543	-4.598705	4.25E-06	1.49E-05
LINC00868	1.1363052	-1.117462	0.2433173	-4.592612	4.38E-06	1.53E-05
CRH	1.5512961	-1.530847	0.3333802	-4.591896	4.39E-06	1.53E-05
HNRNPA1P3	0.4680008	-1.156418	0.2519875	-4.589188	4.45E-06	1.55E-05
ISL1	1.1280534	-1.342909	0.2927585	-4.587089	4.49E-06	1.56E-05
AVP	2.1705437	-1.217996	0.2655299	-4.587041	4.50E-06	1.56E-05
MIR6124	0.4954864	-1.023469	0.2231502	-4.586458	4.51E-06	1.57E-05
BHLHE23	0.633355	1.1540031	0.2518248	4.5825637	4.59E-06	1.60E-05

GM2AP2	0.6925845	-1.019636	0.2227005	-4.578509	4.68E-06	1.62E-05
MBL2	5.5005319	-1.457323	0.3183699	-4.577452	4.71E-06	1.63E-05
CSAG2	0.6038562	-1.67909	0.3668863	-4.576595	4.73E-06	1.64E-05
LINC01142	0.6038128	-1.160888	0.2538134	-4.573785	4.79E-06	1.66E-05
SIX3	4.2950309	-1.040033	0.2277391	-4.566775	4.95E-06	1.71E-05
IGKV1D-13	20.450664	-1.025565	0.2246888	-4.56438	5.01E-06	1.73E-05
ZAN	16.761186	-1.107327	0.243516	-4.547245	5.44E-06	1.87E-05
LINC00703	0.5190854	-1.342922	0.2955337	-4.544059	5.52E-06	1.89E-05
LINC01523	1.0479191	-1.220408	0.2695535	-4.527518	5.97E-06	2.03E-05
IGLV3-30	0.611486	-1.632289	0.360598	-4.526618	5.99E-06	2.04E-05
OR11A1	1.1293244	-1.264014	0.2795234	-4.522033	6.12E-06	2.08E-05
FABP7P2	0.7802413	-1.177657	0.2611124	-4.510154	6.48E-06	2.19E-05
SLC6A20	357.11841	-1.019233	0.226383	-4.502251	6.72E-06	2.27E-05
LINC00330	1.7052068	-1.130866	0.2511863	-4.5021	6.73E-06	2.27E-05
SAMSN1-AS1	0.6899996	-1.166462	0.2593679	-4.497326	6.88E-06	2.32E-05
CAV3	1.2052337	-1.005637	0.2237548	-4.494372	6.98E-06	2.35E-05
NKX2-1	0.6402732	-1.563711	0.3483348	-4.489103	7.15E-06	2.40E-05
SSX5	0.7992538	-1.291397	0.287779	-4.487461	7.21E-06	2.42E-05
PDYN	0.9059127	-1.114918	0.2486359	-4.48414	7.32E-06	2.46E-05
FAM167A-AS	1.3344398	-1.200803	0.2680535	-4.479712	7.47E-06	2.50E-05
RNY4P34	1.0510799	-1.197262	0.2673148	-4.478847	7.50E-06	2.51E-05
C8orf74	1.7759782	-1.104809	0.2466896	-4.478539	7.52E-06	2.52E-05
TMEM239	0.3689512	-1.767152	0.3953811	-4.46949	7.84E-06	2.62E-05
SPATA42	0.9795172	-1.037964	0.2323841	-4.466585	7.95E-06	2.65E-05
LINC00052	2.0543396	-1.457721	0.3266104	-4.463179	8.08E-06	2.69E-05
ASZ1	0.902453	-1.293212	0.2897527	-4.463157	8.08E-06	2.69E-05
MIR4506	0.5709279	-1.055849	0.2368484	-4.45791	8.28E-06	2.75E-05
AMTN	0.6659993	-2.164122	0.4857777	-4.454964	8.39E-06	2.78E-05
LINC00355	1.0346551	-1.496348	0.3363719	-4.448494	8.65E-06	2.86E-05
GOLGA8J	0.6478059	-1.120096	0.2518381	-4.447685	8.68E-06	2.87E-05
CLPSL1	1.0866558	-1.355121	0.3050949	-4.441638	8.93E-06	2.95E-05
KHDC3L	0.9587467	-1.021884	0.2301083	-4.440883	8.96E-06	2.96E-05
RAX	0.8478657	-1.186545	0.2675004	-4.435678	9.18E-06	3.02E-05
LRTM2	1.1660191	-1.025869	0.2319731	-4.422361	9.76E-06	3.21E-05
LINC00645	46.186479	1.0860894	0.2456053	4.4220919	9.77E-06	3.21E-05
MIR3197	1.3537878	-1.092498	0.2470801	-4.421635	9.80E-06	3.22E-05
IL36RN	0.6621409	-1.186576	0.2684094	-4.420769	9.84E-06	3.23E-05
TBATA	2.7329024	-1.050652	0.2379468	-4.415492	1.01E-05	3.30E-05
RNU7-97P	0.6497047	-1.081891	0.2451673	-4.412869	1.02E-05	3.34E-05
RNA5SP466	0.8542618	-1.033144	0.2343925	-4.407753	1.04E-05	3.41E-05
HELT	0.70153	-1.430138	0.3245938	-4.40593	1.05E-05	3.44E-05
FGFBP1	3.2446139	-1.192432	0.2709126	-4.401537	1.07E-05	3.51E-05
CNTNAP4	1.5705424	-1.04591	0.2377299	-4.399572	1.08E-05	3.54E-05
LINC01099	2.4789348	-1.123397	0.2557938	-4.391808	1.12E-05	3.66E-05
AQP2	110.27356	1.4427388	0.3285062	4.391816	1.12E-05	3.66E-05
EPHA5	2.4572198	-1.242152	0.283	-4.38923	1.14E-05	3.70E-05
LINC00466	0.5048033	-1.507734	0.3441689	-4.380795	1.18E-05	3.83E-05
SERPINA11	2.1672837	-1.11833	0.2552805	-4.38079	1.18E-05	3.83E-05
LINC01570	0.623821	-1.525231	0.3492954	-4.366594	1.26E-05	4.07E-05
NR0B1	6.6964948	-1.76176	0.4037387	-4.363613	1.28E-05	4.12E-05
WASIR1	0.5807055	-1.303455	0.2990011	-4.359365	1.30E-05	4.20E-05

ACTL6B	2.0991641	-1.071479	0.2459219	-4.356988	1.32E-05	4.24E-05
GABRG2	0.9517507	-1.463815	0.3362946	-4.352777	1.34E-05	4.31E-05
OR7E91P	0.898051	-1.133442	0.2608598	-4.345023	1.39E-05	4.46E-05
TGM7	1.720255	-1.613379	0.3717863	-4.339534	1.43E-05	4.56E-05
OR2AT4	1.4190994	-1.294218	0.2984777	-4.336061	1.45E-05	4.63E-05
LINC01167	1.9744063	-1.380778	0.3184809	-4.335513	1.45E-05	4.64E-05
SMIM21	0.7410874	-1.168946	0.2696421	-4.335174	1.46E-05	4.65E-05
IL22	0.6946637	-1.01674	0.2346941	-4.332193	1.48E-05	4.71E-05
MIR4539	1.1717044	-1.097726	0.2536642	-4.327477	1.51E-05	4.80E-05
OR10A2	0.7452684	-1.086833	0.251517	-4.321111	1.55E-05	4.94E-05
ARNTL2-AS1	0.4734023	-1.163156	0.2691883	-4.320975	1.55E-05	4.94E-05
CCDC144NL	2.4798118	-1.017013	0.2355253	-4.318062	1.57E-05	5.00E-05
RN7SL200P	1.4190187	-1.00431	0.2326614	-4.316615	1.58E-05	5.03E-05
CAMKV	3.8082829	-1.128167	0.262686	-4.294734	1.75E-05	5.52E-05
AMY2A	1.1797852	-1.239867	0.2889221	-4.291355	1.78E-05	5.60E-05
SNX18P24	1.4713679	-2.079219	0.4862432	-4.276088	1.90E-05	5.97E-05
LINC01192	1.5072251	-1.28141	0.2997304	-4.27521	1.91E-05	5.99E-05
PITX2	118.29315	-1.031106	0.2413137	-4.272886	1.93E-05	6.05E-05
PSG10P	0.8615513	-1.455666	0.3409573	-4.269351	1.96E-05	6.14E-05
RNU6-1004F	0.7028856	-1.05454	0.2472997	-4.264218	2.01E-05	6.27E-05
IGHVIII-67	0.5424274	-1.537735	0.3606957	-4.263248	2.01E-05	6.29E-05
NLRP10	0.6438944	-1.109205	0.260313	-4.261044	2.03E-05	6.35E-05
TAC1	6.8176375	-1.200286	0.2819448	-4.257167	2.07E-05	6.45E-05
LINC00536	1.0415363	-1.084705	0.2552941	-4.248846	2.15E-05	6.68E-05
MSMB	3.3830462	-1.265673	0.2984994	-4.240121	2.23E-05	6.92E-05
IGHV3-50	0.7872712	-1.326796	0.3129162	-4.2401	2.23E-05	6.93E-05
LINC01282	0.3951678	-1.220213	0.2878034	-4.239745	2.24E-05	6.93E-05
ARL2BPP6	0.9106807	-1.505246	0.35544	-4.234882	2.29E-05	7.08E-05
NPM1P10	0.7977611	-1.355276	0.3211419	-4.220177	2.44E-05	7.52E-05
VAX1	1.2620148	-1.145205	0.2714236	-4.219254	2.45E-05	7.54E-05
HMGA1P1	0.5080278	-1.083064	0.2569968	-4.214311	2.51E-05	7.70E-05
HTR2C	1.1174075	-1.320384	0.313447	-4.212465	2.53E-05	7.76E-05
ENPP7P9	0.7628459	-1.117241	0.2652665	-4.21177	2.53E-05	7.78E-05
ST6GAL2-IT	0.8388546	-1.048616	0.2492252	-4.207505	2.58E-05	7.92E-05
KCNH5	2.7352478	-1.111827	0.2642667	-4.207218	2.59E-05	7.93E-05
IGHV3OR16-	0.8373945	-1.642247	0.3906298	-4.204101	2.62E-05	8.03E-05
IGLV7-35	0.5894239	-1.623528	0.3863045	-4.202717	2.64E-05	8.07E-05
IGLV5-37	15.4428	-1.176315	0.2802203	-4.197823	2.69E-05	8.24E-05
DYTN	0.8509931	-1.048164	0.2501569	-4.190026	2.79E-05	8.51E-05
FAM216B	0.7029037	-1.238096	0.2955145	-4.189628	2.79E-05	8.52E-05
SERPINB2	5.4003875	-1.021963	0.2440485	-4.187541	2.82E-05	8.59E-05
SLC6A2	4.0100439	-1.170944	0.2803871	-4.176168	2.96E-05	9.01E-05
IAPP	0.521366	-2.086246	0.4997691	-4.17442	2.99E-05	9.07E-05
LINC00518	0.6178045	-1.308789	0.3143612	-4.163328	3.14E-05	9.50E-05
PLCH1-AS2	0.4965552	-1.1944	0.2874024	-4.155846	3.24E-05	9.79E-05
KIF4CP	0.974445	-1.014907	0.2445792	-4.149604	3.33E-05	0.000100417
LINC00879	1.3504822	-2.069584	0.4997014	-4.141641	3.45E-05	0.000103641
IGKV1OR-2	0.5833241	-1.427275	0.3448268	-4.139107	3.49E-05	0.000104662
SNCA-AS1	1.0372788	-1.003659	0.2428597	-4.132671	3.59E-05	0.000107402
PPIAP10	0.6154152	-1.062332	0.257179	-4.130711	3.62E-05	0.000108248
BPIFB1	11.806434	-1.037706	0.2512546	-4.130098	3.63E-05	0.000108523

TBC1D3D	4.6667034	-1.634496	0.3957632	-4.129985	3.63E-05	0.000108568
FKBP4P6	0.7231322	-1.05676	0.2559217	-4.129232	3.64E-05	0.00010891
FRG2B	0.6772816	-1.619666	0.3936743	-4.114228	3.88E-05	0.000115695
HEPACAM2	61.397407	1.2698455	0.308701	4.1135123	3.90E-05	0.000116015
ALPP	0.7922139	-1.166877	0.2840977	-4.10731	4.00E-05	0.000118988
OR10AH1P	0.5947129	-1.362786	0.3320273	-4.104439	4.05E-05	0.000120377
OPALIN	0.9837183	-1.462924	0.3582101	-4.083984	4.43E-05	0.000130592
CHL1-AS2	2.5875516	-1.357903	0.3325488	-4.083319	4.44E-05	0.000130905
HMGB1P48	0.3719312	-1.055251	0.2587028	-4.07901	4.52E-05	0.000133167
IGKV1OR2-1	0.5775427	-1.335684	0.3275729	-4.077517	4.55E-05	0.00013397
OR1E1	0.7965672	-1.538664	0.3787132	-4.062874	4.85E-05	0.000142012
LINC00380	0.8299684	1.0079699	0.2481138	4.0625311	4.85E-05	0.000142202
SNX18P23	0.4888998	-1.085686	0.2673417	-4.061042	4.89E-05	0.000143055
SPACA4	1.7941145	-1.021379	0.2517249	-4.057521	4.96E-05	0.000144928
OR10K2	0.969269	1.0668565	0.2630976	4.0549837	5.01E-05	0.000146403
BOLA3P2	1.0261856	-1.258972	0.3105194	-4.054408	5.03E-05	0.000146715
RNU6-558P	0.4955208	-1.138122	0.2811208	-4.048515	5.15E-05	0.000150148
AHSG	10.934879	1.2105551	0.299329	4.044229	5.25E-05	0.000152738
RNA5SP174	1.1155147	-1.344001	0.3330177	-4.035825	5.44E-05	0.000158016
LINC01497	1.089156	-1.119829	0.2780133	-4.027968	5.63E-05	0.000162968
IGHD3-10	0.7300845	-2.058039	0.5113078	-4.02505	5.70E-05	0.00016485
ADAM5	1.0479635	-1.111461	0.2761474	-4.024883	5.70E-05	0.000164956
RNU6-1101F	0.3679331	-1.280432	0.3184061	-4.021382	5.79E-05	0.000167196
FOXG1-AS1	0.4394977	-1.841643	0.4579881	-4.021159	5.79E-05	0.00016732
CCDC185	1.6298245	-1.376713	0.3428299	-4.015731	5.93E-05	0.000170915
SNAP91	9.7916689	-1.059922	0.2640779	-4.01367	5.98E-05	0.000172256
LINC01363	0.9953272	-1.011351	0.2519799	-4.013616	5.98E-05	0.000172284
RPS3AP24	0.6073128	-1.007793	0.2517242	-4.00356	6.24E-05	0.000179094
LIN28B	0.665692	-1.71491	0.428541	-4.001741	6.29E-05	0.000180394
LINC01299	0.7063541	-1.061317	0.2653583	-3.999561	6.35E-05	0.000181957
OR10J6P	0.9984087	-1.188258	0.297175	-3.998513	6.37E-05	0.000182681
RPL17P35	0.446626	-1.383838	0.3464459	-3.994383	6.49E-05	0.000185627
RNU6-948P	0.5837465	-1.034677	0.2593008	-3.990258	6.60E-05	0.000188626
RNU6-238P	0.7022099	-1.001346	0.2509696	-3.989908	6.61E-05	0.000188868
HDGFL1	0.4287587	-1.193132	0.2992319	-3.987316	6.68E-05	0.00019083
PSG9	2.3458208	-1.375171	0.3458481	-3.976228	7.00E-05	0.000199283
RNASEH1P2	0.6780928	-1.095246	0.2758072	-3.971058	7.16E-05	0.000203319
OFCC1	1.2613175	-1.037209	0.2615752	-3.965241	7.33E-05	0.000207811
NAA11	0.7869986	-1.127271	0.2843507	-3.964368	7.36E-05	0.000208506
IGHJ2P	0.4894757	-1.49091	0.3768087	-3.956676	7.60E-05	0.00021479
FGB	8759.3266	-1.146668	0.2899139	-3.955201	7.65E-05	0.000215994
CDY4P	0.6341634	-1.108707	0.2803343	-3.954947	7.66E-05	0.000216196
CKMT1A	30.591073	-1.042887	0.2637913	-3.953455	7.70E-05	0.000217465
FOXI3	0.8852036	-1.100977	0.2785537	-3.952478	7.73E-05	0.000218271
NKAIN3	1.6135194	-1.277651	0.3232585	-3.952412	7.74E-05	0.000218303
PIEZO1P2	0.7365221	-1.221357	0.3091272	-3.950983	7.78E-05	0.000219469
PAGE1	0.6859302	-1.729481	0.4383305	-3.945611	7.96E-05	0.00022404
TCEAL4P1	0.5074562	-1.223776	0.3114007	-3.929909	8.50E-05	0.000237984
FAM25A	0.8817683	-1.512709	0.3852709	-3.926351	8.62E-05	0.000241175
HTR3D	0.6646102	-1.048833	0.2679807	-3.913837	9.08E-05	0.000253189
ADIPOQ	4.4821989	-1.311398	0.3354871	-3.908937	9.27E-05	0.000257985

MC3R	0.4945771	-2.051446	0.5254488	-3.904178	9.45E-05	0.000262764
DIO2-AS1	0.5590245	-1.167182	0.2995157	-3.896896	9.74E-05	0.000270069
TLX1	2.758644	-1.043027	0.2677539	-3.89547	9.80E-05	0.000271508
DNAJC19P7	0.5960261	-1.035845	0.2660298	-3.893719	9.87E-05	0.000273287
OR7E140P	0.3965667	-1.160165	0.2985245	-3.88633	0.0001018	0.000281132
ANKRD20A9F	1.5580477	-1.15377	0.297181	-3.882381	0.0001034	0.000285343
MRPS36P4	0.452241	-1.046912	0.2713037	-3.858818	0.0001139	0.0003121
OR12D2	0.6905961	-1.871356	0.4855811	-3.853848	0.0001163	0.000318033
POU6F2-AS1	7.3266287	-1.059721	0.2749903	-3.853669	0.0001164	0.000318224
OPTC	0.6473463	-1.112504	0.2887207	-3.853219	0.0001166	0.000318769
LINC00364	0.9054878	-1.340751	0.3481831	-3.850708	0.0001178	0.000321734
MIR217HG	0.7069033	-1.033859	0.2690949	-3.841988	0.000122	0.000332393
TRIM36-IT1	1.2843827	-1.149202	0.2999543	-3.831255	0.0001275	0.00034607
CTBP2P2	1.2640083	-1.16545	0.3042459	-3.830617	0.0001278	0.000346839
LINC00051	0.7396233	-1.061208	0.2778451	-3.819424	0.0001338	0.00036184
ERVMER61-1	0.7229285	-1.554641	0.4072064	-3.817821	0.0001346	0.00036392
ERC2-IT1	0.4752403	-1.082385	0.2835344	-3.817472	0.0001348	0.000364399
GABRA5	6.2038858	-1.159822	0.3042658	-3.811871	0.0001379	0.00037184
PLSCR5	0.4448847	-1.182829	0.3103331	-3.811483	0.0001381	0.000372379
OR2AT2P	0.802216	-1.553622	0.4079563	-3.808305	0.0001399	0.000376803
CHRNB3	0.60202	-1.101291	0.289867	-3.799299	0.0001451	0.000389754
KLK1	229.35893	1.1552729	0.3042186	3.7975088	0.0001462	0.000392267
TMPRSS11E	7.2305402	1.1246736	0.29658	3.7921417	0.0001494	0.000400109
TUBB8P11	1.1107453	-1.011234	0.2671084	-3.785856	0.0001532	0.000409885
SOHLH1	0.8413888	-1.007894	0.2662737	-3.78518	0.0001536	0.000410824
MUC21	0.6112777	-1.045763	0.2763219	-3.784582	0.000154	0.000411688
RN7SL356P	0.4988414	-1.220774	0.324271	-3.764673	0.0001668	0.00044337
FGF19	1.0768823	-1.153487	0.3064142	-3.76447	0.0001669	0.000443703
GAPDHP34	0.4939751	-1.087052	0.2892521	-3.758147	0.0001712	0.000454292
ARHGAP36	3.583648	-1.021757	0.2725505	-3.748871	0.0001776	0.000469832
LUZP4	0.5692056	-1.565365	0.4178705	-3.746052	0.0001796	0.000474768
SERPINB13	1.3809336	-1.026069	0.2739343	-3.745675	0.0001799	0.000475366
CST4	0.9351694	-1.419741	0.3798084	-3.738046	0.0001855	0.000488638
MCHR2	0.6565535	-1.146152	0.3076143	-3.725937	0.0001946	0.000510587
IGHV3-33-2	0.5019449	-1.166505	0.3133546	-3.722635	0.0001972	0.000516787
TUBA3GP	0.7324322	-1.272891	0.3421362	-3.720421	0.0001989	0.000521058
LINC01405	2.0987372	-1.070012	0.2880595	-3.714551	0.0002036	0.000532443
SLC22A25	0.8834523	-1.040929	0.2804782	-3.711267	0.0002062	0.000538757
AMBN	0.6110575	-1.073878	0.28992	-3.70405	0.0002122	0.000553107
NPSR1	0.9946102	-1.257267	0.3399195	-3.69872	0.0002167	0.000564077
MUCL1	3.1906248	-1.062148	0.2874304	-3.695322	0.0002196	0.000571302
IGLV11-55	0.8534639	-1.070699	0.2902807	-3.688494	0.0002256	0.000585485
ATP8A2P1	0.7973202	-1.223015	0.331663	-3.687525	0.0002264	0.000587451
VN1R7P	1.5147912	-1.147414	0.3113801	-3.684929	0.0002288	0.000593155
LINC01568	0.8297332	-1.147357	0.3128814	-3.667068	0.0002453	0.000632962
UBTFL8	1.0861891	-1.03004	0.2812287	-3.662642	0.0002496	0.000643112
LINC01387	0.7635218	-1.505868	0.4117047	-3.657642	0.0002545	0.000654727
KRT9	0.6814559	-1.146127	0.3137041	-3.653529	0.0002587	0.000664334
MAGEB18	0.475582	-1.947922	0.5333177	-3.652462	0.0002597	0.00066679
AGBL1-AS1	0.9195198	-1.175223	0.3222573	-3.646847	0.0002655	0.000679969
IL36B	1.1378341	-1.115682	0.3066963	-3.637743	0.000275	0.000702198

LINC00397	0.8615828	-1.233084	0.3390806	-3.636552	0.0002763	0.000705121
LINC00858	2.1627673	-1.507642	0.4145993	-3.636383	0.0002765	0.000705501
RNA5SP207	0.5355368	-1.024188	0.281915	-3.632967	0.0002802	0.000714203
BARHL2	0.8448395	-1.059259	0.2916896	-3.631458	0.0002818	0.000718017
PIP	8.6789133	-1.035383	0.2852772	-3.629394	0.0002841	0.00072311
SPAG11A	0.5638259	-1.402446	0.3868304	-3.62548	0.0002884	0.000733213
SNORA74A	1.2176123	-1.030739	0.284354	-3.624843	0.0002891	0.000734909
RNU6-1109F	0.547103	-1.037496	0.2867381	-3.618269	0.0002966	0.000752242
VSTM2A	6.2287551	-1.211139	0.3356842	-3.607971	0.0003086	0.000780248
KLK3	10.273894	-1.18073	0.3274653	-3.605665	0.0003114	0.000786437
DNAJC19P6	0.6666213	-1.029479	0.2858777	-3.601117	0.0003169	0.000798805
C16orf82	0.7547519	-1.041914	0.2896473	-3.59718	0.0003217	0.000810106
OR10AC1	0.6978339	-1.038259	0.2886553	-3.596883	0.0003221	0.000810796
LSAMP-AS1	0.4170975	-1.602085	0.4460247	-3.59192	0.0003283	0.000824789
BPIFB2	0.8102397	-1.346119	0.3749043	-3.590568	0.00033	0.000828745
HMX2	0.7139537	-1.584995	0.4416984	-3.58841	0.0003327	0.000835108
CT45A1	0.511686	-1.692734	0.4719048	-3.587024	0.0003345	0.000839173
RN7SL448P	0.970136	-1.01904	0.2847001	-3.579344	0.0003445	0.00086215
IGHV1OR15-	0.3876233	-1.451569	0.4063382	-3.572318	0.0003538	0.00088375
OR4N2	0.6318527	-1.253193	0.3508993	-3.571375	0.0003551	0.000886586
NTAN1P3	0.4209287	-1.04769	0.2935147	-3.569462	0.0003577	0.000892525
MRPS36P2	0.4238151	-1.243608	0.3485438	-3.568011	0.0003597	0.000896817
MAGEB10	0.5740803	-1.62231	0.4547857	-3.567196	0.0003608	0.000899353
CKS1BP3	1.9791036	-1.066217	0.2990956	-3.564804	0.0003641	0.000906804
CRYAA	0.6191452	-1.649974	0.4629195	-3.564278	0.0003649	0.00090828
LINC01297	0.9517892	-1.125685	0.3162876	-3.559054	0.0003722	0.000924645
OR6S1	0.7977502	-1.221587	0.3433502	-3.557846	0.0003739	0.000928485
VGLL2	0.4580943	-1.418853	0.3997667	-3.549202	0.0003864	0.000956685
MIR3196	1.0145268	-1.273025	0.3589246	-3.546777	0.00039	0.000964717
RNA5SP92	0.2540405	-1.07656	0.3057044	-3.521573	0.000429	0.001052271
IGLVI-63	0.6978293	-1.009961	0.2871391	-3.517323	0.0004359	0.00106807
LINC01549	0.5097049	-1.136217	0.3231755	-3.515788	0.0004385	0.001073785
OR1F1	0.7398472	-1.328956	0.3782415	-3.513511	0.0004422	0.001082182
SPINT4	0.3440372	-1.30754	0.3721797	-3.513194	0.0004428	0.001083232
DCAF8L2	0.5000232	-1.328691	0.3797659	-3.49871	0.0004675	0.001138342
NEUROD1	1.7090695	-1.014313	0.2901321	-3.496038	0.0004722	0.001149101
SLC7A13	11.390563	1.0097808	0.2899301	3.4828422	0.0004961	0.001201943
CYP2AB1P	0.9191005	-1.19923	0.3444345	-3.481734	0.0004982	0.001206166
GABRA6	0.5835388	-1.349053	0.3875004	-3.481424	0.0004988	0.00120743
IGKV1D-37	0.4201676	-1.805947	0.5198345	-3.47408	0.0005126	0.001238093
E2F6P4	0.4372159	-1.136874	0.3272673	-3.473839	0.0005131	0.001239068
SPAM1	0.8304914	-1.11532	0.3212876	-3.471406	0.0005177	0.001249664
DIRC1	0.3756101	-1.341172	0.3880168	-3.456478	0.0005473	0.001314957
NKX2-4	0.5061637	-1.486628	0.4309787	-3.449424	0.0005618	0.00134699
IGLV3-32	1.316954	-1.092832	0.3168511	-3.449041	0.0005626	0.001348607
SLC6A15	19.649759	-1.056562	0.3076492	-3.434307	0.0005941	0.001417498
APCS	24.825863	-1.192383	0.3484049	-3.422407	0.0006207	0.001476278
PWRN3	0.9271449	-1.152001	0.336762	-3.420817	0.0006243	0.00148421
SIGLEC29P	0.6232164	-1.273677	0.3724793	-3.419457	0.0006275	0.001491244
NPIPA7	0.4383077	-1.085009	0.3176703	-3.415519	0.0006366	0.001510844
ELMO2P1	0.633691	-1.102263	0.3238788	-3.403321	0.0006657	0.001575249

MED15P9	0.4287076	-1.561371	0.459159	-3.400502	0.0006726	0.001589778
TOMM20P3	0.5431781	-1.131052	0.3326809	-3.399811	0.0006743	0.001593452
LINC01399	1.0351927	-1.094962	0.3234631	-3.385122	0.0007115	0.00167445
NDUFS5P5	0.6960497	-1.0152	0.300041	-3.383537	0.0007156	0.001683602
ANKRD62P1-	0.5295883	-1.067153	0.3172075	-3.364211	0.0007676	0.00179553
OR10J3	0.5172096	-1.03828	0.3091995	-3.357962	0.0007852	0.001833194
TCHHL1	0.4180757	-1.539473	0.4586548	-3.356495	0.0007894	0.001842651
PSG11	0.880451	-1.045873	0.3128971	-3.342545	0.0008301	0.001929594
LINC01164	0.4088904	-1.312561	0.3930745	-3.339216	0.0008402	0.001950691
TMEM114	0.8840351	-1.049964	0.3151169	-3.331983	0.0008623	0.001998399
KRT77	1.70397	-1.065318	0.3203649	-3.325326	0.0008832	0.002043597
PHOX2B	1.0379875	-1.130538	0.3410655	-3.314723	0.0009173	0.002117429
IGKV2-40	0.4817643	-1.73724	0.5250631	-3.308631	0.0009375	0.002159513
IGHV1OR15-	0.4717005	-1.45903	0.4414046	-3.305426	0.0009483	0.002183098
UBE2V2P1	0.5245727	-1.065169	0.3228208	-3.299567	0.0009683	0.002224976
SPACA1	1.1846353	-1.173745	0.3563704	-3.293611	0.0009891	0.002267663
ANKRD54P1	0.5525181	-1.87283	0.570532	-3.282603	0.0010285	0.002350213
VTRNA2-2P	0.6610631	-1.109784	0.3382401	-3.281054	0.0010342	0.002362419
TAS2R38	0.5717189	-1.113309	0.3403162	-3.271397	0.0010702	0.002437107
IGKV6D-41	0.8275257	-1.230068	0.378614	-3.248871	0.0011586	0.002619498
RNA5SP334	0.331316	-1.569429	0.483398	-3.24666	0.0011677	0.002638468
RN7SL538P	0.5523283	-1.016202	0.3132017	-3.244559	0.0011763	0.002657015
RAET1M	0.4780746	-1.125952	0.3481097	-3.234474	0.0012187	0.002746003
VN1R53P	0.5652412	-1.209073	0.3739172	-3.233532	0.0012227	0.002753658
VN1R8P	0.6194249	-1.175326	0.3640405	-3.228559	0.0012442	0.002798822
KRT38	0.3821698	-1.447545	0.4483854	-3.228349	0.0012451	0.002800738
RNU6-346P	0.3553275	-1.268144	0.3931986	-3.2252	0.0012588	0.002829651
IGLVI-56	0.5827581	-1.517945	0.470913	-3.223409	0.0012667	0.002845403
WFDC10A	0.336022	-1.349565	0.4202156	-3.211601	0.00132	0.002954515
IGHD3-22	0.5468864	-1.282636	0.4005173	-3.202448	0.0013627	0.00304273
GFY	0.4163977	-1.031946	0.3226302	-3.198543	0.0013812	0.003079532
RPL7AP69	0.5904218	-1.053423	0.3299987	-3.192203	0.0014119	0.003141861
CTD-2194D2	0.4597199	-1.810649	0.5682778	-3.186204	0.0014415	0.003201094
DNTT	5.8785022	-1.01809	0.3212285	-3.169363	0.0015277	0.003375051
APOBEC1	0.5774323	-1.037595	0.3286566	-3.157079	0.0015936	0.003506548
MED15P5	0.6943042	-1.822936	0.5788271	-3.149362	0.0016363	0.003591399
RN7SKP75	0.8935797	-1.028711	0.3280957	-3.135397	0.0017162	0.003751709
RN7SKP202	0.4755224	-1.05489	0.3381977	-3.11915	0.0018137	0.003943047
TLX3	0.5233898	-1.153275	0.3704232	-3.1134	0.0018495	0.004014327
GJD2	3.5488948	1.1674629	0.3753146	3.1106245	0.0018669	0.004048832
FOXI1	50.65048	1.1303233	0.3667971	3.0816037	0.0020589	0.004428974
KRT16P2	0.7723195	-1.344471	0.4365828	-3.079532	0.0020733	0.004457268
RN7SKP109	0.8763399	-1.03968	0.3391412	-3.065627	0.0021721	0.004650677
TBC1D3K	0.5112003	-1.086927	0.3560677	-3.052585	0.0022688	0.004842236
LINC01120	0.3726366	-1.143609	0.3773667	-3.030498	0.0024415	0.005174388
IGKV3OR2-5	0.443784	-1.510689	0.4996177	-3.023691	0.0024971	0.005280694
LINC00993	1.0973832	-1.149296	0.38038	-3.021442	0.0025157	0.005314894
DEFB132	0.8200931	-1.70096	0.5641329	-3.015176	0.0025683	0.005417329
RN7SKP17	0.4115273	-1.128936	0.3746298	-3.013472	0.0025828	0.005444534
PPP1R2P2	0.3307314	-1.092231	0.362554	-3.012601	0.0025902	0.005458498
CYP4F34P	2.1658611	-1.396902	0.4643223	-3.008474	0.0026256	0.00552416

VSTM2A-OT1	0.5290579	-1.330012	0.44318	-3.001065	0.0026904	0.005648424
OR4K2	0.810336	-1.048355	0.3494989	-2.999594	0.0027034	0.005673332
ALX3	0.3736323	-1.434425	0.480379	-2.986028	0.0028263	0.005908255
MS4A12	0.6335316	-1.022242	0.3447901	-2.964824	0.0030286	0.006299874
RNU1-136P	0.6895132	-1.016183	0.3435248	-2.958108	0.0030953	0.006426884
SLC25A39P2	0.7356694	-1.227903	0.4164117	-2.948772	0.0031904	0.00660639
FAM47C	0.3191151	-1.272482	0.4320055	-2.945521	0.0032241	0.006671801
SIAH1P1	0.4548395	-1.106754	0.3759126	-2.944178	0.0032381	0.006697656
MARK2P5	0.6903013	-1.008442	0.3432997	-2.937498	0.0033087	0.006832014
MAGEB16	0.4674409	-1.091209	0.3737335	-2.919753	0.0035031	0.007198783
MIR8082	0.3279362	-1.071418	0.3670501	-2.918997	0.0035116	0.007214225
MAGEC1	1.3989858	-1.084423	0.373332	-2.904716	0.0036759	0.007518207
C10orf120	0.5361755	-1.074281	0.3730307	-2.879872	0.0039784	0.008081073
SIX3-AS1	0.3823305	-1.085455	0.37735	-2.876519	0.0040209	0.008158723
GPS2P2	0.7230849	-1.051519	0.3655648	-2.876423	0.0040221	0.008160825
LINC01153	0.4134564	-1.043967	0.3635924	-2.871256	0.0040884	0.008283545
PEX12P1	0.3307578	-1.226374	0.4283443	-2.863058	0.0041957	0.008476237
SEC63P2	0.7051858	-1.032111	0.3627048	-2.845595	0.0044329	0.008907394
TBC1D3	0.6879753	-1.315565	0.4644029	-2.832809	0.0046141	0.009235789
MAGEB1	0.4474584	-1.455795	0.5163021	-2.819658	0.0048075	0.009580424
LINC01029	0.3364702	-1.208822	0.429174	-2.816624	0.0048531	0.00966258
GOLGA8CP	0.287755	-1.007196	0.3577786	-2.815138	0.0048756	0.009699875
OR5A1	0.78577	-1.003228	0.3581402	-2.801215	0.0050911	0.01009089
TEX36	0.3278695	-1.003137	0.3585573	-2.797703	0.0051467	0.01018651
ATP6V1G3	25.288872	1.4601536	0.522908	2.7923719	0.0052323	0.010341872
KCNA10	0.2947582	-1.074181	0.385763	-2.784563	0.00536	0.010568402
PSKH2	0.7096521	-1.263427	0.454073	-2.782432	0.0053953	0.010630942
BCAS2P1	0.4027066	-1.09723	0.3954168	-2.77487	0.0055224	0.010855416
CGB8	0.3297256	-1.20664	0.4359045	-2.768129	0.0056379	0.011061482
DCAF4L2	0.3440952	-1.312167	0.477318	-2.749041	0.005977	0.011659073
RNASEH1P1	0.2750961	-1.19007	0.4355287	-2.732472	0.0062861	0.012213117
RNU6-1191P	0.4639784	-1.115699	0.4099029	-2.721862	0.0064915	0.01257801
CRB3P1	0.4040689	-1.329918	0.490295	-2.712485	0.0066781	0.012904261
LINC00898	0.5101208	-1.095703	0.4047018	-2.707432	0.0067806	0.013087816
RNU6-389P	0.3471773	-1.374533	0.5094915	-2.697852	0.0069788	0.01343509
CSN3	0.6033152	-1.061081	0.3960017	-2.679486	0.0073735	0.014128968
UGT1A12P	0.559973	-1.099384	0.4108466	-2.6759	0.0074529	0.014270542
MAGEA1	0.6532147	-1.444098	0.5410627	-2.669002	0.0076077	0.014530748
RN7SKP137	0.5933242	-1.22794	0.4637324	-2.64795	0.0080982	0.015379013
RPL36P20	0.4985937	-1.036808	0.3918703	-2.645794	0.0081499	0.015465946
LINC00348	0.4985073	-1.011461	0.3829417	-2.641293	0.008259	0.015648193
IGHV7-40	0.3061956	-1.304966	0.4973137	-2.62403	0.0086896	0.016383634
CTB-1I21.1	0.518198	-1.664683	0.6371034	-2.612894	0.0089779	0.016874912
PRORY	0.3782719	-1.296832	0.499038	-2.598664	0.0093587	0.017514154
DSCR8	1.8633572	-1.212081	0.4711374	-2.572669	0.0100918	0.01872736
HIRAP1	0.4523773	-1.552755	0.6048393	-2.567219	0.0102518	0.018997809
LINC00951	0.3402844	-1.207355	0.4748183	-2.542773	0.0109977	0.020252375
IGKV2D-26	0.6014383	-1.042208	0.4103286	-2.539936	0.0110873	0.020396836
MRPL9P1	0.2988364	-1.143595	0.4502848	-2.539715	0.0110943	0.020406129
PEX5L-AS2	0.2792425	-1.12488	0.4464152	-2.519807	0.0117419	0.021473979
IGHV1OR16-	0.3327297	-1.196881	0.4756185	-2.516473	0.0118536	0.021653855

SPANXN1	0.3019354	-1.107574	0.4427732	-2.501447	0.0123687	0.022502816
KRTAP10-12	0.6267724	-1.043697	0.4198219	-2.486047	0.0129171	0.023401437
EPHA5-AS1	0.3108986	-1.500896	0.6046902	-2.48209	0.0130614	0.023639434
MAGEA11	0.349804	-1.063185	0.430769	-2.46811	0.0135829	0.024469941
MRPL45P1	0.4932213	-1.24983	0.5067027	-2.466594	0.0136405	0.024563328
METTL15P3	0.6351842	-1.053005	0.4279886	-2.460357	0.0138799	0.024946543
IGLCOR22-2	0.3675863	-1.301514	0.5292262	-2.459277	0.0139217	0.025009417
IGHVII-60-	0.3112767	-1.103567	0.4497455	-2.453759	0.0141372	0.025360075
KLK9	0.4729388	-1.067062	0.4384298	-2.433826	0.0149402	0.026653205
TMEM207	2.3716386	1.1796143	0.4880917	2.4167882	0.0156581	0.027815082
DSCR4	0.9926506	-1.040282	0.4326059	-2.404688	0.0161863	0.02866493
LINC00388	0.3664234	-1.309871	0.5464756	-2.396943	0.0165325	0.029215458
MIR3972	0.3144407	-1.374608	0.5734871	-2.396928	0.0165332	0.029215458
VN1R48P	0.3341659	-1.096834	0.4582364	-2.393599	0.016684	0.029447502
HBG1	1.6634744	1.2677567	0.5300514	2.3917618	0.0167677	0.029578659
GH2	0.3959241	-1.48722	0.6242965	-2.382233	0.017208	0.030268811
LINC01419	0.2765367	-1.32997	0.5584184	-2.381672	0.0172342	0.030307642
IGLV3-31	0.3487577	-1.073395	0.4584354	-2.34143	0.01921	0.033413277
IGLV3-17	0.4491583	-1.001278	0.4278432	-2.340292	0.0192687	0.03351135
KRTAP5-5	0.3539174	-1.15669	0.4956486	-2.33369	0.019612	0.034035438
IGHD2-15	0.2607391	-1.165984	0.5022645	-2.321454	0.0202623	0.035047446
KRTAP21-3	0.2860247	-1.487645	0.6520743	-2.281404	0.0225246	0.038597587
FMO8P	0.3401154	-1.035236	0.4538268	-2.281125	0.022541	0.038622868
RNU6-1222F	0.255608	-1.110274	0.5042022	-2.202042	0.0276623	0.046328829
CASP14	44.769172	1.0069615	0.4595577	2.1911536	0.0284407	0.047476275
GAGE1	0.413597	-1.334745	0.6118363	-2.181539	0.0291435	0.048507232
