# Supplementary material for: A novel cancer-associated fibroblast signature for kidney renal clear cell carcinoma via integrated analysis of single-cell and bulk RNA-sequencing
Source: Discov Oncol. 2024 Jul 26;15:309. doi: 10.1007/s12672-024-01175-x (PMC11282037; doi:10.1007/s12672-024-01175-x)
Supplement: Supplementary file 3 — (PDF 261 KB) [file 12672_2024_1175_MOESM3_ESM.pdf]

pplementary Table 3. The 22 infiltrated immune cells in 512 KIRC sample

	B cells na	B cells me	Plasma cel	T cells CD	T cells CD	T cells CD	T cells CD
TCGA-3Z-A9	0.097157	0	0.0687308	0.0681764	0	0.1383068	0
TCGA-6D-AA	0.2005466	0	0.1712081	0.1905144	0	0.1066656	0
TCGA-A3-33	0.002146	0.0047782	0.0012983	0.1080281	0	0.297576	0
TCGA-A3-33	0.0003749	0	0.0022785	0.3086887	0	0.0671613	0.0070259
TCGA-A3-33	0.0315721	0	0.0066804	0.0996682	0	0.0931977	0.024586
TCGA-A3-33	0.0730997	0	0.0382705	0.1695483	0	0.1216576	0.0061773
TCGA-A3-33	0.0233946	0	0.0054277	0	0	0.1927679	0
TCGA-A3-33	0.0385973	0	0.0021248	0.0431178	0	0.1033706	0
TCGA-A3-33	0.0070422	0	0.000822	0.0892406	0	0.1887835	0.0052652
TCGA-A3-33	0.0711134	0	0.0202172	0.0807144	0	0.2541039	0
TCGA-A3-33	0.0766752	0	0.0144805	0.1617623	0	0.2593051	0
TCGA-A3-33	0.0270795	0	0.0066229	0.0439294	0	0.1415493	0
TCGA-A3-33	0.0045771	0	0.0017441	0.145388	0	0.2434266	0
TCGA-A3-33	0.0073439	0	0.0023057	0.3255171	0	0.1158771	0
TCGA-A3-33	0.0428895	0	0.0069366	0.071041	0	0.3035908	0.005824
TCGA-A3-33	0.0798487	0	0.0191332	0.0681991	0	0.2860608	0.0175642
TCGA-A3-33	0	0	0.0067123	0	0	0.0627919	0
TCGA-A3-33	0.0235111	0	0.0024703	0.2208254	0	0.1793384	0.0070493
TCGA-A3-33	0.0413736	0	0	0.1642485	0	0.1165888	0
TCGA-A3-33	0.0500539	0	0	0.0571765	0	0.4221872	0.0161745
TCGA-A3-33	0.0432983	0	0.0112962	0.0816922	0	0.2184878	0
TCGA-A3-33	0.0068118	0	0.0016294	0.1480047	0	0.1200879	0.0742476
TCGA-A3-33	0.0387616	0	0.0030115	0.0981816	0	0.126097	0.0039163
TCGA-A3-33	0.1059485	0	0.0219078	0.1865067	0	0.0055375	0.0129082
TCGA-A3-33	0	0	0.0007319	0.4107075	0	0.0799114	0
TCGA-A3-33	0.0432438	0	0.0044213	0.0482958	0	0.2118089	0
TCGA-A3-33	0.0051097	0	0.0041997	0.2307866	0	0.0707915	0.0116842
TCGA-A3-33	0	0.0018736	0.0231606	0.2241803	0	0.1783595	0.0142535
TCGA-A3-33	0.0178255	0	0.0099246	0.2554603	0	0.1994904	0
TCGA-A3-33	0.056444	0	0.0017864	0.1872389	0	0.1902259	0.0124808
TCGA-A3-33	0	0.0031705	0.0009355	0.0658746	0	0.1831618	0
TCGA-A3-33	0.0533409	0	0.0046338	0.1215641	0	0.2368923	0.0360965
TCGA-A3-33	0.0623027	0	0.0067279	0.0760801	0	0.2231864	0
TCGA-A3-33	0	0	0.0023085	0.5113175	0	0	0
TCGA-A3-33	0.0065063	0	0.0102375	0.1592956	0	0.1641501	0
TCGA-A3-33	0.0374885	0	0	0.1070531	0	0.1131572	0.0350139
TCGA-A3-33	0.0039563	0	0	0.000639	0	0.014759	0
TCGA-A3-33	0.0491942	0	0.0016678	0.0705251	0	0.1556084	0
TCGA-A3-33	0.0836539	0	0.0121497	0.1033218	0	0.140997	0.0164543
TCGA-A3-33	0.058498	0	0.0069622	0.1482015	0	0.1563904	0.0241155
TCGA-A3-33	0.0040881	0	0.0035095	0.2656829	0	0.2527117	0
TCGA-A3-33	0.0132769	0	0.002133	0.2235312	0	0.0898976	0
TCGA-A3-33	0.0066316	0	0.0052376	0.0348964	0	0.1046543	0
TCGA-A3-33	0.0497043	0	0.0385708	0.0615334	0	0.1696562	0
TCGA-A3-A6	0	0.0018346	0.0039555	0.0305168	0	0.162108	0
TCGA-A3-A6	0.0232989	0	0.0127893	0.5298211	0	0.0179627	0.0237334
TCGA-A3-A6	0.0827398	0	0.0281232	0.1270522	0	0.1574292	0
TCGA-A3-A6	0.0175771	0	0.0024735	0.1718782	0	0.1204943	0
TCGA-A3-A8	0.0159105	0	0.0056305	0.0857704	0	0.178941	0

TCGA-A3-A8	0.044732	0	0.0226209	0.1221768	0	0.1673698	0.0124355
TCGA-A3-A8	0.0013871	0	0	0.0824007	0	0.0998789	0
TCGA-A3-A8	0.0542405	0	0.0307116	0.4607675	0	0	0
TCGA-A3-A8	0.0469281	0	0.0124478	0.1677799	0	0.2479433	0
TCGA-AK-34	0.0195557	0	0.0043357	0.4612448	0	0.0063862	0.0647758
TCGA-AK-34	0.0115274	0	0	0	0	0.2455426	0
TCGA-AK-34	0.0800736	0	0.0088809	0.1146077	0	0.0642716	0.0019997
TCGA-AK-34	0.0093323	0	0.0051092	0.4789552	0	0.0479833	0.0144299
TCGA-AK-34	0.0402698	0	0.0041406	0.1262837	0	0.2224389	0.0011653
TCGA-AK-34	0.0169547	0	0.018936	0.0275837	0	0.2678227	0
TCGA-AK-34	0	0	0.0023365	0.2850454	0	0.1346285	0.0080651
TCGA-AK-34	0.0755747	0	0.0280703	0.2316207	0	0.2259123	0.01826
TCGA-AK-34	0.0235738	0	0	0	0	0.3199928	0
TCGA-AK-34	0.0708882	0	0	0	0	0.0538313	0
TCGA-AK-34	0.0084809	0	0.0025543	0.1540654	0	0.2523896	0.0142342
TCGA-AK-34	0.0409622	0	0	0.231046	0	0.0564511	0
TCGA-AK-34	0.0681804	0	0.0212373	0.1073417	0	0.2111642	0
TCGA-AK-34	0.016985	0	0.0002445	0.3368597	0	0.0719197	0
TCGA-AK-34	0.0775228	0	0.0246864	0.4608782	0	0.0760504	0
TCGA-AK-34	0.0246881	0	0.0121001	0.2853417	0	0.180886	0
TCGA-AK-34	0.0280462	0	0.074948	0.5909004	0	0	0
TCGA-AK-34	0.0939281	0	0.011535	0.0370812	0	0.0758735	0.0092951
TCGA-AK-34	0.0287501	0	0.0054004	0.3044037	0	0.1592124	0.0248356
TCGA-AK-34	0.0145777	0	0.0027832	0.2128646	0	0.1423367	0.0245755
TCGA-AK-34	0.0210266	0	0.0098653	0.173974	0	0.3095536	0.0241324
TCGA-AK-34	0.0823923	0	0.0140355	0.0177034	0	0.1846216	0
TCGA-AS-37	0.050035	0	0.0046322	0.0665603	0	0.2292383	0.0065162
TCGA-AS-37	0.0046051	0	0.0040413	0.1082998	0	0.1920601	0
TCGA-B0-46	0.0332315	0	0.0069255	0.0406811	0	0.1939309	0.0291445
TCGA-B0-46	0.0187584	0	0.0048862	0.2433654	0	0.1105818	0.0556743
TCGA-B0-46	0.0145198	0	0.0573734	0.3772605	0	0.054115	0
TCGA-B0-46	0.0077709	0	0.0340677	0.4117289	0	0.0691368	0.0189124
TCGA-B0-46	0.0423045	0	0.046492	0.2870474	0	0.0018429	0.0252646
TCGA-B0-46	0.0088781	0	0.0019515	0.0187673	0	0.3493987	0
TCGA-B0-46	0.0157424	0.0366712	0.1067437	0.386897	0	0.0394491	0.0657893
TCGA-B0-46	0	0	0.0051053	0.5225284	0	0	0.1624674
TCGA-B0-46	0	0	0.0023515	0.4333937	0	0	0.0479168
TCGA-B0-47	0.0251695	0	0.0213763	0.3752934	0	0	0.0252924
TCGA-B0-47	0.047073	0	0.0101662	0.1923594	0	0.3131392	0.0083856
TCGA-B0-47	0.0268793	0	0.001507	0.2923998	0	0.1420749	0.0104193
TCGA-B0-47	0.0299192	0	0.0071216	0.3887311	0	0.0540881	0
TCGA-B0-47	0.0647827	0	0.0666482	0.4771833	0	0.0485674	0.0425425
TCGA-B0-47	0.0792915	0	0.0105299	0.2730633	0	0.1791637	0
TCGA-B0-47	0.027883	0	0	0.2178285	0	0.1007634	0.0472245
TCGA-B0-47	0.0178172	0	0.016795	0.2820676	0	0.1151785	0
TCGA-B0-47	0	0	0.0025401	0.5425528	0	0	0.0446767
TCGA-B0-47	0.005455	0	0.0065069	0.3243695	0	0.1074303	0
TCGA-B0-48	0.0559919	0	0.013271	0.3740601	0	0.0619628	0.0140274
TCGA-B0-48	0.0002805	0	0.0129671	0.1540468	0	0.2159735	0.0021753
TCGA-B0-48	0.001046	0	0.0046569	0.0119608	0	0.2407434	0
TCGA-B0-48	0.0012237	0.0139103	0.0267185	0.150218	0	0.1984107	0

TCGA-B0-48	0	0.0004009	0.0968142	0.4208624	0	0	0.0245286
TCGA-B0-48	0.0108993	0	0.0028843	0.129918	0	0.1541911	0.0120546
TCGA-B0-48	0	0.0024461	0.0089993	0.0475081	0	0.1449599	0.0070888
TCGA-B0-48	0.1180398	0	0.2553438	0.1833012	0	0.1221236	0
TCGA-B0-48	0.0721873	0	0.060646	0.4170006	0	0.083263	0.0215447
TCGA-B0-48	0	0	0.01805	0.2381429	0	0.0830778	0
TCGA-B0-48	0.0458532	0	0.009975	0.3866559	0	0.0011617	0
TCGA-B0-48	0.0985085	0	0.030014	0.2816259	0	0.1404434	0.0049028
TCGA-B0-48	0.0053051	0	0.0068033	0.4524887	0	0.0792606	0.0095507
TCGA-B0-48	0.1003254	0	0.1498591	0.2193736	0	0.118844	0
TCGA-B0-48	0.0461638	0	0.0115277	0.2171417	0	0.2453242	0
TCGA-B0-48	0.0967115	0	0.0235977	0.442037	0	0.0433304	0
TCGA-B0-48	0.0184485	0	0	0.1709779	0	0.2178773	0
TCGA-B0-48	0.0437555	0	0.0381344	0.3029295	0	0.089306	0.0154738
TCGA-B0-48	0.0087268	0	0.0004302	0.0663025	0	0.1246633	0
TCGA-B0-48	0.011793	0	0.0079306	0.6749319	0	0	0
TCGA-B0-48	0.0142486	0	0.0031327	0.183333	0	0.1242369	0.0013097
TCGA-B0-48	0.0036766	0	0.000369	0.3198086	0	0.0007327	0
TCGA-B0-48	0.0306971	0	0.0917104	0.1589597	0	0.0938337	0.0015356
TCGA-B0-48	0.0483709	0	0.0034166	0.0290553	0	0.213215	0
TCGA-B0-48	0.0448812	0	0.007624	0.2924645	0	0.1975926	0.0009536
TCGA-B0-48	0.0322435	0	0.0014408	0.0404765	0	0.4633694	0
TCGA-B0-48	0.0034605	0	0.0042532	0.2881256	0	0.2550966	0.0086064
TCGA-B0-48	0.0728409	0	0.0288707	0.4696989	0	0.0398341	0.0081642
TCGA-B0-48	0.0376055	0	0	0.289965	0	0.060315	0
TCGA-B0-48	0.0179108	0	0.0017474	0.1719027	0	0.13249	0
TCGA-B0-48	0.0386501	0	0.009569	0.1898641	0	0.1598828	0.0048881
TCGA-B0-49	0.0429902	0	0.0102082	0.1980118	0	0.1242244	0.0186388
TCGA-B0-50	0.0078441	0	0.0003156	0.1618798	0	0.0453613	0.0018386
TCGA-B0-50	0.0053485	0	0.0055555	0.1583787	0	0.0651756	7.70E-05
TCGA-B0-50	0.1051568	0	0.077292	0.3208645	0	0.0304032	0.001765
TCGA-B0-50	0.0027906	0	0.0238781	0.2655152	0	0.1814166	0.0182102
TCGA-B0-50	0.0453443	0	0.0044395	0.1583051	0	0.0905434	0.0065277
TCGA-B0-50	0.026882	0	0.0007633	0.1775097	0	0.0642929	0
TCGA-B0-50	0	0.0006697	0.0109102	0.2219833	0	0.1559845	0.0752732
TCGA-B0-50	0.0922116	0	0.0041664	0.1818114	0	0.1421746	0
TCGA-B0-50	0.0081755	0	0.0085385	0.6014244	0	0	0.0028131
TCGA-B0-50	0.1164758	0	0.0207839	0.1358863	0	0.0820943	0
TCGA-B0-50	0.0302049	0	0.0073161	0.242749	0	0.1239153	0.0047191
TCGA-B0-50	0.0434869	0	0.0928934	0.003195	0	0.1239315	0.0083971
TCGA-B0-50	0.0434216	0	0.0088796	0.0481531	0	0.1639877	0.041204
TCGA-B0-50	0.1866359	0	0.0525004	0	0	0.1116411	0
TCGA-B0-50	0.0427209	0	0.0101465	0.0666988	0	0.1719883	0
TCGA-B0-51	0.0756224	0	0.009499	0.0756839	0	0.1737015	0
TCGA-B0-51	0.0444818	0	0.0120562	0.2426913	0	0.1993607	0.0057063
TCGA-B0-51	0.0682046	0	0.005481	0.0517907	0	0.2960013	0
TCGA-B0-51	0.1263556	0	0.0350109	0.2989286	0	0.1074466	0
TCGA-B0-51	0	0	0.0020643	0.5208168	0	0	0.0070513
TCGA-B0-51	0.0396637	0	0.009597	0.0837827	0	0.2600091	0.0360239
TCGA-B0-51	0.0578375	0	0.0204875	0.3736416	0	0	0.1611423
TCGA-B0-51	0.0505395	0	0	0.148784	0	0.1587009	0

TCGA-B0-51	0.0222594	0	0.0127325	0.1997513	0	0.2470456	0
TCGA-B0-51	0.0638554	0	0.0131308	0.0818783	0	0.3148877	0.0146813
TCGA-B0-51	0.044276	0	0.0011084	0.1365906	0	0.2906618	0
TCGA-B0-51	0.0289372	0	0.0280118	0	0	0.2214889	0
TCGA-B0-51	0	0	0	0.5132035	0	0.0079818	0
TCGA-B0-51	0.0777586	0	0.0083574	0.0450101	0	0.1503956	0
TCGA-B0-51	0.0036962	0	0.0007602	0.2194456	0	0.219524	0.0199883
TCGA-B0-53	0.0198628	0	0	0.1106814	0	0.2143595	0
TCGA-B0-54	0.0079861	0	0.0052048	0.1991367	0	0.0908026	0
TCGA-B0-54	0.0537501	0	0.012754	0.074345	0	0.2077593	0
TCGA-B0-56	0.1508637	0	0.0044248	0.0556077	0	0.0850462	0.014824
TCGA-B0-56	0.1699998	0	0.0301498	0.1227008	0	0.1355841	0.0200896
TCGA-B0-56	0.021033	0	0.0104028	0.5960532	0	0	0.01598
TCGA-B0-56	0.0957062	0	0.0117408	0.0680076	0	0.0945836	0.0175993
TCGA-B0-56	0.0536604	0	0.0149701	0.0527797	0	0.122939	0.0308927
TCGA-B0-56	0.0886765	0	0.0009266	0.2672518	0	0.0779334	0.0297543
TCGA-B0-56	0.0267136	0	0.0014142	0.3198607	0	0.062972	0
TCGA-B0-56	0.0488512	0	0.0058028	0.3584474	0	0.0825008	0.009958
TCGA-B0-56	0.0786789	0	0.0109845	0.1526184	0	0.2229919	0.0069641
TCGA-B0-56	0.0121444	0	0	0.0948505	0	0.199759	0
TCGA-B0-57	0.0776283	0	0.0107469	0.1433477	0	0.2009057	0
TCGA-B0-57	0.0257234	0	0.0299577	0.2368811	0	0.0514207	0.0360426
TCGA-B0-57	0	0	0.1225646	0.3089337	0	0.1000815	0
TCGA-B0-57	0.0157195	0	0.0058016	0.4018477	0	0.1748822	0
TCGA-B0-57	0.036395	0	0.0128549	0.3086003	0	0.1938172	7.87E-05
TCGA-B0-57	0.1255881	0	0.2495517	0.2661081	0	0.0859436	0.0475098
TCGA-B0-57	0.0808759	0	0.0290568	0.0602471	0	0.1468955	0
TCGA-B0-57	0.0625955	0	0.028849	0.225099	0	0.2258494	0.0046311
TCGA-B0-57	0.0380552	0	0.0027065	0.132832	0	0.1274658	0.006139
TCGA-B0-57	0.0503962	0	0.0137838	0.096517	0	0.2822802	0.0140594
TCGA-B0-57	0.0100716	0	0.0082199	0.3260911	0	0.2347214	0.0080625
TCGA-B0-57	0.005024	0	0.0025717	0.2397354	0	0.0995224	0
TCGA-B0-58	0.025269	0	0.0089755	0.1678486	0	0.1371181	0.0258232
TCGA-B2-39	0.0023434	0	0.0009855	0.0448883	0	0.1348419	0
TCGA-B2-39	0.0087416	0	0.0088587	0.4616654	0	0	0.0265056
TCGA-B2-40	0	0.0278478	0.0459731	0.0402521	0	0.0484222	0.0377574
TCGA-B2-40	0.0125412	0	0.0023264	0.0607364	0	0.1501593	0.0003098
TCGA-B2-41	0.0237844	0	0.0093868	0.2481199	0	0.2066354	0.0005136
TCGA-B2-41	0.0400193	0	0.0094158	0.0691337	0	0.2387737	0.0091982
TCGA-B2-56	0.0044371	0	0	0.1074909	0	0.1394946	0.0164498
TCGA-B2-56	0.0032701	0	0	0.2718314	0	0.1262701	0.0015457
TCGA-B2-56	0.0887281	0	0.0028624	0.0464005	0	0.113792	0
TCGA-B2-56	0.0526532	0	0.0590933	0.0676858	0	0.1832542	0
TCGA-B2-56	0.031826	0	0.0025616	0.4413503	0	0	0
TCGA-B2-A4	0.0507012	0	0.0099865	0.1704336	0	0.198804	0
TCGA-B4-53	0.0283289	0	0.0039157	0.3036597	0	0.1201515	0
TCGA-B4-53	0.0868187	0	0.0117934	0.1527427	0	0.0829505	0
TCGA-B4-58	0.0414859	0	9.48E-05	0	0	0.3710482	0.031595
TCGA-B4-58	0.0602931	0	0.0041153	0.1372427	0	0.1338224	0.0281727
TCGA-B4-58	0.0217252	0	0	0.12225	0	0.1749462	0.0001153
TCGA-B4-58	0.0463378	0	0.0071267	0.198765	0	0.1172999	0.0251906

TCGA-B4-58	0.0626506	0	0.0047227	0.2094196	0	0.1548501	0.0051368
TCGA-B4-58	0.0055489	0	0	0.0793469	0	0.1916863	0
TCGA-B4-58	0.0577037	0	0.0090205	0.0694412	0	0.123732	0.0009707
TCGA-B8-41	0.0243745	0	0.0632532	0.4160762	0	0	0.1077066
TCGA-B8-41	0.0037791	0	0.0039427	0.1108926	0	0.219088	0.0206158
TCGA-B8-41	0.0342176	0	0.0130124	0.5821868	0	0.0174939	0.0123203
TCGA-B8-41	0	0.0100101	0.0039375	0.1204832	0	0.157996	0
TCGA-B8-41	0	0	0.0030335	0.2575334	0	0.1660575	0.0224604
TCGA-B8-41	0.0083337	0	0.0002506	0.3425405	0	0	0.0123927
TCGA-B8-46	0.3353396	0	0.0569616	0.0175022	0	0.167057	0
TCGA-B8-46	0.0273606	0	0.0021666	0	0	0.2364622	0.0131102
TCGA-B8-46	0.0326478	0	0	0.0087478	0	0.1134703	0.000897
TCGA-B8-46	0.0098449	0	0	0.0864816	0	0.1995419	0.0004331
TCGA-B8-51	0.0569113	0	0.0098966	0.1023619	0	0.2406147	0.0050753
TCGA-B8-51	0.0115053	0	0	0.4886931	0	0.0483606	0.0024466
TCGA-B8-51	0	0	0.0982871	0.3053722	0	0.0829807	0.017252
TCGA-B8-51	0.0214042	0	0.0075114	0.2552592	0	0.1718358	0.012713
TCGA-B8-51	0.0131618	0	0.0017923	0.5418019	0	0.0332902	0.0161586
TCGA-B8-51	0.2000055	0	0.0300297	0.0653328	0	0.2944681	0
TCGA-B8-55	0.0834655	0	0.0363098	0.1111061	0	0.1142258	0
TCGA-B8-55	0.0827458	0	0.0019783	0.2029141	0	0.294701	0.0131799
TCGA-B8-55	0.0266487	0	0.0033334	0.4549637	0	0.097176	0
TCGA-B8-55	0.0367831	0	2.62E-05	0.1517908	0	0.0786341	0.0351125
TCGA-B8-55	0	0.0042927	0.0363442	0.2556191	0	0.1232963	0.0371147
TCGA-B8-55	0.0873536	0	0	0.135688	0	0.1725109	0
TCGA-B8-55	0.117416	0	0.0242948	0.2169981	0	0.230025	0.0128918
TCGA-B8-A5	0.0101466	0	0.0032286	0.53717	0	0	0
TCGA-B8-A5	0.0258226	0	0.0150311	0.0546121	0	0.173854	0
TCGA-B8-A5	0.0829563	0	0.0165265	0.0627768	0	0.1608937	0
TCGA-B8-A5	0	0	0.0018166	0.4572883	0	0	0
TCGA-B8-A5	0.026027	0	0.0068333	0.5207368	0	0.0082693	0
TCGA-B8-A5	0.0086201	0.0516314	0.2059973	0.1070072	0	0.118359	0.0838711
TCGA-B8-A5	0.022629	0	0.0071925	0.370733	0	0.1246795	8.42E-05
TCGA-B8-A7	0.192538	0	0.0517891	0.0596522	0	0.1580892	0.0195525
TCGA-B8-A8	0.1172267	0	0.0423912	0.0782207	0.0290378	0.1854193	0.0246672
TCGA-BP-41	0.0057771	0	0.0003712	0.1009812	0	0.181264	0
TCGA-BP-41	0.0112372	0	0.0033689	0.0943491	0	0.1694132	0
TCGA-BP-41	0.022833	0	0.0036735	0.525826	0	0	0.0105961
TCGA-BP-41	0.0415274	0	0.0079608	0.3007574	0	0.1322552	0.0108114
TCGA-BP-41	0.0089123	0	0.0021885	0.4406872	0	0.0215101	0
TCGA-BP-41	0.0411307	0	0.0060957	0.1077735	0	0.1796324	0
TCGA-BP-41	0	0	0.0045478	0.1667175	0	0.2202797	0
TCGA-BP-41	0.0173536	0	0.0111445	0.2984637	0	0.1077177	0
TCGA-BP-41	0.0845379	0	0.038806	0.1874988	0	0.1681877	0.0275378
TCGA-BP-41	0	0	0.0115821	0.3396003	0	0.0818996	0.0211797
TCGA-BP-41	0.073689	0	0.0088897	0.001304	0	0.3163478	0
TCGA-BP-41	0.0262714	0	0.0048941	0.2197043	0	0.1767264	0
TCGA-BP-41	0.0152005	0	0.0078571	0.429701	0	0.1049751	0.0191652
TCGA-BP-41	0.0083363	0	0.0003885	0.0851337	0	0.1851616	0.0074997
TCGA-BP-41	0.0341381	0	0.0197468	0.0771558	0	0.2293409	0.0057352
TCGA-BP-41	0.2120089	0	0.0206854	0.1064114	0	0.2382686	0

TCGA-BP-43	0.0109971	0	0.0040142	0.4889276	0	0.0300226	0
TCGA-BP-43	0.0407543	0	0.0093296	0	0	0.2368087	0.0213886
TCGA-BP-43	0.0165792	0	0.0153635	0.219385	0	0.1387997	0
TCGA-BP-43	0.0163498	0	0	0.1190202	0	0.1952331	0
TCGA-BP-43	0.0692893	0	0.1141069	0.4184233	0	0.0313562	0.0040064
TCGA-BP-43	0.0079221	0	0.0032374	0.1304942	0	0.0715492	0
TCGA-BP-43	0	0.0293793	0.0121657	0	0	0.1339838	0
TCGA-BP-43	0.1369044	0	0.1714157	0.2500205	0	0.0631886	0.0046834
TCGA-BP-43	0.0634731	0	0.0154815	0.149796	0	0.2087692	0.0125852
TCGA-BP-43	0.022951	0	0.0059588	0.0579907	0	0.2269087	0
TCGA-BP-43	0.0294907	0	0.0360944	0.010068	0	0.2849159	0
TCGA-BP-43	0.0123497	0	0.0015232	0.0675873	0	0.2557549	0
TCGA-BP-43	0.0390846	0	0.0063577	0.1016904	0	0.189484	0.0181874
TCGA-BP-43	0.0115718	0	0.0033552	0.0553114	0	0.158889	0
TCGA-BP-43	0	0	0.0086069	0.6141671	0	0	0
TCGA-BP-43	0.0126043	0	0.0045196	0.2296005	0	0.226659	0.010018
TCGA-BP-43	0.0259646	0	0.0023985	0.1461868	0	0.3380784	0
TCGA-BP-43	0.0031065	0	0.0033237	0.1907769	0	0.249798	0
TCGA-BP-43	0.0026202	0	0.0303815	0.0635704	0	0.2036698	0
TCGA-BP-43	0.0072352	0	0.0090745	0.0462351	0	0.0929947	0.0159592
TCGA-BP-43	0	0.055408	0.020137	0.0815156	0	0.1407815	0
TCGA-BP-47	0.0097931	0.0125896	0.0076887	0.2582825	0	0.0782737	0
TCGA-BP-47	0.0342563	0	0.0063409	0.5066393	0	0	0
TCGA-BP-47	0.0162735	0	0	0.2673882	0	0.1561644	0
TCGA-BP-47	0.0665273	0	0.0046405	0.0305508	0	0.0827039	0
TCGA-BP-47	0	0	0.1321433	0.3443364	0	0.0145988	0.0476614
TCGA-BP-47	0.0853269	0	0.0284325	0.1428284	0	0.1149311	0.0010582
TCGA-BP-47	0.0266359	0	0	0.1735225	0	0.1766718	0.0247686
TCGA-BP-47	0.0141083	0	0.009927	0.0412003	0	0.2264177	0
TCGA-BP-47	0.022087	0	0.0060972	0.0274052	0	0.1055405	0
TCGA-BP-47	0	0	0.0029797	0.1964616	0	0.2089398	0
TCGA-BP-47	0.045098	0	0.0113398	0.0771402	0	0.3401897	0
TCGA-BP-47	0.0081776	0	1.05E-05	0.2232175	0	0.0629778	0.0188508
TCGA-BP-47	0.0005329	0	0.0007472	0.5910892	0	0	0
TCGA-BP-47	0.0505053	0	0.0050453	0.1805359	0	0.1672893	0
TCGA-BP-47	0.0463038	0	0	0.0749335	0	0.32317	0
TCGA-BP-47	0.040976	0	0.1023885	0.3094149	0	0.0014713	0
TCGA-BP-47	0.0489254	0	0.0133333	0.4355776	0	0.0263039	0.0309276
TCGA-BP-47	0.0185461	0	0.0010015	0.0473745	0	0.085292	0
TCGA-BP-47	0.0051277	0	0.0038886	0.2675202	0	0.0684064	0.0182085
TCGA-BP-47	0.1132119	0	0.0105565	0.0048067	0	0.2063409	0
TCGA-BP-47	0.0119013	0	0.0017509	0.0521459	0	0.1777557	0
TCGA-BP-47	0.0404475	0	0	0.1425781	0	0.1786915	0
TCGA-BP-47	0.0272193	0	0.0039004	0.2790901	0	0.212951	0.0177712
TCGA-BP-47	0.0907018	0	0.014102	0.1108073	0	0.1705893	0
TCGA-BP-47	0.0210198	0	0.0033972	0.2163163	0	0.1826288	0
TCGA-BP-47	0	0	0.0546253	0.4843069	0	0	0
TCGA-BP-47	0.044161	0	0.0581321	0.3012633	0	0.1126381	0.0560531
TCGA-BP-48	0.0185433	0	0	0.1254238	0	0.0757185	0
TCGA-BP-48	0.0371818	0	0.0041513	0.1894728	0	0.1705443	0
TCGA-BP-48	0	0.0031839	0.0026645	0.3396627	0	0.0339786	0.0180128

TCGA-BP-48	0.0234102	0	0	0.0547136	0	0.1040362	0
TCGA-BP-49	0.0642878	0	0.0080367	0.1293767	0	0.1956208	0
TCGA-BP-49	0.0210938	0	0.0018552	0.1773916	0	0.1040905	0
TCGA-BP-49	0.0201552	0	0	0.1172801	0	0.2157188	0.036536
TCGA-BP-49	0.0180346	0	0.0144138	0.3375329	0	0.0856126	0.0088538
TCGA-BP-49	0.0354995	0	0.0044986	0.141863	0	0.2056258	0
TCGA-BP-49	0.0196167	0	0.003875	0.2058776	0	0.2012213	0
TCGA-BP-49	0.0338495	0	0.0019851	0.049703	0	0.2780074	0
TCGA-BP-49	0.0281994	0	0.0077048	0.2025688	0	0.1675197	0
TCGA-BP-49	0.0158296	0	0.0082592	0.4389205	0	0.0871281	0.0083641
TCGA-BP-49	0.0698514	0	0.0333925	0.1097063	0	0.1439608	0
TCGA-BP-49	0.0176896	0	0.0044582	0.2970606	0	0.1482001	0.0012051
TCGA-BP-49	0	0.0074496	0	0.0716923	0	0.247983	0
TCGA-BP-49	0.0127704	0	0.0019545	0.0528841	0	0.138129	0
TCGA-BP-49	0.0179938	0	0	0.2983163	0	0.1892083	0
TCGA-BP-49	0.0573712	0	0.0091436	0.156167	0	0.3196811	0
TCGA-BP-49	0.1049389	0	0.0235177	0.0912756	0	0.1740198	0
TCGA-BP-49	0.0088464	0	0.0008229	0.1282156	0	0.2402834	0
TCGA-BP-49	0.0030473	0	0.0007271	0.2061396	0	0.2210431	0
TCGA-BP-49	0.0948865	0	0.0140813	0.1467206	0	0.1869853	0.0026224
TCGA-BP-49	0.010122	0	0.0107696	0.3993242	0	0.0346201	0.0324927
TCGA-BP-49	0.048262	0	0.0032233	0.0360264	0	0.2230185	0
TCGA-BP-49	0.0418743	0	0.0045912	0.3363091	0	0.0898517	0.0008311
TCGA-BP-49	0.0717044	0	0.0183336	0.4418733	0	0	0.0244762
TCGA-BP-49	0.0013043	0	0.0012016	0.0713707	0	0.1725648	0
TCGA-BP-49	0.0888924	0	0.020115	0.3126964	0	0.2134521	0.0166834
TCGA-BP-49	0.0441578	0	0.007644	0.4083213	0	0.0901532	0.0129478
TCGA-BP-49	0.0577112	0	0.0137986	0.2306365	0	0.079558	0
TCGA-BP-49	0.0193578	0	0.0045981	0.2228484	0	0.2153845	0
TCGA-BP-49	0.0336999	0	0.0006616	0.0772966	0	0.2396193	0
TCGA-BP-50	0.0080837	0	0.0070428	0.3173584	0	0.1580074	0
TCGA-BP-50	0.0008001	0	0.0085473	0.3881808	0	0.0991619	0.0232481
TCGA-BP-50	0.0090983	0	0.0002727	0.0175696	0.0065008	0.0525775	0.01599
TCGA-BP-50	0.0064644	0	0.006525	0.4056279	0	0.0632152	0.0032806
TCGA-BP-50	0.0533274	0	0.0095671	0.1988465	0	0.3088052	0
TCGA-BP-50	0.0520362	0	0.0138233	0.2826864	0	0.1091361	0.0072354
TCGA-BP-51	0.0181337	0	0	0.1520113	0	0.2204583	0
TCGA-BP-51	0.0417747	0	0.326332	0.1532575	0	0.0629556	0.0014257
TCGA-BP-51	0.0769133	0	0.0182481	0.0545013	0	0.2597053	0
TCGA-BP-51	0.0167981	0	0.0010208	0.5071613	0	0	0.0001416
TCGA-BP-51	0	0.0098588	0.0047046	0.3138967	0	0.0315187	0
TCGA-BP-51	0.0022286	0	0.0012495	0.2836133	0	0	0
TCGA-BP-51	0.04256	0	0	0.2354952	0	0.1078803	0.029484
TCGA-BP-51	0.0348329	0	0.0050194	0.2157577	0	0.145708	0
TCGA-BP-51	0.1357233	0	0.0192808	0.1141541	0	0.1319316	0.0119605
TCGA-BP-51	0.0034841	0.0053529	0.0053974	0.1784273	0	0.2258699	0.0003791
TCGA-BP-51	0.0130557	0	0.0031335	0.1565335	0	0.158359	0.003899
TCGA-BP-51	0.0167869	0	0.002152	0.3989733	0	0.0702447	0.0101128
TCGA-BP-51	0.0357094	0	0	0.2604714	0	0.1607329	0.0166737
TCGA-BP-51	0.0385603	0	0.0067753	0.388212	0	0.0757034	0
TCGA-BP-51	0.1425998	0	0.0643897	0.213664	0	0.0753542	0

TCGA-BP-51	0.0944009	0	0.008062	0.2826089	0	0.2143691	0.0156712
TCGA-BP-51	0.0368426	0	0.0097906	0.0850044	0	0.228666	0.0247337
TCGA-BP-51	0.0004253	0	0.0087095	0.2021726	0	0.1624203	0
TCGA-BP-51	0.0111361	0.0008658	0.0333068	0.1298489	0	0.3130871	0.0187975
TCGA-BP-51	0.043538	0	0.0142236	0.179911	0	0.1815265	0.0034989
TCGA-BP-51	0.1205939	0	0.0347948	0.1240646	0	0.1241846	0.0102631
TCGA-BP-51	0.0204174	0	0.005534	0.1509022	0	0.1960965	0
TCGA-BP-51	0.0204362	0	0.0058691	0.4543815	0	0	0
TCGA-BP-51	0.1496716	0	0.0446707	0.0727054	0	0.1880264	0.0119217
TCGA-BP-52	0.0577242	0	0.0116328	0.2954408	0	0.1253296	0
TCGA-BP-52	0.0333353	0	0.0092061	0.4422982	0	0	0.0312153
TCGA-BP-52	0.0145109	0	0.0030178	0.1397382	0	0.1713547	0
TCGA-CJ-46	0.0332907	0	0.0045527	0.1075117	0	0.1556356	0
TCGA-CJ-46	0.0029889	0	0.0005858	0.5075934	0	0	0.0124873
TCGA-CJ-46	0.0288865	0	0.0040313	0.3636003	0	0.0729502	0
TCGA-CJ-46	0	0.004017	0.0370616	0.4625671	0	0	0.0210633
TCGA-CJ-46	0.0145194	0	0.0470665	0.3286175	0	0.0829095	0
TCGA-CJ-46	0.0203271	0	0.0087314	0.5203807	0	0.1021005	0
TCGA-CJ-46	0.0122351	0	0.0018589	0.3376715	0	0.1182269	0.000182
TCGA-CJ-46	0.038754	0	0.0167905	0.328225	0	0.1158514	0
TCGA-CJ-46	0.0598292	0	0.0127054	0.1155594	0	0.2551789	0
TCGA-CJ-46	0	0	0.0078748	0.4218335	0	0.0279875	0.0266229
TCGA-CJ-48	0.0285764	0	0.0059391	0.2734403	0	0.0949392	0.0326765
TCGA-CJ-48	0.0139657	0	0.0053278	0.5483565	0	0.067816	0.0110331
TCGA-CJ-48	0.0347316	0	0.0074886	0.0538284	0	0.247924	0
TCGA-CJ-48	0.0157591	0	0	0.2063995	0	0.1150144	0.0062612
TCGA-CJ-48	0.1549388	0	0.0321013	0.1123274	0	0.3177916	0.0202599
TCGA-CJ-48	0.0099851	0	0.0435956	0.2073301	0	0.1263845	0.0347907
TCGA-CJ-48	0.0063307	0	0.0007136	0.4662678	0	0.0828867	0.0009604
TCGA-CJ-48	0	0	0.0035714	0.7058399	0	0	0
TCGA-CJ-48	0	0	0.029132	0.6258954	0	0	0.0731604
TCGA-CJ-48	0.0028265	0	0.0400524	0	0	0.3246151	0
TCGA-CJ-48	0.0150347	0	0.0280171	0.1849698	0	0.1409504	0.0089285
TCGA-CJ-48	0.1516311	0	0.0484379	0.1984826	0	0.306375	0.0301255
TCGA-CJ-48	0.0230273	0	0.0047545	0.2157656	0	0.2323153	0
TCGA-CJ-48	0.0515603	0	0	0.2219434	0	0.192873	0
TCGA-CJ-48	0.0244562	0	6.87E-05	0.2945552	0	0.1480174	0
TCGA-CJ-48	0.0080382	0	0.0033774	0.4640171	0	0	0.0069478
TCGA-CJ-48	0.0001919	0	0.0184974	0.4879161	0	0	0.0616802
TCGA-CJ-48	0.0393802	0.1000555	0.0982714	0.1689368	0	0.1993188	0
TCGA-CJ-48	0.097781	0	0.0648839	0.2446412	0	0.1427725	0.0218587
TCGA-CJ-48	0.0391023	0	0.0123153	0.0978305	0	0.0564845	0.0057481
TCGA-CJ-48	8.60E-06	0	0.0051308	0.647333	0	0	0
TCGA-CJ-48	0.0304238	0	0.0034807	0.4524153	0	0.1335686	0.0267226
TCGA-CJ-48	0.0207923	0	0.0359965	0.5352093	0	0	0
TCGA-CJ-48	0.0769766	0	0.0105909	0.2410295	0	0.0666155	0.009238
TCGA-CJ-48	0.0746489	0	0.0307615	0.2560334	0	0.1277574	0
TCGA-CJ-48	0.0523272	0	0.0092259	0.1434693	0	0.1649967	0.0087885
TCGA-CJ-49	0.0560267	0	0.1323065	0.3501548	0	0.038322	0.0432361
TCGA-CJ-49	0.015603	0	0.0177068	0.3561983	0	0.0153309	0.0075777
TCGA-CJ-49	0.0196928	0	0	0.1708128	0	0.1499196	0.0156536

TCGA-CJ-49	0.0378036	0	0.0118505	0.3299009	0	0.2037128	0
TCGA-CJ-49	0.0335374	0	0.0028779	0.2086267	0	0.1752917	0.00669
TCGA-CJ-49	0.0296741	0	0	0.1714302	0	0.1617579	0.0185757
TCGA-CJ-49	0.051592	0	0.0062407	0.1209108	0	0.1307904	0
TCGA-CJ-49	0.0399365	0	0.0082683	0.2768218	0	0.0949473	0
TCGA-CJ-49	0.0018102	0	0.1068784	0.179107	0	0.0873364	0
TCGA-CJ-49	0	0	0.0081742	0.547275	0	0	0
TCGA-CJ-49	0.1570338	0	0.0261888	0.1461718	0	0.1620099	0.0039687
TCGA-CJ-49	0.0283769	0	0.0043494	0.0750325	0	0.2305808	0
TCGA-CJ-56	0.0385394	0	0.0112492	0.1351289	0	0.1971218	0.0287073
TCGA-CJ-56	0	0	0.0139924	0.5346532	0	0	0
TCGA-CJ-56	0.0072161	0	0.0056699	0.6141429	0	0	0.0118413
TCGA-CJ-56	0.0282764	0	0.0606773	0.2373608	0	0.1488623	0
TCGA-CJ-56	0.0624925	0	0.0063709	0.0913587	0	0.0449949	0
TCGA-CJ-56	0.0191519	0	0.0853929	0.2923249	0	0.1810332	0.0304945
TCGA-CJ-56	0	0.0001514	0.0030874	0.1171966	0	0.0987261	0.0001447
TCGA-CJ-56	0	0.0104079	0.0011207	0.1423702	0	0.1362456	0
TCGA-CJ-56	0.018077	0	0	0.0276726	0	0.0613743	0
TCGA-CJ-56	0.1269149	0	0.0321894	0.2593712	0	0.1436909	0
TCGA-CJ-56	0	0	0.0038512	0.2364292	0	0.0819349	0
TCGA-CJ-56	0.0282213	0	0.0108567	0.4135253	0	0.0633844	0.0115066
TCGA-CJ-56	0.01469	0	0.0043385	0.202855	0	0.2401453	0.0431368
TCGA-CJ-56	0.0536521	0	0.0062761	0.2769551	0	0.0373405	0.013847
TCGA-CJ-60	0	0	0.0107393	0.3432535	0	0	0.061577
TCGA-CJ-60	0.0434372	0	0.0255734	0.3425848	0	0.0906298	0.0341749
TCGA-CJ-60	0.0572559	0	0.0141486	0.1539236	0	0.2133977	0.004344
TCGA-CJ-60	0.045638	0	0.0358009	0.277743	0	0.0342557	0.0228245
TCGA-CJ-60	0	0	0.005666	0.352234	0	0.1021416	0
TCGA-CJ-60	0.0321943	0	0.003382	0.1893909	0	0.0043241	0
TCGA-CW-55	0.0112058	0	0	0.0298488	0	0.1380757	0
TCGA-CW-55	0.0289951	0	0	0.1865958	0	0.3315863	0.0225586
TCGA-CW-55	0.0798591	0	0.0099571	0.149486	0	0.0936869	0
TCGA-CW-55	0.0563496	0	0.0065432	0.028153	0	0.2248989	0
TCGA-CW-55	0.0102222	0	0	0.0157083	0	0.2908257	0
TCGA-CW-55	0.0045628	0	0.000527	0.4507868	0	0	0
TCGA-CW-55	0.0356876	0	0	0.109138	0	0.1594231	0.0018101
TCGA-CW-55	0.0765006	0	0.017133	0.0922591	0	0.1331732	0.01012
TCGA-CW-55	0.0079594	0	0	0.1827537	0	0.2243801	0
TCGA-CW-55	0.070085	0	0	0.0910352	0	0.0810123	0
TCGA-CW-60	0.0202044	0	0.0109879	0.4347299	0	0	0.0417996
TCGA-CW-60	0.046924	0	0.0064776	0.1103127	0	0.1247782	0.0148756
TCGA-CW-60	0.0452384	0	0.0193148	0.1687985	0	0.1492239	0.0221771
TCGA-CW-60	0.0231071	0	0.0007125	0.0686845	0	0.1131868	0.0134497
TCGA-CW-60	0.1055963	0	0.0355586	0.1547538	0	0.3729126	0.0157908
TCGA-CZ-48	0.034682	0	0.0085491	0.1049818	0	0.2103499	0.0036165
TCGA-CZ-48	0.0373161	0	0.00861	0.1470544	0	0.1617403	0
TCGA-CZ-48	0.0552599	0	0.0129363	0.2831979	0	0.1268825	0
TCGA-CZ-48	0.0208251	0	0.0037965	0.111404	0	0.2384416	0.004558
TCGA-CZ-48	0.0888513	0	0.0148701	0.3738584	0	0.0147699	0.0867891
TCGA-CZ-48	0.0873891	0	0.009049	0.196499	0	0.106781	0.0282035
TCGA-CZ-48	0	0	0.0069914	0.145159	0	0.2651796	0.0192629

TCGA-CZ-48	0.0049448	0	0.0025185	0.053507	0	0.2486106	0.0346931
TCGA-CZ-48	0.0084814	0	0	0.4970028	0	0.0111122	0.0014725
TCGA-CZ-48	0.0059811	0	0.0035608	0.5769395	0	0	0
TCGA-CZ-48	0.0017051	0	0.0047373	0.432405	0	0.0219909	0
TCGA-CZ-48	0.0248594	0	0.0104539	0.0731944	0	0.1253015	0
TCGA-CZ-54	0.0316836	0	0.0042319	0.1878867	0	0.2356154	0
TCGA-CZ-54	0.0057642	0	0.0261807	0.5353311	0	0.0045362	0
TCGA-CZ-54	0.0055923	0	0	0	0	0.2527268	0
TCGA-CZ-54	0.008307	0	0	0.109124	0	0.1110325	0.0066436
TCGA-CZ-54	0.0180052	0	0.0018257	0.4406027	0	0	0.0054702
TCGA-CZ-54	0.0338152	0	0.0287871	0.1411916	0	0.2142674	0
TCGA-CZ-54	0.0580028	0	0.0525606	0.2349377	0	0.0590766	0
TCGA-CZ-54	0.0372408	0	0.0022846	0.2124452	0	0.3466115	0.0138539
TCGA-CZ-54	0.0217985	0	0.001655	0.3100094	0	0.1723068	0.0038063
TCGA-CZ-54	0.0451852	0	0.0175252	0.3130772	0	0.1297611	0
TCGA-CZ-54	0.0454363	0	0.0052535	0.0857786	0	0.2651977	0.0105349
TCGA-CZ-54	0.0434205	0	0.0062647	0.0306961	0	0.2081713	0
TCGA-CZ-54	0.0046538	0	0.0040307	0.2448788	0	0.1230082	0
TCGA-CZ-54	0.005992	0	0.0135398	0.2190612	0	0.0501714	0
TCGA-CZ-54	0.0858647	0	0.0148446	0.0812895	0	0.1783742	0
TCGA-CZ-54	0.0095229	0.017917	0.0014535	0.1909482	0	0.1106422	0
TCGA-CZ-54	0.0343188	0	0.007414	0.1312195	0	0.1796932	0
TCGA-CZ-54	3.18E-05	0	0.0009002	0.1811016	0	0.1110856	0
TCGA-CZ-54	0.0028249	0	0.000681	0.1631416	0	0.1911917	0
TCGA-CZ-54	0.0163823	0	0.0345764	0.2924915	0	0.1272712	0.0194639
TCGA-CZ-59	0.0299401	0	0.0046562	0.1043476	0	0.2641715	0
TCGA-CZ-59	0.119964	0	0.0860378	0.0731173	0	0.3144215	0.0094713
TCGA-CZ-59	0.0468221	0	0.0445573	0.1431096	0	0.1641609	0.013337
TCGA-CZ-59	0.0323594	0	0	0.0531021	0	0.2036123	0.0228184
TCGA-CZ-59	0.004891	0.0059924	0.0033737	0.1865606	0	0.1845485	0.0471898
TCGA-CZ-59	0.0627645	0	0.0083734	0.2291799	0	0.2036212	0.0171854
TCGA-CZ-59	0.0150461	0	0	0.065143	0	0.2898357	0.0443086
TCGA-DV-55	0.0246432	0	0.0018821	0.168143	0	0.06333	0
TCGA-DV-55	0.0419559	0	0	0.1547049	0	0.1917465	0.0005032
TCGA-DV-55	0.010096	0	0.0017203	0	0	0.2280962	0
TCGA-DV-55	0.0601642	0	0.000798	0.2281175	0	0.1427207	0.0068098
TCGA-DV-55	0.1117109	0	0.1127603	0.2392921	0	0.0083876	0.0338856
TCGA-DV-55	0.0434775	0	0.0059239	0.2535465	0	0.2706362	0.0044679
TCGA-DV-55	0.000851	0.0117065	0.0073198	0.0159109	0	0.2606237	0
TCGA-DV-A4	0.005452	0	0.0072016	0.375052	0	0.0012317	0.0126936
TCGA-DV-A4	0.1285406	0	0.0247108	0.0097368	0	0.2490969	0
TCGA-EU-59	0.0174837	0	0	0.0716201	0	0.3263312	0
TCGA-EU-59	0	0	0	0.0976066	0	0.2139217	0
TCGA-EU-59	0.0490333	0	0.0044573	0.0712003	0	0.2279387	0.0256941
TCGA-EU-59	0	0.0105735	0	0.0699481	0	0.1910782	0
TCGA-G6-A5	0.0257166	0	0.0243642	0.5529783	0	0.0126697	0
TCGA-G6-A8	0.03543	0	0.0142056	0.1518018	0	0.222882	0
TCGA-G6-A8	0	0	0.0028289	0.3130169	0	0	0.04041
TCGA-G6-A8	0.0138634	0	0.0058631	0.0840273	0	0.1833147	0
TCGA-GK-A6	0.0337383	0	0.0091436	0.1891719	0	0.126446	0
TCGA-MM-A5	0.0303531	0	0.0615235	0.1715891	0	0.0904502	0

TCGA-MM-A5	0	0.0008482	0.0045774	0.0857727		0	0.0913428		0
TCGA-MM-A8	0.0505544	0	0.0304197	0.119836		0	0.1848423		0
TCGA-MW-A4	0.0053738	0.006509	0.0019482	0.1503633		0	0.1445003		0
TCGA-T7-A9	0.0140586	0	0.0283773	0.1417748		0	0.2534146		0

T cells fc	T cells re	T cells ga	NK cells r	NK cells a	Monocytes	Macrophage	Macrophage
0	0.0120668	0	0	0.1200969	0.0856971	0.0086823	0.141649
0.022444	0.034992	0	0	0.0434118	0.036217	0	0.0136462
0.0046016	0	0.0002142	0	0.029615	0.1323258	0	0.0895805
0	0.0155459	0.0112847	0	0	0.1455194	0	0.1467377
0.0051512	0.0269416	0	0.0296011	0	0.0791445	0.050615	0.1165097
0.0325566	0.020926	0.022444	0	0	0.14347	0	0.0779094
0.004777	0.0011415	0	0.0691993	0.0139277	0.0879363	0.0142202	0.0464658
0.0132913	0.0081976	0.0043462	0	0.0281394	0.1134404	0.1568996	0.0439147
0.0128522	0.0080903	0	0	0.0244994	0.0278066	0.0235912	0.1676572
0.021667	0.0245148	0	0	0.0164572	0.1734447	0	0.1016998
0	0	0	0.0005118	0.0050885	0.047139	0	0.136541
0.0024059	0.0191479	0	0.0220671	0.0129149	0.0500886	0	0.0456435
0	0.0091179	0	0.009715	0.0109282	0.1198386	0	0.1172136
0.0086656	0.0372849	0.0217075	0	0.0142428	0.0902528	0	0.0952141
0	0.0048747	0	0.0177909	0	0.0495775	0	0.0418863
0	0.0245776	0	0.0253996	0	0.0449057	0.0068957	0.1082638
0.0167997	0.0327283	0	0.1156201	0.0225433	0.1506366	0.2155471	0
0	0.0305028	0	0	0.0065262	0.0645231	0	0.1949304
0.035944	0.0003017	0	0	0.0583239	0.0739334	0	0.1121112
0	0.0044391	0.0012627	0	0	0.0512779	0.0584251	0.0541391
5.58E-06	0	0	0.0463375	0.0286035	0.0769851	0.0250284	0.1390245
0.013256	0.0227264	0	0.0457653	0	0.0351667	0.1344253	0.09413
0.0161144	0.027815	0.0223298	0	0	0	0.2050689	0.0715738
0	0.0107677	0	0.0693108	0.0228738	0.0585317	0	0.0196038
0.0172497	0.0240781	0.0069495	0	0.0138562	0.1372643	0	0.0743916
0	0.0155727	0	0.0932601	0.0206445	0.1002686	0	0.141453
0.0239786	0.0203767	0.0443206	0	0.0302349	0.028674	0	0.0290649
0.0300761	0.0212805	0.0160493	0	0.0215351	0.041583	0	0.0546461
0.0093286	0.0106767	0	0	0.0025531	0.0521705	0	0.1635456
0	0.0088988	0	0.0122604	0	0.0902981	0	0.0999339
0	0.0134391	0	0	0.0317787	0.0546524	0.0023423	0.0092476
0	0	0	0.0739437	0	0.0128238	0	0.1051341
0	0	0	0.03918	0	0.0280534	0	0.109815
0.0094509	0.0192768	0.028101	0	0.03121	0.0277619	0	0.1000354
0.0085906	0.0107387	0.0050799	0	0.0298105	0.0937283	0.0026103	0.1911991
0	0.0110992	0	0.0347085	0	0.0261035	0.0272883	0.124057
0.0036918	0.0081107	0.0083193	0.0040693	0.0026706	0	0.7226433	0.0050357
0	0.0071767	0	0.0727191	0	0.2185445	0	0.0249715
0.028773	0.0192677	0.0055227	0	0	0.0058888	0	0.0821208
0	0	0	0.0866615	0	0.0204676	0.0142974	0.0649235
0.0069784	0.0016008	0	0	0.0194807	0.0025873	0.025286	0.0866623
0.0348923	0.0603185	0	0	0.0433503	0.0382573	0	0.1085969
0	0.0162104	0.008587	0	0.0061074	0.0490806	0.0248172	0.0471297
0	0.0121708	0	0.0217994	0.0193275	0.0963898	0	0.0169183
0.0036846	0.0122177	0	0.0374137	0.0454107	0.122359	0	0.0547649
0.0235758	0.0402914	0	0	0.0164651	0.0735241	0	0.116224
0.0142197	0.0329598	0	0.0114862	0.0870714	0.0684834	0.0165477	0.0876662
0	0.0007201	0	0.1656471	0.0188821	0.0650095	0	0.0881585
0.002423	0.0399686	0	0.0765601	0.0141085	0.1447495	0	0.0671134

0.0075864	0.0384484	0	0.1232864	0	0.1441498	0	0.0431817
0	0.0004814	0	0.0404235	0.097015	0.1921607	0	0.0761512
0.0352258	0.047525	0.0195472	0	0.0258899	0.0422488	0	0.0494182
0.0303392	0.03557	0	0	0.0248632	0.0223221	0.0241562	0.1179103
0.0390154	0.0629664	0	0	0.0316678	0.0229077	0.0211249	0.0998065
0.003716	0.0194317	0	0.0451165	0.0797514	0.1138789	0.0050962	0.0552846
0.0101238	0.0176724	0	0	0.0043093	0.3665461	0.0425437	0.0859367
0.0467974	0.0476491	0.0848768	0	0.0285064	0.0082588	0	0.1131198
0	0	0	0.1128435	0	0.0751486	0.1511221	0.0526438
0.0069417	0.0413321	0	0	0.0566515	0.0194657	0.034432	0.0560766
0.0249906	0.0198963	0.1769123	0	0	0	0	0.1140884
0.0041308	0.0183244	0	0.0204418	0	0	0.1278699	0.0375898
0.0214504	0.0549666	0	0.1290635	0	0.073779	0.0454679	0
0.0007217	0.0455831	0.0318028	0.0200663	0	0.0866055	0.2648813	0.0105689
0.0126317	0.0632212	0	0	0.0187513	0.0018673	0.008835	0.1065637
0.0305573	0	0.0186001	0.1238189	0	0.0602381	0	0.1178655
0.0028146	0	0	0	0.0945874	0.0698852	0.0084126	0.1115841
0.0244805	0.004314	0.0223638	0	0.024531	0.0452303	0.039765	0.049567
0.0346676	0.0715747	0.0212713	0	0.0644716	0.0340385	0	0.0676708
0.016665	0.0397514	0	0	0.0451384	0.0120533	0.0214973	0.054576
0.0371042	0.0366782	0.1203923	0	0.0341654	0	0	0.0550862
0	0.0123067	0	0.0222111	0	0.0260439	0.0946457	0.019793
0	0	0.1635983	0	0	0.0257177	0	0.0679843
0	0.0105434	0.003617	0.0494354	0	0.0718845	0	0.0644825
0	0	0.0087878	0.0913542	0.0009513	0.1309284	0	0.0687484
0.0040318	0	0	0.0696841	0.0089991	0.0439612	0.0147453	0.1345144
0	0	0.0323525	0	0.0152419	0.0125923	0.0721622	0.0938716
0	0.004665	0	0.0276203	0.0249196	0.0994871	0	0.1627751
0	0.012113	0	0.0099464	0.071067	0.1433879	0.023298	0.0192725
0.0218649	0.0346744	0.013711	0	0.0213852	0.1050141	0	0.1079115
0.0757465	0.045063	0.0576943	0	0.0624034	0.0226298	0	0.1119978
0	0.0225564	0.0110986	0	0.0101548	0.114017	0	0.1289093
0.0238896	0.0418785	0.0573926	0	0	0	0.1606708	0.062012
0	0	0	0.0207375	0.0199629	0.030244	0.3867558	0.0064462
0.0247709	0.0971541	0.0184557	0	0.0080666	0	0.0272348	0.0980051
0.0459985	0.0021153	0	0	0	0	0.0278467	0.0765583
0.0530556	0.0498179	0.0246251	0	0.0391664	0.0004963	0.0315822	0.0610682
0.0586103	0.0565753	0.039071	0	0.0161266	0	0.1264736	0.1254344
0	0.0322922	0	0	0.0077625	0.0321789	0.0091132	0.1083787
0.0549824	0.0297207	0	0	0	0.0364521	0.0378838	0.1172423
0	0.0300905	0	0.1874684	0.0539156	0.0428773	0.0096209	0.0821186
0.0350907	0.0955016	0	0.0030158	0.0167035	0.0184975	0.0194909	0.0791907
0.0143073	0.0225958	0	0.0702837	0	0.0789254	0.0693422	0.0417828
0.017032	0.0609311	0.0228307	0	0.0358977	0.0483299	0.1127119	0.1329336
0.0383304	0.0948995	0.0282222	0.0473524	0.0447383	0.0100293	0.0250214	0.1207137
0.0117926	0.0427519	0.0172666	0.0428293	0.0074887	0.0111794	0	0.1035667
0.0843095	0.0298481	0.0155209	0	0.0383103	0.0083042	0.0112938	0.1210615
0.0425826	0.0405928	0	0	0.0853292	0.0327312	0	0.0589259
0.0035138	0.0587151	0	0	0.0737918	0.0225223	0	0.1789911
0.0108463	0.0819056	0	0	0.0456776	0.0603877	0.1552017	0.0165728
0	0.0209985	0	0	0.0723267	0.0321903	0.0339531	0.0730042

0.0577198	0.0459158	0.0609889	0	0.005636	0	0.0074788	0.1021623
0.0019024	0.0017144	0	0.0511883	0.0150637	0.2557515	0	0.046524
0.0424248	0.0099419	0.021156	0	0.0461209	0	0.0530612	0.0356497
0.0008986	0.0192404	0.0138241	0	0.0791672	0.0362092	0.0007158	0.0802094
0.0490871	0.0398416	0.0440787	0	0.0406071	0	0.0229932	0.0709529
0.060499	0.0444577	0.0301617	0	0.0659	0	0.3329188	0.0588273
0.0270158	0.1055681	0	0	0.0777985	0.0189039	0	0.0008475
0.024241	0.0159392	0.0152616	0	0.0286926	0.010954	0	0.0493105
0.0265817	0.0433281	0	0	0.0720832	0.0264463	0	0.0933424
0.0247269	0.0155644	0	0	0.0515775	0.0317342	0	0.0646968
0.0406738	0.0533947	0.004344	0.0951605	0	0.0855273	0	0.0862064
0.055171	0.0189059	0	0	0.0555298	0.0185343	0.0125273	0.0773306
0	0	0	0.0585662	0.0306906	0.04979	0	0.0457677
0.0225596	0.0693051	0.004028	0	0	0	0.1396372	0.1275439
0.0051464	0.0765298	0	0	0.0378749	0	0.4559192	0.018544
0.0447628	0.0425927	0.015091	0	0.0517355	0.0058431	0	0.0563145
0.0081797	0.0112946	0	0.0233539	0.0914682	0.109148	0	0.0425005
0	0.0484361	0	0.0906142	0.0612914	0.0453532	0.005761	0.108035
0.0221315	0.1595873	0.0147303	0	0.0193225	0.0118956	0.1440251	0.0647042
0.0081671	0.0850495	0	0.05179	0.0112234	0.0412432	0.2534241	0.0422556
0.0259239	0.0383826	0.0123851	0	0.0031217	0.0479456	0.0414127	0.1203057
0	0.0004894	0	0.0006545	0.0183516	0.0613954	0.0612353	0.0845978
0.006971	0.0142922	0.0252874	0	0	0.0132155	0	0.1660794
0.0578252	0.0813601	0.0134545	0	0.051231	0.0369433	0	0.0608
0.0054713	0.0465968	0	0	0	0.0882764	0.053749	0.1179813
0.0217523	0.0500251	0	0.079092	0	0.1833075	0.0028224	0.1224892
0.0218173	0.0063134	0.0490797	0.0104259	0.0041821	0.1182088	0	0.1262992
0.018816	0.0068241	0	0.0296348	0	0.0458235	0	0.1934348
0.0375062	0.0672699	0	0	0.0365176	0.0178431	0.1464198	0.0369354
0.0430924	0.0520868	0	0.0043571	0.0588079	0.0034833	0.2616053	0.0799255
0.0116306	0.1225124	0	0	0.0429048	0.0112774	0.0070636	0.0651762
0.0903636	0.0548509	0.0486273	0	0	0.0214395	0.0328781	0.1161297
0.0095498	0.0096266	0.0112353	0	0	0.0275514	0.1969273	0.0566156
0.0279509	0.1056661	0	0	0.038586	0.0073585	0.2226263	0.0509237
0.0146113	0.0248631	0	0.0811965	0.0464976	0.0187927	0.0253287	0.1274617
0.0177081	0.0224574	0	0	0.0515701	0	0.1053795	0.059406
0.0547722	0.0793831	0.0184836	0	0.0584137	0.0012418	0	0.0753991
0	0.0081466	0.1025605	0.0059308	0.1078244	0.0021657	0.0725634	0.0524587
0.0151764	0.0178267	0	0	0.108653	0.0544627	0.0443845	0.1132097
0	0.0180364	0.0030975	0	0.0192158	0.0552877	0.2017986	0.0658132
0.0055578	0.0216492	0.0086425	0	0	0	0.331973	0.0380079
0.0239166	0.0003242	0.038047	0	0.007285	0.038811	0.3781644	0.1316122
0.0003306	0.0256195	0	0.1221847	0.009313	0	0.174626	0.0579351
0	0.0101687	0	0.0868701	0.0415828	0.0325517	0.2189498	0.0648865
0.0135568	0.0129449	0	0	0.0281821	0.0027292	0.0410151	0.1001594
0	0.0234617	0	0.0166503	0.004425	0.2280313	0	0.094634
0.0139424	0.0226927	0	0	0.0657489	0.0643031	0	0.0393194
0.0300062	0.1152317	0	0	0.0261311	0.0547755	0	0.1243841
0.0032734	0.0065675	0	0.0267381	0	0.0180852	0.2171781	0.0839143
0.0272941	0.0150968	0	0.0094514	0.0368561	0.0269734	0.0407494	0.0931638
0	0.0673352	0	0.0153999	0.0235478	0.0700926	0	0.1194597

0.0380052	0.0447582	0.0166795	0	0.0072269	0.0673276	0	0.088781
0.0029503	0.028151	0	0.0765337	0	0.0291982	0.0584858	0.0891977
0.0205805	0.056113	0.0332498	0	0.0487685	0.0088742	0.0095624	0.1058322
0.0634879	0.0024495	0.020443	0	0.1410474	0.025026	0	0.0874755
0.0173238	0.0315368	0	0	0.0374813	0.0740084	0	0.0672102
0.0207709	0.0197692	0.0135611	0	0.0633889	0.0341862	0.0102164	0.0566756
0	0.0156468	0	0.1564053	0	0.1234836	0.0037801	0.0650371
0	0.0724812	0.0221818	0	0.0970435	0.0146432	0	0.1114411
0.0204903	0.0451724	0.0503402	0	0.0736794	0	0.1675734	0.0474934
0	0	0.0783253	0	0.052493	0.0335011	0	0.2002036
0	0.0748296	0	0.0603973	0.0234467	0.109289	0.0747706	0.1070894
0	0.0220468	0.0022921	0	0.001714	0.0460217	0.0575181	0.1539793
0.0629988	0.0517091	0.0199748	0	0.0631505	0.0051288	0	0.0688549
0	0.0041747	0	0.0587716	0.0261683	0.0335712	0.002111	0.1014098
0	0.0197189	0	0.1027193	0.0258729	0.0601944	0.0795929	0.0870126
0.0022324	0.0582782	0	0.0728149	0	0.0448908	0	0.0982517
0.0045612	0.0181824	0	0	0.0320727	0.1149559	0	0.1169953
0.0200852	0.0481086	0.0152654	0	0.0580558	0.0132933	0	0.054674
0.0319785	0.0197177	0	0.0125631	0	0.0927533	0.0488675	0.1192442
0	0.0454455	0	0.0499946	0.0469094	0.0787906	0.0051286	0.1498574
0.0216636	0.0204863	0	0	0.0862565	0.0917061	0.0192598	0.1001721
0.031987	0.0519919	0.0258902	0	0.0086561	0.0305625	0.1267411	0.1084421
0	0	0	0.034937	0.03267	0.0482868	0	0.0179286
0	0.0114801	0	0.1512764	0.048406	0.0464789	0.0287201	0.0525307
0.0147913	0.0456245	0	0	0.0111151	0.0508519	0	0.0767825
0.0374233	0.0298907	0	0	0.0263149	0.0094644	0.0241786	0.0427718
0	0.0113433	0	0	0.0433866	0.0136823	0.1650973	0.0103138
0.0074017	0.0275677	0	0	0.0108842	0.084719	0	0.1028667
0.0448401	0.0194579	0.0633848	0.015647	0	0.0153325	0.1994308	0.1308609
0	0.0206648	0.0370562	0	0.0222265	0.0600822	0.0759666	0.0859329
0.068161	0.036731	0	0	0.0384131	0.0442115	0	0.0734092
0.0226941	0.0087767	0	0.0404434	0.0488561	0.0709266	0	0.2275842
0	0.0238191	0.0187715	0.0692505	0	0.0595568	0	0.1471883
0	0	0	0	0.0433022	0.1023702	0	0.090632
0.0445801	0.0204356	0.0914385	0	0.0137257	0.0574764	0	0.1030091
0.0289574	0.0167483	0.0052257	0	0.0060922	0	0.2818642	0.0441926
0.0143214	0.0134117	0.0250547	0.0114936	0.0380416	0.1218005	0	0.1372303
0	0.0144099	0.0041134	0.0125282	0.0316332	0.0552762	0	0.1021341
0	0.0178048	0	0.0215642	0.0132668	0.0383282	0	0.1494413
0.0025665	0	0.0795546	0	0.0044785	0.0212834	0.0639877	0.1116969
0.025475	0.0066521	0.0155049	0	0.0125981	0.1476018	0	0.0724637
0.0099152	0.0131266	0	0	0.052033	0.1562043	0	0.0628407
0.0282856	0.100123	0.0158808	0	0.0241585	0.1027609	0.0031192	0.0691112
0.0357511	0.067923	0.0731444	0	0.0340612	0.0524201	0.0032065	0.0612646
0.0119076	0.0202394	0.0009828	0.0334489	0.002251	0.0124985	0.0206062	0.1301764
0.0251774	0.0294969	0	0	0.0440284	0.1487108	0.0007725	0.0685347
0	0.002849	0	0.090179	0.033384	0.0483542	0	0.0258741
0	0	0.0597713	0	0.019185	0.0491007	0	0.0961413
0	0.0041061	0.0035479	0.0541088	0	0.042718	0	0.2032925
0.018261	0.0281811	0.0224114	0	0.0235637	0.0862156	0.063328	0.0611653
0	0	0	0.0975073	0.0096211	0.0722084	0.0059292	0.1324288

0.0082233	0.0110799	0.0151471	0	0.0027055	0.0137235	0.0621108	0.135016
0	0	0	0.0563778	0	0.0360082	0	0.0438983
0	0.0057851	0	0.0880658	0.0103809	0.178636	0.1893887	0.074971
0.018484	0.0430355	0	0	0	0.0124213	0.0322728	0.1029975
0.0649054	0	0	0	0.0731418	0.0175077	0.0301169	0.1538811
0.0327932	0.015959	0	0	0.0534626	0.037995	0	0.0851568
0	0	0.0002523	0	0.0470883	0.0791873	0	0.1176937
0.0071033	0.0057759	0	0.0405572	0.0458019	0.0672828	0	0.0503617
0	0.0174242	0.0178358	0.0260318	0.0249905	0.0426125	0	0.1458734
0.0020542	0.0203878	0.0391267	0	0	0.0256019	0.0334469	0.0389945
0	0.0368635	0.0129705	0.0362222	0	0.0868408	0.1243096	0.0213686
0	0.0121703	0.0034984	0	0.0255199	0.0999643	0.0812964	0.0385665
0	0.0343534	0	0.0930343	0	0.156967	0	0.0710809
0.0098704	0.0346485	0.0053663	0.0175505	0	0.0395922	0.0298928	0.1488172
0.0283705	0.0324246	0	0	0.0218072	0.0250022	0.0294894	0.0914409
0.0202234	0.0136736	0.0069132	0	0.0084437	0.1070635	0.0326661	0.0639395
0.0140895	0.0661778	0.0374185	0	0	0.0626816	0	0.0825181
0.0447601	0.0185286	0	0	0.0412428	0.0213775	0	0.0612568
0	0.0013529	0	0.0136401	0	0.0110095	0.0194406	0.0753777
0.0070218	0.0159624	0.0228391	0	0.0559121	0.0181556	0	0.0851977
0	0.0324764	0	0.0014158	0.0222862	0.0858384	0.0329667	0.0009768
0.0040914	0.0125168	0.0364184	0	0.027343	0.0524552	0.0023884	0.079471
0.0082138	0.0426871	0	0.0119351	0	0	0.1852434	0.0773924
0.0274306	0.0435581	0	0	1.38E-05	0.106959	0	0.1038159
0	0.0326499	0	0	0.0183127	0.0115508	0.1459182	0.0502354
0	0.0171945	0.0164027	0	0	0.0318525	0	0.0858493
0.0501875	0.0269221	0.0538742	0	0.0665937	0	0	0.0410578
0.0636501	0.0020265	0.0263525	0	0.037918	0.0780802	0	0.0883991
0	0	0	0.0025604	0.0218307	0.2262026	0	0.0206899
0.0517344	0.059361	0.0724891	0	0.0292625	0.0669343	0	0.0660152
0.0405858	0.0621596	0	0	0.0694624	0.0321884	0	0.0343999
0	0.017152	0	0	0.0313356	0.0103484	0	0.003615
0.0396101	0.0249102	0	0.0620348	0	0.0912547	0	0.1019045
0.0134316	0.0235842	0	0.0173668	0.0379018	0.0587006	0.0681285	0.0599708
0	0	0	0.0873496	0	0.0399412	0	0.0388258
0	0.0080112	0.0231831	0.0044657	0.1016055	0.0186418	0.0150755	0.10235
0	0.0020629	0.0515898	0.1154648	0.0245078	0.1213395	0	0.0882419
0.0321815	0	0.0728084	0	0.0651287	0.0087734	0	0.0679819
0.0360154	0.0337668	0	0	0.0192107	0.0160703	0.0170267	0.0731572
0.0541972	0.0093187	0.0499487	0	0.0900561	0.0370589	0	0.0691243
0	0.0181526	0.0052938	0	0.0176771	0.1920201	0	0.0868769
0.0461134	0.0084455	0.0245271	0	0.0409294	0.1006256	0	0.108701
0.0238652	0.0193125	0	0.074014	0.118935	0.0433645	0.0312801	0.0887207
0	0.0129033	0	0.042428	0.0053065	0.0139409	0.0340237	0.0721566
0.0287428	0.079734	0.0429911	0	0.0193631	0.0335213	0.0018951	0.0678314
0	0.0105974	0.0036384	0	0.0381763	0.1047933	0.0165645	0.1641097
0.0594514	0.0059621	0.0334492	0	0.0515426	0.0779286	0	0.0987082
0.0567428	0.0318216	0.0815842	0	0.0283715	0.0167896	0	0.0862709
0	0.0458765	0.0233438	0	0.0193961	0.0597856	0.0332657	0.0916586
0	0.0116913	0.0042677	0	0.0247234	0.072595	0.1625182	0.0295381
0	0.0070399	0	0	0.0146596	0.0310078	0	0.08401

0.0260017	0.0283969	0.0110061	0	0.0871816	0.0209318	0	0.0813531
0	0.0851917	0.0225132	0.0575411	0	0.0055988	0.1667748	0.0552061
0.0100966	0.0206458	0	0.096872	0.0273439	0.0508275	0.0013542	0.0785753
0.0088493	0.0226799	0	0.0309662	0.0074198	0.1001562	0.0574323	0.1270773
0.0174524	0.0387328	0	0	0.012098	0.0400797	0	0.1037351
0	0.0030098	0	0.0067106	0.0385608	0.0587418	0.0975893	0.0364577
0.0202582	0.0495689	0.0061067	0	0.1108979	0.0785062	0.0527766	0.0422544
0.0814208	0.006161	0	0	0.0253158	0.0014293	0	0.1384965
0	0.0376131	0	0	0.0120027	0.0225737	0.0841038	0.0836462
0	0.0347343	0	0	0.0068981	0.0025553	0	0.0301834
0.0097703	0.0152586	0.0412715	0	0.0743804	0.0491852	0	0.1070665
0	0.004096	0	0.0846017	0.0406968	0.1082023	0.0287244	0.044579
0	0.0041838	0.1055195	0	0	0.0214498	0.0462887	0.1502635
0.0280319	0.0392165	0.007166	0	0.0239405	0	0.3824327	0.0545106
0.0677804	0.0552203	0	0	0.0546472	0.0414069	0	0.0659759
0.0087687	0.0167578	0	0.0333061	0.0078581	0.0615561	0	0.0876328
0.0005184	0.002707	0	0.0818843	0.0060927	0.0311485	0	0.1384067
0.0114221	0.0117331	0	0.1367046	4.42E-05	0.0513835	0	0.0612239
0.0095466	0.1080278	0	0	0.1079405	0.0430811	0	0.1117824
0	0.015539	0.0489776	0	0.0012487	0.070664	0.0051759	0.0769451
0	0.0381758	0	0	0.0221576	0.1297068	0	0.0468271
0.0370245	0.021167	0	0	0.0574238	0.0100336	0.0752346	0.1032607
0.0834794	0.0679793	0.0418922	0	0.0457588	0.0092645	0	0.0664552
1.97E-05	0.0170835	0	0.0767101	0.0229368	0.0356276	0.0465992	0.0841125
0	0	0	0.0082785	0.009519	0	0.5353117	0.0341894
0.0484952	0.1106312	0	0	0.0352122	0.0292208	0.0317302	0.0759463
0	0.0278403	0.030789	0	0.0288634	0.0712468	0	0.0519758
0.0273898	0	0.012849	0.0515366	0	0.0457817	0	0.1634364
0	0	0	0.1647958	0.0471703	0.1269154	0	0.0512283
0.0084797	2.14E-05	0	0.0274398	0.0787032	0.2256337	0.1210279	0.0204993
0.0418071	0.0357387	0	0	0.0348499	0.016467	0.2018992	0.0446174
0.009961	0.0249823	0	0	0.0853094	0.0588004	0.0693892	0.0748462
0	0.0208262	0	0	0.0063768	0	0.2508306	0.036845
0.0437573	0.0709079	0.0287398	0	0.0976685	0.019018	0	0.0615289
0.0217632	0.011329	0	0	0.0875793	0.0547488	0	0.0958567
0.0096789	0	0.0281167	0	0.0524662	0.1065954	0	0.0993575
0.0934264	0.0190942	0	0	0.1111399	0.0121731	0	0.0984753
0.0669103	0.0247201	0.0392504	0	0.0351187	0.0144071	0.0161923	0.0465314
0.0134636	0.0218588	0.0149074	0	0.0441497	0.0816662	0.1498066	0.0698881
0.0121876	0.0362795	0.0204717	0	0.0303806	0.0146634	0.0604368	0.0880186
0	0.0240857	0	0	0.0399462	0.0063742	0.1009721	0.0366752
0	0.0146655	0.0296077	0	0.0308535	0.0993512	0.0110403	0.0813568
0	0.0187021	0	0.0748398	0.0328371	0.0235115	0.0041476	0.0894159
0.0091238	0.0248787	0	0	0.0414699	0.019065	0.0781418	0.0867615
0	0.0601164	0	0	0.0606328	0.0579589	0	0.0264035
0.0088899	0.0265961	0.0088547	0	0.0522439	0.063481	0.0489338	0.064367
0.017435	0.0672547	0.0041137	0	0.0318248	0.0943187	0	0.0955707
0.051212	0.0178412	0.05475	0	0	0.0006589	0.10068	0.0564438
0.0025623	0.0239983	0	0.0008361	0.0189309	0.0166801	0.0864688	0.0704161
0.0172601	0.0148646	0	0.0161368	0.0450379	0.0108373	0.0753994	0.0742901
0.0187063	0.0471343	0.0505738	0	0.0136824	0	0.1480496	0.0903183

0.0051738	0.0050983	0.0054852	0	0.0222361	0.0632181	0.0071728	0.0479819
0	0.0005562	0	0	0.0128534	0.0369972	0.1155579	0.0150668
0.0122553	0.0877351	0	0	0.0312104	0.1392183	0.1369645	0.0708468
0	0	0	0.0941559	0	0.0407445	0.0175678	0.0396998
0.0275894	0.0360688	0.0407173	0	0.0345578	0.0626754	0.0171096	0.0629171
0.0054513	0.0324623	0	0.0096868	0.0008993	0.1140014	0.0468692	0.0976212
0.0109863	0.0478046	0.0102155	0	0.0358776	0.0034337	0.0350735	0.1400129
0	0.0140944	0.0061877	0.0023338	0.0073087	0.1026813	0	0.0954956
0.00412	0.0052437	0.0008892	0.0154107	0.0222747	0.0587575	0.0030295	0.0838767
0.0326654	0.0426743	0.0262082	0	0.0495363	0.0452681	0	0.0976016
0.0081762	0.0235136	0	0.0301998	0.0801126	0.0102258	0.0208965	0.1201254
0	0.0195161	0.0149819	0	0.0214544	0.1277385	0	0.0729571
0.0037719	0.0931606	0	0	0.0439904	0.0100672	0.2002067	0.0659422
0	0	0	0	0.0324796	0.0261389	0.0753673	0.0818143
0	0	0	0	0.0431182	0.0368976	0	0.1184937
0.042014	0.0024042	0	0.0158071	0.0511649	0.0135842	0.0117486	0.0991991
0	0.0057331	0	0.0151926	0.0330799	0.0640459	0	0.0440298
0.0095614	0.0465423	0	0	0.0139019	0.0531524	0.0479536	0.0938109
0.0319626	0.0661613	0	0.0926085	0	0.0576799	0	0.0900636
0.0035377	0.0172452	0	0.0350246	0	0.0547571	0	0.0777594
0.0333044	0.036031	0.0923879	0	0.0108111	0	0.1476	0.0587683
0.0160578	0.0335957	0.0176158	0.012581	0.0113526	0.0204762	0.1365467	0.0600177
0.0053066	0.0346096	0	0.0425023	0.0150345	0.0561302	0	0.0732746
0.0292484	0.0506817	0.0826124	0	0.0864867	0.0087615	0.001161	0.0450768
0	0.0003556	0	0.0851537	0.0195372	0.0448634	0	0.1182754
0.0626893	0.0629359	0	0	0.0498273	0.0133688	0.0063061	0.0668465
0.0133885	0	0.021644	0	0.0513717	0.0237113	0	0.0727108
0.0247327	0.0159877	0.0129829	0	0.0892278	0.0824141	0.029722	0.0832996
0.0020866	0.0052141	0	0.0020934	0	0.0986409	0	0.0944261
0	0	0	0.0418038	0.0185006	0.1069169	0	0.059819
0.0406605	0.0650474	0.0271154	0	0.064176	0.0128502	0.0117254	0.1316603
0.065058	0.0460966	0	0	0.1001436	0.0139326	0	0.1048357
0	0.0251334	0.0153329	0.0382574	0	0	0.3936642	0.0538596
0.0113695	0.0249163	0.0163467	0	0.0057625	0.0634165	0	0.1281784
0	0	0.0124957	0	0.0236534	0.0277749	0.0225702	0.1442951
0.0535737	0.0706002	0.0573583	0	0.0053179	0	0.1259937	0.0876665
0.0150452	0.0422067	0.0184104	0	0.0764912	0.01848	0.0349949	0.1483837
0.0143185	0.1190879	0	0	0.0133476	0	0.0877542	0.0416302
0.0177657	0.0233441	0.0197681	0	0.0246989	0	0.0531798	0.0305246
0.0491497	0.0277707	0.0658904	0	0.0407152	0	0.0084969	0.0955008
0.0405805	0.0370721	0	0	0.0218134	0.1603259	0.0237392	0.0936595
0.0326622	0.164087	0.0727952	0	0.0155455	0.0226436	0.0950817	0.0623676
0.0064931	0.052425	0	0.0835944	0.011754	0.031457	0.014968	0.1030648
0	0.058209	0.0225063	0.018766	0.052403	0.004655	0.1244967	0.132557
0	0.0826528	0.0001603	0.0263031	0	0	0.1488074	0.0346996
0.036163	0.0357555	0	0	0.0148945	0.09605	0.0821286	0.0737162
0.0061154	0.028068	0.0040859	0	0.0217855	0.0179211	0.1230226	0.0768573
0.049115	0.0533132	0	0	0.0060596	0.0868724	0.0146272	0.092671
0	0.0061366	0	0.0254026	0	0.0245009	0.0160876	0.0816185
0.0252895	0.1143337	0	0	0.0619226	0.0140823	0.0140649	0.0693578
0.0079908	0.0145887	0.0039204	0	0.0878528	0.01106	0	0.0745929

0.010396	0.0302889	0.0151417	0	0	0.0207207	0.0145728	0.1484529
0.0036536	0.0188629	0	0.0086636	0	0.1876527	0	0.0800768
0.0138901	0.0299252	0.0407855	0.0066455	0.0295456	0.0923939	0	0.1143639
0.0278092	0.0687837	0.0627082	0	0.0322272	0.0088231	0.0580095	0.1033489
0.01016	0.0108724	0	0.0406968	0.0031453	0.0061034	0	0.1007981
0	0.0212479	0	0.0412805	0.0100541	0.0011672	0.0764065	0.071316
0.0120434	0.0356076	0	0.0133207	0.006528	0.1259167	0	0.0776874
0.0711239	0.0444947	0.0342424	0	0.0416171	0	0.081414	0.0722516
0	0.0301297	0	0.0159442	0	0	0.2924726	0.0303917
0.0323637	0.0287192	0.0153268	0	0.0621308	0.0269427	0.0376243	0.0602435
0.0635574	0.0558263	0.1130387	0	0.0232215	0.0207588	0	0.0988806
0.0053446	0.001567	0	0.045952	0.0232	0.1224028	0.0086683	0.1004855
0	0.0188804	0	0.0322185	0	0.1127067	0.0121453	0.0494181
0.0172773	0.0820181	0.0181716	0	0.0030733	0.0001791	0.0937063	0.0515192
0.0156059	0.0276056	0.0226897	0	0.0108914	0.045008	0.0043791	0.0702258
0.0616054	0.066333	0.0601128	0	0.0193413	0.0471358	0	0.1236756
0.0386211	0.1537369	0	0	0.0615222	0.0237036	0.0338014	0.0585071
0.0356927	0	0.0367281	0	0.0488205	0.0059023	0	0.0842651
0.0007674	0.0287586	0.0444561	0	0.0238591	0.070591	0	0.1149881
0.0450672	0.0465875	0.0091143	0	0.0210756	0.0144506	0.0167326	0.1012919
0	0	0.0162204	0	0.0473649	0.1029027	0	0.1641218
0.0031679	0.0059132	0.0438412	0	8.16E-05	0.0965916	0	0.0978174
0.018506	0.0269731	0	0.0001113	0	0.0233427	0.1048337	0.0901831
0.0349935	0.0747936	0.0133488	0	0.0431719	0.0210439	0	0.0506171
0	0.0098945	0	0.0106064	0.0257202	0.1147185	0.1450477	0.0288942
0.014197	0.0386271	0	0.002229	0.0152236	0.0925606	0.064044	0.1293333
0.0078805	0.0123822	0	0.0081738	0.0157918	0.0322508	0.0344424	0.0825987
0.0029926	0.0929106	0.0090461	0.0006565	0	0.0408029	0.031214	0.07699
0.0103814	0.0122135	0.0004813	0	0.0297206	0.0340888	0	0.1427935
0.0331398	0.0145302	0	0	0.0682547	0.0255405	0.0027609	0.0286944
0.022452	0.0777133	0	0	0.0320053	0.0189836	0	0.0678544
0	0	0	0.0231018	0.0396295	0.0895034	0.0505327	0.1732687
0.0294889	0.0779272	0	0	0.0388145	0.0937525	0.0322799	0.0618684
0	0.0486095	0	0	0	0.0022466	0.0354874	0.0714226
0.0219894	0.0302526	0	0	0.0532842	0.0130147	0.0247601	0.0774754
0	0.0010994	0.0006619	0.0122846	0.0116929	0.1543916	0	0.0618134
0.0085883	0.030688	0.0486535	0	0.0492021	0.0294835	0	0.116235
0.0449915	0.0514582	0	0	0.0452131	0.0443663	0	0.0824069
0.0168319	0.0481192	0.0015618	0	0.0097808	0.0486959	0.0693592	0.0838651
0.0106402	0.0195051	0	0	0.0175499	0.0043475	0.0332348	0.0831929
0.0175475	0.018993	0	0	0.0179723	0.0055279	0.0471093	0.0436877
0	0.0562723	0	0	0.010114	0.141487	0.1807201	0.0582501
0.0215548	0.056709	0.0325028	0	0.071989	0.0248797	0	0.044603
0.0110022	0.0082134	0.0013552	0	0	0.0640706	0	0.0840611
0.0513468	0.0245854	0.0620286	0	0.0378013	0.042538	0	0.0701507
0.0456168	0.0203437	0.0100314	0	0.0268088	0.0443371	0.0735713	0.1061665
0.0064165	0.0172626	0	0.0135899	0.0069621	0.04645	0	0.146711
0	0.0080207	0	0.0203998	0	0.0101895	0.0111376	0.0416649
0.037949	0.0525325	0	0	0	0.030881	0.0219987	0.0804261
0.0942805	0.0557949	0.0744588	0	0	0	0.0302733	0.1363327
0.0154422	0.0238589	0	0.0162373	0.0278706	0.017243	0.0552467	0.0956257

0	0.0045879	0	0	0.0565291	0.0287612	0	0.0914539
0	0.0056166	0.0222633	0.0111261	0	0.0460668	0.0224529	0.1907433
0	0.0086846	0	0.1669024	0	0.0843215	0.0167708	0.0764823
0.00305	0.0215514	0	0.0192836	0.0383137	0.1224479	0	0.0990888
0.0271234	0.0206885	0	0.0220852	0.0080913	0.0596097	0	0.1037144
0.0274826	0.0669685	0	0.0473764	0.0233175	0.0288733	0.1414291	0.0245805
0.0421314	0.0328031	0.069378	0	0.0563012	0.0217216	0	0.0846139
0.0058937	0.0363046	0	0	0.0361742	0.014414	0.1022659	0.0608911
0.060641	0.0327172	0	0.0515364	0.0057423	0.1019596	0.0766222	0.0648323
0.0090165	0.0495119	0.0042791	0	0.0011124	0.0209904	0.1878207	0.0452514
0.0224277	0.0878384	0	0	0.0431809	0.0750903	0	0.0571234
0.028127	0.0521001	0.0853358	0	0.0266768	0.0063417	0	0.0562287
0.0633349	0.0472417	0.0270815	0	0.0149899	0.0530517	0.005815	0.0484017
0.0022378	0.0521497	0.0129256	0	0.0326845	0.0153814	0.3223109	0.0342779
0.0158264	0.0499159	0.0188854	0	0.0368191	0.0240113	0	0.078265
0	0.0363734	0.0166978	0.0454716	0	0	0.5269824	0.0211361
0	0.000794	0	0	0.0494002	0.1909332	0	0.0596975
0	0.0230534	0	0	0.0252435	0.059941	0.0469547	0.0001543
0	0.0216298	0	0	0.0879846	0.0211754	0	0.1331467
0.0181854	0.0031477	0	0	0.0739018	0.0432853	0.063858	0.0833709
0.0235885	0.0316327	0.1092893	0	0.014113	0.0031487	0.0062019	0.0711285
0.0142834	0	0.0054766	0.0060953	0	0.1203937	0	0.0729203
0.0002639	0.0346695	0	0.009644	0.0652122	0.0464416	0.0444272	0.0324737
0.0366301	0.0209112	0.1408032	0	0.0172151	0	0.086801	0.1119519
0.0383388	0.0206861	0.0938959	0	0.0051276	0	0.0258768	0.0661101
0.0192578	0.0082686	0.0343213	0	0	0.0705693	0.0359007	0.0729667
0.0069448	0.0388627	0.0132131	0	0.023325	0	0.106597	0.0702458
0.0256443	0.0435727	0.002661	0	0.0569616	0.0677163	0	0.1092963
0.0084054	0.1152582	0	0.0371428	0	0.0694909	0.234963	0.0824555
0.000991	0.0004668	0	0.0357708	0.0216367	0.221316	0	0.0879632
0.0091357	0.016063	0	0	0.0207318	0.0220922	0	0.1099014
0.0073058	0.0344108	0	0.0943974	0.0169882	0.0401681	0	0.1254735
0	0.0047351	0	0	0.0378939	0.2242031	0	0.1176098
0	0.0378678	0	0.0074654	0.1019492	0.1343481	0	0.0715909
0.0187587	0.0481334	0.0613155	0	0.0343242	0.0641011	0	0.0887113
0	0.0146669	0.0085088	0	0.0002786	0.098868	0.0758308	0.0676197
0.0007357	0.0097285	0	0.0299374	0.0193003	0.0291535	0.0859347	0.0408279
0.031758	0.0170073	0	0	0.0921599	0.0331411	0.0505356	0.1449987
0	0.0259955	0	0	0.0797997	0.1031318	0.0094689	0.0429947
0.0235934	0.0740023	0.0325523	0	0.0256657	0.0311078	0	0.1197455
0	0.0006134	0	0.0865269	0	0.0878847	0	0.0851486
0.0050495	0.0020786	0	0.0220141	0	0.0319698	0.096015	0.0483086
0	0	0.0690537	0.0732352	0.0142879	0.1793696	0	0.0523971
0	0.0028798	0	0	0	0.0230236	0.011119	0.0620371
0	0.0708941	0.0460715	0.0181905	0.0021753	0.0360011	0	0.1520996
0.0262599	0.0446227	0.0310775	0	0.0367981	0	0.2455732	0.0795465
0.0178432	0.0330152	0.0197318	0	0.0390502	0.0126281	0	0.113805
0.0256252	0.0305953	0	0	0.0253697	0.1163856	0.0475647	0.0681425
0.0610351	0.058055	0	0	0.0101233	0.0142387	0.0468603	0.08608
0	0.0106698	0	0.0423128	0.0222962	0.1025543	0	0.1172559
0.001185	0.0477092	0	0	0.026461	0.0094844	0.0367069	0.1212751

0.0082787	0.0362979	0	0	0.0084392	0.0471891	0	0.1591844
0.0149096	0.0441303	0.0583052	0	0.0102216	0.0452053	0	0.136086
0.0407637	0.0161761	0.0644297	0	0.0445104	0.0010459	0	0.0796654
0.0643086	0.0253626	0.0340276	0	0.0136621	0.0624637	0	0.1049118
0.0017956	0	0	0.0161019	0.0072137	0.1219111	0.1421201	0.0461629
0	0	0.0385111	0	0.0526792	0.014942	0	0.1131808
0.0737187	0.0704214	0	0	0.044183	0.0544509	0	0.055362
0	0	0	0.0061639	0.0694906	0.1241884	0	0.0745457
0	0.0009323	0.001122	0	0.0165903	0.0001831	0.1903987	0.0161303
0.0466559	0.0154225	0	0	0.0202539	0.0328737	0	0.1349816
0.0353133	0.0411936	0.0028705	0	0.0202682	0.067187	0.1266555	0.0320661
0	0.0225295	0.0366466	0	0.0262299	0.0432401	0	0.0865466
0	0	0	0	0.0084112	0.0979664	0	0.0803898
0.0349897	0.003002	0.0103483	0	0.0066629	0	0.0211124	0.1535195
0.0647643	0.0898793	0	0	0.0367121	0.0575174	0.0322987	0.0629192
0.0134473	0.032514	0.0199789	0	0	0.1309368	0	0.1321179
0.0374728	0.0016986	0.0382092	0	0.0530447	0.1440941	0.0359519	0.1295781
0.0056597	0.0238562	0.0550501	0	0.0244501	0.0422934	0	0.1946969
0.0665351	0.0634091	0.0758135	0	0.0677936	0.0482878	0.0514197	0.0856958
0.0204563	0	0	0	0.044516	0.1507571	0.0121528	0.123564
0.0106872	0.0735209	0	0	0.0511055	0.0125475	0.1224586	0.0713599
0.0093561	0.0527419	0	0	0.0565738	0.0601333	0.0352609	0.132341
0	0.0820174	0	0.0056933	0.0327122	0.0099729	0.2762443	0.0375502
0.0038968	0.0467483	0	0	0.0295132	0.025089	0.1308906	0.0367861
0.0308449	0.0588817	0	0	0.0159116	0.0806042	0	0.0868987
0.0132472	0	0.0164503	0.0364088	0	0.0919312	0	0.0901465
0	0.0479892	0	0.0280769	0.0040285	0.0290316	0	0.0583241
0.0244592	0.0113939	0	0	0.0025114	0.0479048	0.0804428	0.1014008
0	0.0255479	0.0052639	0.0071963	0.0269679	0.0242434	0.0062659	0.1162704
0.0589906	0.0684789	0.015719	0.0096635	0	0.096256	0.0108359	0.1022539
0.0156464	0.0089567	0.0549652	0	0	0.0202219	0.0821166	0.1084841
0.0047773	0	0.1515104	0	0.0052272	0.0743241	0	0.0757793
0.0204566	0.0342709	0	0.0249497	0.0251347	0.1102583	0.1344725	0.0892912
0.0335168	0.0131784	0.0061722	0	0.0338749	0.0685116	0.0495427	0.1545568
0.005096	0.0300969	0	0.1227914	0.0062592	0.0509109	0.1519849	0.0099931
0.0022735	0.0455418	0	0	0.0149247	0.0515846	0.0754616	0.1064518
0.0036911	0.0038351	0.0196146	0	0	0.0241427	0.0516727	0.05757
0	0.0146016	0.0341962	0	0.0160958	0.026822	0	0.1289522
0.0078868	0.0414779	0	0.0440978	0.0792185	0.089914	0.0429112	0.0273527
0.0401665	0.0486152	0	0	0.0235222	0.0437068	0.1856591	0.0828021
0	0.0017417	0	0.0134755	0.0439082	0	0.1101928	0.0262519
0	0.011249	0	0	0.0673128	0.10945	0	0.1118755
0	0.0239481	0	0	0.0517792	0.103945	0	0.0674795
0	0	0	0.0375755	0	0.0692338	0.0176457	0.0812198
0.0231637	0.0259571	0.0145381	0	0.0257292	0.108488	0.0246278	0.0890557
0.0236244	0.0863332	0	0	0.0277926	0.0569529	0	0.0663427
0	0.0598434	0	0.0619683	0	0.2059522	0.0128529	0.0599555
0.0340543	0.1829671	0	0.0361757	0.1685003	0.0211112	0.0113504	0.0628859
0	0.0287597	0	0.0356159	0.1908042	0.1067038	0.0792083	0.0593621
0.0043495	0.0012689	0	0.0927929	0.056868	0.0897914	0	0.1110151
0.010231	0.079457	0.0384845	0	0.023404	0.0670076	0.0673612	0.1083282

0	0.0215937	0	0.2500506	0.0746365	0.1753374	0.033264	0.0433051
0.0113869	0.0800551	0	0	0.0828948	0.0711236	0	0.0759254
0.0306542	0.0344897	0	0	0.0523252	0.0709118	0.0128743	0.0918627
0	0.0024575	0	0	0.0612291	0.0704596	0	0.0089997

Macrophage	Dendritic	Dendritic	Mast cells	Mast cells	Eosinophil	Neutrophil	P-value
0.1387399	0.0395051	0	0.0811919	0	0	0	0.02
0.1317003	0.0099492	0	0.037799	0.0009057	0	0	0
0.2722457	0	0	0.0408701	0	0	0.0167205	0.04
0.2409923	0	0.0008228	0.0371144	0	0	0.0164535	0
0.3669192	0.0016788	0	0.0677345	0	0	0	0
0.2505357	0.0020381	0	0.0370812	0	0	0.0042855	0
0.3728858	0.0242778	0	0.0862343	0	0.0573441	0	0.24
0.4008625	0	0	0	0.0333936	0	0.0103042	0
0.3321853	0.0314839	0	0.0806804	0	0	0	0
0.1871519	0	0	0.0322148	0	0	0.016701	0.02
0.2153058	0.0183957	0	0.0204321	0.0150072	0	0.0293558	0.12
0.5501093	0.0459447	0	0.0212263	0	0	0.0112708	0.02
0.286263	0	0	0.0508448	0	0	0.0009429	0
0.230943	0.0126706	0	0.0369497	0	0	0.0010252	0
0.3350843	0.0068078	0	0.1036387	0	0	0.010058	0
0.1546373	0.0565288	0	0.1028102	0	0	0.0051754	0
0.0673844	0	0	0.2008185	0	0.0237664	0.0846514	0.46
0.191349	0.0269555	0	0.046713	0	0	0.0053053	0
0.2854123	0.0095642	0	0	0.0936533	0	0.0085451	0.04
0.1994537	0.0018371	0.006918	0.0350694	0	0	0.0415859	0.16
0.1737398	0.0361701	0	0.1096476	0	0	0.0096834	0
0.2319014	0.0038217	0	0.0142933	0	0	0.0537325	0
0.3234978	0	0	0.0340097	0	0	0.0296228	0
0.2440888	0.0158267	0	0.2047711	0	0	0.0214169	0
0.2213648	0	0	0.0124765	0	0	0.0010186	0
0.2101357	0.018331	0	0.0925645	0	0	0	0.1
0.4041093	0	0	0.0779645	0	0	0.0187049	0
0.3049965	0.0215008	0	0.0439408	0	0	0.0025643	0.02
0.2073666	0.0126367	0	0.0498723	0	0.0003044	0.0088446	0
0.2566063	0	0	0.0648998	0	0	0.0189267	0
0.4588086	0.0060029	0	0.1419998	0	0	0.0285863	0
0.250827	0.0047557	0.0001459	0.0998421	0	0	0	0
0.3332338	0.0732464	0	0.0446294	0	0	0.0035448	0.1
0.207994	0.0167596	0	0.0418575	0	0	0.0039268	0
0.1607711	0.0145481	0	0.1423027	0	0.0004312	0	0
0.2758927	0.0937343	0	0.0992772	0	0	0.0151266	0
0.1860363	0	0	0.0400688	0	0	0	0
0.2998164	0.0157848	0	0.0839913	0	0	0	0
0.3562084	0.0040602	0	0.052293	0	0	0.0892887	0
0.3429031	0	0.0017299	0.0604808	0	0	0.0143687	0
0.3103374	0	0	0.0187407	0	0	0.0023341	0
0.1916327	0.1097408	0	0.0802561	0	0	0.0041163	0.42
0.2254587	0.2398076	0	0.2019556	0	0	0.0294259	0.28
0.4476956	0.0042772	0.0130563	0.0333357	0	0	0.0155648	0
0.4257065	0.0334104	0	0.0617315	0	0	0.0048861	0.06
0.0940914	0.0046443	0	0.0235785	0	0	0	0
0.1436497	0.0253324	0	0.1121952	0	0	0.005044	0
0.2523803	0.0205001	0	0.0762792	0	0	0	0
0.1987064	0.0735709	0	0.0965474	0	0	0	0.1

0.208394	0.0340834	0	0.0315349	0	0	0	0.04
0.2430222	0.0195365	0	0.144305	0	0	0.0032378	0.34
0.1620811	0.0218425	0	0.0463437	0	0	0.0041582	0
0.1521815	0.0508449	0	0.0524557	0	0	0.0142578	0.04
0.1076427	0.0270567	0	0.0315137	0	0	0	0
0.1496848	0.0195152	0.1019277	0.1437285	0	0.0044042	0.0013943	0.46
0.0525793	0	0.0276004	0.037938	0	0.0150567	0.0698601	0
0.0609444	0.0200461	0	0.0339913	0	0	0	0
0.1696758	0	0	0.0391815	0	0	0.0050863	0
0.2291836	0	0	0.2244677	0	0	0.0001521	0.44
0.2071349	0	0.0021928	0.0247092	0	0	0	0
0.1813078	0	0.003737	0.0271605	0	0	0	0
0.094753	0	0.151354	0.0665765	0	0.0149632	0.0040592	0.46
0.1245395	0	0.0800671	0.1904164	0	0.0060292	0.0139986	0.58
0.2545024	0.0163403	0	0.0834595	0	0	0.0021031	0
0.2265623	0	0.0024712	0.0577587	0	0.0070809	0.0265877	0.14
0.1453217	0.0277148	0.0014428	0.1303134	0	0	0	0.6
0.2008271	0.0896693	0	0.0723944	0	0	0.0008487	0
0.0499347	0.0167942	0	0.0004389	0	0	0	0
0.2665968	0.0001056	0	0.0406003	0	0	0	0
0.0183934	0.0042857	0	0	0	0	0	0
0.4811527	0.0649602	0	0.0337401	0	0	0.0174339	0
0.1564662	0.0139716	0.0021384	0.0461502	0	0	0.0013712	0
0.3686139	0	0	0.0278887	0	0	0.006397	0
0.0826613	0	0.0051496	0.05997	0	0	0.0128973	0.08
0.1799081	0.16525	0	0.0801534	0	0	0	0.08
0.2521554	0.0068958	0	0.1445729	0	0	0.0131733	0.14
0.2829447	0.009463	0.0062882	0.0728308	0	0	0	0
0.2813355	0	0.018752	0.1169141	0	0	0	0.08
0.2054364	0.0043571	0	0.015369	0	0	0.0370101	0
0.0836256	0.0188821	0	0.0186887	0	0	0	0
0.1398573	0	0	0.0270936	0	0	0.0046963	0
0.2267659	0.007347	0	0.0145446	0	0	0.0025477	0
0.1359478	0	0	0	0.0209101	0	0	0.1
0.0561156	0	0	0.0189046	0	0	0	0
0.1347658	0.0001208	0	0	0.0044256	0	0.0180677	0
0.1900463	0.0366338	0	0	0	0.0298462	0	0
0.1205434	0	0	0.0100335	0	0	0	0
0.1253722	0.0261084	0	0.0850674	0	0	0.0026031	0
0.229087	0	0	0.0213514	0	0	0	0
0.0361188	0	0	0.0779301	0	0	0	0
0.0182632	0.0041537	0	0.0103683	0	0	0	0
0.1245492	0	0	0.0267975	0	0	0.0093675	0
0.1472734	0	0	0.0283603	0	0	0	0
0.1123884	0.0047964	0	0.0416497	0	0	0	0
0.1420862	4.09E-05	0	0.0312282	0	0	0	0
0.1503778	0.0031786	0	0.0913147	0	0	0.0027188	0
0.1608452	0.0117157	0	0.0479642	0	0	0	0
0.1738382	0.0194102	0	0.0837744	0	0	0	0
0.3030893	0	0	0	0.0628496	0.0050623	0	0
0.1842833	0.0213002	0	0.1564463	0	0	0.0150163	0.12

0.1517028	0.0140911	0	0.0084993	0	0	0.0031991	0
0.2601753	0	0.0029391	0.044932	0	0	0.0098622	0
0.0861769	0	0	0.0310472	0	0	0.4634192	0
0.0344347	0.0169516	0	0.0395405	0	0	0	0
0.0748362	0.0029618	0	0	0	0	0	0
0.0468043	0	0	0.0211606	0	0	0	0
0.2782789	0	0	0.045054	0	0	0.0028875	0
0.2268568	0.0098212	0	0.0634284	0	0	0	0
0.110653	0.0227744	0	0.0499706	0	0	0.001412	0.02
0.1744965	0	0	0.0432902	0	0	0.0055112	0
0.090419	0.0057337	0	0.0183831	0	0	0	0.02
0.0857727	0.0184621	0	0.0518557	0	0	0.000234	0
0.3310056	0	0	0.0768763	0	0	0	0.88
0.1202843	0.0049823	0	0.0220603	0	0	0	0
0.1737092	0	0	0.0251094	0	0	0.0070443	0
0.0342531	0.0138806	0	0.0388708	0	0	0.0020006	0
0.2191311	0.0214	0.0058897	0.1318243	0	0	0.0095491	0
0.2313102	0	0	0.0846119	0	0	0	0
0.1022136	0.0136127	0	0.0333839	0	0	0.0376567	0
0.1695512	0	0	0.0323053	0.0051016	0	0.0058312	0
0.1371038	0	0	0.0299029	0	0	0	0
0.1737497	0	0	0.0564983	0	0	0.0054978	0.46
0.1619464	0.0107583	0	0	0.0322588	0	0.0096489	0
0.0638413	0.0117611	0	0	0	0	0.0033745	0
0.2607975	0	0	0.0343224	0	0	0.0049197	0
0.1681998	0	0	0.0463766	0	0	0.0018843	0
0.2057644	0	0	0.0502637	0	0	0.0047914	0
0.2663839	0	0	0.0450094	0	0	0	0
0.4286256	0.0057988	0	0.0017797	0	0	0.0040645	0
0.1697989	0.0025355	0	0.089772	0	0	0	0
0.1447033	0.0015593	0	0.0568216	0	0	0.0008694	0
0.1373849	0.0027275	0	0.003788	0	0	0	0
0.3212548	0.0311737	0	0.0309055	0	0	0	0
0.2132276	0.0014723	0	0.0133255	0	0	0.0494151	0
0.1906904	0	0	0.0057371	0	0	0	0.32
0.2950668	0.0105501	0	0.017498	0	0	0	0
0.0779992	0.0032611	0	0.0100946	0	0	0	0
0.218946	0	0	0.0571081	0	0	0.0170554	0.02
0.1923545	0.0087371	0	0	0.036291	0	0	0
0.3022456	0	0	0	0.0312767	0	0.0313244	0
0.2175597	0	0.0175183	0.0483819	0	0	0.0050639	0
0	0	0	0	0.027091	0.0039711	0	0.28
0.276511	0	0.0075593	0.0343663	0	0	0	0.6
0.1399164	0	0	0.0647931	0	0	0.0057741	0
0.2638349	0.0068999	0	0.0263816	0	0	0	0
0.1462005	0.0079517	0	0.0428146	0	0	0.0143534	0.04
0.1631037	0	0.0004143	0.0553441	0	0	0.0073898	0
0.119539	0	0	0	0	0	0	0
0.2069407	0	0	0.0076317	0	0	0.0005947	0
0.0830205	0.0108765	0	0.0291269	0	0	0.014282	0.04
0.2421167	0.0583629	0	0.0169518	0.0114469	0	0.017262	0.22

0.2144477	0	0	0.040196	0	0	0.0007892	0
0.1663907	0.0193497	0	0.0413094	0	0	0	0
0.171351	0.0022382	0	0.0707934	0	0	0	0.12
0.250447	0.0225176	0	0.1086683	0	0	0	0.42
0.2053313	0	0.0007984	0.0403498	0	0	0.0047747	0.08
0.3583043	0.0097641	0	0	0.1318415	0	0	0
0.0846408	0.006407	0	0.0811851	0	0	0	0
0.1242958	0.1135614	0	0.0994482	0	0	0	0
0.2269141	0	0	0	0.0128619	0	0.0523444	0
0.1860387	0.0081826	0	0.0786675	0	0	0.0139798	0
0.1081622	0.0389514	0	0.0922975	0	0	0	0
0.126467	0.0072654	0	0.0967641	0	0	0.0074075	0
0.0306481	0.0164441	0	0.0347553	0	0	0.0028666	0
0.2774641	0.0267068	0	0.1518224	0	0	0.0301626	0.06
0.246119	0.0420028	0	0.0593002	0	0	0.0022251	0
0.1175023	0.0033533	0	0.1288031	0	0	0.0093306	0.26
0.2292068	0.0069804	0	0.0643257	0	0	0.001759	0
0.1914849	0.0215929	0	0.0618514	0	0	0.0100285	0
0.1351363	0.0156794	0	0.0250428	0	0.0048396	0.0219398	0
0.1788132	0.0289644	0.0055415	0.0949631	0	0	0.0088377	0
0.1624173	0.0043247	0.0038938	0	0.0571912	0	0	0.44
0.194025	0.0018355	0	0.0253421	0	0	0.014501	0
0.2620926	0	0.0210226	0.0514828	0	0	0	0
0.0031791	0.0018524	0	0.0578254	0	0	0	0
0.1946362	0.0186887	0	0.0293188	0	0	0.0064449	0.08
0.0267149	0	0	0.0245813	0	0	0.0039589	0
0.332636	0.0362754	0	0.0608909	0	0	0.0092992	0
0.1339833	0.0373541	0	0.0479092	0	0	0.0002902	0
0.1482224	0.0086047	0	0.0470205	0	0	0	0
0.1258507	0.0264905	0	0.088693	0	0	0	0
0.1422717	0	0	0.0057094	0	0	0.0039266	0.06
0.1503731	0.0013672	0	0.0821252	0	0	0	0
0.1956786	0.0476481	0	0.0677243	0	0	0.0053283	0.06
0.3793824	0.0278492	0	0.173405	0	0	0	0.42
0.1477338	0	0.0001823	0.0156472	0	0	0	0
0.3218752	0.004634	0	0.0298023	0	0	0.0603553	0
0.3205522	0.0249982	0	0.0603135	0	0	0.0067091	0
0.1501859	0.0464439	0	0.094835	0	0	0	0
0.2889805	0.0103978	0	0.0710758	0	0	0.0225998	0.02
0.3525302	0.0111835	0	0.0806831	0	0	0.0041632	0
0.2812789	0	0.0001846	0.0353236	0	0	0	0
0.2603101	0.001109	0	0.1825943	0	0	0.0100837	0.04
0.1650677	0.0864823	0	0.0329268	0	0	0.0093975	0
0.1622309	0	0	0.0302566	0	0	0.0040037	0
0.1960497	0.0749236	0	0.0669907	0	0	0	0
0.1957425	0	0	0.0275088	0	0	0.0039724	0.28
0.2102759	0	0.0019757	0.2164655	0	0	0.0363372	0.28
0.2040193	0	0	0.0596795	0	0.0544978	0.0133811	0.1
0.1944796	0.0174209	0	0.1166798	0	0	0	0
0.2995035	0.0121408	0.0293152	0.0368777	0	0	0	0.04
0.1776503	0	0	0.0858637	0	0	0.0240711	0.16

0.2114226	0.0285071	0	0.0631048	0	0	0.0121795	0
0.4676475	0.0179237	0	0.1010178	0	0	0.0005447	0.1
0.0780355	0	0	0.1135143	0	0	0.0003545	0
0.1715512	0	0	0.0016493	0	0	0.0061779	0
0.1394975	0.0052074	0.0084114	0.1434496	0	0	0.005563	0
0.0944931	0.0100003	0	0	0.010909	0	0	0
0.3220513	0	0.0310319	0.0927923	0	0	0.017476	0.06
0.248473	0	0.0070723	0.0761222	0	0	0.0023649	0.66
0.2611445	0.0313912	0	0.0586753	0	0	0.0105033	0
0.1399452	0	0.0026159	0.1207641	0	0	0.0002024	0
0.2086147	0	0.0779896	0.0108964	0	0	0.1048246	0
0.4537055	0	0	0.1088835	0	0	0.0206324	0
0.289177	0.0011198	0	0.0504328	0	0	0.0075332	0
0.1117149	0.1288476	0	0.0226811	0	0	0.0361588	0
0.1212772	0.0078251	0	0.0842064	0	0	0.0071509	0.04
0.2401709	0	0	0.0030143	0	0	0	0
0.2526875	0	0	0.015162	0	0	0.0005417	0
0.1240232	0.0249346	0	0.0511384	0	0.0008063	0.0057268	0
0.2130161	0.0032444	0	0.0635452	0	0	0.0095375	0
0.3085519	0.0278561	0	0.0985251	0	0	0.0148711	0
0.1825347	0	0.0049294	0.0395717	0	0.0014849	0	0.24
0.1665688	0	0	0.0223972	0	0	0.014228	0
0.3035777	0	0	0.0390713	0	0	0.0295324	0
0.2138762	0.022417	0	0.0252624	0	0	0	0.1
0.2922816	0.0270329	0	0.0203454	0	0	0.0061207	0
0.1998991	0.0078349	0	0.0168926	0	0	0.0224488	0
0.1894437	0.0087232	0	0.0083894	0	0	0.0042633	0
0.3086719	0.0381089	0.0250861	0.062387	0	0	0	0.04
0.3589483	0.0036078	0	0.0430069	0	0	0	0
0.1608234	0.0342751	0	0	0	0	0	0
0.1018686	0.0155356	0	0.0785742	0	0	0.003359	0
0.2074686	0.0222378	0.0125944	0.1108116	0	0	0.0089505	0
0.1286944	0	0.0016786	0.0245945	0	0	0	0.06
0.1547277	0	0	0	0.0755857	0	0.0089812	0.14
0.2435233	0.0052953	0	0.108102	0	0	0	0
0.1593829	0.0334695	0	0.2454213	0	0	0	0.36
0.2179402	0.0098007	0	0.090684	0	0	0	0
0.118261	0.0147968	0	0.0550582	0	0	0.0020815	0
0.22772	0.0196941	0	0.0603248	0	0	0.0037017	0
0.2017993	0.0073182	0	0.001017	0.0024081	0	0.0044555	0
0.2847869	0	0	0.0605602	0	0	0	0
0.209358	0.0046465	0	0.0622205	0	0	0.002888	0.08
0.035954	0.0251405	0	0.1047342	0	0	0	0
0.2359145	0.0193098	0	0.0547567	0	0	0.0026919	0
0.2563336	0	0	0.0153259	0	0	0	0
0.1298211	0.0315641	0	0	0.0556705	0	0.0448342	0
0.2056497	0.0142369	0	0.0076367	0.0010357	0	0.0168027	0
0.0864726	0.0153721	0	0.0098978	0	0	0.009778	0
0.2878186	0.0229006	0	0.1052619	0	0	0.0241729	0
0.2754133	0	0.0188632	0	0.0045216	0	0.0297513	0
0.177805	0.0736345	0	0.0342925	0.0001765	0	0	0.06

0.1694189	0.0156527	0	0.0158842	0	0.0016415	0.00857	0
0.1649654	0	0.0006326	0.1025735	0	0	0.0307216	0
0.2747768	0.0045349	0	0.0448456	0	0	0	0.06
0.2178643	0.0154136	0	0.0815378	0	0	0	0
0.0971116	0.0161891	0	0.0372228	0	0	0.0001965	0
0.4310867	0.006361	0	0.1082794	0	0	0	0
0.2567813	0.0032595	0	0.1941521	0	0	0.0099093	0.28
0.1117273	0.0052327	0	0.0037017	0	0	0.0003023	0
0.1953838	0.0033617	0	0.11121	0	0	0	0
0.5136195	0	0.0060522	0.0842154	0	0	0.0079326	0.06
0.1809545	0.0147625	0.0107699	0.1346965	0	0	0.001315	0.12
0.152518	0	0	0.190479	0	0	0.0088879	0.82
0.2384349	0.009386	0	0.0671924	0	0	0.0024773	0
0.1908613	0	0	0.0447132	0	0	0	0
0.0900483	0.002147	0	0	0	0	0	0
0.2366541	0.0138322	0	0.0502327	0	0	0	0.3
0.2018925	0	0	0.0247215	0	0	0	0.14
0.2497166	0.0005194	0	0.0302475	0	0	0	0.08
0.279129	0	0	0.0386139	0	0.0016369	0	0.6
0.4877796	0	0	0.0137725	0	0	0.1083989	0
0.3577159	0.000633	0.0004892	0.1064526	0	0	0	0.04
0.2311916	0.0894251	0	0	0.0086115	0	0	0
0.1070939	0.0063836	0.0059335	0.0185231	0	0	0	0
0.2043044	0	0.0060514	0.0662273	0	0	0.0005014	0
0.1455603	0	0	0.0817957	0	0	0.0009228	0
0.09786	0.006274	0	0.0258903	0	0	0	0
0.2360287	0.0708618	0	0.1085869	0	0	0.0012303	0
0.2255151	0.0152594	0	0.0566332	0	0	0	0
0.2334195	0.01463	0	0.0701874	0	0	0	0.06
0.2945637	0	0	0	0.0383502	0	0.0241514	0
0.202597	0	0	0	0.0136426	0	0	0.08
0.0908171	0.0040217	0	0.1067222	0	0	0.0013829	0.26
0.3331524	0	0	0.0387349	0	0	0	0
0.0664549	0.0080326	0	0.0115226	0	0	0	0
0.1371511	0.0171433	0	0	0.1710528	0	0	0.16
0.1828293	0.0280195	0	0	0.0293599	0	0.0191692	0.14
0.1278126	0	0.0004224	0	0.0832053	0	0	0
0.1858845	0.0074068	0	0.0052865	0	0	0.003224	0
0.3264501	0	0	0	0.1196071	0	0.0059883	0
0.2978143	0.0112309	0	0.0588689	0	0	0.0064961	0
0.3027858	0	0.0523587	0.1018862	0	0	0	0
0.3744835	0.0032181	0	0.0596639	0	0	0.0522057	0
0.2080813	0.0305058	0	0.1479277	0	0	0.0083141	0.08
0.1179475	0.0277151	0	0.0539648	0	0	0	0
0.1861194	0.0868881	0	0.1304916	0	0	0.0051888	0
0.2407476	0.0025372	0	0.0332432	0	0	0.0267434	0
0.1393181	0	0	0.0107829	0	0	0.0004491	0
0.1305986	0	0	0	0.0155679	0	0	0
0.4861411	0.0226881	0	0.0463787	0	0	0.0052137	0
0.2834401	0.0228827	0	0.0335628	0	0	0.004938	0
0.2046819	0	0	0.0293509	0	0	0	0

0.5387396	0	0.0503021	0	0.040237	0.0015599	0.0306353	0
0.3232653	0.0159683	0.0300383	0.0513476	0	0	0.001027	0
0.1884449	0	0	0	0.0248178	0	0.0040757	0.1
0.3446644	0.003197	0	0.0617127	0	0	0.0085676	0.04
0.1801951	0.0427466	0	0.0309753	0	0	0	0
0.2555359	0.0247479	0	0.0241279	0	0	0.0011098	0
0.1943922	0.0441317	0	0.0474815	0	0	0	0
0.3086901	0.0101591	0	0.0746699	0	0	0.0148346	0
0.3134279	0	0	0.0841163	0	0	0.0028612	0.06
0.1301487	0.0077164	0	0.0096795	0	0	0	0
0.2170503	0.0109963	0	0.1217925	0	0	0	0.1
0.2029365	0.0407262	0	0.0271201	0	0	0.0039557	0
0.1553995	0.0561035	0	0.0442331	0	0	0	0
0.5154998	0.0034261	0	0.0595361	0	0	0	0
0.177104	0.0215538	0	0.0850948	0	0	0.0122194	0
0.1828985	0.0131973	0	0	0.0195935	0	0.0060257	0.08
0.316996	0	0	0.1201732	0	0.0069977	0	0
0.2805157	0.0117456	0	0.0646208	0	0	2.72E-05	0
0.2056566	0	0	0.0249104	0	0	0	0
0.3245287	0.0013144	0	0.0366206	0	0	0.0039162	0
0.1040839	0.025381	0	0.0043038	0	0	0	0.1
0.2131956	0	0.0827751	0	0.0689718	0	0.0162838	0.04
0.2088189	0.0214687	0	0.0620507	0	0	0.0073465	0
0.0827713	0.0172164	0	0.0367419	0	0	0.0028543	0
0.3644391	0.0145024	0	0.0914333	0	0	0.0149986	0.02
0.0561421	0.0057713	0	0.0225959	0	0	0.0016775	0
0.1724354	0.0096931	0	0.071821	0	0	0	0
0.2025875	0	0	0	0.0602555	0	0.0170859	0.04
0.2757128	0.0166316	0	0.0426069	0	0	0.0003988	0
0.3117575	0	0.0012079	0.0975622	0	0	0.0111547	0.06
0.0683999	0.0244176	0	0.0615908	0	0	0.0018642	0
0.1008105	0.0097116	0	0.0394732	0	0	0	0.24
0.2955107	0	0	0.0733096	0	0	0.0029234	0
0.235636	0	0.0011056	0.0281554	0	0	0	0
0.1427988	0.0178851	0	0.0379806	0	0	0	0
0.1193666	0	0	0	0.0152057	0	0	0
0.1364977	0.0160955	0	0.1027914	0	0	0	0
0.1115077	0.004106	0	0.0212099	0	0	0.0012924	0
0.3503785	0	0.0055377	0.0456435	0.0059604	0	0.0138307	0
0.1197676	0.0173234	0.0023295	0.0479338	0	0	0	0
0.2288502	0	0	0.0339802	0	0	0	0.06
0.1979303	0	0	0.0328771	0	0	0.0169184	0
0.1884166	0	0.0050674	0.0873402	0	0	0	0
0.1004162	0.004675	0	0.0799978	0	0	0	0
0.2602224	0	0	0.0216866	0	0	0.0124175	0
0.2423816	0	0	0	0	0	0	0
0.3190785	0.0392239	0	0.0263723	0	0	0.0024889	0
0.1777018	0.0058922	0	0.0154778	0	0	0	0
0.3240757	0.0198883	0	0.0287024	0	0	0	0
0.1453629	0.001266	0	0.0450693	0	0	0	0.06
0.1055408	0.029171	0	0.1692749	0	0	0	0

0.1214079	0	0	0.0239069	0	0	0	0
0.256362	0	0.0020634	0.0501402	0	0	0.0074873	0
0.2430681	0	0.0023047	0.05335	0	0	0	0.02
0.0862388	0.0082834	0	0.0367259	0	0	0	0.02
0.2399416	0.0185388	0.0279917	0.1190538	0	0	0	0.44
0.2498539	0.0349122	0	0.0753845	0	0	0.004476	0
0.28282	0.0028319	0	0.0536923	0	0	0.016602	0
0.129381	0	0	0	0.0432325	0	0.0015559	0
0.1497615	0	0	0.0096153	0	0	0.0046892	0
0.1824801	0.0053639	0	0	0.0303813	0	0.0282961	0
0.0944867	0	0.0001054	0.0140698	0	0	0	0
0.2854198	0.0190494	0	0.059289	0	0	0	0
0.360247	0	0	0.1133933	0	0	0	0.02
0.2042838	0.000746	0	0.0053699	0	0	0	0
0.3076107	0.0005388	0	0.0257625	0	0	0.0002142	0
0.0919026	0.0008078	0	0.0043767	0	0	0	0
0.1178775	0.0017364	0	0.0373808	0	0	0	0.04
0.0867382	0.0067882	0.0010282	0.042497	0	0	0	0
0.182106	0.0164147	0	0.0478845	0	0	0	0
0.1285919	0.0920713	0	0.0253961	0	0	0	0
0.1618826	0.0230923	0	0.0411425	0	0	0	0
0.2400276	0	0	0.0281026	0	0	0.0001383	0
0.2240742	0.0292794	0	0.0356071	0	0	0.0115178	0
0.0873941	0.0050136	0.0007621	0.0223622	0	0	0	0
0.1736565	0.0074163	0.0005998	0.1257084	0	0	0.0137649	0.04
0.2406175	0	0	0.0524546	0	0	0.0072791	0
0.1084373	0.01335	0	0.0472735	0	0	0	0
0.2967163	0.0053267	0	0.0212586	0	0	0	0
0.1717063	0.0043689	0	0.0370866	0	0	0	0
0.1101045	0	0	0.0075637	0	0	0	0
0.0415112	0.0050758	0	0.0062166	0	0	0	0
0.1261531	0.0037203	0.0044619	0.0952428	0	0	0.0268919	0.22
0.2251059	0.0022591	0	0.0357634	0	0	0.0248398	0.02
0.0720879	0.0054881	0	0.0296059	0	0	0	0
0.2135892	0.0144015	0.001052	0.0732912	0	8.18E-05	0.0009452	0
0.2155518	0	0.0132899	0.0628379	0	0	0	0.1
0.2195102	0.0032312	0	0.0273106	0	0	0	0
0.2274064	0.0077698	0	0.0140073	0	0	0	0
0.1083302	0.0113065	0	0.0305677	0	0	0.0032959	0.04
0.168126	0.0171272	0	0.0403139	0	0	0	0
0.2046996	0.0165553	0	0.0466751	0	0	0.0092953	0
0.2396738	0	0.0446875	0.0156837	0	0	0.0416307	0
0.0636134	0.003677	0	0.0279989	0	0	0	0
0.1428034	0.0144299	0	0.0274531	0	0	0	0
0.097491	0.0119807	0	0.0100793	0	0	0	0
0.2503563	0.0039473	0	0	0.0068102	0.0001836	0.0073765	0
0.1770141	0.0261127	0	0.0700968	0	0	0.0001832	0
0.4627922	0.0028018	0	0.0641859	0	0	0	0
0.1280279	0.0043816	0	0.0143911	0	0	0.0093661	0
0.1355739	0.0473889	0	0.0134803	0	0	0	0
0.3315196	0.0066782	0	0.0539956	0	0	0.0002032	0

0.1791037	0.0043051	0	0	0.0487225	0.0019296	0.0013393	0.02
0.203723	0.0038689	0	0.0671155	0	0	0	0
0.1681982	0.0282689	0	0.0682379	0	0	0.0006955	0
0.2930539	0.0138684	0	0.0796747	0	0	0.0001336	0
0.2664079	0.0115013	0	0.0608043	0	0	0	0
0.2640587	0	0	0	0	0	0.0007814	0
0.1375385	6.32E-05	0	0	0	0	0	0
0.2092508	0	0	0.0376499	0	0	0.0017829	0
0.2551801	0	0	0.0027952	0.007252	0	0.0023822	0
0.2326593	0.0050311	0	0.0335807	0	0	0	0
0.1323191	0.0111438	0.0007902	0.0214405	0	0	0	0
0.0935757	0.0113914	0	0	0	0.0001437	0.001209	0
0.2355037	0	0	0.0230536	0	0	0.0063496	0
0.2979099	0	0	0.0249053	0	0	0	0
0.1225919	0.0225356	0	0.0227521	0	0	0	0
0.1144068	0	0.0006878	0.018938	0	0	0	0
0.2951874	0.0015861	0.0012756	0.0808297	0	0	0.0301518	0.04
0.1210326	0.5557876	0.0130357	0.0476732	0	0	0	0
0.0931762	0.0323223	0	0.0412042	0	0	0.0071944	0
0.2971398	0.0110325	0	0.0838631	0	0	0	0.54
0.1561271	0.0081328	0	0.0491432	0	0	0	0
0.2307301	0.0026927	0	0.0208819	0	0	0.0213606	0.02
0.2306442	0.0239301	0.0107717	0.1116718	0	0	0.0017793	0
0.1427537	0.0020179	0	0.0253461	0	0	0	0
0.1872583	0.001217	0	0.0230783	0	0	0.002011	0.02
0.2903814	0.0006363	0	0.0246283	0	0	0	0
0.2366069	0.0104903	0	0.0605083	0	0	0.0169439	0
0.1843262	0.0105119	0	0.0383303	0	0	0.0009376	0.08
0.1912209	0	0	0.031772	0	0	0	0
0.339408	0.0205504	0.0793892	0	0.0064353	0	0.0069424	0
0.1391318	0.0465012	0.0085001	0.053745	0	0	0.004462	0
0.1870168	0	0.0079772	0.1153413	0	0	0.037932	0.02
0.2096033	0.004301	0.0004057	0.0853033	0	0	0	0
0.2472814	0	0.0069933	0.0641053	0	0	0.0116424	0.6
0.1891071	0.0073795	0	0.0322926	0	0	0	0
0.373485	0.0072791	0	0.0341508	0	0	0.0132533	0
0.3705651	0.0399912	0	0.0426211	0	0	0.0020187	0
0.1889572	0	0	0.0263491	0	0	0	0
0.3086075	0.0439433	0	0.1286304	0	0	0.0152956	0.46
0.1618399	0.0012357	0	0.0159307	0	0.0015674	0.0050375	0
0.2888794	0.0280605	0	0.0902977	0	0	0.0292207	0.1
0.3629034	0	0	0.0231319	0	0	0.0037764	0
0.2159568	0.0282321	0.0043244	0.126271	0	0	0.0177316	0.06
0.1493645	0.0352115	0	0.0269944	0	0	0.004758	0
0.2215074	0.0180888	0.0030707	0.0697217	0	0	0	0.04
0.1528948	0	0	0.0285066	0	0	0	0.08
0.1433734	0.0308378	0	0.1114389	0	0	0	0
0.2552007	0.0011	0	0.0378416	0	0	0.0131495	0
0.0975546	0.0063204	0	0.0346854	0	0	0.0059084	0
0.1440139	0.0378611	0.0113767	0.0705436	0	0	0.0131942	0.08
0.2770292	0.0229046	0	0.0160101	0	0.0012348	0.0034068	0

0.3040093	0.0130341	0	0	0.0184375	0.0178772	0.0429787	0
0.1348977	0.0094826	0	0.0286926	0	0	0	0
0.0880818	0.0219749	0	0.0568707	0	0	0	0
0.2090795	0	0	0.0253457	0	0	0	0
0.4001193	7.77E-05	0	0.0290247	0	0	0.0016637	0
0.2724648	3.71E-05	0.0007519	0.0480154	0	0	0	0.42
0.1284598	0.001592	0	0	0	0	0	0
0.3427851	0	0.0014212	0.1043204	0	0	0.0187656	0.32
0.4659824	0.023381	0.0115041	0.0231334	0	0	0.0155352	0
0.2308668	0.0003758	0	0.0437623	0	0	0.0089035	0
0.2401174	0.0071129	0	0.0048775	0	0	0.0042768	0.04
0.3049704	0.0038101	0	0.0606336	0	0	0.0108156	0
0.1693117	0	0.0030085	0.0265543	0	0	0.0019222	0
0.2031811	0	0.0012463	0.0539271	0	0	0.0024347	0
0.113796	0.0062266	0	0.0230675	0	0	0.0072702	0
0.2026785	0	0.0031509	0.052975	0	0	0	0
0.1665042	0.0082836	0	0	0.0384482	0	0.0581622	0
0.2327832	0	0	0.0434857	0	0	0.0011531	0
0.1942854	0.0031192	0	0.0547423	0	0	0.0001342	0
0.2604688	0.0009929	0.0050329	0.0216862	0	0	0	0.04
0.2183163	0.0586356	0	0.0508846	0	0	0	0
0.1032077	0.0131672	0	0.1821288	0	0	0.0024437	0
0.2476552	0	0	0.0119275	0	0	0.0031079	0
0.3364506	0.0024466	0	0.0303396	0	0	0	0.22
0.1607097	0.0386777	0	0.0372863	0	0	0	0
0.1603947	0.0384295	0	0.0971265	0	0	0.05275	0.28
0.1564108	0.0466824	0.0074907	0.0188839	0	0	6.99E-05	0
0.2657666	0.0159877	0	0.0261053	0	0	0.0120406	0
0.3058207	0.0912508	0	0.072479	0	0	0.0068017	0.06
0.1521054	0	0.0054838	0.0422725	0	0	0.0053844	0
0.170764	0	0	0.0177206	0	0	0	0.02
0.2188602	0	0.0022904	0.0400129	0	0.0128848	0	0.04
0.2311577	0	0.020676	0.0444533	0	0	0.0068808	0
0.2015349	0.0151078	0	0.0221421	0	0	0.0129514	0
0.1228747	0	0	0.2592824	0	0	0.000798	0.38
0.2099208	0.0097664	0	0.04134	0	0	0.0041248	0
0.2717322	0.0373341	0	0.0229156	0	0	0.0014554	0
0.177289	0.014682	0	0.0049686	0	0.0016723	0.0026682	0
0.1604653	0	0.005594	0.1926509	0	0	0.0120191	0.48
0.1533652	0	0	0.0040914	0	0	0.0164406	0.02
0.3433751	0	0.0072959	0.0416737	0	0	0	0.7
0.1761412	0.0121221	0.0142963	0.0454727	0.0137494	0	0.022896	0.22
0.3567366	0.0017014	0	0.0801716	0	0	0.0027103	0
0.253064	0.0487101	0	0.0877932	0	0	0.0264342	0
0.3070822	0	0.0623227	0.0465608	0	0	0.000875	0.1
0.1137276	0	0	0.0094977	0	0	0	0
0.1615369	0	0	0.0135713	0	0	0	0
0.0896645	0	0	0.0370347	0	0	0	0
0.0403635	0.0166547	0.0221196	0.1333399	0	0	0	0.28
0.1476439	0.0355267	0	0.1022439	0	0	0	0
0.1667808	0.02818	0	0.0568498	0	0	0	0

0.1414837	0	0	0.0777879	0	0	0	0
0.2002602	0.0117274	0	0.0809743	0	0	0	0
0.2751608	0.0326972	0	0.0863564	0	0	0.0039729	0
0.3077097	0.0095587	0.0039322	0.0921728	0	0	0.0058553	0.06

Correlatic	RMSE
0.1524796	1.0272812
0.4003609	0.9232233
0.1421937	1.030805
0.3315195	0.9517732
0.2327056	0.9909517
0.1891681	1.0052154
0.0673588	1.0519275
0.2819723	0.9774782
0.3070135	0.9621062
0.1583743	1.02004
0.0878302	1.0570759
0.1473245	1.0391143
0.3031009	0.9616324
0.3035633	0.9651668
0.2160311	0.9944889
0.287095	0.9637966
0.0145329	1.0867075
0.3138916	0.963241
0.1316659	1.0377962
0.0803683	1.0575831
0.3579145	0.9345627
0.3705153	0.9299464
0.3395494	0.9471438
0.2334042	0.9857967
0.3351022	0.9531015
0.096663	1.0462262
0.1932613	1.0105173
0.1492842	1.0284682
0.3758913	0.9309196
0.2031314	1.0018291
0.2099287	1.0026214
0.2020329	1.0024096
0.0965976	1.0446579
0.39873	0.9245409
0.2431959	0.992758
0.2307836	0.9875588
0.8465913	0.5440205
0.2284392	0.9888623
0.2506967	0.981097
0.291172	0.9639777
0.2107925	1.0131086
0.0278414	1.0737194
0.0582481	1.0571116
0.299871	0.9616394
0.1163916	1.0415324
0.3971725	0.9279582
0.2996617	0.9596821
0.2895672	0.9696166
0.0922361	1.0385331

0.1378919	1.0262127
0.0424531	1.0691022
0.1675409	1.0361165
0.1403327	1.0339427
0.5385671	0.8421984
0.0144194	1.0719247
0.2992914	0.9664725
0.464603	0.8930636
0.3064415	0.959
0.0224975	1.0708371
0.3453369	0.9553042
0.2170717	1.00263
0.0165721	1.0773826
0.0037723	1.0855114
0.3196092	0.9547946
0.0837946	1.0613938
0.0014985	1.0826096
0.2731014	0.9748498
0.4401813	0.9068734
0.1767936	1.025161
0.4615668	0.9070876
0.3023624	0.9667237
0.2538969	0.9994082
0.2319752	0.993184
0.1009888	1.0575295
0.1004886	1.0376933
0.082132	1.0467877
0.2399745	0.9918681
0.1035283	1.0353763
0.3472242	0.9416782
0.3036379	0.9802427
0.1703913	1.0342474
0.358285	0.9367366
0.0935315	1.0807022
0.2344604	1.0110136
0.3898859	0.9303886
0.3528047	0.9467777
0.3335214	0.9559934
0.2593127	0.9851377
0.3053344	0.9654369
0.3949984	0.9374098
0.2553635	1.0125371
0.1658756	1.0276791
0.3129452	0.9594483
0.1724699	1.0406829
0.4632575	0.890785
0.2830834	0.9807348
0.3244929	0.9599418
0.1804637	1.0277951
0.2448514	0.9898869
0.0896121	1.0455222

0.1689518	1.0421359
0.2669222	0.975776
0.1610362	1.0950075
0.4503414	0.8996986
0.4057486	0.9243332
0.4603041	0.8880663
0.2109624	1.0155426
0.3402233	0.9455128
0.1508716	1.0607185
0.3804092	0.9278387
0.1559148	1.0428593
0.4028747	0.9214232
-0.020445	1.0975359
0.232861	0.9999198
0.531767	0.847067
0.357747	0.9771055
0.2171645	0.996256
0.3133717	0.9640908
0.3034314	0.9600098
0.2291887	0.9947382
0.1902123	1.0237625
0.0157904	1.0875852
0.2817112	0.9886252
0.4357833	0.9079595
0.2746776	0.9753054
0.1771542	1.0171673
0.1644679	1.0227886
0.1826429	1.0239646
0.4184746	0.9094138
0.2921587	0.9689187
0.1867584	1.021529
0.1904831	1.0304359
0.3745655	0.9297798
0.1612933	1.0288031
0.044444	1.0935622
0.3641207	0.9335499
0.5687913	0.8240302
0.1474335	1.0349161
0.2389412	0.9989458
0.2654169	0.9822395
0.4175168	0.9102086
0.0524991	1.0973896
0.0006984	1.0911185
0.1618225	1.0214597
0.2611164	0.9840517
0.1272118	1.0330491
0.2820061	0.9743824
0.2470666	1.0189366
0.37464	0.9293923
0.130439	1.0465992
0.0738294	1.0567324

0.1989129	1.0119363
0.2345579	0.9902663
0.0881902	1.0649681
0.0298413	1.0744933
0.1006024	1.0757664
0.2518866	0.9842729
0.2636835	0.9876548
0.3700502	0.9309692
0.2584307	0.9878955
0.3080409	0.9656519
0.2332479	0.984766
0.3671221	0.9317567
0.4723128	0.8933693
0.1168168	1.0308121
0.2569722	0.9763482
0.0637159	1.06568
0.2029146	1.008097
0.4337896	0.9012011
0.2557008	0.9815912
0.230521	0.9942012
0.0187619	1.0859189
0.2169053	0.9990959
0.4160611	0.9090234
0.3783804	0.9511896
0.0976268	1.0614818
0.5087096	0.8627542
0.2885304	0.9675055
0.304421	0.9612505
0.3714574	0.9309192
0.2003299	1.0014314
0.1071304	1.0793646
0.1945524	1.03282
0.1147249	1.0424928
0.0268859	1.0755986
0.3298779	0.9654469
0.1821959	1.0288162
0.3604027	0.9349219
0.1664506	1.0235195
0.1468113	1.0282016
0.2786894	0.9727301
0.2695987	0.9778562
0.1341648	1.0239157
0.2317883	0.9850982
0.4645261	0.8866498
0.2214339	0.9959101
0.0530738	1.0754245
0.0561197	1.0587487
0.0959383	1.0451407
0.2325414	0.9978655
0.1305117	1.0318411
0.0784984	1.058689

0.2303448	0.993737
0.0930246	1.0516506
0.1630785	1.014591
0.3209833	0.9584073
0.2548429	0.9868022
0.2812168	1.0039963
0.1159359	1.0402514
-0.003021	1.0962677
0.2875697	0.9740423
0.1810419	1.0299675
0.297815	0.9621318
0.278162	0.9761952
0.2971615	0.9616584
0.2489139	0.984476
0.1405106	1.057172
0.1918146	1.0108131
0.2498786	0.9903824
0.4353531	0.9061942
0.3125691	0.9567491
0.3129905	0.9531384
0.0669225	1.0700498
0.2755145	0.9916804
0.4277759	0.9033321
0.0930555	1.0505422
0.3610702	0.9346416
0.3076608	0.9612536
0.3876896	0.9409166
0.1260478	1.0294778
0.2181319	0.9968908
0.4993348	0.8662739
0.2509613	1.0111893
0.3961912	0.9188534
0.1128543	1.0664894
0.0842685	1.0526969
0.2726452	0.9689538
0.0385178	1.0777567
0.3385571	0.9449588
0.4436149	0.904523
0.2430319	0.9906063
0.258984	1.0064183
0.2541087	0.9794582
0.0970579	1.0532277
0.1661124	1.049127
0.32499	0.9489495
0.4241074	0.9068092
0.2860163	0.9704848
0.1845362	1.0209796
0.4607764	0.8933462
0.3489378	0.9382437
0.3039542	0.9609493
0.1053732	1.0466351

0.2930285	0.9888445
0.1696722	1.0118425
0.1090328	1.0496533
0.1900244	1.0076154
0.342565	0.9511919
0.2469792	0.9887639
0.0529222	1.0576277
0.4548905	0.8932617
0.1663772	1.0145741
0.1220337	1.0436706
0.0862194	1.047353
-0.012555	1.0862609
0.3519239	0.9396368
0.5593741	0.8307178
0.4168971	0.9291894
0.0469435	1.0753732
0.0858111	1.0721621
0.0984601	1.0620437
0.0028021	1.0931901
0.5577161	0.8331821
0.1303458	1.0264796
0.1786165	1.0199501
0.1629489	1.0682497
0.3167207	0.9584683
0.2674372	1.0132095
0.2510649	0.9941859
0.3431946	0.9393775
0.1835522	1.019289
0.1180507	1.0416416
0.1622192	1.0295745
0.097449	1.0632438
0.0627463	1.064852
0.605933	0.8049288
0.5855476	0.8120457
0.0806163	1.0715119
0.0833797	1.0574217
0.1772428	1.0356581
0.4992639	0.865793
0.3559898	0.9401803
0.3867163	0.9230259
0.1883782	1.0045763
0.3757603	0.9274424
0.1039112	1.042104
0.2004629	1.0150583
0.2672433	0.9703151
0.3569662	0.9370853
0.2033439	1.0252576
0.1917767	1.0189414
0.4446451	0.8954601
0.2327543	0.9929226
0.4878965	0.8721579

0.3087377	0.9651309
0.2749067	0.9720008
0.0964942	1.0492001
0.1360755	1.0299933
0.3431318	0.9462372
0.2950206	0.9631676
0.2814752	0.9758055
0.1971766	1.0031424
0.109077	1.0415977
0.4333074	0.9070122
0.0968905	1.0468008
0.3023291	0.9632493
0.2074592	1.0047864
0.4390787	0.8987798
0.2529131	0.9903657
0.1029907	1.0614962
0.2289892	0.9865554
0.2032368	1.0038909
0.246685	0.9950918
0.249909	0.9819763
0.0932547	1.0792733
0.1448486	1.0271527
0.2121513	1.0051251
0.5682124	0.8223011
0.1529861	1.0265234
0.3443708	0.9579459
0.2948875	0.975092
0.1290369	1.0408819
0.2401906	0.9896751
0.1201037	1.0318633
0.3763333	0.9384772
0.0658683	1.1086831
0.5020571	0.8657529
0.2832816	0.9803683
0.1650119	1.0348176
0.2531286	0.9935185
0.2011952	1.0145471
0.4379156	0.9088157
0.2577237	0.9790196
0.3991492	0.9302736
0.1111658	1.0465652
0.2536687	0.989682
0.1762532	1.019173
0.2429952	0.9947606
0.3430918	0.9422489
0.2260207	0.9983024
0.4000668	0.9167516
0.3213102	0.9604794
0.1700158	1.0222772
0.120895	1.066768
0.3033717	0.9623947

0.2474433	1.0011043
0.2702739	0.9717687
0.1497858	1.0316852
0.1514824	1.0423165
0.0244238	1.0737466
0.2375702	0.9840151
0.2405236	0.9858676
0.3274668	0.9648538
0.2915682	0.9708474
0.166955	1.0305717
0.3088222	0.9841851
0.167526	1.0170664
0.1525604	1.0193834
0.4045417	0.9216276
0.4473472	0.8937576
0.3125908	0.9788933
0.1362703	1.054001
0.4259362	0.9182478
0.410973	0.9135545
0.4540406	0.8903078
0.255554	0.9859212
0.3762102	0.9314436
0.4704188	0.8824756
0.5074849	0.8655037
0.1394292	1.0227507
0.2285627	0.9946085
0.3960803	0.91875
0.2948794	0.9637772
0.3422529	0.9578589
0.4614674	0.905989
0.2406836	1.0395132
0.0742169	1.0607999
0.1506852	1.0235973
0.3427571	0.9503753
0.2161237	1.0038199
0.0927924	1.0478035
0.3513178	0.9463004
0.4714673	0.8818793
0.1313616	1.0553502
0.3283657	0.9508267
0.3090223	0.9577352
0.2070996	1.0041672
0.5199248	0.8626658
0.2720409	0.9903994
0.2639914	1.0108159
0.3513964	0.9396828
0.2631497	0.9809316
0.2150157	1.000041
0.3050828	0.9662345
0.2843611	0.9831173
0.2528312	0.9836259

0.1527409	1.0457801
0.3950598	0.921415
0.1852346	1.0145581
0.1896308	1.0049844
0.2137852	1.0008347
0.1944342	1.0102311
0.4739164	0.8885882
0.3835949	0.9236712
0.2149306	0.9981699
0.2534608	0.9833495
0.3765365	0.9401274
0.5456002	0.8419389
0.2572583	0.9834866
0.3365124	0.9546393
0.2784712	0.9812197
0.2422754	1.0258678
0.1336667	1.0305463
0.3257866	0.9624482
0.3156291	0.9639188
0.0056295	1.0886853
0.4116419	0.9177543
0.1578695	1.0253636
0.2562119	0.9790741
0.5436937	0.8392689
0.1569809	1.0416424
0.2819917	0.9693862
0.35558	0.9361748
0.1001755	1.0674539
0.1613891	1.0254799
0.2206025	1.0015144
0.1690028	1.0293258
0.1484668	1.0255741
0.2408658	0.9849698
0.0003866	1.0856141
0.4469437	0.8964027
0.2906851	0.9667616
0.2830101	0.9679224
0.4050706	0.917303
0.0150869	1.0696709
0.483724	0.8750681
0.0910649	1.0410464
0.4084418	0.912452
0.104828	1.0377205
0.3440439	0.9449816
0.1419698	1.0336628
0.0994558	1.0568991
0.2359556	0.9957385
0.2022347	1.0023682
0.3989782	0.91905
0.1027219	1.0416572
0.2199711	1.0033135

0.4229319	0.9058544
0.3636843	0.9489659
0.4260173	0.919632
0.3790307	0.9329609
0.1919624	1.0164555
0.0297577	1.0887865
0.4608705	0.8924196
0.0446098	1.0655256
0.4320672	0.9042602
0.1765423	1.033496
0.1346022	1.0337864
0.2783196	0.9701538
0.3004411	0.9680838
0.2479992	0.9987875
0.160234	1.0387556
0.2667333	0.9766083
0.2618035	0.9798153
0.2734371	0.9880791
0.3141736	0.959123
0.1424966	1.028479
0.4344442	0.9000002
0.1679307	1.0189553
0.2433037	0.9986045
0.0740599	1.0658277
0.246813	0.9880934
0.0583373	1.0568053
0.3068739	0.9597465
0.2830133	0.967559
0.1190397	1.0341248
0.1831827	1.0168973
0.1567044	1.0334055
0.1451793	1.032634
0.2039534	1.0025192
0.1808448	1.0196816
0.0342064	1.0718486
0.2305199	0.9931149
0.4279967	0.9029772
0.2268774	1.0104677
0.012706	1.0747939
0.1555995	1.0375668
-0.005257	1.0884442
0.0720862	1.0617268
0.1677431	1.0176372
0.2017584	0.9961729
0.0915208	1.0483651
0.3541015	0.956148
0.2530396	0.9830974
0.1877671	1.0563261
0.0553673	1.0833665
0.1885111	1.0136452
0.3386397	0.9428887

0.2293295	1.0112696
0.2354971	0.9877334
0.1614542	1.0179983
0.1171915	1.0372178
